# Multi-kingdom microbial assemblage modulates its metabolism under contrasted cloud conditions

**DOI:** 10.1093/ismeco/ycaf200

**Published:** 2025-11-03

**Authors:** Domitille Jarrige, Jonathan M Vyskocil, Muriel Joly, Binta Dieme, Marie Lagrée, Emilie E L Muller, Pierre Amato, Françoise Bringel

**Affiliations:** Génétique Moléculaire, Génomique, Microbiologie (GMGM), Université de Strasbourg, UMR 7156 CNRS, Strasbourg, France; Université Bourgogne Europe, Institut Agro Dijon, INRAE, Agroécologie, Dijon, France; Institut de Chimie de Clermont-Ferrand (ICCF), Université Clermont Auvergne, UMR 6296 CNRS-INP, Clermont-Ferrand, France; Department of Global, Environmental, and Occupational Health, School of Public Health, University of Maryland, College Park, Maryland, United States of America; Institut de Chimie de Clermont-Ferrand (ICCF), Université Clermont Auvergne, UMR 6296 CNRS-INP, Clermont-Ferrand, France; Université Clermont Auvergne, INRAE, UNH, Plateforme d’Exploration du Métabolisme, MetaboHUB Clermont, Clermont-Ferrand, France; Université Clermont Auvergne, INRAE, UNH, Plateforme d’Exploration du Métabolisme, MetaboHUB Clermont, Clermont-Ferrand, France; Génétique Moléculaire, Génomique, Microbiologie (GMGM), Université de Strasbourg, UMR 7156 CNRS, Strasbourg, France; Institut de Chimie de Clermont-Ferrand (ICCF), Université Clermont Auvergne, UMR 6296 CNRS-INP, Clermont-Ferrand, France; Laboratoire: Microorganismes, Génome et Environnement (LMGE), Université Clermont Auvergne UMR 6023 CNRS, Clermont-Ferrand, France; Génétique Moléculaire, Génomique, Microbiologie (GMGM), Université de Strasbourg, UMR 7156 CNRS, Strasbourg, France

**Keywords:** aerobiology, airborne microorganisms, cloud water, oxidative stress, metatranscriptomics, metabolomics, acyl carnitines, methionine sulfoxide

## Abstract

Microorganisms maintain metabolic activity in clouds, with recognized impacts on the chemistry of small organic compounds, radicals, and their precursors. However, how microbial activity is modulated by cloud environmental variables remains unknown. Here we explored the metabolic response of an assemblage of representative microbial isolates from cloud water, composed of a basidiomycetous yeast (*Dioszegia hungarica*) and three bacterial strains (*Rhodococcus enclensis*, *Pseudomonas syringae*, and *Pseudomonas graminis*), in synthetic cloud water exposed to contrasted conditions of temperature (5°C vs 17°C), light (dark vs artificial solar light) and oxidants (0 μM vs 250 μM H_2_O_2_), to mimic typical cloud conditions during winter night and summer day. Metabolomics and metatranscriptomics allowed the identification of 25 differentially abundant metabolites and 218 differentially expressed genes (DEGs). Both summer day metabolomes and metatranscriptomes suggested active mitochondria-driven energy production, with fungal DEGs involved in fatty acids biosynthesis and succinate assimilation, and three differentially abundant acylcarnitines that support fatty acid transport into the mitochondrion for oxidative phosphorylation. In contrast, bacteria displayed DEGs for cell division arrest and components of reactive oxygen species scavenging systems. Under the winter night condition, both bacteria and yeast exhibited a similar prosperous state with DEGs encoding translation, protein repair and turnover, as well as cell cycle related functions. Thus, eukaryotes and prokaryotes may engage in distinct strategies to survive in clouds, depending on environmental conditions. This study consolidates our understanding of microbial roles and interactions in cloud water, paving the way for deeper insights into the chemistry of atmospheric systems.

## Graphical Abstract

**Graphical Abstract:**
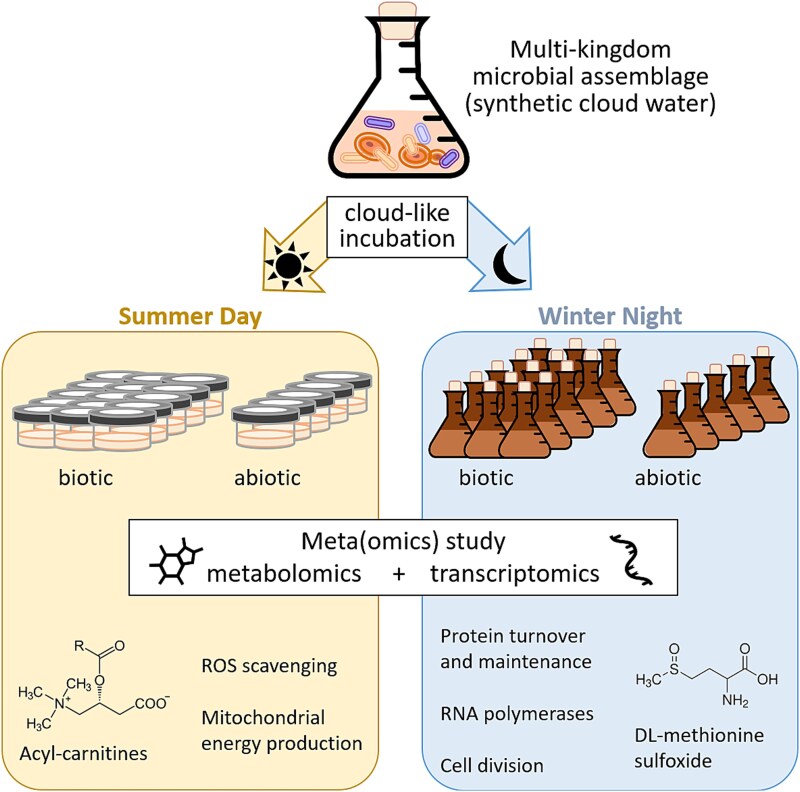

## Introduction

Clouds offer conditions for living cells to maintain metabolic activity. Distinctively to other environments on Earth, clouds contain low microbial biomass (10^3^–10^4^ bacterial and 10^2^–10^3^ eukaryotic cells·ml^−1^) in spatially partitioned droplets with a short residence time (e.g. a few hours at maximum) [1, 2]. These unique complex features limit the establishment of microbial ecological networks [1, 2] between phylogenetically diverse bacteria and fungi that are known to be viable and metabolically active in clouds. The most frequent microbial taxa in clouds include Proteobacteria, Actinobacteria, and Basidiomycetes [3]. In airborne conditions, both bacteria and yeasts utilize simple dissolved carbon compounds such as formate, acetate, and formaldehyde [4–6], at rates depending on strain and conditions such as temperature, concentration of oxidants (e.g. H_2_O_2_), radicals (e.g. HO_2_^•^ and HO^•^), and the level of UV radiations [1, 4, 7, 8].

Great progress has been made over the last years to better understand microbiological processes in clouds and their multiple drivers, using natural cloud communities and cultures of isolated bacteria and yeast strains [1, 2]. Metatranscriptomics revealed that multiple metabolic processes are triggered in airborne cells in clouds in comparison with clear atmosphere, due to the presence of liquid water [9], impacting the expression of pathways contributing to responses to thermal and oxidative stresses [10]. Metabolomic studies specified functional adjustments to low temperature and oxidants in bacterial strains isolated from clouds (*Pseudomonas syringae*, *Pseudomonas graminis*), and evidenced active pathways related to carbohydrate, glutathione, and energy metabolisms [11, 12]. Both low pH (4.3) and exposure to solar light were shown to alter the capacity of a strain of *Enterobacter* isolated from aerosols to biodegrade formate, oxalate, maleate, and malonate, with possible synergy between the two stress factors [6].

As in other ecosystems, environmental conditions are expected to have significant impacts on microbial metabolism, with possible consequences on the overall atmospheric chemical balances [6, 13–17]. A dual role for microorganisms in atmospheric chemistry has been proposed accordingly: on one hand, they utilize organic carbon species such as formaldehyde, and on the other hand, they reduce the available source of oxidants [H_2_O_2_ and reactive oxygen species (ROS)] through oxidative stress response metabolism [8]. The combined influence of intricate chemical reactions and multiple stresses (temperature, oxidants, nutrient availability) on the metabolic functioning of cloud microbial communities, including small eukaryotes, in a physical distancing context, remains poorly understood.

The aim of this study was to assess the metabolic functioning of microbial cells in clouds using experimental model conditions mimicking different environmental scenarios found at the site where microbial strains were isolated. This included contrasted conditions of temperature, light, and oxidants. We hypothesized that reconstructing in synthetic cloud water, with formaldehyde as the carbon source, a microbial assemblage at low cell concentration, composed of three representative bacteria and one yeast, will allow to observe and then decipher for the first time the typical microbial metabolic pattern under summer day (SD) and winter night (WN) clouds. To pinpoint the metabolic responses of the exposed microbial assemblage, the metabolic patterns were examined at designated time points (a few hours) through combined omics approaches (transcriptomics and metabolomics) with regards to H_2_O_2_ and formaldehyde concentrations. The results showed that the overall microbial assemblage displayed distinct responses to SD compared to WN conditions. Bacteria responded distinctly from the eukaryote, and some functions associated with differentially expressed genes (DEGs; metatranscriptomics) could be related with differentially abundant metabolites (metabolomics) between SD and WN conditions. This innovative work consolidates our knowledge regarding microbial functioning in cloud water and its modulations by environmental condition.

## Materials and methods

### Mixed cell suspensions, monitoring of biological and chemical variables in the microcosms, and sample processing

**Figure 1:**
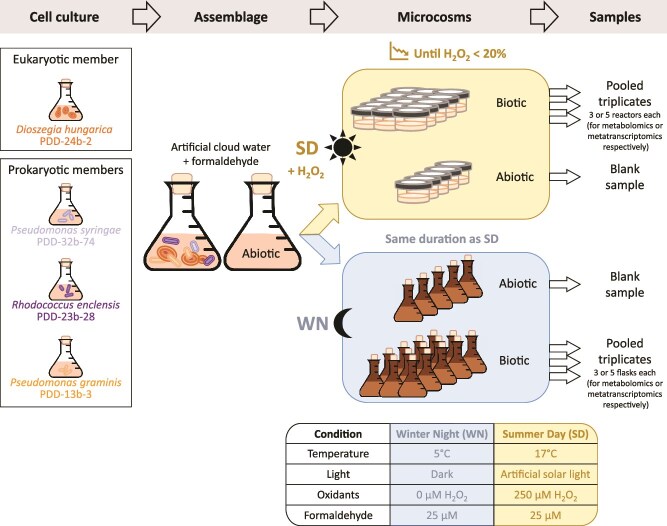
Experimental setup: microcosms of synthetic microbial assemblage incubated in WN or SD cloud-like conditions; each sample corresponds to five-pooled technical replicates and triplicates of biological samples were performed for each of the tested conditions.

The microbial assemblage consisted of four strains isolated from cloud water: *Pseudomonas syringae* (*Ps*) strain PDD-32b-74 [18], *P. graminis* (*Pg*) strain PDD-13b-3 [19], *Rhodococcus enclensis* (*Re*) strain PDD-23b-28 [20], and *Dioszegia hungarica* (*Dh*) strain PDD-24b-2 [21]. An overview of the experimental setup is presented in Fig. 1.

In order to simulate the very low interaction probability between several taxa (due to the natural sequestration of cells in the cloud droplets, in accordance to the current scientific literature body of cloud ecology [2]), we targeted a low richness for our assemblage. The selection of members from representative and frequent viable taxa [3] was based on the following criteria: (i) distinct taxonomies (i.e. bacteria vs eukaryote, Gram-positive vs Gram-negative bacteria), and on the contrary, closely related organisms (two *Pseudomonas* species); (ii) with growth rates and conditions compatible with experimental constraints; (iii) with fully sequenced genomes; and (iv) strains (or close representatives of them) that have been extensively characterized for their individual physiological responses to stresses and their metabolic capacities (e.g. response to cold and oxidants, utilization of carbon sources) [5, 8, 11, 12, 22–25].

For the preparation of cell suspensions, each of the four individual strain (characteristics detailed in Table 1) was grown from glycerol stock in liquid R2A medium at 17°C in aerobiosis (200 rpm agitation) until mid-log phase. Cells were harvested by centrifugation (8000 × *g*, 3 min) and rinsed twice in freshly prepared synthetic cloud water solution (Supplementary Table S1). Cells of each strain were then resuspended, as a mix, in freshly prepared synthetic cloud water solution at a final concentration of ~10^5^ cell·ml^−1^ based on OD_600nm_ (to mimic the low cell concentrations and thus low potential for cell interactions inferred in natural clouds [2]), and an aliquot was collected for precise cell counts by flow cytometry (see below). The concentration of each strain in the initial assemblage was also evaluated by plate-count of colony forming units (CFU) of the mixed cell suspension on R2A medium incubated 6 days at 17°C (strains have different phenotypes and colours and are easily distinguishable visually from each other). Incubations of the 40 ml mixed microbial cell suspensions were realized under agitation (200 rpm) in microcosms consisting of dark Erlenmeyer flasks at 5°C in the dark (WN condition) or in custom-made quartz lidded photo-bioreactors providing artificial solar light irradiation, at 17°C and with addition of 250 μM H_2_O_2_ (SD condition). To mimic the high oxidative stress encountered by airborne microorganisms in the SD cloud-like condition, a combination of H_2_O_2_ supplementation and high-light exposition was used to increase the level of oxidants and ROS generated, as previously described [26]. Note that in absence of high-light exposure generating high level of ROS species, a concentration of 250 μM H_2_O_2_ is far below concentrations impacting bacterial survival [27], although higher than the H_2_O_2_ concentrations typically reported in cloud water (up to ~20 μM) [28]. Abiotic controls consisted of similar 40 ml solutions incubated in parallel in the absence of cells. Two batch of experiments were performed, one for the metabolomic and another for the metatranscriptomic analyses.

**Table 1:** Composition and characteristics of the synthetic cloud assemblage.

Strains	(Kingdom) Phylum	Class	Species	Genome size in Mbp and number of predicted CDS*^a^*	Genome BioProject accession n°	Reference for isolation and genome	Generation time at 17°C in R2A (h)	Cloud isolation events (%)^b^	CFU ml^−1^^c^ Meta-metabolomics	CFU ml^−1^^c^ Metatranscriptomics
PDD-13b-3	(Bacteria) Pseudomonadota	Gammaproteobacteria	*Pseudomonas graminis*	6.02 *(5795)*	PRJNA362581	[19]	2.0	58	5.0 × 10^5^	1.3 × 10^5^
PDD-32b-74	(Bacteria) Pseudomonadota	Gammaproteobacteria	*Pseudomonas syringae*	5.71 *(5324)*	PRJNA362578	[18]	3.1	58	3.1 × 10^6^	7.2 × 10^5^
PDD-23b-28	(Bacteria) Actinomycetota	Actinomycetes	*Rhodococcus enclensis*	7.22 *(7280)*	PRJNA395791	[20]	5.6	29	8.0 × 10^5^	6.1 × 10^5^
PDD-24b-2	(Fungi) Basidiomycota	Tremellomycetes	*Dioszegia hungarica*	20.98 *(8234)*	PRJNA809585	[3, 21]	6.3	70	3.0 × 10^5^	2.5 × 10^5^

a Size of the genome in Mbp and corresponding number of predicted CDS in parentheses and italic script.

b Average frequency of isolation of the corresponding genus from cloud samples at puy de Dôme Mountain station, as reported in [3].

c CFU ml^−1^ for the corresponding series of experiments, as determined by plate-count on R2A agar medium.

Monitoring of the microcosms during incubations included cell counts, H_2_O_2_ and formaldehyde quantification at specific times (just before incubation (T_0_), and after 1, 2, 3, 4, 5 and 24 h of incubation), from dedicated biological triplicates of microcosms per condition. For cell counts by flow cytometry, triplicate aliquots of 450 μl were collected and mixed with 50 μl of 5% glutaraldehyde, then stored at 4°C until analysis. Before analysis, the cell suspensions were buffered with 50 μl of Tris-EDTA (40 mM Tris-base, 1 mM EDTA, acetic acid to pH 8.0), then stained with 5 μl of 100X SYBR Green I (Molecular Probes Inc. Eugene, OR), and incubated for 15 min in the dark. The analyses were performed using a cytometer (LSR Fortessa, Becton Dickinson, Franklin Lakes, NJ, USA). Hydrogen peroxide and formaldehyde concentrations were quantified by fluorimetric assays following the methods in [13]. When less than 20% of H_2_O_2_ remained in the biotic SD microcosms (i.e. ~3.5 h for the meta-metabolomic experiment and 4 h for the metatranscriptomic one), the incubation for biological analyses was stopped (T_F_) and the cells were collected for metabolite and transcript extraction (Supplementary Fig. S1A). For the meta-metabolomic approach, the cells from three-pooled microcosms (i.e. 120 ml total volume per replicate) were centrifuged (10 000 × *g*, 4 min), and the pellets were stored at −80°C after rinsing with sterile 0.8% w/v NaCl solution, and the supernatants discarded. Metabolites were extracted with 1.2 ml of a cold solvent mixture of water/methanol/acetonitrile [1:2:2], as in [12]. The tubes were then immersed in liquid nitrogen, thawed in ice and thoroughly vortexed before centrifugation (10 min at 10 000 × *g*). After supernatant evaporation, the metabolites were stored as dry extracts.

For metatranscriptomics, triplicates series of five-pooled microcosms (i.e. 200 ml total volume per replicate) were individually filtered through 0.22 μm porosity membranes of mixed ester cellulose (ref. 0421A00023; ClearLine®, Bernolsheim, France). Blanks were treated similarly and in parallel. The membranes were then independently rolled (bottom side out) and placed into 5 ml bead-beating tubes (ref. 740799.50; Macherey–Nagel, Hoerdt, France), added with 1.2 ml of MR1 lysis buffer (ref. 744351.125; Macherey–Nagel), and subjected to bead-beating for 10 min using a Genie2 vortex, and then stored at −80°C until nucleic acid extraction. Total nucleic acids were extracted from lysate using NucleoMag DNA/RNA Water (Macherey–Nagel) kits following the manufacturer’s recommendations, adapted for 47 mm diameter membranes as in [29]. Bacterial ribosomal RNAs were depleted from the extracts using MICROBExpress™ Bacterial mRNA Enrichment Kit (Invitrogen). After DNA digestion with the turbo DNA-free kit (Invitrogen), nucleic acids were quantified (Qubit high-sensitivity DNA and RNA kit, Invitrogen) and then quality-checked using high-resolution automated electrophoresis (Bioanalyser, Agilent). The purified RNAs (100 ng) were then further treated using the Repli-g WTA single cell kit (Qiagen), as recommended by the manufacturer, which included an extra step of DNA removal, followed by reverse transcription for cDNA production and subsequent multiple displacement amplification. GenoScreen (Lille, France) performed library preparation (Illumina DNA Prep kit) and sequencing (Illumina PE 2 × 300 bp; ~4 million reads per sample).

### Untargeted meta-metabolomics and data (pre)processing

The dry metabolite extracts were resuspended in 50 μl of 50/50 acetonitrile/water (v/v) added with 0.1% formic acid. Extracts (5 μl) were injected in an UHPLC Ultimate 3000 system (Thermo FisherScientific) coupled to a TimsTOF mass spectrometer (Bruker) equipped with an electrospray (ESI) source and operating in the positive ion mode. The chromatographic separation was performed using an Acquity HSST3 column (2.1 × 150 mm; 1.8 μm-Waters) operating at 30°C. The flow rate was fixed at 0.4 ml/min with 0.1% of formic acid in water (A) and 0.1% of formic acid in acetonitrile (B) as the mobile phases at the following gradient: (i) initial 0–2 min, 100% A, (ii) 2–15 min linear gradient to 0% A, (iii) 15–22 min, 0% A, and (iv) final 22–22.10 min linear gradient to 100% A. This was followed by a 4 min-long step of washing, reconditioning, and equilibration of the column with 100% A. The detection was performed with full scan from m/z 50 to 1000 with capillary voltage at 4 kV and a capillary temperature set at 200°C. Data Analysis software (version 4.1) was used for data acquisition and analysis. The metabolites were first identified using an in-house database containing the reference spectra of >1000 standard compounds analysed using similar analytical conditions as ultra-performance liquid chromatography-mass spectrometry (UPLC-MS) profiling. Database queries were performed with a mass error of 0.005 Da and a retention time difference of 0.3 min. Database results were confirmed using appropriate standards when available, isotopic patterns and mass fragmentation analyses using collision induced dissociation MS2 data acquired on the same sample. Metabolites were classified accordingly to [30] concerning the levels of confidence in the identification process.

For data (pre) processing, raw data were converted to network common data form (NetCDF) in order to process them using the web-based platform Galaxy Workflow4Metabolomics (W4M) framework [31]. XCMS software (version 3.12.0) implanted in the W4M platform was used to preprocess the raw data for feature detection, alignment, and grouping, and missing peak filling [32]. After preprocessing, in order to remove unreliable peaks, the data table from ultra-high-performance liquid chromatography high-resolution mass spectrometry (UHPLC-HRMS) (UHPLC-ESI(+)-HRMS mode) was subjected to some filters: the ions eluted at the beginning (before 0.4 min) and at the end (after 22 min) of chromatographic separation were removed, as well as those whose intensity was lower than four times that in the negative control samples injected at the beginning of the batch. In order to correct for instrumental drift during batch analyses, for each peak, a regression model was fitted to the intensities of pool samples and used to adjust the intensities in biological samples [33]. Finally, the stability of signal intensities across the batch was evaluated by calculating the relative standard deviation of each peak in pools (mix of all biological samples), and those higher than 30% were excluded. The intensities of remaining features were normalized to the total spectra area.

### Metatranscriptomics data analysis and differentially expressed gene annotation

The metatranscriptomics sequences were processed using a custom Snakemake-based [34] workflow (Supplementary Fig. S2). Paired-end reads were quality checked using FastQC v0.11.9 (Andrews, Braham Bioinformatics) and MultiQC v1.13 [35]. Reads were cleaned and trimmed using fastp v0.23.2 [36] with the following parameters: -qualified_quality_phred- 25; -unqualified_percent_limit- 15; -cut_mean_quality- 25; -cut_window_size- 8; -cut_front-; -cut_tail-; -length_required- 130; -correction. Remaining rRNA reads, in particular fungal sequences, were filtered *in silico* with SortMeRNA v4.3.6 [37] using identified rRNA sequences from the previously sequenced genomes (Table 1). The obtained filtered non-rRNA paired-end reads were mapped against the reference genomes using STAR v2.7.10a [38] (--outFilterScoreMinOverLread 0.02; --outFilterMatchNminOverLread 0.02; --outFilterMatchNmin 0; --outFilterMismatchNmax 10; --alignEndsProtrude 10 ConcordantPair; --peOverlapNbasesMin 1; --outFilterMultimapNmax 10; --outSAMprimaryFlag AllBestScore). Counts for each gene of the assemblage were obtained using featureCounts [39] from subread package v2.0.1 [40] (with g “ID”;- -M; -O; --fraction; -byReadGroup-; -p; -t “exon, CDS, misc_RNA, ncRNA, rRNA, tRNA, tmRNA, mRNA”; extraAttributes “-gene, product, product_name, Name, locus_tag, transcriptId-”). Read count matrixes were then statistically analysed to detect DEGs (see statistical analysis below). Controls consisting of the abiotic microcosms (blank samples) were treated in the same way as biological samples.

Each DEG annotation was curated using a combination of automated combination of automated annotation of Clusters of Orthologous Genes (COG/KOG), predicted protein sequence analysis with CD search [41] and BLAST web server (accessible at https://blast.ncbi.nlm.nih.gov/Blast.cgi) [42], the JGI Mycocosm platform [43] (accessible at https://mycocosm.jgi.doe.gov/Diohu1/Diohu1.info.html) for the genome of *D. hungarica* PDD-24b-2 [21], and MicroScope (the Microbial Genome Annotation and Analysis Platform, accessible at https://mage.genoscope.cns.fr/microscope/home/index.php? [44] for the bacterial genomes.

### Statistical analyses

Statistical analyses were performed from read count or metabolite matrixes using R v4.3.1 [45] in the RStudio software v2023.09.1-494 [46]. Plots were generated with ggplot2 v3.4.3 [47] unless stated otherwise. A detailed R Markdown report is available at (https://zenodo.org/records/16754027). Metatranscriptomic blank samples were set aside. Zero count (undetected) genes or metabolites in more than 70% of the biological samples were considered unexpressed/unreliable and taken out of the statistical analyses. A centred log ratio transformation (CLR) [48] was applied to both transcript and metabolite counts for normalization prior to principal component analyses (PCA) and hierarchical clustering. To detect DEGs between SD and WN conditions, two methods with their own integrated normalization procedures were used on untransformed filtered data: MTXmodel v1.2.4 [49] (normalization = “CLR”, analysis_method = “LM”, correction = “BH”, standardize = FALSE, transform = “NONE”) on the complete community at once, and DESeq2 v1.40.2 [50] (independentFiltering = TRUE) on each species independently. Genes detected by at least one method (*P*_adj_ ≤ .2 or DESeq2 or qval ≤ 0.2 for MTXmodel) were considered differentially expressed. For metabolites: fold of intensities of meta-metabolomic features were calculated in order to highlight differences in amounts of metabolites between WN and SD conditions. Metabolic features, present in a condition and absent in the other or with fold among the five highest (whether identified or not) were considered. We also considered identified features with a fold change of at least 1.5 between incubation conditions.

## Results

The temporal dynamics of H_2_O_2_ and formaldehyde concentrations in the microcosms are shown in Supplementary Fig. S1. In the abiotic controls, these remained constant during the course of the experiments. In the biotic SD condition, the microbial assemblage significantly degraded H_2_O_2_ (as compared to the abiotic samples, Kruskal–Wallis, *P-*value < 0.05), as previously observed [13, 51]. The concentration dropped under 20% of its initial value after 2 and 3 h of incubation for the meta-metabolomic and metatranscriptomic samples, respectively (Supplementary Fig. S1B). Formaldehyde concentration decreased in both the WN and SD biotic samples (Kruskal–Wallis, *P-*value < 0.05 Supplementary Fig. S1C). The meta-metabolomics and metatranscriptomics analyses were phased according to these degradation kinetics, allowing the detection of 461 metabolites and 7191 transcripts, respectively at sampling times. Detected metabolites were representative of the global microbial assemblage pathway activities, unless uniquely produced by an individual member of the assemblage (see below). On the other hand, transcripts bearing the signature from individual genomes, were indicative of the contribution of each individual member to the global transcriptional activity of the microbial assemblage. Expressed transcripts were highlighted on global metabolic pathway maps (Supplementary Fig. S3).

**Figure 2:**
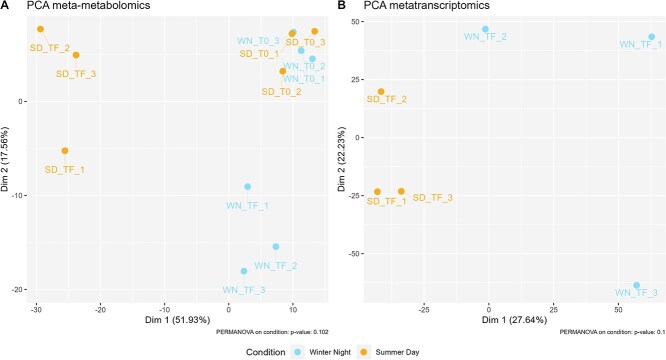
PCAs of synthetic microbial assemblages. (A) PCA score plot of metabolomics CLR normalized positive ionization mode data. (B) PCA score plot of metatranscriptomics CLR normalized count data. Samples are annotated by incubation condition, sampling time, (T_0 and T_F mean initial and final time, respectively) and replicate number.

Although most of the detected metabolites (95%) displayed no noticeable difference in their abundance, the metabolomic patterns between the SD and WN conditions were distinct from each other at the end of the incubation, and these also both differed from the initial patterns (Fig. 2A). These observations indicate distinct metabolic responses of the microbial assemblage to the two conditions investigated. A total of 25 metabolites, of which 10 could be identified, were detected as differentially abundant between conditions at the sampling time (Supplementary Table S2). DL-Met sulfoxide, 2-aminobenzoic acid, and six other unidentified compounds, were detected in only one of the conditions (below the detection limit in the other condition), which may suggest that they are not produced, or readily transformed into other product under one of the two tested conditions.

**Figure 3:**
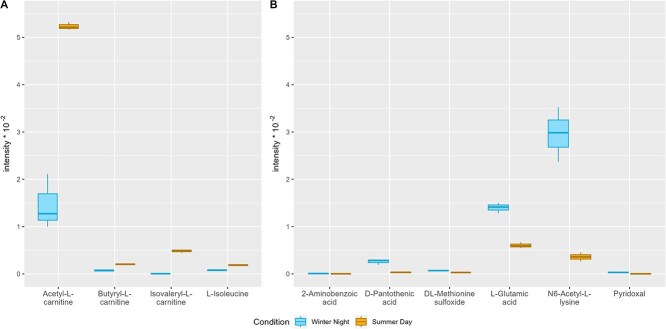
Boxplots comparing identified metabolites intensities in WN and SD conditions. (A) Identified metabolites more abundant in the SD T_F_ condition. (B) Identified metabolites more abundant in the WN T_F_ condition. Each boxplot was constructed using three biological replicates, the bold line corresponds to the median, the lower and upper hinges to the 25th and 75th percentiles, the whiskers extend to the smallest and largest values.

**Figure 4:**
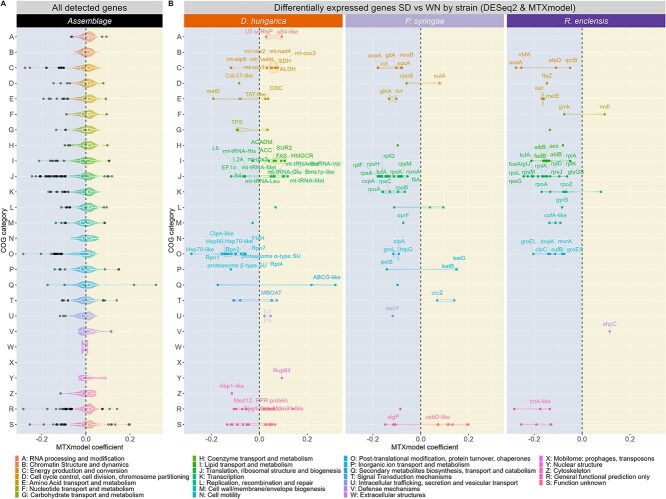
Expression coefficients of detected transcripts. (A) Expression coefficient distribution of all genes (data is grouped with all organisms together) with detectable expression in the complete assemblage according to functional category (clusters of orthologous genes—COG), dots correspond to outliers. (B) DEGs presented separately for each organism with each coloured dot corresponding to a DEG identified in either *D. hungarica, P. syringae,* or *R. enclensis* with DESeq2 or MTXmodel. For both panels, data are compared between cloud-like conditions found at WN (blue panel) and SD (yellow panel), genes are distributed on the vertical axis according to their classification into COGs functional categories from A to Z, the horizontal axis represents MTXmodel coefficients, with negative values corresponding to transcripts more abundant in the WN condition, positive values to transcripts more abundant in the SD condition.

The amino acid L-isoleucine and three types of acyl-carnitines [acetyl-L-carnitine (C2), butyryl-L-carnitine (C4), and isovaleryl-L-carnitine (C5)] were statistically more abundant in the SD than in the WN condition (Fig. 3A). Acyl-carnitines are signature metabolites of the yeast member of the microbial assemblage. In eukaryotes, they play a key role in the translocation of acetyl units between cellular compartments to support oxidative phosphorylation within the mitochondrion [52]. The need of active carnitine-mediated transport in the mitochondrion for lipid metabolism in the SD compared to the WN condition was also suggested by higher amount of yeast mitochondrial transcripts involved in the process by which fatty acids are broken down to produce energy (see transcriptomic data analysis, Fig. 4A).

Metabolites found more abundant in the WN condition included D-pantothenic acid (precursor of the synthesis of coenzyme A), vitamin B6-related pyridoxal (coenzyme of many enzymes, among which transaminases), and the amino acid L-glutamate, N6-acetyl-L-lysine, and DL-methionine sulfoxide (Fig. 3B). If oxidized methionine residues such as Met sulfoxide accumulate in proteins, they alter protein function. Moreover, higher transcript abundance of methionine sulfoxide reductase MsrA, which is a central component in the repair of oxidized methionine residues, was observed in WN than in SD in one of the bacterial members (Fig. 4B).

To summarize, among 25 differently abundant metabolites, 10 were identified, and 4 (acetyl-L-carnitine, butyryl-L-carnitine, isovaleryl-L-carnitine, and Met sulfoxide) have been linked with metatranscriptomics data, as detailed hereafter.

Regarding metatranscriptomics, 1 377 886 filtered non-rRNA paired-end reads were mapped against the 26 453 non-rRNA predicted genes of the combined microbial genomes of the assemblage, resulting in the detection of 7191 distinct expressed genes in total (Supplementary Fig. S2). Abiotic control-derived-metatranscriptome demonstrated minimal cross-contamination, pervasive or template-independent amplification (under 7.5% and more than 71.1% of bases of filtered non-rRNA paired-end reads mapped on the combined microbial genomes, for abiotic and biotic samples respectively).

All the members of the assemblage contributed to the total RNA pool, although at different proportions, with a predominance of *Dh* reads (93.36% of all reads), especially in SD, and only few affiliated with *Pg* (0.45% of all reads) (Supplementary Figs. S3A and S4).

The SD and WN samples formed two distinct groups in the PCA analysis of metatranscriptomics and metabolomics data (Fig. 2A and B). RNA expression was rather stable at the assemblage level between SD and WN for many functional categories (median expression coefficients of detectable transcripts close to zero), despite heterogeneity in expression patterns of genes within categories (violin shape) (Fig. 4A). Nonetheless, more enzyme-encoding genes were active in WN, with also more functional redundancy between member strains compared with the SD condition (Supplementary Fig. S3). To observe finer transcriptional responses of assemblage members, especially since yeast transcripts dominated the dataset, we then performed differential expression analyses, strain by strain.

Among all expressed genes, a total of 218 were differentially expressed (DEGs) between the WN and SD condition at the end of incubation (Fig. 4B, Supplementary Table S3). Nearly half of these DEGs (48%) was contributed by the yeast. Bacterial DEGs were evenly distributed between *Ps* and *Re* (27% and 25% of the DEGs, respectively), and no DEG was detected in *Pg* (owing to its low amounts of reads and biomass, and potentially indicating low activity level, see statistical analysis in Supplementary Material 1). About 69% of the fungal and 83% of the bacterial DEGs had a predicted function (excluding “Unknown function” and “General function” COG prediction categories) (Supplementary Table S3).

### Fungal mitochondrion energy production and associated acetyl-CoA metabolism occur in the SD condition

A total of 15 mitochondrial genome-encoded DEGs were significantly more expressed in the SD than in the WN condition (Fig. 4B, COG categories C and J). These included transcripts encoding components of the respiratory chain for energy production (Atp8, Cox1-3, Nad4-4L) and of the mitochondrial translation machinery (7 tRNAs; 1 ribosomal protein). Other SD-detected DEGs, nucleus-encoded, also contribute to mitochondrial functions: succinate dehydrogenase, a putative mitochondrial inner membrane protein (MDM31), and a pentatricopeptide repeat protein which could be a potential regulator of organelle posttranscriptional expression [53].

To fuel the mitochondrial tricarboxylic acid (TCA) cycle for ATP and C2-unit production, acetyl coenzyme A (acetyl-CoA) is a central metabolite [52]. In the SD condition, key genes encoding enzymes involved in acetyl-CoA metabolism displayed increased transcript abundance. These included acetyl-CoA carboxylase (ACC; EC. 6.4.1.2) that catalyzes the irreversible carboxylation of acetyl-CoA to produce the substrate for the biosynthesis of fatty acids, malonyl-CoA and one subunit of the fatty-acyl-CoA synthase system, the 3-oxoacyl-[acyl-carrier-protein] synthase (EC 2.3.1.86) that uses malonyl-CoA and acetyl-CoA to produce long-chain fatty acids (Supplementary Table S3). As acetyl-CoA is a compound unable to readily pass-through biological membranes, including those of organelles, acyl unit transport systems are needed for mitochondrial metabolic activity [52]. Several DEGs associated with lipid metabolism and transport (medium-chain acyl-CoA dehydrogenase ACADM, ACC, sphinganine C4-hydroxylase SUR2, and membrane-bound O-acyltransferase MBOAT) indicate that acyl transport was active in the SD condition (Fig. 4B). Overall, fungal DEG analysis suggests active energy production in the mitochondrion and acetyl-CoA-associated metabolism during the SD condition.

### Bacterial oxidative stress responses differ between the SD and WN conditions

Since H_2_O_2_ and light generate ROS harmful for cellular components [54], the DEGs encoding enzymes with oxidant detoxification functions were expected in the SD condition [55]. Indeed, higher abundance of catalase transcripts were detected in *Ps* (KatB [56]; KatG linked to a broad-spectrum peroxidase [57]), along with a gene overlapping *sodA* which encodes the Mn-containing superoxide dismutase. In *Re* transcripts more expressed in SD included that of hydroxyperoxidase AhpC (thioredoxin-based element of antioxidant defence) localized next to the H_2_O_2_-induced gene activator OxyR (Fig. 4B, Supplementary Table S3) [58], as well as *nrdI* transcripts. The NrdI flavodoxin is known to control the reduction of ribonuclease reductase class Ib activity and maintenance in *Escherichia coli* under severe oxidative stress [59]. This results in lower amounts of deoxynucleotide building blocks available for DNA synthesis [60], which may limit cell division under the SD condition. Altogether, these data suggest that the SD condition represented an extreme oxidative stress for the bacterial members of the assemblage.

On the other hand, WN-higher abundance DEGs included functions involved in the repair of covalent modifications of proteins harbouring the oxidative-sensitive sulphur atom, as found in the amino acid side chains of cysteine and methionine, and in iron–sulphur clusters [61]. These included in *Re*, the oxidized iron–sulphur cluster rescue SufB and the methionine sulfoxide reductase MsrA, which is a central component of the repair of oxidized methionine residue. Moreover, protein quality control in WN was suggested by DEGs related to major molecular chaperones (reviewed in [62]) with transcripts encoding DnaK and GroL/GroEL [63], and the ClpC desaggregase that cooperates with DnaK in efficient reactivation of severely aggregated proteins [64] (Fig. 4B, Supplementary Table S3). Proteostasis might also imply the collaboration of the heat shock proteins Hsp90, encoded by the gene *htpG* [65], a DEG overrepresented in WN, and Hsp70 encoded by the gene *dnaK*, chaperonin (GroEL), and the ATP-powered unfoldase ClpA with its partner ClpP peptidase, which all plays a role in damaged and misfolded protein remodelling [66].

Thus, the recruitment of different oxidative stress response processes occurs in bacteria exposed to SD and WN conditions, with prioritization towards oxidant detoxification during SD, when concentrations are highest, and protein function protection and repair mechanisms during the night.

### Yeast and bacteria favour biomass production and cell division in the WN condition, unlike in the SD condition

The WN condition seemed to favour bacterial cell division as deduced from significantly higher expression of key genes such as gene *ftsZ* (central to initiate cell-division) and *cpoB* (key for the coordinating of peptidoglycan synthesis and outer membrane constriction during cell division) [67] (Fig. 4B). In contrast, in the SD condition, decreased abundance of the transcript encoding FtsZ, along with the increased abundance of the transcript of SulA, a protein that halts bacterial cell division by binding to FtsZ, to avoid premature segregation of damaged DNA to daughter cells [68], suggested that bacteria were not dividing.

In the yeast member of the microbial assemblage, high transcript abundances during WN were found for a gene encoding Cdc37, which is a Hsp90 co-chaperone required to pass through the START phase of the yeast cell cycle into the DNA replication phase (Fig. 4B). This would be expected from cells getting ready to divide, suggesting, as for bacteria, on-going cell division processes.

Biomass production requires active protein synthesis. This probably occurred in the WN condition in the yeast member of the assemblage, with an ensemble of DEGs involved in translation processes and protein turnover. It included transcripts of the translation elongation factor EF1 alpha, several ribosomal proteins, factors of proteasome regulation, proteasome components, and a putative FtsH mitochondrial protease.

**Figure 5:**
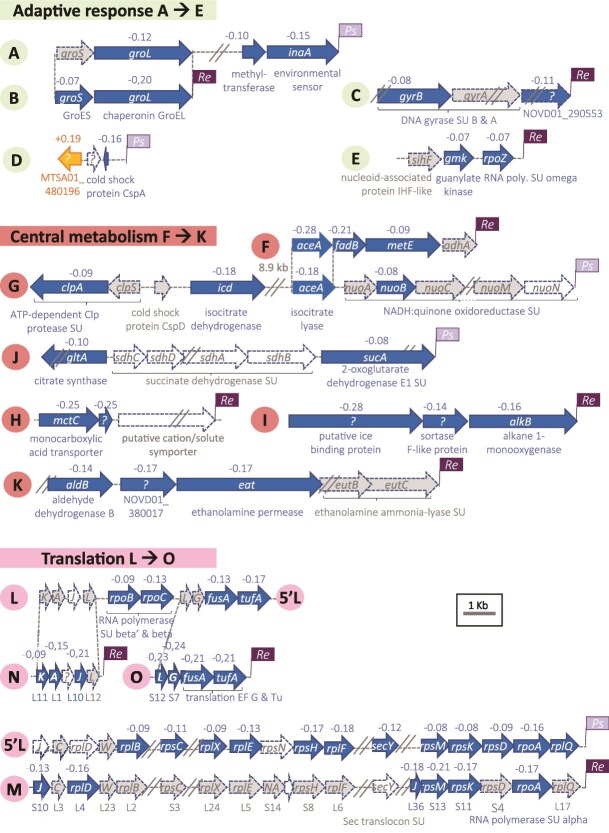
Co-localized bacterial DEGs. Fifteen gene clusters (named A to O) contain at least two DEGs with a predicted function related to stress response, metabolism or translation, among those, cluster A-B, F-G and L-O were retrieved in both *R. enclensis* (Genbank accession n° NOVD00000000, labelled *Re* in a flag), and *P. syringae* (Genbank accession n°HQ256872, labelled *Ps* in a flag), arrows indicate genes MTXmodel coefficient values are indicated above the gene name, with negative values corresponding to DEGs more expressed in the WN compared to the SD condition and positive values to DEGs more expressed in the SD compared to the WN condition, genes in light shading were not found as differently expressed, genes with no shading had no detected expression, the names of 50S and 30S ribosomal protein subunit-encoded genes start with L and S, respectively, in cluster F, gene *fabB* encodes 3-hydroxybutyrylCoA dehydrogenase and gene *metE* encodes 3-hydroxybutyrylCoA dehydrogenase.

**Figure 6:**
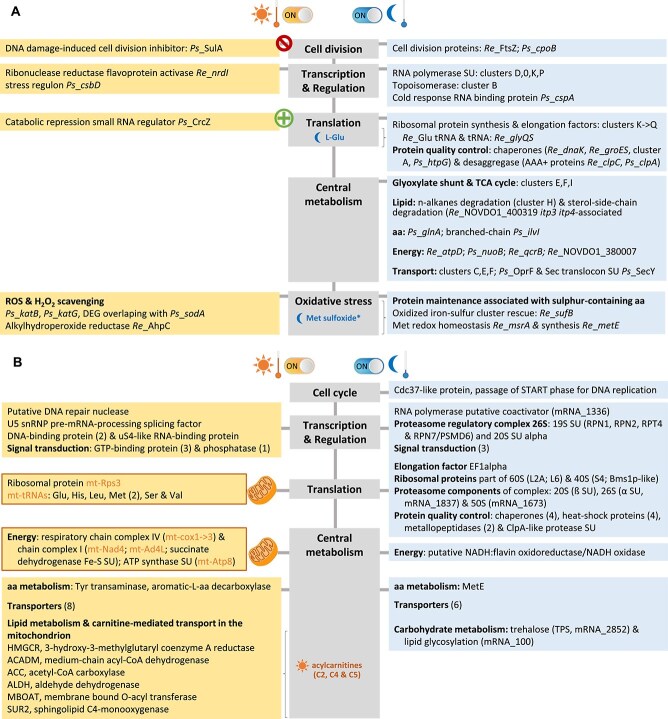
Overview of the microbial functions impacted during SD and WN cloud-like incubations. Relevant differently abundant metabolites indicated in the central grey rectangles are written in orange or blue colour when found as more abundant in cells incubated in the SD or in the WN conditions, respectively, metabolite names followed by asterisks were only detected in the WN condition, DEGs detected as more expressed in the SD (left rectangles) and WN (right rectangles) cloud-like conditions are indicated. (A) Fungal DEGs. "mt-" indicates mitochondrion-encoded genes, three types of acylcarnitines were detected as more abundant in the SD condition including acetyl-L-carnitine (C2), butyryl-L-carnitine (C4) and isovaleryl-L-carnitine (C5). (B) Bacterial DEGs. The origin is indicated as *Re* for *R enclensis* and *Ps* for *P syringae* (details in Supplementary Table S3), bacterial cell division is active in WN unlike the SD cloud-like condition, as shown with “prohibited direction sign”.

In bacteria, in the WN condition, protein expression was corroborated by several DEGs, arranged in clusters of genes coding for ribosomal protein components, translation elongation, translation factors, and RNA polymerase subunit alpha (Fig. 5). This suggests transcription and translation coupling in a context of active transcription processes as indicated by higher relative transcript abundance of genes encoding DNA-dependent RNA polymerase subunits alpha, beta, beta’, and omega (Fig. 4B). Other DEGs that were close in the genome were involved in central metabolism, with the first enzyme of the glyoxylate cycle that redirects isocitrate from the TCA cycle to the glyoxylate bypass (gene *aceA*, Fig. 6 clusters F-G) [69, 70] and TCA cycle enzymes (genes *gltA* and *sucA*, Fig. 6 cluster J). The proximity of these DEGs in the genome, with a total of 15 DEG clusters (Fig. 6), suggests a coordinated expression of genes required in translation processes and central metabolism. In summary, DEG patterns indicate that both prokaryotic and eukaryotic members of the microbial assemblage are likely thriving in the WN cloud-like condition.

## Discussion

The microbial assemblage degraded formaldehyde both in the WN and the SD conditions. The bacterial members of the assemblage were each previously shown to individually process atmospheric organic compounds including formaldehyde [4, 5, 8, 25] (J.M.V. and P.A., unpublished data). Here, transcripts of the bacterial glutathione-independent formaldehyde dehydrogenase (*fdhA* in *Re*) and yeast formaldehyde transketolase (mRNA_5434) were detected in both the WN and SD conditions at similar abundance.

### Bacterial and fungal cell maintain metabolic activity in the cold WN condition

The bacteria and yeast displayed overall similar functional responses under the WN condition, with high levels of transcripts oriented towards the production of biomass: TCA cycle, glyoxylate shunt, lipid degradation, amino acid metabolism, and energy and transport in bacteria (Fig. 6A), and protein biosynthesis, degradation and quality control, along with cell cycle progression into DNA replication in the yeast (Fig. 6B). These overall corroborate and specify previous observations in natural clouds [10].

Both bacteria and the yeast also expressed markers of stress response, with higher transcript abundance of genes encoding chaperones in the WN condition. In particular, several fungal Hsp70-like and a bacterial DnaK were detected. Throughout the tree of life, chaperones of the Hsp70 family are known to contribute to numerous stress responses [71]. A transcript encoding an RNA chaperone cold-shock protein CspA in *Ps* was also found more expressed in WN than SD (Fig. 4B). This protein destabilizes secondary structures of transcripts to ease translation at low temperatures [72]. Another acclimation to the low temperature condition of WN consisted in a more expressed putative bacterial ice-binding protein in *Re*, belonging to the widespread DUF3494 family (Fig. 4B, Supplementary Table S3, NOVD01_700017). This protein may bind ice crystals to inhibit their growth [73]. Altogether, it appears that both eukaryotic and prokaryotic strains can exploit diverse acclimation mechanisms and remain metabolically active in simulated low temperature cloud-water conditions, at low levels of oxidants.

### High oxidant levels in the SD condition have distinct impacts on bacteria and fungi

Within the assemblage exposed to SD, the fungal *Dh* DEG pattern suggests maintenance of the mitochondrial-driven ATP production (Fig. 6B). Functional mitochondria are essential in the fight against oxidants, as demonstrated in another basidiomycetous yeast (*Cryptococcus neoformans*) treated with exogenous H_2_O_2_ [74]. In our study, mitochondrial activity is in line with the increased abundance of DEGs encoding enzymes of the acyl-CoA metabolism and the detection at higher abundance of 3 acyl-L-carnitines metabolites. Indeed, these metabolites may be involved in the transport of fatty acids into the mitochondrion for energy production. In the SD condition, the levels of fungal translation-related DEG abundance decreased, as also observed in H_2_O_2_-exposed *Saccharomyces cerevisiae.* In this latter, translation postinitiation was rapidly inhibited after exposure to oxidants, leading to rapid but reversible dose-dependent accumulation of stress response mRNAs, allowing for swift protein synthesis once the stress is alleviated [75].

In contrast, the SD condition can be considered as an extreme oxidative stress for bacterial members of the assemblage, that in response entered a survival mode, with cell division arrest and activation of H_2_O_2_ scavenging processes (Fig. 6B). In bacteria, the presence of oxidants such as H_2_O_2_ is known to cause a slowdown of protein synthesis due to a general down-regulation of tRNAs [76], resulting in a temporary arrest of *E. coli* cell growth [77]. In addition, ROS can damage reactive thiol-containing amino acids, and for instance oxide methionine residues to methionine sulfoxide which may lead to protein inactivation. In this study, DL-methionine sulfoxide was detected exclusively in the WN condition, which correlated with DEGs encoding a MsrA methionine sulfoxide reductase in *Re* (Fig. 6A). One could expect that in such condition where cells are not metabolically active, the maintenance of housekeeping enzymes is (temporarily) dispensable, as found in non-dividing bacteria under the SD condition. This suggests that the high intensity oxidative stress generated by the combination of H_2_O_2_ and light under the SD condition represents a major and stringent stress for bacteria that need to be addressed before anabolic activity. In contrast, oxidative damage protein rescue and response to the SD oxidative stress was not so detrimental to the maintenance of other primary functions than stress responses in the yeast. Our results support the fact that oxidants are one of the main drivers of bacteria metabolism in clouds, as suggested by earlier observations [10, 12, 13, 45], with impacts on cloud chemistry [15, 17]. The larger biovolume represented by yeast cells, in comparison to bacteria, along with their specific organelles dedicated to oxidant detoxification (peroxisomes), may be involved in the yeast capacity to tolerate higher concentrations of oxidants.

### Cloud droplet microbial ecology, a dynamic interplay between multi-kingdom microbial metabolism and cloud chemistry

Clouds consist of multitudinous water droplets of typically ~5 to 50 μm in diameter, where microbial cells occur at low frequency with statistically ~1 out of >1000 cloud droplets that includes a bacterial or a yeast cell [2]. Therefore, the possibility that two or more cells of distinct affiliations are hosted together in the same droplet is low, although the dynamic and massive number of droplets forming clouds (~10^7^/m^3^ or more) may favour transient microbial cell co-occurrence within a same droplet. When this occurs, synergic (e.g. detoxification) or antagonist interactions (e.g. competition for resources) may rapidly arise given the micro-volume size of a cloud droplet. In this study, we found DEGs involved in functions that favour rapid cell interactions such as cell motility associated with flagella driven chemotaxis (Fig. 4B, in *Ps, fliA* encodes the RNA polymerase sigma factor for flagella operon expression [78]), and volatile cell–cell communication signals (Fig. 4B, in *Re*, *vbfA* [synonymous to *ywcA*] encodes a monocarboxylic acid transporter possibly involved in volatile signal for biofilm formation [79]). Other microbial functions that may potentially help microbial dissemination between cloud droplets (e.g. biosurfactant production in *Ps* [18] and *Dh* ballistospore production [21]) were not identified in our study. Thus, airborne microorganisms may take strategies to encounter, evade, interact, cooperate or compete directly with other cells in responses to stresses encountered in transiently shared cloud droplets.

On the other hand, microbial cells, even located in different cloud droplets, collectively impact the availability of carbon sources such as volatile compounds. It has been previously shown that condensed water in clouds is associated with increased metabolic activity in airborne cells as compared to clear atmosphere, with potentially numerous impacts on the atmospheric chemical reactivity and on the ecology of these microorganisms [80]. To consider the different levels of complexity, the impacts of biodegradation processes on cloud chemical reactivity were previously estimated by models [8, 15–17]. These demonstrated that the most significant impacts of biological activity are to be expected towards small, moderately soluble, and semi-volatile compounds, such as formaldehyde, formate, and acetate, which can be efficiently transferred between the gas and the liquid phases and so can equilibrate between droplets [15]. In the case of depletion such as caused by microbial utilization, the aqueous phase therefore rapidly replenishes with these compounds, which are therefore not expected to represent limitations for cloud microorganisms. Up to 20 ppt.h^−1^ of formate are estimated to be processed by bacteria-driven processes in clouds under optimal pH conditions [17].

Due to temperature limitations, the highest impacts of microbial metabolism on chemistry are expected during summer, and in particular during the night when oxidants are at their lowest concentration due to the slow-down of photocatalytic processes [8, 16]. The concentration of oxidants in natural clouds follows a diurnal cycle. H_2_O_2_ concentration usually ranges from <1 to ~20 μM, with higher concentrations during the day [28]. Under exposure to light, H_2_O_2_ decays with a half-life time of ~2 to 5 h [28], generating hydroxyl radicals at a rate of ~0.2 to 4 × 10^−10^ M·s^−1^ depending on the chemical composition (H_2_O_2_, nitrate and iron contents) [81, 82].

In our study, we observed multiple oxidative stress responses in the microbial assemblage during the SD condition, under light exposure at high oxidant concentration, and rather repair mechanisms and potentially growth during the WN condition, in the dark and with no added oxidant. The cell division arrest observed during the SD condition may represent a transient stage in which cells can cope with the day-conditions typical of the cloud environment, waiting for the alleviation of oxidative stress at night, in which cell would demonstrate resilience by their capacity to reactivate their metabolism, as previously suggested [12]. This raises the question of how the cloud microbial community metabolism changes at sunset to cope with resident oxidants generated during the day before being able to recover for cell division at night. The temporal scale and kinetic at which metabolic switches and recovery occur (resilience, survival) after high oxidant exposure remains to be assessed in laboratory microcosm-driven time series experiments, using microbial isolates, assemblages, and ultimately natural cloud water samples, and atmospheric simulation chambers.

Here, we observed that the microbial response to the SD stresses differs between bacteria and yeast. Indeed, the yeast was metabolically active in both SD and WN conditions, which illustrates that the cellular responses to environmental conditions, such as oxidants, are largely kingdom-dependent. This suggests that the yeast might conduct most of the formaldehyde biodegradation observed in SD. As such, biodegradation processes could rely on prokaryotic or eukaryotic actors depending on conditions and the ability of strains to face high oxidant concentrations. Altogether, the multi-kingdom microbial assemblages in clouds likely exhibit complex biological responses to environmental conditions, in accordance with their resilience and metabolic capabilities.

As suggested by our study, estimating the biological contribution to cloud chemistry might be more complex than currently conceptualized so far from prokaryote-focused studies [23], which may need revisions. Deeper behaviour investigation of yeasts in cloud conditions will help to estimate their potential impact on atmospheric chemical processes, including the fate of oxidants and dissolved carbon. Investigations on inter-species short-term interactions in the cloud environment could also be an avenue to better understand cloud microbial assemblage dynamics and their role in the global ecosystem.

## Supplementary Material

Jarrige_et_al_MS_Supplementary_Material_ycaf200

Table_S3_DEGs_annotations_ycaf200

Supplementary_material_1_ycaf200

## Data Availability

Metatranscriptomic datasets are available under the accession number: PRJNA986488. The metabolomic data have been deposited to MetaboLights repository with the study identifier MTBLS12281. Bioinformatics scripts and statistical analysis reports are available at the following Zenodo accession: https://zenodo.org/records/16754027.

## References

[1] Amato P, Joly M, Besaury L. et al. Active microorganisms thrive among extremely diverse communities in cloud water. *PLoS One* 2017;12:e0182869. 10.1371/journal.pone.0182869 28792539 PMC5549752

[2] Ervens B, Amato P, Aregahegn K. et al. Ideas and perspectives: microorganisms in the air through the lenses of atmospheric chemistry and microphysics. *Biogeosciences* 2025;22:243–56. 10.5194/bg-22-243-2025

[3] Vaïtilingom M, Attard E, Gaiani N. et al. Long-term features of cloud microbiology at the puy de Dôme (France). *Atmos Environ* 2012;56:88–100. 10.1016/j.atmosenv.2012.03.072

[4] Amato P, Demeer F, Melaouhi A. et al. A fate for organic acids, formaldehyde and methanol in cloud water: their biotransformation by micro-organisms. *Atmos Chem Phys* 2007;7:4159–69. 10.5194/acp-7-4159-2007

[5] Husárová S, Vaïtilingom M, Deguillaume L. et al. Biotransformation of methanol and formaldehyde by bacteria isolated from clouds. Comparison with radical chemistry. *Atmos Environ* 2011;45:6093–102. 10.1016/j.atmosenv.2011.06.035

[6] Liu Y, Lim CK, Shen Z. et al. Effects of pH and light exposure on the survival of bacteria and their ability to biodegrade organic compounds in clouds: implications for microbial activity in acidic cloud water. *Atmos Chem Phys* 2023;23:1731–47. 10.5194/acp-23-1731-2023

[7] Sattler B, Puxbaum H, Psenner R. Bacterial growth in supercooled cloud droplets. *Geophys Res Lett* 2001;28:239–42. 10.1029/2000GL011684

[8] Vaïtilingom M, Charbouillot T, Deguillaume L. et al. Atmospheric chemistry of carboxylic acids: microbial implication versus photochemistry. *Atmos Chem Phys* 2011;11:8721–33. 10.5194/acp-11-8721-2011

[9] Péguilhan R, Rossi F, Joly M. et al. Clouds influence the functioning of airborne microorganisms. *Biogeosciences* 2025;22:1257–75. 10.5194/bg-22-1257-2025

[10] Amato P, Besaury L, Joly M. et al. Metatranscriptomic exploration of microbial functioning in clouds. *Sci Rep* 2019;9:4383. 10.1038/s41598-019-41032-4 30867542 PMC6416334

[11] Jousse C, Dalle C, Canet I. et al. Metabolomic study of the response to cold shock in a strain of *Pseudomonas syringae* isolated from cloud water. *Metabolomics* 2018;14:11. 10.1007/s11306-017-1295-7 30830325

[12] Wirgot N, Lagrée M, Traïkia M. et al. Metabolic modulations of *Pseudomonas graminis* in response to H_2_O_2_ in cloud water. *Sci Rep* 2019;9:12799. 10.1038/s41598-019-49319-2 31488860 PMC6728378

[13] Vaïtilingom M, Deguillaume L, Vinatier V. et al. Potential impact of microbial activity on the oxidant capacity and organic carbon budget in clouds. *Proc Natl Acad Sci* 2013;110:559–64. 10.1073/pnas.1205743110 23263871 PMC3545818

[14] Ervens B, Amato P. The global impact of bacterial processes on carbon mass. *Atmos Chem Phys* 2020;20:1777–94. 10.5194/acp-20-1777-2020

[15] Khaled A, Zhang M, Amato P. et al. Biodegradation by bacteria in clouds: an underestimated sink for some organics in the atmospheric multiphase system. *Atmos Chem Phys* 2021;21:3123–41. 10.5194/acp-21-3123-2021

[16] Pailler L, Wirgot N, Joly M. et al. Assessing the efficiency of water-soluble organic compound biodegradation in clouds under various environmental conditions. *Environ Sci Atmos* 2023;3:731–48. 10.1039/D2EA00153E

[17] Nuñez López L, Amato P, Ervens B. Bacteria in clouds biodegrade atmospheric formic and acetic acids. *Atmos Chem Phys* 2024;24:5181–98. 10.5194/acp-24-5181-2024

[18] Besaury L, Amato P, Sancelme M. et al. Draft genome sequence of *Pseudomonas syringae* PDD-32b-74, a model strain for ice-nucleation studies in the atmosphere. *Genome Announc* 2017;5:e00742–17. 10.1128/genomeA.00742-17 28751406 PMC5532844

[19] Besaury L, Amato P, Wirgot N. et al. Draft genome sequence of *Pseudomonas graminis* PDD-13b-3, a model strain isolated from cloud water. *Genome Announc* 2017;5:e00464–17. 10.1128/genomeA.00464-17 28663290 PMC5638274

[20] Lallement A, Besaury L, Eyheraguibel B. et al. Draft genome sequence of *Rhodococcus enclensis* 23b-28, a model strain isolated from cloud water. *Genome Announc* 2017;5:e01199–17. 10.1128/genomeA.01199-17 29074669 PMC5658507

[21] Jarrige D, Haridas S, Bleykasten-Grosshans C. et al. High-quality genome of the basidiomycete yeast *Dioszegia hungarica* PDD-24b-2 isolated from cloud water. *G3 (Bethesda)* 2022;12:jkac282. 10.1093/g3journal/jkac282 36259934 PMC9713403

[22] Jaber S, Joly M, Brissy M. et al. Biotic and abiotic transformation of amino acids in cloud water: experimental studies and atmospheric implications. *Biogeosciences* 2021;18:1067–80. 10.5194/bg-18-1067-2021

[23] Jaber S, Lallement A, Sancelme M. et al. Biodegradation of phenol and catechol in cloud water: comparison to chemical oxidation in the atmospheric multiphase system. *Atmos Chem Phys* 2020;20:4987–97. 10.5194/acp-20-4987-2020

[24] Lallement A, Besaury L, Tixier E. et al. Potential for phenol biodegradation in cloud waters. *Biogeosciences* 2018;15:5733–44. 10.5194/bg-15-5733-2018

[25] Vaïtilingom M, Amato P, Sancelme M. et al. Contribution of microbial activity to carbon chemistry in clouds. *Appl Environ Microbiol* 2010;76:23–9. 10.1128/AEM.01127-09 19854931 PMC2798665

[26] Lallement A, Vinatier V, Brigante M. et al. First evaluation of the effect of microorganisms on steady state hydroxyl radical concentrations in atmospheric waters. *Chemosphere* 2018;212:715–22. 10.1016/j.chemosphere.2018.08.128 30179836

[27] Joly M, Amato P, Sancelme M. et al. Survival of microbial isolates from clouds toward simulated atmospheric stress factors. *Atmos Environ* 2015;117:92–8. 10.1016/j.atmosenv.2015.07.009

[28] Marinoni A, Parazols M, Brigante M. et al. Hydrogen peroxide in natural cloud water: sources and photoreactivity. *Atmos Res* 2011;101:256–63. 10.1016/j.atmosres.2011.02.013

[29] Rossi F, Péguilhan R, Turgeon N. et al. Quantification of antibiotic resistance genes (ARGs) in clouds at a mountain site (puy de Dôme, Central France). *Sci Total Environ* 2023;865:161264. 10.1016/j.scitotenv.2022.161264 36587700

[30] Sumner LW, Amberg A, Barrett D. et al. Proposed minimum reporting standards for chemical analysis: chemical analysis working group (CAWG) metabolomics standards initiative (MSI). *Metabolomics* 2007;3:211–21. 10.1007/s11306-007-0082-2 24039616 PMC3772505

[31] Giacomoni F, Le Corguillé G, Monsoor M. et al. Workflow4Metabolomics: a collaborative research infrastructure for computational metabolomics. *Bioinformatics* 2015;31:1493–5. 10.1093/bioinformatics/btu813 25527831 PMC4410648

[32] Smith CA, Want EJ, O’Maille G. et al. XCMS: processing mass spectrometry data for metabolite profiling using nonlinear peak alignment, matching, and identification. *Anal Chem* 2006;78:779–87. 10.1021/ac051437y 16448051

[33] Van Der Kloet FM, Bobeldijk I, Verheij ER. et al. Analytical error reduction using single point calibration for accurate and precise metabolomic phenotyping. *J Proteome Res* 2009;8:5132–41. 10.1021/pr900499r 19754161

[34] Mölder F, Jablonski KP, Letcher B. et al. Sustainable data analysis with Snakemake. *F1000Research* 2021;10:33. 10.12688/f1000research.29032.2 34035898 PMC8114187

[35] Ewels P, Magnusson M, Lundin S. et al. Multi QC: summarize analysis results for multiple tools and samples in a single report. *Bioinformatics* 2016;32:3047–8. 10.1093/bioinformatics/btw354 27312411 PMC5039924

[36] Chen S, Zhou Y, Chen Y. et al. Fastp: an ultra-fast all-in-one FASTQ preprocessor. *Bioinformatics* 2018;34:i884–90. 10.1093/bioinformatics/bty560 30423086 PMC6129281

[37] Kopylova E, Noé L, Touzet H. SortMeRNA: fast and accurate filtering of ribosomal RNAs in metatranscriptomic data. *Bioinformatics* 2012;28:3211–7. 10.1093/bioinformatics/bts611 23071270

[38] Dobin A, Davis CA, Schlesinger F. et al. STAR: ultrafast universal RNA-seq aligner. *Bioinformatics* 2013;29:15–21. 10.1093/bioinformatics/bts635 23104886 PMC3530905

[39] Liao Y, Smyth GK, Shi W. featureCounts: an efficient general purpose program for assigning sequence reads to genomic features. *Bioinformatics* 2014;30:923–30. 10.1093/bioinformatics/btt656 24227677

[40] Liao Y, Smyth GK, Shi W. The R package Rsubread is easier, faster, cheaper and better for alignment and quantification of RNA sequencing reads. *Nucleic Acids Res* 2019;47:e47–7. 10.1093/nar/gkz114 30783653 PMC6486549

[41] Marchler-Bauer A, Bo Y, Han L. et al. CDD/SPARCLE: functional classification of proteins via subfamily domain architectures. *Nucleic Acids Res* 2017;45:D200–3. 10.1093/nar/gkw1129 27899674 PMC5210587

[42] Johnson M, Zaretskaya I, Raytselis Y. et al. NCBI BLAST: a better web interface. *Nucleic Acids Res* 2008;36:W5–9. 10.1093/nar/gkn201 18440982 PMC2447716

[43] Grigoriev IV, Nikitin R, Haridas S. et al. MycoCosm portal: gearing up for 1000 fungal genomes. *Nucleic Acids Res* 2014;42:D699–704. 10.1093/nar/gkt1183 24297253 PMC3965089

[44] Vallenet D, Calteau A, Dubois M. et al. MicroScope: an integrated platform for the annotation and exploration of microbial gene functions through genomic, pangenomic and metabolic comparative analysis. *Nucleic Acids Res* 2019;48:D579–89. 10.1093/nar/gkz926 PMC7145621 31647104

[45] R Core Team . R: A Language and Environment for Statistical Computing. Vienna: R Foundation for Statistical Computing, 2023, 2023.

[46] Posit team . RStudio: Integrated Development Environment for R, Vol. 2023. Boston, USA: Posit Software, PBC, 2023.

[47] Wickham H . ggplot2: Elegant Graphics for Data Analysis. New York: Springer-Verlag, 2016, 10.1007/978-3-319-24277-4.

[48] Aitchison J, Barcelo-Vidal C, Martın-Fernandez JA. et al. Logratio analysis and compositional distance. *Math Geol* 2000;32:271–5. 10.1023/A:1007529726302

[49] Zhang Y, Thompson KN, Huttenhower C. et al. Statistical approaches for differential expression analysis in metatranscriptomics. *Bioinformatics* 2021;37:i34–41. 10.1093/bioinformatics/btab327 34252963 PMC8275336

[50] Love MI, Huber W, Anders S. Moderated estimation of fold change and dispersion for RNA-seq data with DESeq2. *Genome Biol* 2014;15:550. 10.1186/s13059-014-0550-8 25516281 PMC4302049

[51] Wirgot N, Vinatier V, Deguillaume L. et al. H2O2 modulates the energetic metabolism of the cloud microbiome. *Atmos Chem Phys* 2017;17:14841–51. 10.5194/acp-17-14841-2017

[52] Strijbis K, Distel B. Intracellular acetyl unit transport in fungal carbon metabolism. *Eukaryot Cell* 2010;9:1809–15. 10.1128/EC.00172-10 20889721 PMC3008284

[53] Herbert CJ, Golik P, Bonnefoy N. Yeast PPR proteins, watchdogs of mitochondrial gene expression. *RNA Biol* 2013;10:1477–94. 10.4161/rna.25392 24184848 PMC3858431

[54] Imlay JA . The molecular mechanisms and physiological consequences of oxidative stress: lessons from a model bacterium. *Nat Rev Microbiol* 2013;11:443–54. 10.1038/nrmicro3032 23712352 PMC4018742

[55] Mishra S, Imlay J. Why do bacteria use so many enzymes to scavenge hydrogen peroxide? *Arch Biochem Biophys* 2012;525:145–60. 10.1016/j.abb.2012.04.014 22609271 PMC3413786

[56] Klotz MG, Kim YC, Anderson AJ. Cloning, characterization and phenotypic expression in *Escherichia coli* of *catF*, which encodes the catalytic subunit of catalase isozyme CatF of *Pseudomonas syringae*. *Appl Microbiol Biotechnol* 1995;43:656–66. 10.1007/BF00164770 7546603

[57] Singh R, Wiseman B, Deemagarn T. et al. Catalase-peroxidases (KatG) exhibit NADH oxidase activity. *J Biol Chem* 2004;279:43098–106. 10.1074/jbc.M406374200 15280362

[58] Hillas PJ, Del Alba FS, Oyarzabal J. et al. The AhpC and AhpD antioxidant defense system of *Mycobacterium tuberculosis*. *J Biol Chem* 2000;275:18801–9. 10.1074/jbc.M001001200 10766746

[59] Cotruvo JA, Stubbe J. NrdI, a flavodoxin involved in maintenance of the diferric-tyrosyl radical cofactor in *Escherichia coli* class Ib ribonucleotide reductase. *Proc Natl Acad Sci* 2008;105:14383–8. 10.1073/pnas.0807348105 18799738 PMC2567162

[60] Monje-Casas F, Jurado J, Prieto-Álamo M-J. et al. Expression analysis of the nrdHIEF operon from *Escherichia coli*. *J Biol Chem* 2001;276:18031–7. 10.1074/jbc.M011728200 11278973

[61] da Cruz Nizer WS, Inkovskiy V, Versey Z. et al. Oxidative stress response in *Pseudomonas aeruginosa*. *Pathogens* 2021;10:1187. 10.3390/pathogens10091187 34578219 PMC8466533

[62] Wickramaratne AC, Wickner S, Kravats AN. Hsp90, a team player in protein quality control and the stress response in bacteria. *Microbiol Mol Biol Rev* 2024;88:e0017622–2. 10.1128/mmbr.00176-22 38534118 PMC11332350

[63] Aussel L, Ezraty B. Methionine redox homeostasis in protein quality control. *Front Mol Biosci* 2021;8:665492. 10.3389/fmolb.2021.665492 33928125 PMC8076862

[64] Kedzierska S, Akoev V, Barnett ME. et al. Structure and function of the middle domain of ClpB from *Escherichia coli*. *Biochemistry* 2003;42:14242–8. 10.1021/bi035573d 14640692 PMC1821349

[65] Nemoto TK, Ono T, Kobayakawa T. et al. Domain–domain interactions of HtpG, an *Escherichia coli* homologue of eukaryotic HSP90 molecular chaperone. *Eur J Biochem* 2001;268:5258–69. 10.1046/j.0014-2956.2001.02457.x 11606187

[66] Kotamarthi HC, Sauer RT, Baker TA. The non-dominant AAA+ ring in the ClpAP protease functions as an anti-stalling motor to accelerate protein unfolding and translocation. *Cell Rep* 2020;30:2644–2654.e3. 10.1016/j.celrep.2020.01.110 32101742 PMC7888974

[67] Gray AN, Egan AJ, vant Veer IL. et al. Coordination of peptidoglycan synthesis and outer membrane constriction during *Escherichia coli* cell division. *eLife* 2015;4:e07118. 10.7554/eLife.07118 25951518 PMC4458516

[68] Cordell SC, Robinson EJH, Löwe J. Crystal structure of the SOS cell division inhibitor SulA and in complex with FtsZ. *Proc Natl Acad Sci* 2003;100:7889–94. 10.1073/pnas.1330742100 12808143 PMC164683

[69] Nimmo H, Borthwick A, Elmansi E. et al. Regulation of the enzymes at the branchpoint between the citric acid cycle and the glyoxylate bypass in *Escherichia coli*. *Biochem Soc Symp* 1987;54:93–101. 3333001

[70] Ha S, Shin B, Park W. Lack of glyoxylate shunt dysregulates iron homeostasis in *Pseudomonas aeruginosa*. *Microbiology* 2018;164:587–99. 10.1099/mic.0.000623 29465342

[71] Yu A, Li P, Tang T. et al. Roles of Hsp70s in stress responses of microorganisms, plants, and animals. *Biomed Res Int* 2015;2015:1–8. 10.1155/2015/510319 PMC4663327 26649306

[72] Jiang W, Hou Y, Inouye M. CspA, the major cold-shock protein of *Escherichia coli*, is an RNA chaperone. *J Biol Chem* 1997;272:196–202. 10.1074/jbc.272.1.196 8995247

[73] Vance TDR, Bayer-Giraldi M, Davies PL. et al. Ice-binding proteins and the ‘domain of unknown function’ 3494 family. *FEBS J* 2019;286:855–73. 10.1111/febs.14764 30680879

[74] Upadhya R, Campbell LT, Donlin MJ. et al. Global transcriptome profile of *Cryptococcus neoformans* during exposure to hydrogen peroxide induced oxidative stress. *PLoS One* 2013;8:e55110. 10.1371/journal.pone.0055110 23383070 PMC3557267

[75] Shenton D, Smirnova JB, Selley JN. et al. Global translational responses to oxidative stress impact upon multiple levels of protein synthesis. *J Biol Chem* 2006;281:29011–21. 10.1074/jbc.M601545200 16849329

[76] Zhong J, Xiao C, Gu W. et al. Transfer RNAs mediate the rapid adaptation of *Escherichia coli* to oxidative stress. *PLoS Genet* 2015;11:e1005302. 10.1371/journal.pgen.1005302 26090660 PMC4474833

[77] Zhu M, Dai X. Maintenance of translational elongation rate underlies the survival of *Escherichia coli* during oxidative stress. *Nucleic Acids Res* 2019;47:7592–604. 10.1093/nar/gkz467 31131413 PMC6698664

[78] Ditty JL, Grimm AC, Harwood CS. Identification of a chemotaxis gene region from *Pseudomonas putida*. *FEMS Microbiol Lett* 1998;159:267–73. 10.1111/j.1574-6968.1998.tb12871.x 9503621

[79] Chen Y, Gozzi K, Yan F. et al. Acetic acid acts as a volatile signal to stimulate bacterial biofilm formation. *mBio* 2015;6:e00392–15. 10.1128/mBio.00392-15 26060272 PMC4462622

[80] Péguilhan R, Rossi F, Joly M. et al. Clouds influence the functioning of airborne microorganisms. Biogeosciences 2025;22:1257–1275. 10.5194/bg-22-1257-2025

[81] Bianco A, Passananti M, Perroux H. et al. A better understanding of hydroxyl radical photochemical sources in cloud waters collected at the puy de Dôme station – experimental versus modelled formation rates. *Atmos Chem Phys* 2015;15:9191–202. 10.5194/acp-15-9191-2015

[82] Bianco A, Passananti M, Brigante M. et al. Photochemistry of the cloud aqueous phase: a review. *Molecules* 2020;25:423. 10.3390/molecules25020423 31968643 PMC7024559

