# Supplementary material for: Multi-kingdom microbial assemblage modulates its metabolism under contrasted cloud conditions
Source: ISME Commun. 2025 Nov 3;5(1):ycaf200. doi: 10.1093/ismeco/ycaf200 (PMC12721874; doi:10.1093/ismeco/ycaf200)
Supplement: Jarrige_et_al_MS_Supplementary_Material_ycaf200 [file jarrige_et_al_ms_supplementary_material_ycaf200.pdf]

Supplementary material for: Multi-kingdom microbial assemblage modulates its metabolism under contrasted cloud conditions

Supplementary Figures

Figure S1 Sample characteristics of the combined metabolomic and transcriptomic approaches. **A.** Temporal relative H_2O_2 concentrations in the SD-incubated samples. **B.** Temporal relative formaldehyde concentration in the SD and WN cloud-like samples. Relative concentration of H_2O_2 and formaldehyde (CH_2O) were calculated as ratio of the initial quantities measured at time 0. Translucent coloured zones denote standard errors based on the standard deviation calculated on triplicate biological replicates. Asterisks denote significant differences (Kruskal-Wallis test, *p*value < 0.05) between biotic and abiotic samples at each time point. When the remaining H_2O_2 concentration reached under 20% of the initial concentration in the SD samples (horizontal red dashed line), the samples were harvested (black vertical lines). Thus, incubations for meta-metabolomic and metatrancriptomic samples were of 3h30min and 4h, respectively.

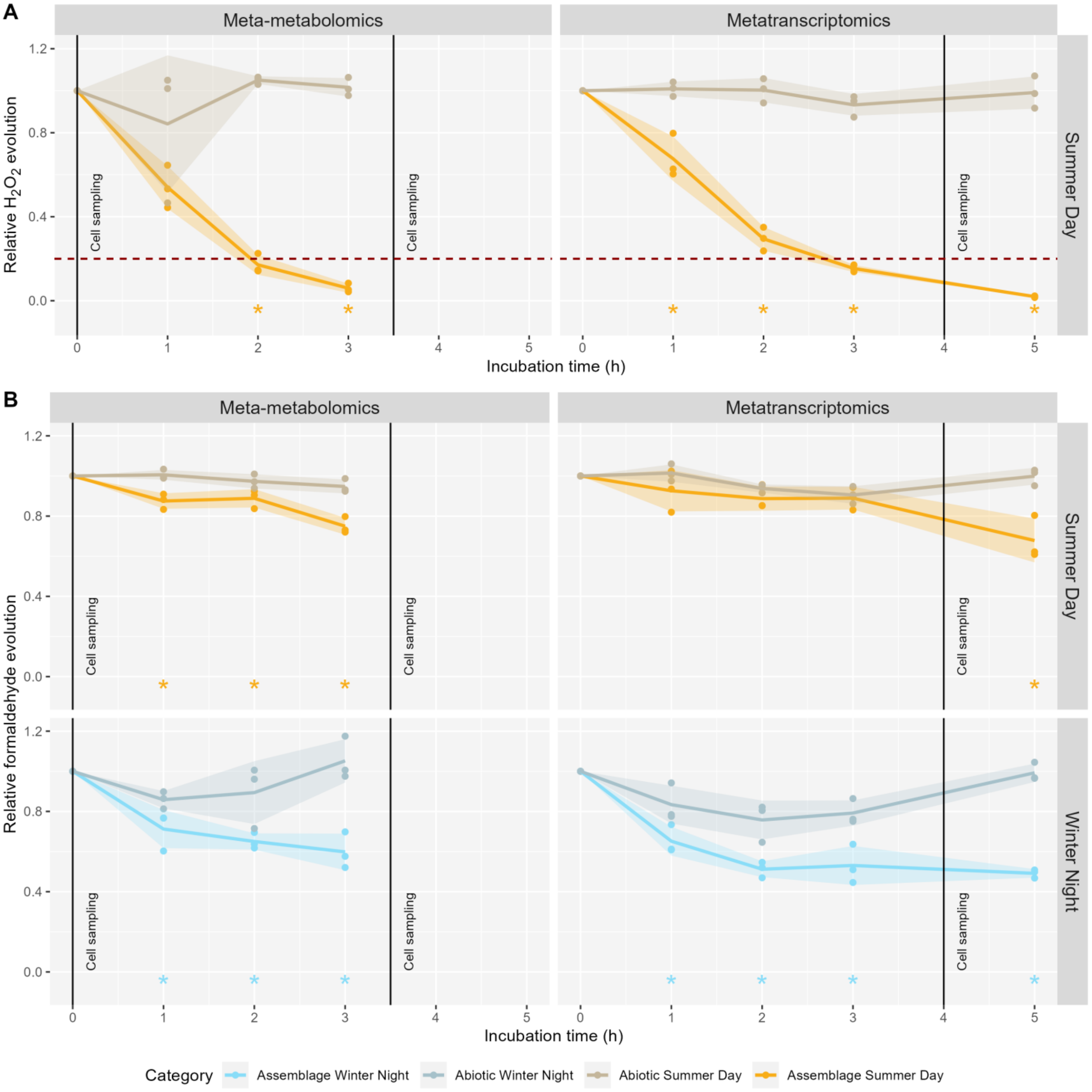

Figure S2 Metatranscriptomic analysis workflow and summary of mapped reads per sample and conditions

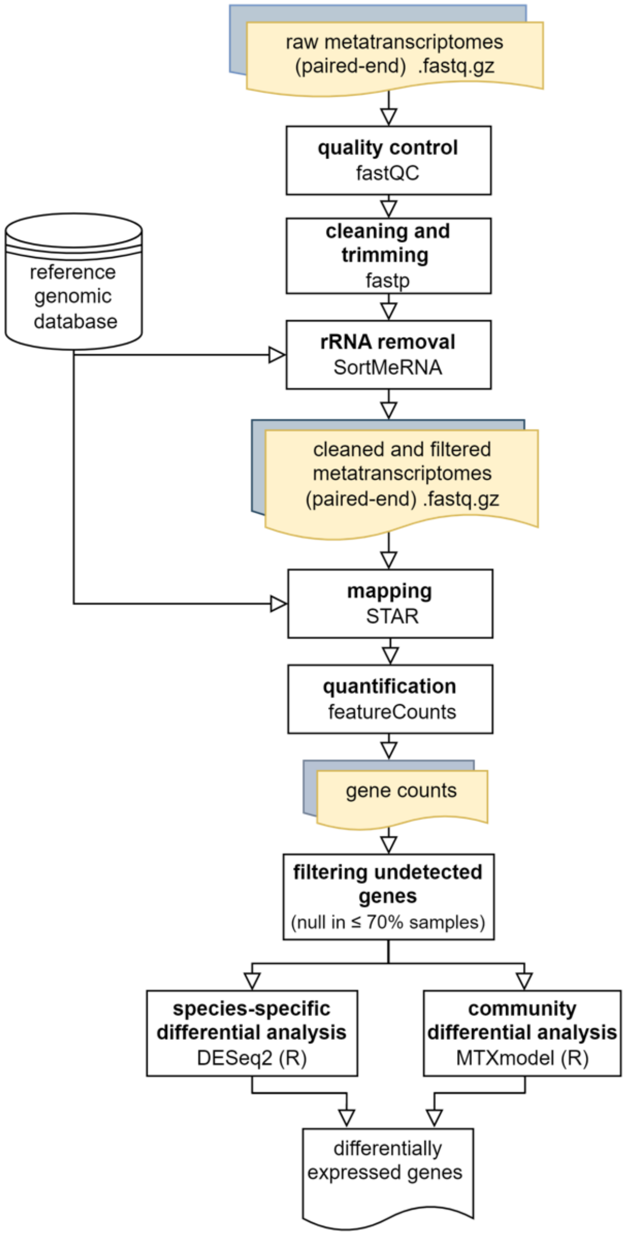

Total raw read pairs	**33 736 154**
Total cleaned read pairs	**18 025 718**
Total non-rRNA read pairs	**1 377 886**

Total non-rRNA mapped read pairs (biological samples)	**721 062**
Mean % mapped non-rRNA reads (biological samples)	**93.32 %**

Total non-rRNA genes	**26 487**
Expressed genes detected	**7 191**
Differentially expressed genes	**218**

Figure S3 Assemblage combined metabolic maps in SD and WN conditions. Global overview of the expressed metabolic maps detected in metatranscriptomics (DEGs and constitutive expression) and metabolomics (differentially abundant metabolites) data for the SD (panel A) and WN (panel B) conditions. Thick lines indicate pathways that are expressed with a colour reference for each of the member of the microbial inter-kingdom assemblage. Diamonds indicate differentially abundant metabolites detected by metabolomics. For detailed information, expressed pathways annotations can be recovered in the Zenodo repository: https://zenodo.org/records/16754027 and inputted in the interactive KEGG map "color" section at: https://www.kegg.jp/pathway/map01100.

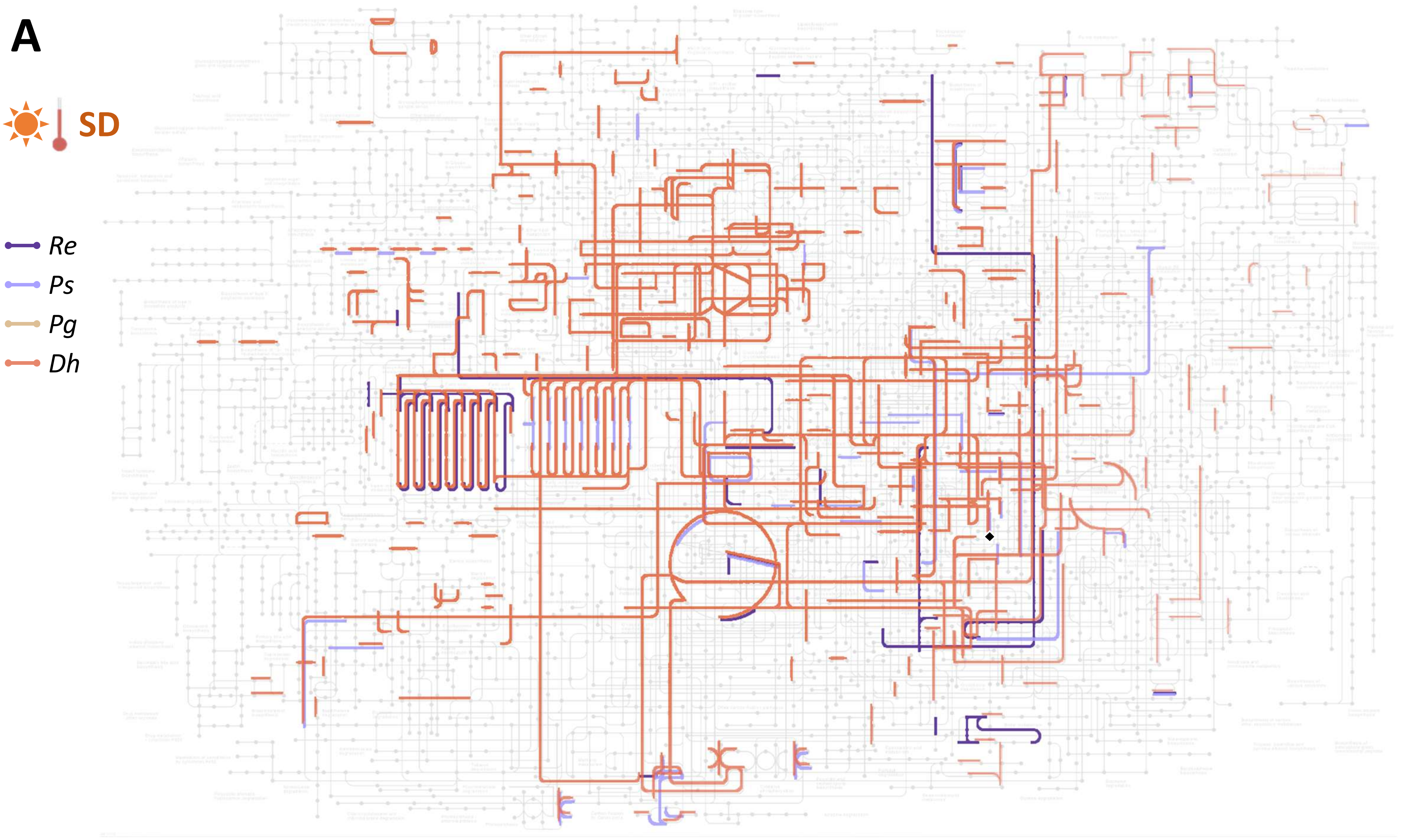
A
SD
Re
Ps
Pg
Dh

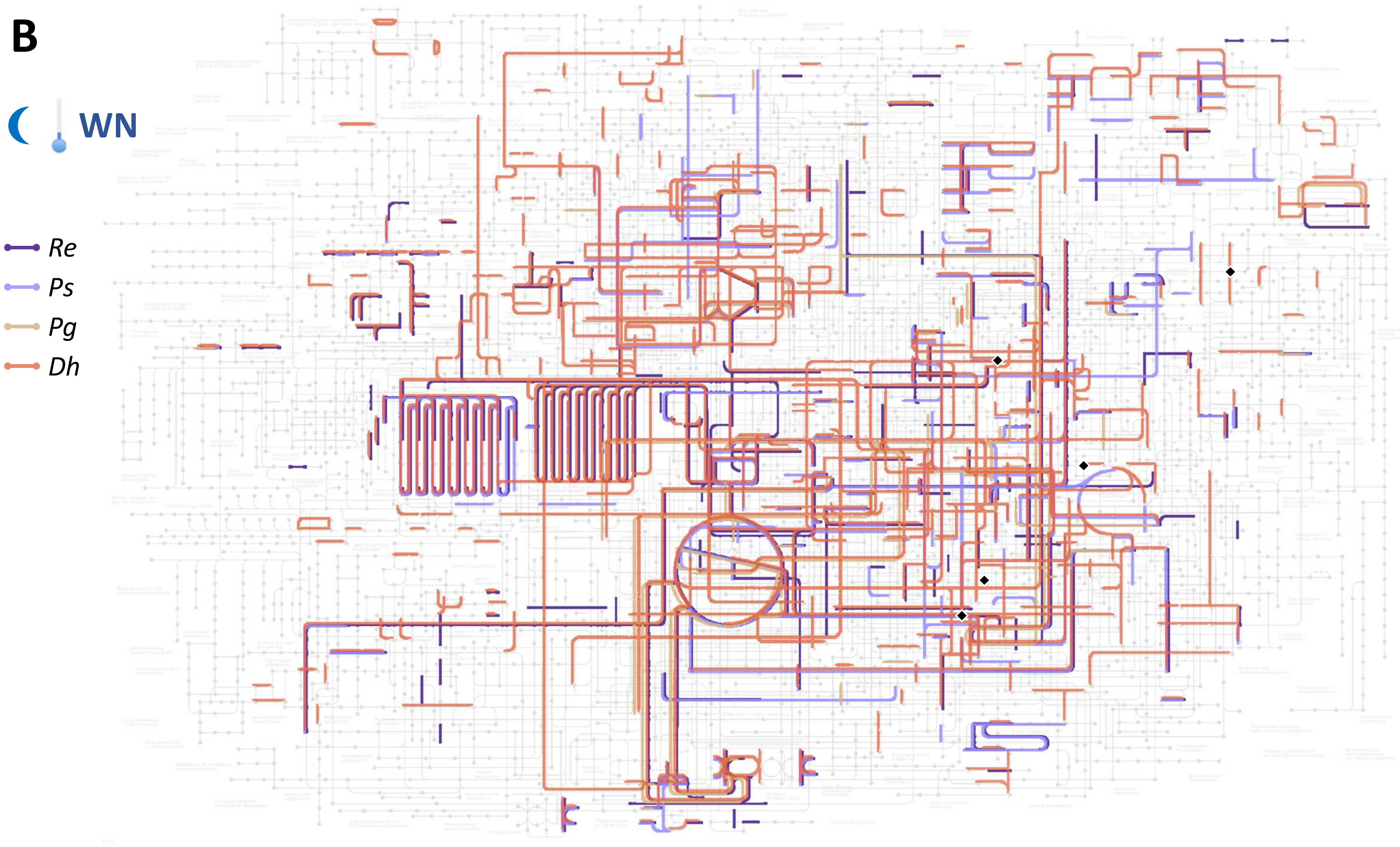
B
WN
Re
Ps
Pg
Dh

Figure S4 Assemblage structure based on transcriptomic data (non-ribosomal reads). Percentage of each species in the filtered counts of each sample that includes triplicates for SD and WN conditions.

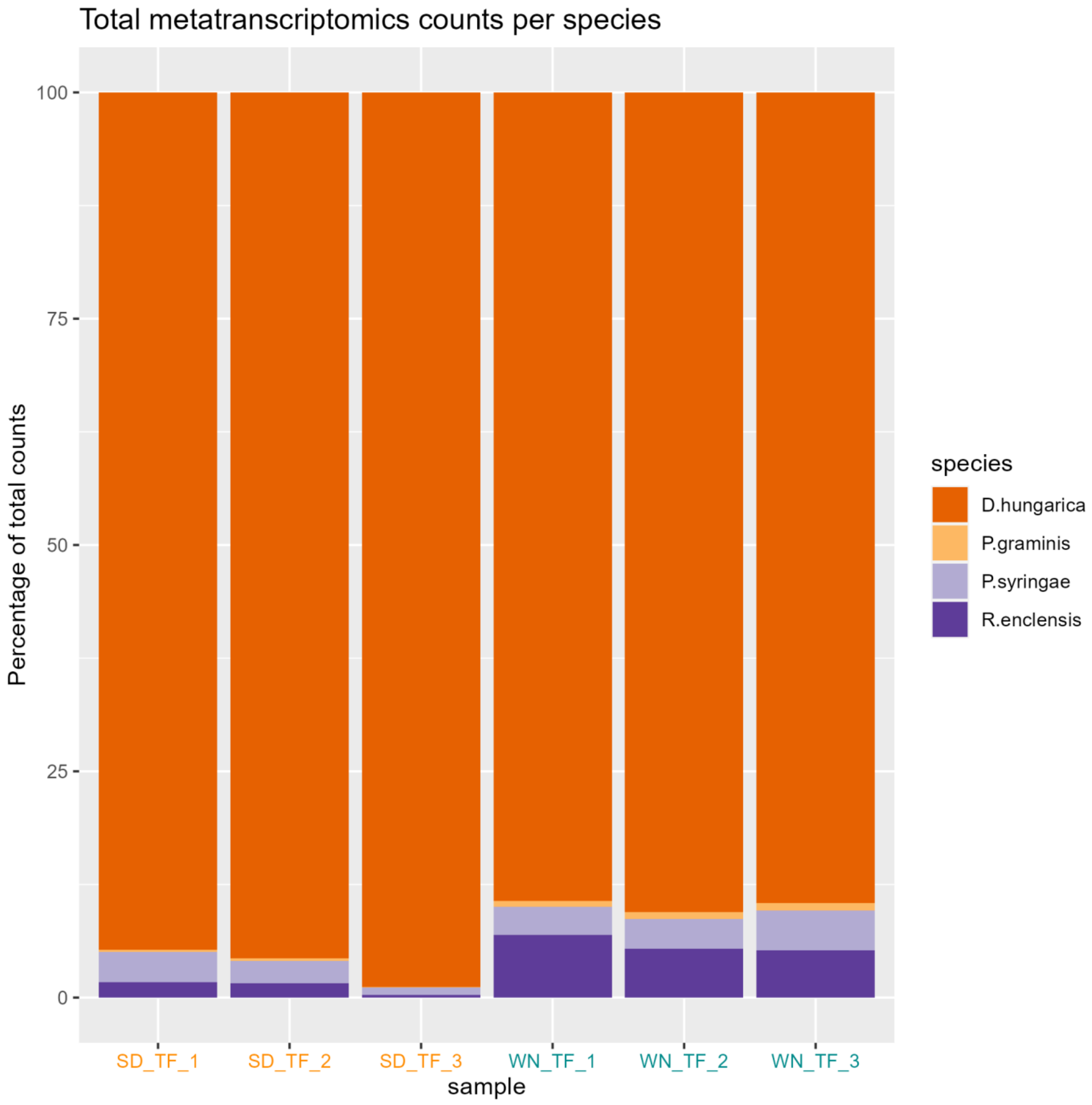

Supplementary Tables

Table S1 Composition of synthetic marine cloud water medium (MCW)

**Compounds**	**Concentration in 5X (MCW) (µM)**
NO_3NH_4	140.0
$MgCl_2$	20.0
K_2SO_4	7.5
$CaCl_2$	40.0
sodium formate	29.0
sodium acetate	20.0
succinic acid	1.5
oxalic acid	4.5
ammonium sulfate	25.0
formaldehyde	25.0
NaOH	80.0
H_2SO_4	40.0

Table S2 Metabolites identified according to LC-MS analysis

metabolite identification	ID	RT (min)	detected mass m/z	theoretical mass m/z	elemental formula	ionic formula	Fold change SD/WN[a]	class[b]	annotation confidence[c]
2-aminobenzoic acid	M138.0552T6.83	6.83	138.06	138.06	$C_7H_7NO_2$	[M+H]+	-inf	II	a,e
DL-methionine sulfoxide	M166.0533T0.94	0.94	166.05	166.05	$C_5H_{11}N_O3_S$	[M+H]+	-inf	I	a,b,c
unknown	M301.0829T6.12	6.12	301.08	unknown	unknown	unknown	-inf	IV	
unknown	M407.5501T11.06	11.06	407.55	unknown	unknown	unknown	-inf	IV	
unknown	M556.1378T1.21	1.21	556.14	unknown	unknown	unknown	-inf	IV	
unknown	M557.1949T6.12	6.12	557.19	unknown	unknown	unknown	-inf	IV	
unknown	M279.1009T6.12	6.12	279.10	unknown	unknown	unknown	-106.79	IV	
unknown	M145.1084T1.07	1.07	145.11	unknown	unknown	unknown	-98.57	IV	
unknown	M261.1445T7.67	7.67	261.14	unknown	unknown	unknown	-56.30	IV	
pyridoxal	M168.0658T1.2	1.20	168.07	168.07	$C_8H_9NO_3$	[M+H]+	-13.50	I	a,b,c
D-pantothenic acid	M220.118T6.95	6.95	220.12	220.12	$C_9H_{17}NO_5$	[M+H]+	-8.70	I	a,b,c
N^6-acetyl-L-lysine	M189.1235T1.19_2	1.19	189.12	189.12	$C_8H_{16}N_2O_3$	[M+H]+	-8.30	I	a,b,c
L-glutamic acid	M148.0605T0.94	0.94	148.06	148.06	$C_5H_9NO_4$	[M-H]-	-2.38	I	a,b,c
L-isoleucine	M132.102T2.04	2.04	132.10	132.10	$C_6H_{13}NO_2$	[M+H]+	2.28	I	a,b,c
unknown	M116.0707T0.99	0.99	116.07	unknown	unknown	unknown	2.86	IV	
butyryl-L-carnitine	M232.1544T7.07	7.07	232.15	232.15	$C_{11}H_{21}O_4$	[M+H]+	3.00	I	a,b,c
unknown	M132.1019T1.21	1.21	132.10	unknown	unknown	unknown	3.85	IV	
acetyl-L-carnitine	M204.1231T1.21	1.21	204.12	204.12	$C_9H_{17}NO_4$	[M+H]+	4.10	I	a,b,c
unknown	M245.1133T1.18	1.18	245.11	unknown	unknown	unknown	5.58	IV	
unknown	M385.6536T6.31	6.31	385.65	unknown	unknown	unknown	17.19	IV	
unknown	M816.0793T8.33	8.33	816.08	unknown	unknown	unknown	19.37	IV	
unknown	M291.1187T1.14	1.14	291.12	unknown	unknown	unknown	99.80	IV	
isovaleryl-L-carnitine	M246.17T8.11	8.11	246.17	246,17	$C_{12}H_{24}NO_4$	[M+H]+	109.60	I	a,b,c
unknown	M146.1177T6.99	6.99	146.12	unknown	unknown	unknown	+inf	IV	
unknown	M246.1701T8.22	8.22	246.17	unknown	unknown	unknown	+inf	IV	

[a] Ratio SD (in the presence of hydrogen peroxide at 17°C) versus WN (at 5°C without hydrogen peroxide) were determined from relative intensities into profiles. -inf and +inf, metabolite only detected in WN or SD condition, respectively.

[b] Metabolite identification class I: identified compound, II: putatively annotated compound and IV: unknown compound, according to [#].

[c] a for accurate mass (MS), b for retention time, c for consistent MS/MS, d means consistent MS/MS with external database and e means consistent MS/MS with fragmentation interpretation

[#] Sumner LW, Amberg A, Barrett D, Beale MH, Beger R, Daykin CA, et al. Proposed minimum reporting standards for chemical analysis: Chemical Analysis Working Group (CAWG) Metabolomics Standards Initiative (MSI). *Metabolomics* 2007;**3**:211–221. https://doi.org/10.1007/s11306-007-0082-2

Table S3 Differentially abundant genes between SD and WN cloud-like conditions

(Excel file)

Supplementary Material 1: Statistical analysis report for 'Multi-kingdom microbial assemblage modulates its metabolism under contrasted cloud conditions'.

(html file)
