# Supplementary material for: Multi-kingdom microbial assemblage modulates its metabolism under contrasted cloud conditions
Source: ISME Commun. 2025 Nov 3;5(1):ycaf200. doi: 10.1093/ismeco/ycaf200 (PMC12721874; doi:10.1093/ismeco/ycaf200)
Supplement: Supplementary_material_1_ycaf200 [file supplementary_material_1_ycaf200.html]

Statistical analysis report for ‘Multi-kingdom microbial assemblage modulates its metabolism under contrasted cloud conditions’.


Code 

- Show All Code
- Hide All Code
- Download Rmd

# Statistical analysis report for ‘Multi-kingdom microbial assemblage modulates its metabolism under contrasted cloud conditions’.

#### Domitille Jarrige

#### 2025-10-27

```
setwd("C:/Users/djarrige/Desktop/Domitille/projets/Anciens_projets/METACLOUD/scripts/")
```

# Libraries

# Variables

```
vect_COG_category_long <- c("A" = "A: RNA processing and modification",
                            "B" = "B: Chromatin Structure and dynamics",
                            "C" = "C: Energy production and conversion",
                            "D" = "D: Cell cycle control, cell division, chromosome partitioning",
                            "E" = "E: Amino Acid transport and metabolism",
                            "F" = "F: Nucleotide transport and metabolism",
                            "G" = "G: Carbohydrate transport and metabolism",
                            "H" = "H: Coenzyme transport and metabolism",
                            "I" = "I: Lipid transport and metabolism",
                            "J" = "J: Translation, ribosomal structure and biogenesis",
                            "K" = "K: Transcription",
                            "L" = "L: Replication, recombination and repair",
                            "M" = "M: Cell wall/membrane/envelope biogenesis",
                            "N" = "N: Cell motility",
                            "O" = "O: Post-translational modification, protein turnover, chaperones",
                            "P" = "P: Inorganic ion transport and metabolism",
                            "Q" = "Q: Secondary metabolites biosynthesis, transport and catabolism",
                            "T" = "T: Signal Transduction mechanisms",
                            "U" = "U: Intracellular trafficking, secretion and vesicular transport",
                            "V" = "V: Defense mechanisms",
                            "W" = "W: Extracellular structures",
                            "X" = "X: Mobilome: prophages, transposons",
                            "Y" = "Y: Nuclear structure",
                            "Z" = "Z: Cytoskeleton",
                            "R" = "R: General functional prediction only",
                            "S" = "S: Function unknown" #,
                            # "-" = "-: Other"
                            )


COG_colours <- hue_pal()(length(vect_COG_category_long))
names(COG_colours) <- vect_COG_category_long

species_colours <- c("D.hungarica" = "#e66101", "P.graminis" = "#fdb863", 
                     "P.syringae" = "#b2abd2", "R.enclensis" = "#5e3c99")
```

# Functions

```
compute_deseq2_analysis = function(myData, sample_data, subset_var=FALSE, select=FALSE, 
                                   contrast_col, ref, tested){
  data <- myData
  sample <- sample_data
  if ((subset_var != FALSE) & (select != FALSE)){
    sample %>% filter(!!as.name(subset_var) == select) -> sample
  }
  
  sample[[contrast_col]] <- factor(sample[[contrast_col]])
  data <- data[row.names(sample)]
  
  data_matrix <- round(as.matrix(data))
  d = formula(paste("~", " ", contrast_col))
  
  dds <- DESeqDataSetFromMatrix(countData = data_matrix, 
                                colData = sample,
                                design = d)
  dds <- estimateSizeFactors(dds)
  dds <- DESeq(dds)
  resultsNames(dds)
  resDESeq <- results(dds, contrast = c(contrast_col, tested, ref),
                      independentFiltering = TRUE, alpha=0.1)
  
  resDESeq <- resDESeq[order(resDESeq$padj),]
  res <- data.frame(resDESeq)
  res$gene = row.names(res)
  res$condition = select
  res$SAMPLE_COMPARISON = paste0(tested, "_VS_", ref) 
  
  return(res)
}
```

# Experimental setup

Comparison of an artificial microbial assemblage gene expression
under two cloud-like conditions:

- **summer day (SD):** light, 250µM H2O2,
  17°C
- **winter night (WN):** dark, no added H2O2,
  5°C

# Bioinformatic workflow and mapping overview

The metatranscriptomics data was processed using a custom made
Snakemake workflow.

Two sequencing runs were performed by Genoscreen (Lille, France) as
the first run produced relatively low quality reads. Both runs are added
into a single dataset in our analyses.

After quality control steps and read cleaning (report)
remaining rRNA (not depleted prior to sequencing) were removed with
sortmeRNA. As fungal rRNA were not depleted in our libraries, the vast
majority of our reads correspond to *Dioszegia*
*hungarica* rRNAs.

Non ribosomal and ribosomal RNAs were then mapped separately on our
reference genomes using STAR.

For non rRNA reads: **~90-95% of reads mapped in biological
samples**. In blank samples, around 40% of reads were mapped, and
only partially.

Few reads were mapped on *P. graminis* PDD-13b-3 genome. It
will be difficult to get significant results for this species.

Lastly, for each gene of the assemblage, read counts were obtained
with featureCounts.’-M’ and ‘–fraction’ options were used to count
multi-mapping reads fractionally (if a read maps on x features: each
feature gets 1/x counts).

# Analyses

##### Load counts and annotation data

```
counts_table <- read.csv("../results/all_counts_community_artificial_no_rRNA.tsv", sep = "\t", row.names = "Geneid")

metadata_table <- read.csv("../data/metadata.txt", sep = "\t", row.names = "name") # row.names = "sample"


counts_table %>% rename("WN_TF_1" = "S_5C1", 
                        "WN_TF_2" = "S_5C2", 
                        "WN_TF_3" = "S_5C3",
                        "WN_BLK" = "S_5BLK",
                        "SD_TF_1" = "S_17C1", 
                        "SD_TF_2" = "S_17C2", 
                        "SD_TF_3" = "S_17C3",
                        "SD_BLK" = "S_17BLK") -> counts_table

annotation_table <- read.csv("../data/annotations_final_community_updated.tsv", sep="\t", row.names = "Geneid")
annotation_all <- read.csv("../data/annotations_final_community_updated.tsv", sep="\t", row.names = "Geneid")
annotation_table_long <- read.csv("../data/annotations_final_community_long2.tsv", sep="\t", row.names = "Geneid")

## added july 2024
counts_table <- counts_table[intersect(rownames(counts_table), rownames(annotation_all)),]


annotation_diohu <- read.csv("../data/dioszegia_kegg.tsv", sep="\t", row.names = "Geneid")
annotation_psegr <- read.csv("../data/pseudomonas_graminis_kegg.tsv", sep="\t", row.names = "Geneid")
annotation_psesy <- read.csv("../data/pseudomonas_syringae_kegg.tsv", sep="\t", row.names = "Geneid")
annotation_rhoen <- read.csv("../data/rhodococcus_kegg.tsv", sep="\t", row.names = "Geneid")

chem_data_metaT <- read.csv("../data/formaldehyde_evolution_transcriptomics.txt", sep="\t")
chem_data_metaB <- read.csv("../data/formaldehyde_evolution_metabolomics.txt", sep="\t")

## Data for figure

annotation_table_fig <- readxl::read_excel("../data/Table_S3_DEGs_annotations.xlsx")
rename(annotation_table_fig, "COG_category" = "COG category", 
       "COG_category_long" = "COG category long",
       "COG_process" = "COG process") -> annotation_table_fig

metabolomics_df <- read.csv("../data/metabolomics.txt", sep="\t", row.names = 1)

# keep a version of the metabolomics dataset with all time points
metabolomics_df -> metabolomics_all_times_df
metabolomics_all_times_df %>% t() %>% data.frame() -> metabolomics_all_times_df

metabolomics_all_times_df %>% rename("WN_T0_1" = "S5C_T0_1", 
                                     "WN_T0_2" = "S5C_T0_2", 
                                     "WN_T0_3" = "S5C_T0_3",
                                     "SD_T0_1" = "S17C_T0_1", 
                                     "SD_T0_2" = "S17C_T0_2", 
                                     "SD_T0_3" = "S17C_T0_3",
                                     "WN_TF_1" = "S5C_TF_1", 
                                     "WN_TF_2" = "S5C_TF_2", 
                                     "WN_TF_3" = "S5C_TF_3",
                                     "SD_TF_1" = "S17C_TF_1", 
                                     "SD_TF_2" = "S17C_TF_2", 
                                     "SD_TF_3" = "S17C_TF_3") -> metabolomics_all_times_df

metabolomics_all_times_df <- metabolomics_all_times_df[,c("WN_T0_1", "WN_T0_2", "WN_T0_3", "SD_T0_1", "SD_T0_2", "SD_T0_3",
                                                          "WN_TF_1", "WN_TF_2", "WN_TF_3", "SD_TF_1", "SD_TF_2", "SD_TF_3")]

metadata_table_metaB_all_times <- read.csv("../data/metadata_metaB.txt", sep = "\t", row.names = "name") # row.names = "sample"

# Keep final time points only, like for metatranscriptomics
metabolomics_df  %>% t() %>% 
  data.frame() %>% select(starts_with(c("S17C_TF_", "S5C_TF_"))) -> metabolomics_df


metabolomics_df %>% rename("WN_TF_1" = "S5C_TF_1", 
                           "WN_TF_2" = "S5C_TF_2", 
                           "WN_TF_3" = "S5C_TF_3",
                           "SD_TF_1" = "S17C_TF_1", 
                           "SD_TF_2" = "S17C_TF_2", 
                           "SD_TF_3" = "S17C_TF_3") -> metabolomics_df

metabolomics_box_plot_df <- readxl::read_excel("../results/metabolomics_annotation_boxplot.xlsx")

metabolomics_annotations <- readxl::read_excel("../results/Table_S2_Metabolites_identified.xlsx", n_max = 25)
```

##### Removal of blank samples

```
counts_table <- counts_table[,c("WN_TF_1", "WN_TF_2", "WN_TF_3", "SD_TF_1", "SD_TF_2", "SD_TF_3")]
metadata_table <- metadata_table[c("WN_TF_1", "WN_TF_2", "WN_TF_3", "SD_TF_1", "SD_TF_2", "SD_TF_3"),]
```

## Chemical properties of the samples

### Formaldehyde evolution

```
chem_data_metaT$experiment <- "metaT"
chem_data_metaB$experiment <- "metaB"

chem_data <- rbind(chem_data_metaT, chem_data_metaB)


## Metatranscriptomics data

chem_data[chem_data$color == "#89DDF8" & chem_data$experiment == "metaT",] -> tmp1
group_by(tmp1, sampling_time) %>% summarise(formaldehyde_mean=mean(formaldehyde_ratio_to_initial), 
                                            formaldehyde_sd=sd(formaldehyde_ratio_to_initial)) -> tmp1
tmp1$color <- "#89DDF8"
tmp1$condition <- "WN"
tmp1$category <- "biotic_WN"
tmp1$experiment <- "metaT"

chem_data[chem_data$color == "#F8AD18" & chem_data$experiment == "metaT",] -> tmp2
group_by(tmp2, sampling_time) %>% summarise(formaldehyde_mean=mean(formaldehyde_ratio_to_initial), 
                                            formaldehyde_sd=sd(formaldehyde_ratio_to_initial)) -> tmp2
tmp2$color <- "#F8AD18"
tmp2$condition <- "SD"
tmp2$category <- "biotic_SD"
tmp2$experiment <- "metaT"

chem_data[chem_data$color == "#A6C1CA" & chem_data$experiment == "metaT",] -> tmp3
group_by(tmp3, sampling_time) %>% summarise(formaldehyde_mean=mean(formaldehyde_ratio_to_initial), 
                                            formaldehyde_sd=sd(formaldehyde_ratio_to_initial)) -> tmp3
tmp3$color <- "#A6C1CA"
tmp3$condition <- "WN"
tmp3$category <- "abiotic_WN"
tmp3$experiment <- "metaT"

chem_data[chem_data$color == "#C4B69B" & chem_data$experiment == "metaT",] -> tmp4
group_by(tmp4, sampling_time) %>% summarise(formaldehyde_mean=mean(formaldehyde_ratio_to_initial), 
                                            formaldehyde_sd=sd(formaldehyde_ratio_to_initial)) -> tmp4
tmp4$color <- "#C4B69B"
tmp4$condition <- "SD"
tmp4$category <- "abiotic_SD"
tmp4$experiment <- "metaT"

## Metabolomics data

chem_data[chem_data$color == "#89DDF8" & chem_data$experiment == "metaB",] -> tmp5
group_by(tmp5, sampling_time) %>% summarise(formaldehyde_mean=mean(formaldehyde_ratio_to_initial), 
                                            formaldehyde_sd=sd(formaldehyde_ratio_to_initial)) -> tmp5
tmp5$color <- "#89DDF8"
tmp5$condition <- "WN"
tmp5$category <- "biotic_WN"
tmp5$experiment <- "metaB"

chem_data[chem_data$color == "#F8AD18" & chem_data$experiment == "metaB",] -> tmp6
group_by(tmp6, sampling_time) %>% summarise(formaldehyde_mean=mean(formaldehyde_ratio_to_initial), 
                                            formaldehyde_sd=sd(formaldehyde_ratio_to_initial)) -> tmp6
tmp6$color <- "#F8AD18"
tmp6$condition <- "SD"
tmp6$category <- "biotic_SD"
tmp6$experiment <- "metaB"

chem_data[chem_data$color == "#A6C1CA" & chem_data$experiment == "metaB",] -> tmp7
group_by(tmp7, sampling_time) %>% summarise(formaldehyde_mean=mean(formaldehyde_ratio_to_initial), 
                                            formaldehyde_sd=sd(formaldehyde_ratio_to_initial)) -> tmp7
tmp7$color <- "#A6C1CA"
tmp7$condition <- "WN"
tmp7$category <- "abiotic_WN"
tmp7$experiment <- "metaB"

chem_data[chem_data$color == "#C4B69B" & chem_data$experiment == "metaB",] -> tmp8
group_by(tmp8, sampling_time) %>% summarise(formaldehyde_mean=mean(formaldehyde_ratio_to_initial), 
                                            formaldehyde_sd=sd(formaldehyde_ratio_to_initial)) -> tmp8
tmp8$color <- "#C4B69B"
tmp8$condition <- "SD"
tmp8$category <- "abiotic_SD"
tmp8$experiment <- "metaB"


## Merge data

line_data <- rbind(tmp1, tmp2, tmp3, tmp4, tmp5, tmp6, tmp7, tmp8)
rm(tmp1, tmp2, tmp3, tmp4, tmp5, tmp6, tmp7, tmp8)


## Test difference between biotic and abiotic


kruskal_formaldehyde <- c("",
                          try(if(kruskal.test(x = c(chem_data_metaB[(chem_data_metaB$sampling_time == 1 &
                                                                     chem_data_metaB$color == "#F8AD18"),
                                                                    "formaldehyde_ratio_to_initial"],
                                                    chem_data_metaB[(chem_data_metaB$sampling_time == 1 &
                                                                     chem_data_metaB$color == "#C4B69B"),
                                                                    "formaldehyde_ratio_to_initial"]),
                                              g = c(rep("biotic_SD", 3), rep("abiotic_SD", 3))
                          )[["p.value"]] < 0.05){"*"}else{""}, silent = TRUE) ,
                          try(if(kruskal.test(x = c(chem_data_metaB[(chem_data_metaB$sampling_time == 2 &
                                                                     chem_data_metaB$color == "#F8AD18"),
                                                                    "formaldehyde_ratio_to_initial"],
                                                    chem_data_metaB[(chem_data_metaB$sampling_time == 2 &
                                                                     chem_data_metaB$color == "#C4B69B"),
                                                                    "formaldehyde_ratio_to_initial"]),
                                              g = c(rep("biotic_SD", 3), rep("abiotic_SD", 3))
                          )[["p.value"]] < 0.05){"*"}else{""}, silent = TRUE),
                          try(if(kruskal.test(x = c(chem_data_metaB[(chem_data_metaB$sampling_time == 3 &
                                                                     chem_data_metaB$color == "#F8AD18"),
                                                                    "formaldehyde_ratio_to_initial"],
                                                    chem_data_metaB[(chem_data_metaB$sampling_time == 3 &
                                                                     chem_data_metaB$color == "#C4B69B"),
                                                                    "formaldehyde_ratio_to_initial"]),
                                              g = c(rep("biotic_SD", 3), rep("abiotic_SD", 3))
                          )[["p.value"]] < 0.05){"*"}else{""}, silent = TRUE),
                          "",
                          try(if(kruskal.test(x = c(chem_data_metaT[(chem_data_metaT$sampling_time == 1 &
                                                                     chem_data_metaT$color == "#F8AD18"),
                                                                    "formaldehyde_ratio_to_initial"],
                                                    chem_data_metaT[(chem_data_metaT$sampling_time == 1 &
                                                                     chem_data_metaT$color == "#C4B69B"),
                                                                    "formaldehyde_ratio_to_initial"]),
                                              g = c(rep("biotic_SD", 3), rep("abiotic_SD", 3))
                          )[["p.value"]] < 0.05){"*"}else{""}, silent = TRUE) ,
                          try(if(kruskal.test(x = c(chem_data_metaT[(chem_data_metaT$sampling_time == 2 &
                                                                     chem_data_metaT$color == "#F8AD18"),
                                                                    "formaldehyde_ratio_to_initial"],
                                                    chem_data_metaT[(chem_data_metaT$sampling_time == 2 &
                                                                     chem_data_metaT$color == "#C4B69B"),
                                                                    "formaldehyde_ratio_to_initial"]),
                                              g = c(rep("biotic_SD", 3), rep("abiotic_SD", 3))
                          )[["p.value"]] < 0.05){"*"}else{""}, silent = TRUE),
                          try(if(kruskal.test(x = c(chem_data_metaT[(chem_data_metaT$sampling_time == 3 &
                                                                     chem_data_metaT$color == "#F8AD18"),
                                                                    "formaldehyde_ratio_to_initial"],
                                                    chem_data_metaT[(chem_data_metaT$sampling_time == 3 &
                                                                     chem_data_metaT$color == "#C4B69B"),
                                                                    "formaldehyde_ratio_to_initial"]),
                                              g = c(rep("biotic_SD", 3), rep("abiotic_SD", 3))
                          )[["p.value"]] < 0.05){"*"}else{""}, silent = TRUE),
                          try(if(kruskal.test(x = c(chem_data_metaT[(chem_data_metaT$sampling_time == 5 &
                                                                     chem_data_metaT$color == "#F8AD18"),
                                                                    "formaldehyde_ratio_to_initial"],
                                                    chem_data_metaT[(chem_data_metaT$sampling_time == 5 &
                                                                     chem_data_metaT$color == "#C4B69B"),
                                                                    "formaldehyde_ratio_to_initial"]),
                                              g = c(rep("biotic_SD", 3), rep("abiotic_SD", 3))
                          )[["p.value"]] < 0.05){"*"}else{""}, silent = TRUE),
                          "",
                          try(if(kruskal.test(x = c(chem_data_metaB[(chem_data_metaB$sampling_time == 1 &
                                                                     chem_data_metaB$color == "#89DDF8"),
                                                                    "formaldehyde_ratio_to_initial"],
                                                    chem_data_metaB[(chem_data_metaB$sampling_time == 1 &
                                                                     chem_data_metaB$color == "#A6C1CA"),
                                                                    "formaldehyde_ratio_to_initial"]),
                                              g = c(rep("biotic_WN", 3), rep("abiotic_WN", 3))
                          )[["p.value"]] < 0.05){"*"}else{""}, silent = TRUE) ,
                          try(if(kruskal.test(x = c(chem_data_metaB[(chem_data_metaB$sampling_time == 2 &
                                                                     chem_data_metaB$color == "#89DDF8"),
                                                                    "formaldehyde_ratio_to_initial"],
                                                    chem_data_metaB[(chem_data_metaB$sampling_time == 2 &
                                                                     chem_data_metaB$color == "#A6C1CA"),
                                                                    "formaldehyde_ratio_to_initial"]),
                                              g = c(rep("biotic_WN", 3), rep("abiotic_WN", 3))
                          )[["p.value"]] < 0.05){"*"}else{""}, silent = TRUE),
                          try(if(kruskal.test(x = c(chem_data_metaB[(chem_data_metaB$sampling_time == 3 &
                                                                     chem_data_metaB$color == "#89DDF8"),
                                                                    "formaldehyde_ratio_to_initial"],
                                                    chem_data_metaB[(chem_data_metaB$sampling_time == 3 &
                                                                     chem_data_metaB$color == "#A6C1CA"),
                                                                    "formaldehyde_ratio_to_initial"]),
                                              g = c(rep("biotic_WN", 3), rep("abiotic_WN", 3))
                          )[["p.value"]] < 0.05){"*"}else{""}, silent = TRUE),
                          "",
                          try(if(kruskal.test(x = c(chem_data_metaT[(chem_data_metaT$sampling_time == 1 &
                                                                     chem_data_metaT$color == "#89DDF8"),
                                                                    "formaldehyde_ratio_to_initial"],
                                                    chem_data_metaT[(chem_data_metaT$sampling_time == 1 &
                                                                     chem_data_metaT$color == "#A6C1CA"),
                                                                    "formaldehyde_ratio_to_initial"]),
                                              g = c(rep("biotic_WN", 3), rep("abiotic_WN", 3))
                          )[["p.value"]] < 0.05){"*"}else{""}, silent = TRUE) ,
                          try(if(kruskal.test(x = c(chem_data_metaT[(chem_data_metaT$sampling_time == 2 &
                                                                     chem_data_metaT$color == "#89DDF8"),
                                                                    "formaldehyde_ratio_to_initial"],
                                                    chem_data_metaT[(chem_data_metaT$sampling_time == 2 &
                                                                     chem_data_metaT$color == "#A6C1CA"),
                                                                    "formaldehyde_ratio_to_initial"]),
                                              g = c(rep("biotic_WN", 3), rep("abiotic_WN", 3))
                          )[["p.value"]] < 0.05){"*"}else{""}, silent = TRUE),
                          try(if(kruskal.test(x = c(chem_data_metaT[(chem_data_metaT$sampling_time == 3 &
                                                                     chem_data_metaT$color == "#89DDF8"),
                                                                    "formaldehyde_ratio_to_initial"],
                                                    chem_data_metaT[(chem_data_metaT$sampling_time == 3 &
                                                                     chem_data_metaT$color == "#A6C1CA"),
                                                                    "formaldehyde_ratio_to_initial"]),
                                              g = c(rep("biotic_WN", 3), rep("abiotic_WN", 3))
                          )[["p.value"]] < 0.05){"*"}else{""}, silent = TRUE),
                          try(if(kruskal.test(x = c(chem_data_metaT[(chem_data_metaT$sampling_time == 5 &
                                                                     chem_data_metaT$color == "#89DDF8"),
                                                                    "formaldehyde_ratio_to_initial"],
                                                    chem_data_metaT[(chem_data_metaT$sampling_time == 5 &
                                                                     chem_data_metaT$color == "#A6C1CA"),
                                                                    "formaldehyde_ratio_to_initial"]),
                                              g = c(rep("biotic_WN", 3), rep("abiotic_WN", 3))
                          )[["p.value"]] < 0.05){"*"}else{""}, silent = TRUE)
)

## Test difference between time 0 an the other time points

kruskal_df <- data.frame(time = rep(c(0, 1, 2, 3, 0, 1, 2, 3, 5), 2),
                         height = rep(0, 18),
                         experiment = rep(c(rep("metaB", 4), rep("metaT", 5)), 2),
                         condition = c(rep("SD", 9), rep("WN", 9)),
                         category = c(rep("community SD", 9), rep("community WN", 9)),
                         color = c(rep("#F8AD18", 9), rep("#89DDF8", 9)),
                         pvalue = kruskal_formaldehyde)


krusk <- kruskal_df


## Segment data for sampling time

segment_data <- data.frame(x = c(4, 0, 3.5), xend = c(4, 0, 3.5), 
                           y = c(-Inf, -Inf, -Inf), yend = c(Inf, Inf, Inf),
                           experiment = c("metaT", "metaB", "metaB"))
  
chem_data %>% ggplot() +
  geom_point(aes(x=sampling_time, y=formaldehyde_ratio_to_initial, color=color)) +
  ylim(c(-0.1, 1.2)) +
  xlim(c(0, 5)) +
  scale_colour_identity("Category", labels = c("Assemblage Winter Night", "Abiotic Winter Night",
                                               "Abiotic Summer Day", "Assemblage Summer Day"), 
                        guide="legend") +
  scale_fill_identity() +
  ylab(label = "Relative formaldehyde evolution") +
  xlab(label = "Incubation time (h)") +
  facet_grid(rows = vars(condition), cols = vars(experiment),
             labeller = as_labeller(c("metaT" = "Metatranscriptomics", 
                                      "metaB" = "Meta-metabolomics",
                                      "SD" = "Summer Day",
                                      "WN" = "Winter Night"))) + 
  theme(legend.position = "bottom",
        panel.background = element_rect(fill="#f4f4f4"),
        strip.text = element_text(size=12)) +
  
  
  geom_path(data = line_data[line_data$category == "biotic_SD",], 
            aes(x = sampling_time, y = formaldehyde_mean, color = color), 
            linewidth = 1) + 
  geom_ribbon(data = line_data[line_data$category == "biotic_SD",], 
              aes(x = sampling_time, 
                  ymin = formaldehyde_mean - formaldehyde_sd,
                  ymax = formaldehyde_mean + formaldehyde_sd,
                  fill = color), alpha=0.25, linewidth=0.05) +
  
  geom_path(data = line_data[line_data$category == "abiotic_SD",], 
            aes(x = sampling_time, y = formaldehyde_mean, color = color), 
            linewidth = 1) + 
  geom_ribbon(data = line_data[line_data$category == "abiotic_SD",], 
              aes(x = sampling_time, 
                  ymin = formaldehyde_mean - formaldehyde_sd,
                  ymax = formaldehyde_mean + formaldehyde_sd,
                  fill = color), alpha=0.25, linewidth=0.05) +
  
  geom_path(data = line_data[line_data$category == "biotic_WN",], 
            aes(x = sampling_time, y = formaldehyde_mean, color = color), 
            linewidth = 1) + 
  geom_ribbon(data = line_data[line_data$category == "biotic_WN",], 
              aes(x = sampling_time, 
                  ymin = formaldehyde_mean - formaldehyde_sd,
                  ymax = formaldehyde_mean + formaldehyde_sd,
                  fill = color), alpha=0.25, linewidth=0.05) + 
  
  geom_path(data = line_data[line_data$category == "abiotic_WN",], 
            aes(x = sampling_time, y = formaldehyde_mean, color = color), 
            linewidth = 1) + 
  geom_ribbon(data = line_data[line_data$category == "abiotic_WN",], 
              aes(x = sampling_time, 
                  ymin = formaldehyde_mean - formaldehyde_sd,
                  ymax = formaldehyde_mean + formaldehyde_sd,
                  fill = color), alpha=0.25, linewidth=0.05) + 
  
  # plot significance stars
  geom_text(data=krusk[krusk$category == "community SD",], 
            aes(x = time, y = height, label = pvalue, colour=color), 
            size = 7, show.legend = FALSE, nudge_y = -0.07) +
  geom_text(data=krusk[krusk$category == "community WN",], 
            aes(x = time, y = height, label = pvalue, colour=color), 
            size = 7, show.legend = FALSE, nudge_y = -0.07) +
  geom_text(data=krusk[krusk$category == "abiotic SD",],
            aes(x = time, y = height, label = pvalue, colour=color),
            size = 7, show.legend = FALSE) +
  geom_text(data=krusk[krusk$category == "abiotic WN",],
            aes(x = time, y = height, label = pvalue, colour=color),
            size = 7, show.legend = FALSE) +
  
  geom_segment(data=segment_data, aes(x = x, xend = xend, y = y, yend = yend)) +
  geom_text(data=segment_data, aes(x = x + 0.2, y = 0.20, label = "Cell sampling"), angle=90, size=3) -> plot_formaldehyde

print(plot_formaldehyde)
```

```
# ggsave(plot = plot_formaldehyde, filename = "../figures/formaldehyde_evolution.tiff", dpi = 300, width = 10, height = 6, bg="white")
```

### H2O2 evolution

```
chem_data_metaT$experiment <- "metaT"
chem_data_metaB$experiment <- "metaB"

chem_data <- rbind(chem_data_metaT, chem_data_metaB)


## Metatranscriptomics data

chem_data[chem_data$color == "#F8AD18" & chem_data$experiment == "metaT",] -> tmp2
group_by(tmp2, sampling_time) %>% summarise(H2O2_mean=mean(H2O2_ratio_to_initial), 
                                            H2O2_sd=sd(H2O2_ratio_to_initial)) -> tmp2
tmp2$color <- "#F8AD18"
tmp2$condition <- "SD"
tmp2$category <- "biotic_SD"
tmp2$experiment <- "metaT"


chem_data[chem_data$color == "#C4B69B" & chem_data$experiment == "metaT",] -> tmp4
group_by(tmp4, sampling_time) %>% summarise(H2O2_mean=mean(H2O2_ratio_to_initial), 
                                            H2O2_sd=sd(H2O2_ratio_to_initial)) -> tmp4
tmp4$color <- "#C4B69B"
tmp4$condition <- "SD"
tmp4$category <- "abiotic_SD"
tmp4$experiment <- "metaT"


## Metabolomics data

chem_data[chem_data$color == "#F8AD18" & chem_data$experiment == "metaB",] -> tmp6
group_by(tmp6, sampling_time) %>% summarise(H2O2_mean=mean(H2O2_ratio_to_initial), 
                                            H2O2_sd=sd(H2O2_ratio_to_initial)) -> tmp6
tmp6$color <- "#F8AD18"
tmp6$condition <- "SD"
tmp6$category <- "biotic_SD"
tmp6$experiment <- "metaB"


chem_data[chem_data$color == "#C4B69B" & chem_data$experiment == "metaB",] -> tmp8
group_by(tmp8, sampling_time) %>% summarise(H2O2_mean=mean(H2O2_ratio_to_initial), 
                                            H2O2_sd=sd(H2O2_ratio_to_initial)) -> tmp8
tmp8$color <- "#C4B69B"
tmp8$condition <- "SD"
tmp8$category <- "abiotic_SD"
tmp8$experiment <- "metaB"

chem_data$color <- as.factor(chem_data$color)
## Merge data

line_data <- rbind(tmp2, tmp4, tmp6, tmp8)
rm(tmp2, tmp4, tmp6, tmp8)

## Test difference between time 0 an the other time points

kruskal_h2o2 <-  c("",
                          try(if(kruskal.test(x = c(chem_data_metaB[(chem_data_metaB$sampling_time == 1 &
                                                                     chem_data_metaB$color == "#F8AD18"),
                                                                    "H2O2_ratio_to_initial"],
                                                    chem_data_metaB[(chem_data_metaB$sampling_time == 1 &
                                                                     chem_data_metaB$color == "#C4B69B"),
                                                                    "H2O2_ratio_to_initial"]),
                                              g = c(rep("biotic_SD", 3), rep("abiotic_SD", 3))
                          )[["p.value"]] < 0.05){"*"}else{""}, silent = TRUE) ,
                          try(if(kruskal.test(x = c(chem_data_metaB[(chem_data_metaB$sampling_time == 2 &
                                                                     chem_data_metaB$color == "#F8AD18"),
                                                                    "H2O2_ratio_to_initial"],
                                                    chem_data_metaB[(chem_data_metaB$sampling_time == 2 &
                                                                     chem_data_metaB$color == "#C4B69B"),
                                                                    "H2O2_ratio_to_initial"]),
                                              g = c(rep("biotic_SD", 3), rep("abiotic_SD", 3))
                          )[["p.value"]] < 0.05){"*"}else{""}, silent = TRUE),
                          try(if(kruskal.test(x = c(chem_data_metaB[(chem_data_metaB$sampling_time == 3 &
                                                                     chem_data_metaB$color == "#F8AD18"),
                                                                    "H2O2_ratio_to_initial"],
                                                    chem_data_metaB[(chem_data_metaB$sampling_time == 3 &
                                                                     chem_data_metaB$color == "#C4B69B"),
                                                                    "H2O2_ratio_to_initial"]),
                                              g = c(rep("biotic_SD", 3), rep("abiotic_SD", 3))
                          )[["p.value"]] < 0.05){"*"}else{""}, silent = TRUE),
                          "",
                          try(if(kruskal.test(x = c(chem_data_metaT[(chem_data_metaT$sampling_time == 1 &
                                                                     chem_data_metaT$color == "#F8AD18"),
                                                                    "H2O2_ratio_to_initial"],
                                                    chem_data_metaT[(chem_data_metaT$sampling_time == 1 &
                                                                     chem_data_metaT$color == "#C4B69B"),
                                                                    "H2O2_ratio_to_initial"]),
                                              g = c(rep("biotic_SD", 3), rep("abiotic_SD", 3))
                          )[["p.value"]] < 0.05){"*"}else{""}, silent = TRUE) ,
                          try(if(kruskal.test(x = c(chem_data_metaT[(chem_data_metaT$sampling_time == 2 &
                                                                     chem_data_metaT$color == "#F8AD18"),
                                                                    "H2O2_ratio_to_initial"],
                                                    chem_data_metaT[(chem_data_metaT$sampling_time == 2 &
                                                                     chem_data_metaT$color == "#C4B69B"),
                                                                    "H2O2_ratio_to_initial"]),
                                              g = c(rep("biotic_SD", 3), rep("abiotic_SD", 3))
                          )[["p.value"]] < 0.05){"*"}else{""}, silent = TRUE),
                          try(if(kruskal.test(x = c(chem_data_metaT[(chem_data_metaT$sampling_time == 3 &
                                                                     chem_data_metaT$color == "#F8AD18"),
                                                                    "H2O2_ratio_to_initial"],
                                                    chem_data_metaT[(chem_data_metaT$sampling_time == 3 &
                                                                     chem_data_metaT$color == "#C4B69B"),
                                                                    "H2O2_ratio_to_initial"]),
                                              g = c(rep("biotic_SD", 3), rep("abiotic_SD", 3))
                          )[["p.value"]] < 0.05){"*"}else{""}, silent = TRUE),
                          try(if(kruskal.test(x = c(chem_data_metaT[(chem_data_metaT$sampling_time == 5 &
                                                                     chem_data_metaT$color == "#F8AD18"),
                                                                    "H2O2_ratio_to_initial"],
                                                    chem_data_metaT[(chem_data_metaT$sampling_time == 5 &
                                                                     chem_data_metaT$color == "#C4B69B"),
                                                                    "H2O2_ratio_to_initial"]),
                                              g = c(rep("biotic_SD", 3), rep("abiotic_SD", 3))
                          )[["p.value"]] < 0.05){"*"}else{""}, silent = TRUE)
                     )

kruskal_df <- data.frame(time = c(0, 1, 2, 3, 0, 1, 2, 3, 5),
                         height = rep(0, 9),
                         experiment = c(rep("metaB", 4), rep("metaT", 5)),
                         condition = rep("SD", 9),
                         category = rep("community SD", 9),
                         color = rep("#F8AD18", 9),
                         pvalue = kruskal_h2o2)


krusk <- kruskal_df


## Segment data for sampling time

segment_data <- data.frame(x = c(4, 0, 3.5), xend = c(4, 0, 3.5), 
                           y = c(-Inf, -Inf, -Inf), yend = c(Inf, Inf, Inf),
                           experiment = c("metaT", "metaB", "metaB"))
  
chem_data[chem_data$condition == "SD",] %>% ggplot() +
  geom_point(aes(x=sampling_time, y=H2O2_ratio_to_initial, color=color)) +
  ylim(c(-0.1, 1.2)) +
  xlim(c(0, 5)) +
  scale_colour_identity("Category", labels = c("Assemblage Winter Night", "Abiotic Winter Night",
                                               "Abiotic Summer Day", "Assemblage Summer Day"), 
                        guide="legend", drop = FALSE) +
  scale_fill_identity() +

  ylab("Relative H<sub>2</sub>O<sub>2</sub> evolution") +
  xlab(label = "Incubation time (h)") +

  facet_grid(rows = vars(condition), cols = vars(experiment),
             labeller = as_labeller(c("metaT" = "Metatranscriptomics", 
                                      "metaB" = "Meta-metabolomics",
                                      "SD" = "Summer Day",
                                      "WN" = "Winter Night"))) + 
  theme(legend.position = "bottom",
        panel.background = element_rect(fill="#f4f4f4"),
        axis.title.y = element_markdown(),
        strip.text = element_text(size=12)) +
  
  
  geom_path(data = line_data[line_data$category == "biotic_SD",], 
            aes(x = sampling_time, y = H2O2_mean, color = color), 
            linewidth = 1) + 
  geom_ribbon(data = line_data[line_data$category == "biotic_SD",], 
              aes(x = sampling_time, 
                  ymin = H2O2_mean - H2O2_sd,
                  ymax = H2O2_mean + H2O2_sd,
                  fill = color), alpha=0.25, linewidth=0.05) +
  
  geom_path(data = line_data[line_data$category == "abiotic_SD",], 
            aes(x = sampling_time,y =H2O2_mean, color = color), 
            linewidth = 1) + 
  geom_ribbon(data = line_data[line_data$category == "abiotic_SD",], 
              aes(x = sampling_time, 
                  ymin = H2O2_mean - H2O2_sd,
                  ymax = H2O2_mean + H2O2_sd,
                  fill = color), alpha=0.25, linewidth=0.05) +
  
  # plot significance stars
  geom_text(data=krusk[krusk$category == "community SD",], 
            aes(x = time, y = height, label = pvalue, colour=color), 
            size = 7, show.legend = FALSE, nudge_y = -0.07) +
  geom_text(data=krusk[krusk$category == "abiotic SD",],
            aes(x = time, y = height, label = pvalue, colour=color),
            size = 7, show.legend = FALSE) +

  
  # geom_segment(aes(x=4, xend=4, y=-Inf, yend=Inf)) +
  geom_segment(data=segment_data, aes(x = x, xend = xend, y = y, yend = yend)) +
  geom_segment(aes(x = -Inf, xend = Inf, y = 0.2, yend = 0.2), colour = "darkred", linetype = 2) +
  geom_text(data=segment_data, aes(x = x + 0.2, y = 0.42, label = "Cell sampling"), angle=90, size=3) -> plot_h2o2

print(plot_h2o2)
```

```
# ggsave(plot = plot_h2o2, filename = "../figures/H2O2_evolution.tiff", dpi = 300, width = 10, height = 3.6, bg="white")
```

```
plot_chemical <- ggarrange(plot_h2o2, plot_formaldehyde,  
                      labels = c("A", "B"), 
                      common.legend=TRUE,
                      legend="bottom",
                      nrow=2,
                      heights = c(1.1, 1.9))


ggsave(plot = plot_chemical, filename = "../figures/Figure_S1.tiff", dpi = 300, width = 10, height = 10, bg="white")

# print(plot_chemical)
```

## Descriptive statistics on complete assemblage metatranscriptomics and metabolomics data

### Metatranscriptomics

#### Sample-wise statistics (raw metatranscriptomics counts)

```
message("Computing sample-wise statistics on raw counts")
sample_stat_prenorm <- data.frame(
  mean = apply(counts_table, 2, mean, na.rm = TRUE),
  sd = apply(counts_table, 2, sd, na.rm = TRUE),
  iqr = apply(counts_table, 2, IQR, na.rm = TRUE),
  Q1 = apply(counts_table, 2, quantile, p = 0.25, na.rm = TRUE),
  median = apply(counts_table, 2, median, na.rm = TRUE),
  Q3 = apply(counts_table, 2, quantile, p = 0.75, na.rm = TRUE),
  max = apply(counts_table, 2, max, na.rm = TRUE),
  null = apply(counts_table == 0, 2, sum, na.rm = TRUE)
)
```

#### Gene-wise statistics (raw metatranscriptomics counts)

```
message("Computing gene-wise statistics on raw counts")
gene_stat_prenorm <- data.frame(
  mean = apply(counts_table, 1, mean, na.rm = TRUE),
  sd = apply(counts_table, 1, sd, na.rm = TRUE),
  iqr = apply(counts_table, 1, IQR, na.rm = TRUE),
  Q1 = apply(counts_table, 1, quantile, p = 0.25, na.rm = TRUE),
  median = apply(counts_table, 1, median, na.rm = TRUE),
  Q3 = apply(counts_table, 1, quantile, p = 0.75, na.rm = TRUE),
  max = apply(counts_table, 1, max, na.rm = TRUE),
  null = apply(counts_table == 0, 1, sum, na.rm = TRUE)
)
```

#### Zeroes filtering and smoothing inspired by MTXmodel article (Zhang et al. 2021)

```
species <- unlist(unique(annotation_table[,"Organism"]))
samples <- row.names(metadata_table)
```

Gene with 0 counts in more than 70% of samples are deemed unexpressed
and are removed from the dataset.

```
message("Filtering undetected genes")
undetected_genes <- gene_stat_prenorm$null >= ncol(counts_table) * 0.70
print(paste0("Undetected genes (null in >= 70% samples): ", sum(undetected_genes)))
```

[1] “Undetected genes (null in >= 70% samples): 19262”

```
kept_genes <- !undetected_genes
print(paste0("Kept genes: ", sum(kept_genes)))
```

[1] “Kept genes: 7191”

```
## Genes after filtering
counts_filtered <- counts_table[kept_genes, ]
annotation_table <- annotation_all[rownames(counts_filtered),] #row.names()

## Species abundance (sum of all counts)
totals_df <- data.frame()
for (spe in species) {
  spe_geneid <- annotation_table$Organism == spe
  for (samp in samples) {
    totals_df[spe, samp] <- sum(counts_filtered[spe_geneid, samp], na.rm=TRUE)
  }
}
```

```
# no standardisation was used afterall
counts_standard <- counts_filtered
table_gene_expr <- counts_table
table_gene_expr$detected <- "No"
table_gene_expr[kept_genes, "detected"] <- "yes"
table_gene_expr <- rownames_to_column(table_gene_expr)
table_gene_expr <- left_join(table_gene_expr, rownames_to_column(annotation_table_long)[,c("rowname", "locus_tag", "transcriptId")])
```

```
## remaining zeroes smoothing (as in Zhang et al. 2021)

min_val <- apply(counts_standard, 1, function(x) (min(x[x>0])/2))


for (gene in row.names(counts_standard)) {
  counts_standard[gene,][counts_standard[gene,] == 0] <- min_val[gene]
}
```

```
message("Computing sample-wise statistics on filtered counts")
sample_stat_filt <- data.frame(
  mean = apply(counts_standard, 2, mean, na.rm = TRUE),
  sd = apply(counts_standard, 2, sd, na.rm = TRUE),
  iqr = apply(counts_standard, 2, IQR, na.rm = TRUE),
  Q1 = apply(counts_standard, 2, quantile, p = 0.25, na.rm = TRUE),
  median = apply(counts_standard, 2, median, na.rm = TRUE),
  Q3 = apply(counts_standard, 2, quantile, p = 0.75, na.rm = TRUE),
  max = apply(counts_standard, 2, max, na.rm = TRUE),
  null = apply(counts_standard == 0, 2, sum, na.rm = TRUE)
)

kable(sample_stat_filt[0:6, ], caption = "Sample-wise statistics after filtering")
```

Sample-wise statistics after filtering


|  | mean | sd | iqr | Q1 | median | Q3 | max | null |
| --- | --- | --- | --- | --- | --- | --- | --- | --- |
| WN\_TF\_1 | 7.274402 | 153.42034 | 1.745 | 0.250 | 0.750 | 1.995 | 8701.91 | 0 |
| WN\_TF\_2 | 5.798768 | 141.55673 | 0.835 | 0.165 | 0.330 | 1.000 | 6777.67 | 0 |
| WN\_TF\_3 | 7.436439 | 154.65538 | 1.670 | 0.330 | 0.830 | 2.000 | 8361.50 | 0 |
| SD\_TF\_1 | 7.435202 | 192.23869 | 0.830 | 0.170 | 0.375 | 1.000 | 10856.58 | 0 |
| SD\_TF\_2 | 3.346716 | 85.01516 | 0.375 | 0.125 | 0.250 | 0.500 | 4582.83 | 0 |
| SD\_TF\_3 | 10.483380 | 285.44101 | 0.700 | 0.170 | 0.330 | 0.870 | 14318.75 | 0 |

#### CLR (Centered Log ratio) transformation of whole assemblage metatranscriptomics counts

```
counts_clr <- counts_standard

for (samp in samples) {
  counts_clr[,samp] <- clr(counts_standard[,samp])
}
```

```
message("Computing sample-wise statistics on filtered counts")
sample_stat_clr <- data.frame(
  mean = apply(counts_clr, 2, mean, na.rm = TRUE),
  sd = apply(counts_clr, 2, sd, na.rm = TRUE),
  iqr = apply(counts_clr, 2, IQR, na.rm = TRUE),
  Q1 = apply(counts_clr, 2, quantile, p = 0.25, na.rm = TRUE),
  median = apply(counts_clr, 2, median, na.rm = TRUE),
  Q3 = apply(counts_clr, 2, quantile, p = 0.75, na.rm = TRUE),
  max = apply(counts_clr, 2, max, na.rm = TRUE),
  null = apply(counts_clr == 0, 2, sum, na.rm = TRUE)
)

kable(sample_stat_clr[0:6, ], caption = "Sample-wise statistics after CLR transformation")
```

Sample-wise statistics after CLR transformation


|  | mean | sd | iqr | Q1 | median | Q3 | max | null |
| --- | --- | --- | --- | --- | --- | --- | --- | --- |
| WN\_TF\_1 | 0 | 1.366662 | 2.076935 | -1.1413059 | -0.0426936 | 0.9356294 | 9.316286 | 0 |
| WN\_TF\_2 | 0 | 1.246467 | 1.801810 | -0.9619634 | -0.2688162 | 0.8398464 | 9.661235 | 0 |
| WN\_TF\_3 | 0 | 1.357223 | 1.801810 | -0.9519112 | -0.0295782 | 0.8498986 | 9.188144 | 0 |
| SD\_TF\_1 | 0 | 1.220095 | 1.771957 | -0.9654242 | -0.1742966 | 0.8065326 | 10.099059 | 0 |
| SD\_TF\_2 | 0 | 1.061585 | 1.386294 | -0.8828711 | -0.1897239 | 0.5034233 | 9.626643 | 0 |
| SD\_TF\_3 | 0 | 1.204212 | 1.632695 | -0.9117566 | -0.2484624 | 0.7209382 | 10.429525 | 0 |

```
## Gene-wise statistics after normalisation
message("Computing gene-wise statistics on log-transformed and normalised counts")
gene_stat_norm <- data.frame(mean = apply(counts_clr, 1, mean, na.rm=TRUE),
                             var = apply(counts_clr, 1, var, na.rm=TRUE),
                             sd = apply(counts_clr, 1, sd, na.rm=TRUE),
                             iqr = apply(counts_clr, 1, IQR, na.rm=TRUE),
                             min = apply(counts_clr, 1, min, na.rm=TRUE),
                             med = apply(counts_clr, 1, median, na.rm=TRUE),
                             max = apply(counts_clr, 1, max, na.rm=TRUE))

# Ajout du coefficient de variation
gene_stat_norm$coef_var <- (gene_stat_norm$sd / gene_stat_norm$mean)
```

#### Metatranscriptomics counts distribution

```
par(mfrow=c(1,1))
hist(unlist(counts_table),
     breaks = 200,
     cex.axis = 0.7,
     las = 1,
     col = "skyblue",
     xlab = "raw counts",
     main = "Distribution of raw counts")
```

```
par(mfrow=c(1,1))
hist(unlist(counts_clr),
     breaks = 200,
     cex.axis = 0.7,
     las = 1,
     col = "skyblue",
     xlab = "clr-transformed counts",
     main = "Distribution of clr-transformed counts")
```

The distribution of the whole assemblage metatranscriptomics counts
seems close to normal.

```
# Raw data
boxplot(counts_table,
        main = "Raw expression",
        horizontal = TRUE,
        col = metadata_table$color,
        cex = 0.5,
        cex.axis = 0.8,
        las = 1)
```

```
boxplot(counts_clr,
        main = "clr-transformed expression",
        horizontal = TRUE,
        col = metadata_table$color,
        cex = 0.5,
        cex.axis = 0.8,
        las = 1)
```

The CLR transformation centred our data.

#### Assemblage metatranscriptomics counts repartition by species

```
totals_df$species <- row.names((totals_df))
totals_df %>%
    pivot_longer(!species, 
                names_to=c("sample"),
                values_to="total_counts") %>%
  mutate(sample = case_when(sample == "SD_TF_1" ~ "SD_TF_1",
                            sample == "SD_TF_2" ~ "SD_TF_2",
                            sample == "SD_TF_3" ~ "SD_TF_3",
                            sample == "WN_TF_1" ~ "WN_TF_1",
                            sample == "WN_TF_2" ~ "WN_TF_2",
                            sample == "WN_TF_3" ~ "WN_TF_3")) -> totals_df
```

```
ggplot(data=totals_df, mapping=aes(x=sample, y=total_counts, fill=species)) + 
  geom_bar(position="fill", stat="identity") + 
  scale_fill_brewer(palette = "PuOr") +
  labs(y = "Percentage of total counts") +
  scale_y_continuous(labels = as_function(~ 100 * .)) + 
  theme(axis.text.x = element_text(colour = c("SD_TF_1" = "darkorange",
                                              "SD_TF_2" = "darkorange",
                                              "SD_TF_3" = "darkorange",
                                              "WN_TF_1" = "darkcyan",
                                              "WN_TF_2" = "darkcyan",
                                              "WN_TF_3" = "darkcyan"))) + 
  ggtitle("Total metatranscriptomics counts per species") -> p 

print(p)
```

```
ggsave("../figures/Figure_S3_counts_repartition.png", p, dpi = 300)
ggsave("../figures/Figure_S3_counts_repartition.tiff", p, height = 7, width = 7, dpi = 300)
```

As previously noted, *Dioszegia hungarica* counts are largely
dominant in our data. *Pseudomonas graminis* associated counts
are very rare.

##### *D. hungarica* filtered counts repartition by nucleus or mitochondrial origin

```
organelle_df <- data.frame()

mito_geneid <- annotation_table$Chr == "Dioszegia_hungarica_PDD-24b-2_contig_35"
nucl_geneid <- (annotation_table$Organism == "D.hungarica" & annotation_table$Chr != "Dioszegia_hungarica_PDD-24b-2_contig_35")

for (samp in samples) {
    organelle_df["mitochondria", samp] <- sum(counts_filtered[mito_geneid, samp], na.rm=TRUE)
    organelle_df["nucleus", samp] <- sum(counts_filtered[nucl_geneid, samp], na.rm=TRUE)
}

organelle_df$organelle <- row.names((organelle_df))
organelle_df %>%
    pivot_longer(!organelle, 
                names_to=c("sample"),
                values_to="total_counts") %>%
  mutate(sample = case_when(sample == "SD_TF_1" ~ "SD_TF_1",
                            sample == "SD_TF_2" ~ "SD_TF_2",
                            sample == "SD_TF_3" ~ "SD_TF_3",
                            sample == "WN_TF_1" ~ "WN_TF_1",
                            sample == "WN_TF_2" ~ "WN_TF_2",
                            sample == "WN_TF_3" ~ "WN_TF_3")) -> organelle_df

ggplot(data=organelle_df, mapping=aes(x=sample, y=total_counts, fill=organelle)) + 
  geom_bar(position="fill", stat="identity") + 
  scale_fill_brewer(palette = "PuOr") +
  labs(y = "Percentage of total counts") +
  scale_y_continuous(labels = as_function(~ 100 * .)) + 
  theme(axis.text.x = element_text(colour = c("SD_TF_1" = "darkorange",
                                              "SD_TF_2" = "darkorange",
                                              "SD_TF_3" = "darkorange",
                                              "WN_TF_1" = "darkcyan",
                                              "WN_TF_2" = "darkcyan",
                                              "WN_TF_3" = "darkcyan"))) + 
  ggtitle("Total transcript counts per D. hungarica compartment") -> p 

print(p)
```

```
ggsave("../figures/Diohu_counts_repartition.png", p, dpi = 300)
ggsave("../figures/Diohu_counts_repartition.tiff", p, height = 7, width = 7, dpi = 300)
```

**Among *Dioszegia hungarica* counts, a large portion
come from transcripts produced in the mitochondria, especially in SD
conditions.**

### Metabolomics

#### Metabolite-wise statistics (raw data)

```
# message("Computing metabolite-wise statistics on raw counts")

metabolite_stat_prenorm <- data.frame(
  mean = apply(metabolomics_all_times_df, 1, mean, na.rm = TRUE),
  sd = apply(metabolomics_all_times_df, 1, sd, na.rm = TRUE),
  iqr = apply(metabolomics_all_times_df, 1, IQR, na.rm = TRUE),
  Q1 = apply(metabolomics_all_times_df, 1, quantile, p = 0.25, na.rm = TRUE),
  median = apply(metabolomics_all_times_df, 1, median, na.rm = TRUE),
  Q3 = apply(metabolomics_all_times_df, 1, quantile, p = 0.75, na.rm = TRUE),
  max = apply(metabolomics_all_times_df, 1, max, na.rm = TRUE),
  null = apply(metabolomics_all_times_df == 0, 1, sum, na.rm = TRUE)
)

kable(metabolite_stat_prenorm[100:109, ], caption = "Gene-wise statistics before normalisation")
```

Gene-wise statistics before normalisation


|  | mean | sd | iqr | Q1 | median | Q3 | max | null |
| --- | --- | --- | --- | --- | --- | --- | --- | --- |
| M227.1754T7.46 | 0.0297637 | 0.0125831 | 0.0153123 | 0.0216464 | 0.0248620 | 0.0369588 | 0.0545123 | 0 |
| M227.1756T7.71 | 0.0009423 | 0.0005382 | 0.0005236 | 0.0006028 | 0.0008935 | 0.0011263 | 0.0023538 | 0 |
| M229.1547T7.3 | 0.0002872 | 0.0000654 | 0.0001292 | 0.0002224 | 0.0002810 | 0.0003516 | 0.0003785 | 0 |
| M229.1548T7.51 | 0.0002844 | 0.0000405 | 0.0000395 | 0.0002643 | 0.0002889 | 0.0003038 | 0.0003452 | 0 |
| M231.1704T7.41 | 0.0007549 | 0.0004071 | 0.0004789 | 0.0004242 | 0.0006295 | 0.0009030 | 0.0015536 | 0 |
| M231.1704T7.7 | 0.0007458 | 0.0003289 | 0.0004379 | 0.0004945 | 0.0007700 | 0.0009324 | 0.0013820 | 0 |
| M231.1705T8.71 | 0.0001531 | 0.0000906 | 0.0001410 | 0.0000854 | 0.0001604 | 0.0002264 | 0.0002715 | 0 |
| M232.1544T7.21 | 0.0019758 | 0.0016928 | 0.0009050 | 0.0010000 | 0.0011673 | 0.0019050 | 0.0056565 | 0 |
| M232.1544T7.07 | 0.0010623 | 0.0005972 | 0.0005553 | 0.0006736 | 0.0007847 | 0.0012289 | 0.0021863 | 0 |
| M233.0592T7.35 | 0.0000623 | 0.0000277 | 0.0000356 | 0.0000442 | 0.0000653 | 0.0000798 | 0.0001143 | 0 |

#### Zero filtering and smoothing metabolomics

Metabolites with 0 counts in more than 70% of samples are deemed
unexpressed and are removed from the dataset.

```
message("Filtering undetected metabolites")
undetected_metabolites <- metabolite_stat_prenorm$null >= ncol(metabolomics_all_times_df) * 0.70
print(paste0("undetected_metabolites (null in >= 70% samples): ", sum(undetected_metabolites)))
```

[1] “undetected\_metabolites (null in >= 70% samples): 5”

```
kept_metabolites <- !undetected_metabolites
print(paste0("Kept metabolites: ", sum(kept_metabolites)))
```

[1] “Kept metabolites: 465”

```
## metabolites after filtering
metabolomics_all_times_filtered <- metabolomics_all_times_df[kept_metabolites, ]
```

```
## remaining zeroes smoothing (as in Zhang et al. 2021)

min_val <- apply(metabolomics_all_times_filtered, 1, function(x) (min(x[x>0])/2))


for (metabolite in row.names(metabolomics_all_times_filtered)) {
  metabolomics_all_times_filtered[metabolite,][metabolomics_all_times_filtered[metabolite,] == 0] <- min_val[metabolite]
}
```

#### CLR transformation metabolomics

```
metabolomics_all_times_clr <- metabolomics_all_times_filtered

for (samp in names(metabolomics_all_times_filtered )){
  metabolomics_all_times_clr[,samp] <- as.vector(clr(metabolomics_all_times_filtered[,samp]))
}
```

#### Metabolomics data repartition

```
par(mfrow=c(1,1))
hist(unlist(metabolomics_all_times_df),
     breaks = 200,
     cex.axis = 0.7,
     las = 1,
     col = "skyblue",
     xlab = "raw metabolomics data",
     main = "Distribution of raw metabolomics data")
```

```
par(mfrow=c(1,1))
hist(unlist(metabolomics_all_times_clr),
     breaks = 200,
     cex.axis = 0.7,
     las = 1,
     col = "skyblue",
     xlab = "clr-transformed metabolomics data, all times",
     main = "Distribution of clr-transformed metabolomics data, all times")
```

The distribution of metabolomics data is close to normal.

```
boxplot(metabolomics_all_times_df,
        main = "metabolomics raw data",
        horizontal = TRUE,
        col = metadata_table_metaB_all_times$color,
        cex = 0.5,
        cex.axis = 0.8,
        las = 1)
```

```
boxplot(metabolomics_all_times_clr,
        main = "metabolomics clr-transformed expression",
        horizontal = TRUE,
        col = metadata_table_metaB_all_times$color,
        cex = 0.5,
        cex.axis = 0.8,
        las = 1)
```

The CLR transformation centred our data.

## Exploratory statistical analyses of the whole assemblage

### Principal Component Analyses (PCA) metatranscriptomic

```
par(mfrow = c(2,2))
res_pca <- PCA(t(counts_clr), scale.unit = TRUE, graph = FALSE)
```

```
counts_data <- t(counts_clr)
metadata_tmp <- data.frame(metadata_table, stringsAsFactors = TRUE)
permanova <- adonis2(formula = as.formula(paste0("counts_data~", "condition")), 
                     data = metadata_tmp, method="euclidean", # method="bray", #otu_data~TIME
                     permutations = 999, sqrt.dist = FALSE, add = FALSE, by = "terms")
pv = permanova$`Pr(>F)`[1]
# print(pv)

rm(counts_data, metadata_tmp)
```

```
pca_tmp <- rbind(res_pca$ind$coord,
                 res_pca$ind.sup$coord) %>% data.frame() %>% 
  rownames_to_column("sample")


pca_data <- left_join(pca_tmp, rownames_to_column(metadata_table), by=join_by("sample" == "rowname"))
rm(pca_tmp)

D1_text = paste0("Dim 1 (", 
                 round(data.frame(res_pca$eig)$percentage.of.variance[1], digits=2), 
                 "%)")
D2_text = paste0("Dim 2 (", 
                 round(data.frame(res_pca$eig)$percentage.of.variance[2], digits=2), 
                 "%)")
D3_text = paste0("Dim 3 (", 
                 round(data.frame(res_pca$eig)$percentage.of.variance[3], digits=2), 
                 "%)")

ggplot(pca_data) +
  geom_point(aes(x=Dim.1, y=Dim.2, colour=color, ), size=3) +
  scale_colour_identity("Condition", labels=c("Winter Night", "Summer Day"), guide = "legend") +
  ggrepel::geom_text_repel(aes(x=Dim.1, y=Dim.2, colour=color, label=sample), 
                           nudge_y = -2,  seed = 42, segment.size=0.1, show.legend = FALSE) +
  xlab(D1_text) + 
  ylab(D2_text) +
  ggtitle("PCA metatranscriptomics") +
  labs(caption =  paste0("PERMANOVA on condition: p-value: ", pv)) + 
  theme(panel.background= element_rect(fill="#f4f4f4"),
        plot.caption = element_text(size=7)) -> p1_metaT
 

ggplot(pca_data) +
  geom_point(aes(x=Dim.1, y=Dim.3, colour=color), size=3) +
  scale_colour_identity("Condition", labels=c("Winter Night", "Summer Day"), guide = "legend") +
  ggrepel::geom_text_repel(aes(x=Dim.1, y=Dim.3, colour=color, label=sample), 
                           nudge_y = -2,  seed = 42, segment.size=0.1, show.legend = FALSE) +
  xlab(D1_text) + 
  ylab(D3_text) +
  ggtitle("PCA metatranscriptomics") +
  labs(caption =  paste0("PERMANOVA on condition: p-value: ", pv)) + 
  theme(panel.background= element_rect(fill="#f4f4f4"),
        plot.caption = element_text(size=7)) -> p2

ggplot(pca_data) +
  geom_point(aes(x=Dim.2, y=Dim.3, colour=color), size=3) +
  scale_colour_identity("Condition", labels=c("Winter Night", "Summer Day"), guide = "legend") +
  ggrepel::geom_text_repel(aes(x=Dim.2, y=Dim.3, colour=color, label=sample), 
                           nudge_y = -2,  seed = 42, segment.size=0.1, show.legend = FALSE) +
  xlab(D2_text) + 
  ylab(D3_text) +
  ggtitle("PCA metatranscriptomics") +
  labs(caption =  paste0("PERMANOVA on condition: p-value: ", pv)) + 
  theme(panel.background= element_rect(fill="#f4f4f4"),
        plot.caption = element_text(size=7)) -> p3

plotgrid_PCA <- ggarrange(p1_metaT, p2, p3, labels = c("A", "B", "C"), common.legend=TRUE, legend="bottom", nrow=1) 

print(plotgrid_PCA)
```

```
ggsave(plot = plotgrid_PCA, filename = "../figures/PCA_metatranscriptomics_3_composantes.tiff", dpi = 300, width = 15, height = 5, bg="white")

ggsave(plot=p1_metaT, filename = "../figures/PCA_metatranscriptomics.tiff", dpi = 300, width = 6, height = 5, bg="white")
```

```
row_coord <- res_pca$ind$coord

invisible(rgl::open3d())

#rgl::bg3d("lightgray")
rgl::plot3d(x=row_coord[c("WN_TF_1", "WN_TF_2", "WN_TF_3"), 1], 
            y=row_coord[c("WN_TF_1", "WN_TF_2", "WN_TF_3"), 2], 
            z=row_coord[c("WN_TF_1", "WN_TF_2", "WN_TF_3"), 3],
            xlab = paste0(colnames(row_coord)[1] , " (" , substr(as.character(res_pca$eig[,2][1]), 1, 5) , "%)"),
            ylab = paste0(colnames(row_coord)[2] , " (" , substr(as.character(res_pca$eig[,2][2]), 1, 5) , "%)"),
            zlab = paste0(colnames(row_coord)[3] , " (" , substr(as.character(res_pca$eig[,2][3]), 1, 5) , "%)"),
            xlim = c(min(row_coord[, 1]-10), max(row_coord[, 1])+10), 
            ylim = c(min(row_coord[, 2]-10), max(row_coord[, 2])+10), 
            zlim = c(min(row_coord[, 3]-10), max(row_coord[, 3])+10),
            col = "darkcyan",
            size=10,
            main = "PCA Assemblage")

rgl::text3d(x=row_coord[c("WN_TF_1", "WN_TF_2", "WN_TF_3"), 1]+5, 
            y=row_coord[c("WN_TF_1", "WN_TF_2", "WN_TF_3"), 2]+5, 
            z=row_coord[c("WN_TF_1", "WN_TF_2", "WN_TF_3"), 3]+5,
            text=c("WN_TF_1", "WN_TF_2", "WN_TF_3"),
            col = "darkcyan",
            size=10)


rgl::points3d(x=row_coord[c("SD_TF_1", "SD_TF_2", "SD_TF_3"), 1], 
              y=row_coord[c("SD_TF_1", "SD_TF_2", "SD_TF_3"), 2], 
              z=row_coord[c("SD_TF_1", "SD_TF_2", "SD_TF_3"), 3],
              col = "darkorange",
              size=10)

rgl::text3d(x=row_coord[c("SD_TF_1", "SD_TF_2", "SD_TF_3"), 1]+5, 
            y=row_coord[c("SD_TF_1", "SD_TF_2", "SD_TF_3"), 2]+5, 
            z=row_coord[c("SD_TF_1", "SD_TF_2", "SD_TF_3"), 3]+5,
            text=c("SD_TF_1", "SD_TF_2", "SD_TF_3"),
            col = "darkorange",
            size=10)

# Activate for or gif generation
par3d(windowRect = c(20, 30, 800, 800))

# rgl::movie3d(
#  movie="3dAnimated_PCA_metaT_assemblage",
#  spin3d( axis = c(0, 0, 1), rpm = 3),
#  duration = 20,
#  dir = "../figures",
#  type = "gif",
#  clean = TRUE, webshot=FALSE, fps=20)
```

### PCA metabolomics

```
par(mfrow = c(2,2))
res_pca_metaB_all_times <- PCA(t(metabolomics_all_times_clr), scale.unit = TRUE, graph = FALSE)
```

```
metabolomics_data <- t(metabolomics_all_times_clr)
metabolomics_tmp <- data.frame(metadata_table_metaB_all_times, stringsAsFactors = TRUE)
permanova <- adonis2(formula = as.formula(paste0("metabolomics_data~", "condition")),
                     data = metabolomics_tmp, method="euclidean", 
                     permutations = 999, sqrt.dist = FALSE, add = FALSE, by = "terms")
pv = permanova$`Pr(>F)`[1]

rm(metabolomics_data, metabolomics_tmp)
```

```
pca_tmp_metaB <- rbind(res_pca_metaB_all_times$ind$coord,
                 res_pca_metaB_all_times$ind.sup$coord) %>% data.frame() %>% 
  rownames_to_column("sample")


pca_data_metaB <- left_join(pca_tmp_metaB, rownames_to_column(metadata_table_metaB_all_times), by=join_by("sample" == "rowname"))
rm(pca_tmp_metaB)

D1_text = paste0("Dim 1 (", 
                 round(data.frame(res_pca_metaB_all_times$eig)$percentage.of.variance[1], digits=2), 
                 "%)")
D2_text = paste0("Dim 2 (", 
                 round(data.frame(res_pca_metaB_all_times$eig)$percentage.of.variance[2], digits=2), 
                 "%)")
D3_text = paste0("Dim 3 (", 
                 round(data.frame(res_pca_metaB_all_times$eig)$percentage.of.variance[3], digits=2), 
                 "%)")

ggplot(pca_data_metaB) +
  geom_point(aes(x=Dim.1, y=Dim.2, colour=color, ), size=3) +
  scale_colour_identity("Condition", labels=c("Winter Night", "Summer Day"), guide = "legend") +
  ggrepel::geom_text_repel(aes(x=Dim.1, y=Dim.2, colour=color, label=sample), 
                           nudge_y = -2,  seed = 42, segment.size=0.1, show.legend = FALSE) +
  xlab(D1_text) + 
  ylab(D2_text) +
  ggtitle("PCA meta-metabolomics") +
  labs(caption =  paste0("PERMANOVA on condition: p-value: ", pv)) + 
  theme(panel.background= element_rect(fill="#f4f4f4"),
        plot.caption = element_text(size=7)) -> p1_metaB
 

ggplot(pca_data_metaB) +
  geom_point(aes(x=Dim.1, y=Dim.3, colour=color), size=3) +
  scale_colour_identity("Condition", labels=c("Winter Night", "Summer Day"), guide = "legend") +
  ggrepel::geom_text_repel(aes(x=Dim.1, y=Dim.3, colour=color, label=sample), 
                           nudge_y = -2,  seed = 42, segment.size=0.1, show.legend = FALSE) +
  xlab(D1_text) + 
  ylab(D3_text) +
  ggtitle("PCA meta-metabolomics") +
  labs(caption =  paste0("PERMANOVA on condition: p-value: ", pv)) + 
  theme(panel.background= element_rect(fill="#f4f4f4"),
        plot.caption = element_text(size=7)) -> p2_metaB

ggplot(pca_data_metaB) +
  geom_point(aes(x=Dim.2, y=Dim.3, colour=color), size=3) +
  scale_colour_identity("Condition", labels=c("Winter Night", "Summer Day"), guide = "legend") +
  ggrepel::geom_text_repel(aes(x=Dim.2, y=Dim.3, colour=color, label=sample), 
                           nudge_y = -2,  seed = 42, segment.size=0.1, show.legend = FALSE) +
  xlab(D2_text) + 
  ylab(D3_text) +
  ggtitle("PCA meta-metabolomics") +
  labs(caption =  paste0("PERMANOVA on condition: p-value: ", pv)) + 
  theme(panel.background= element_rect(fill="#f4f4f4"),
        plot.caption = element_text(size=7)) -> p3_metaB

plotgrid_PCA_metaB <- ggarrange(p1_metaB, p2_metaB, p3_metaB, labels = c("A", "B", "C"), common.legend=TRUE, legend="bottom", nrow=1) 

print(plotgrid_PCA_metaB)
```

```
ggsave(plot = p1_metaB, filename = "../figures/PCA_metabolomics_all_times.tiff", dpi = 300, width = 5, height = 5, bg="white")

ggsave(plot = plotgrid_PCA_metaB, filename = "../figures/PCA_metabolomics_3_composantes_all_times.tiff", dpi = 300, width = 15, height = 5, bg="white")

ggsave(plot=p1_metaB, filename = "../figures/PCA_metabolomics_all_times.tiff", dpi = 300, width = 6, height = 5, bg="white")
```

```
row_coord <- res_pca_metaB_all_times$ind$coord

invisible(rgl::open3d())

#rgl::bg3d("lightgray")
rgl::plot3d(x=row_coord[c("WN_TF_1", "WN_TF_2", "WN_TF_3"), 1], 
            y=row_coord[c("WN_TF_1", "WN_TF_2", "WN_TF_3"), 2], 
            z=row_coord[c("WN_TF_1", "WN_TF_2", "WN_TF_3"), 3],
            xlab = paste0(colnames(row_coord)[1] , " (" , substr(as.character(res_pca$eig[,2][1]), 1, 5) , "%)"),
            ylab = paste0(colnames(row_coord)[2] , " (" , substr(as.character(res_pca$eig[,2][2]), 1, 5) , "%)"),
            zlab = paste0(colnames(row_coord)[3] , " (" , substr(as.character(res_pca$eig[,2][3]), 1, 5) , "%)"),
            xlim = c(min(row_coord[, 1]-10), max(row_coord[, 1])+10), 
            ylim = c(min(row_coord[, 2]-10), max(row_coord[, 2])+10), 
            zlim = c(min(row_coord[, 3]-10), max(row_coord[, 3])+10),
            col = "darkcyan",
            size=10,
            main = "PCA meta-metabolomics assemblage")

rgl::text3d(x=row_coord[c("WN_TF_1", "WN_TF_2", "WN_TF_3"), 1]+5, 
            y=row_coord[c("WN_TF_1", "WN_TF_2", "WN_TF_3"), 2]+5, 
            z=row_coord[c("WN_TF_1", "WN_TF_2", "WN_TF_3"), 3]+5,
            text=c("WN_TF_1", "WN_TF_2", "WN_TF_3"),
            col = "darkcyan",
            size=10)


rgl::points3d(x=row_coord[c("SD_TF_1", "SD_TF_2", "SD_TF_3"), 1], 
              y=row_coord[c("SD_TF_1", "SD_TF_2", "SD_TF_3"), 2], 
              z=row_coord[c("SD_TF_1", "SD_TF_2", "SD_TF_3"), 3],
              col = "darkorange",
              size=10)

rgl::text3d(x=row_coord[c("SD_TF_1", "SD_TF_2", "SD_TF_3"), 1]+5, 
            y=row_coord[c("SD_TF_1", "SD_TF_2", "SD_TF_3"), 2]+5, 
            z=row_coord[c("SD_TF_1", "SD_TF_2", "SD_TF_3"), 3]+5,
            text=c("SD_TF_1", "SD_TF_2", "SD_TF_3"),
            col = "darkorange",
            size=10)


rgl::points3d(x=row_coord[c("WN_T0_1", "WN_T0_2", "WN_T0_3"), 1], 
              y=row_coord[c("WN_T0_1", "WN_T0_2", "WN_T0_3"), 2], 
              z=row_coord[c("WN_T0_1", "WN_T0_2", "WN_T0_3"), 3],
              col = "darkcyan",
              size=10)

rgl::text3d(x=row_coord[c("WN_T0_1", "WN_T0_2", "WN_T0_3"), 1]+5, 
            y=row_coord[c("WN_T0_1", "WN_T0_2", "WN_T0_3"), 2]+5, 
            z=row_coord[c("WN_T0_1", "WN_T0_2", "WN_T0_3"), 3]+5,
            text=c("WN_T0_1", "WN_T0_2", "WN_T0_3"),
            col = "darkcyan",
            size=10)


rgl::points3d(x=row_coord[c("SD_T0_1", "SD_T0_2", "SD_T0_3"), 1], 
              y=row_coord[c("SD_T0_1", "SD_T0_2", "SD_T0_3"), 2], 
              z=row_coord[c("SD_T0_1", "SD_T0_2", "SD_T0_3"), 3],
              col = "darkorange",
              size=10)

rgl::text3d(x=row_coord[c("SD_T0_1", "SD_T0_2", "SD_T0_3"), 1]+5, 
            y=row_coord[c("SD_T0_1", "SD_T0_2", "SD_T0_3"), 2]+5, 
            z=row_coord[c("SD_T0_1", "SD_T0_2", "SD_T0_3"), 3]+5,
            text=c("SD_T0_1", "SD_T0_2", "SD_T0_3"),
            col = "darkorange",
            size=10)


# Activate for or gif generation
# par3d(windowRect = c(20, 30, 800, 800))
# 
# rgl::movie3d(
#  movie="3dAnimated_PCA_metaB_assemblage",
#  spin3d( axis = c(0, 0, 1), rpm = 3),
#  duration = 20,
#  dir = "../figures",
#  type = "gif",
#  clean = TRUE, webshot=FALSE, fps=20)
```

```
plot_all <- ggarrange(p1_metaB, p1_metaT, 
                      labels = c("A", "B"), 
                      common.legend=TRUE,
                      legend="bottom",
                      nrow=1,
                      widths = c(1, 1))

ggsave(plot = plot_all, filename = "../figures/Figure_2.tiff", dpi = 300, width = 11, height = 6, bg="white")
```

## Individual exploratory analysis of metatranscriptomics data by species

#### Data preparation

#### Load data by species

```
diohu_geneid <- annotation_table$Organism == "D.hungarica"
diohu_df <- counts_standard[startsWith(rownames_to_column(counts_standard)$rowname, "D.hungarica"),]

psesy_geneid <- annotation_table$Organism == "P.syringae"
psesy_df <- counts_standard[startsWith(rownames_to_column(counts_standard)$rowname, "P.syringae"),]

psegr_geneid <- annotation_table$Organism == "P.graminis"
psegr_df <- counts_standard[startsWith(rownames_to_column(counts_standard)$rowname, "P.graminis"),]

rhoen_geneid <- annotation_table$Organism == "R.enclensis"
rhoen_df <- counts_standard[startsWith(rownames_to_column(counts_standard)$rowname, "R.enclensis"),]
```

#### Zeroes smoothing by species

```
## remaining zeroes smoothing (as in Zhang et al. 2021)
min_val <- apply(diohu_df, 1, function(x) (min(x[x>0])/2))
for (gene in row.names(diohu_df)) {
  diohu_df[gene,][diohu_df[gene,] == 0] <- min_val[gene]
}

min_val <- apply(psesy_df, 1, function(x) (min(x[x>0])/2))
for (gene in row.names(psesy_df)) {
  psesy_df[gene,][psesy_df[gene,] == 0] <- min_val[gene]
}

min_val <- apply(psegr_df, 1, function(x) (min(x[x>0])/2))
for (gene in row.names(psegr_df)) {
  psegr_df[gene,][psegr_df[gene,] == 0] <- min_val[gene]
}

min_val <- apply(rhoen_df, 1, function(x) (min(x[x>0])/2))
for (gene in row.names(rhoen_df)) {
  rhoen_df[gene,][rhoen_df[gene,] == 0] <- min_val[gene]
}
```

#### CLR Transformation of counts by species

```
diohu_clr <- diohu_df
psesy_clr <- psesy_df
psegr_clr <- psegr_df
rhoen_clr <- rhoen_df

for (samp in samples) {
    diohu_clr[,samp] <- as.vector(clr(diohu_df[,samp]))
    psesy_clr[,samp] <- as.vector(clr(psesy_df[,samp]))
    psegr_clr[,samp] <- as.vector(clr(psegr_df[,samp]))
    rhoen_clr[,samp] <- as.vector(clr(rhoen_df[,samp]))
}
```

### Transcriptomics counts histograms by species

#### *Dioszegia hungarica*

```
par(mfrow=c(1,1))
hist(unlist(diohu_clr),
     breaks = 200,
     cex.axis = 0.7,
     las = 1,
     col = "skyblue",
     ylim = c(0,1800),
     xlim = c(-5,15),
     xlab = "clr-transformed counts",
     main = "Distribution of D. hungarica clr-transformed counts")
```

#### *Pseudomonas graminis*

```
par(mfrow=c(1,1))
hist(unlist(psegr_clr),
     breaks = 200,
     cex.axis = 0.7,
     las = 1,
     col = "skyblue",
     ylim = c(0,1800),
     xlim = c(-5,15),
     xlab = "clr-transformed counts",
     main = "Distribution of P. graminis clr-transformed counts")
```

Very few *P. graminis* counts, probably not exploitable.

#### *Pseudomonas syringae*

```
par(mfrow=c(1,1))
hist(unlist(psesy_clr),
     breaks = 200,
     cex.axis = 0.7,
     las = 1,
     col = "skyblue",
     ylim = c(0,1800),
     xlim = c(-5,15),
     xlab = "clr-transformed counts",
     main = "Distribution of P. syringae clr-transformed counts")
```

#### *Rhodococcus enclensis*

```
par(mfrow=c(1,1))
hist(unlist(rhoen_clr),
     breaks = 200,
     cex.axis = 0.7,
     las = 1,
     col = "skyblue",
     ylim = c(0,1800),
     xlim = c(-5,15),
     xlab = "clr-transformed counts",
     main = "Distribution of R. enclensis clr-transformed counts")
```

### Transcriptomics counts box-plots by species

#### *Dioszegia hungarica*

```
boxplot(diohu_clr,
        main = "D. hungarica clr-transformed expression",
        horizontal = TRUE,
        col = metadata_table$color,
        cex = 0.5,
        cex.axis = 0.8,
        las = 1)
```

#### *Pseudomonas graminis*

```
boxplot(psegr_clr,
        main = "P. graminis clr-transformed expression",
        horizontal = TRUE,
        col = metadata_table$color,
        cex = 0.5,
        cex.axis = 0.8,
        las = 1)
```

#### *Pseudomonas syringae*

```
boxplot(psesy_clr,
        main = "P. syringae clr-transformed expression",
        horizontal = TRUE,
        col = metadata_table$color,
        cex = 0.5,
        cex.axis = 0.8,
        las = 1)
```

#### *Rhodococcus enclensis*

```
boxplot(rhoen_clr,
        main = "R. enclensis clr-transformed expression",
        horizontal = TRUE,
        col = metadata_table$color,
        cex = 0.5,
        cex.axis = 0.8,
        las = 1)
```

### Transcriptomics counts PCA by species

#### *Dioszegia hungarica*

```
res_pca <- PCA(t(diohu_clr), scale.unit = TRUE, graph = FALSE)
```

```
counts_data <- t(diohu_clr)
metadata_tmp <- data.frame(metadata_table, stringsAsFactors = TRUE)
permanova <- adonis2(formula = as.formula(paste0("counts_data~", "condition")), 
                     data = metadata_tmp, method="euclidean", # method="bray", #otu_data~TIME
                     permutations = 999, sqrt.dist = FALSE, add = FALSE, by = "terms")
pv = permanova$`Pr(>F)`[1]

rm(counts_data, metadata_tmp)
```

```
pca_tmp <- rbind(res_pca$ind$coord,
                 res_pca$ind.sup$coord) %>% data.frame() %>% 
  rownames_to_column("sample")


pca_data <- left_join(pca_tmp, rownames_to_column(metadata_table), by=join_by("sample" == "rowname"))
rm(pca_tmp)

D1_text = paste0("Dim 1 (", 
                 round(data.frame(res_pca$eig)$percentage.of.variance[1], digits=2), 
                 "%)")
D2_text = paste0("Dim 2 (", 
                 round(data.frame(res_pca$eig)$percentage.of.variance[2], digits=2), 
                 "%)")
D3_text = paste0("Dim 3 (", 
                 round(data.frame(res_pca$eig)$percentage.of.variance[3], digits=2), 
                 "%)")

ggplot(pca_data) +
  geom_point(aes(x=Dim.1, y=Dim.2, colour=color, ), size=3) +
  scale_colour_identity("Condition", labels=c("Winter Night", "Summer Day"), guide = "legend") +
  ggrepel::geom_text_repel(aes(x=Dim.1, y=Dim.2, colour=color, label=sample), 
                           nudge_y = -2,  seed = 42, segment.size=0.1, show.legend = FALSE) +
  xlab(D1_text) + 
  ylab(D2_text) +
  ggtitle("PCA metatranscriptomics  \nD. hungarica") +
  labs(caption =  paste0("PERMANOVA on condition: p-value: ", pv)) + 
  theme(panel.background= element_rect(fill="#f4f4f4"),
        plot.caption = element_text(size=7)) -> p1
 

ggplot(pca_data) +
  geom_point(aes(x=Dim.1, y=Dim.3, colour=color), size=3) +
  scale_colour_identity("Condition", labels=c("Winter Night", "Summer Day"), guide = "legend") +
  ggrepel::geom_text_repel(aes(x=Dim.1, y=Dim.3, colour=color, label=sample), 
                           nudge_y = -2,  seed = 42, segment.size=0.1, show.legend = FALSE) +
  xlab(D1_text) + 
  ylab(D3_text) +
  ggtitle("PCA metatranscriptomics  \nD. hungarica") +
  labs(caption =  paste0("PERMANOVA on condition: p-value: ", pv)) + 
  theme(panel.background= element_rect(fill="#f4f4f4"),
        plot.caption = element_text(size=7)) -> p2

ggplot(pca_data) +
  geom_point(aes(x=Dim.2, y=Dim.3, colour=color), size=3) +
  scale_colour_identity("Condition", labels=c("Winter Night", "Summer Day"), guide = "legend") +
  ggrepel::geom_text_repel(aes(x=Dim.2, y=Dim.3, colour=color, label=sample), 
                           nudge_y = -2,  seed = 42, segment.size=0.1, show.legend = FALSE) +
  xlab(D2_text) + 
  ylab(D3_text) +
  ggtitle("PCA metatranscriptomics  \nD. hungarica") +
  labs(caption =  paste0("PERMANOVA on condition: p-value: ", pv)) + 
  theme(panel.background= element_rect(fill="#f4f4f4"),
        plot.caption = element_text(size=7)) -> p3

plotgrid_PCA <- ggarrange(p1, p2, p3, labels = c("A", "B", "C"), common.legend=TRUE, legend="bottom", nrow=1) 

print(plotgrid_PCA)
```

```
ggsave(plot = plotgrid_PCA, filename = "../figures/PCA_metatranscriptomics_3_composantes_diohu.tiff", dpi = 300, width = 15, height = 5, bg="white")

ggsave(plot=p1, filename = "../figures/PCA_metatranscriptomics_diohu.tiff", dpi = 300, width = 6, height = 5, bg="white")
```

```
row_coord <- res_pca$ind$coord


invisible(rgl::open3d())

#rgl::bg3d("lightgray")
rgl::plot3d(x=row_coord[c("WN_TF_1", "WN_TF_2", "WN_TF_3"), 1], 
            y=row_coord[c("WN_TF_1", "WN_TF_2", "WN_TF_3"), 2], 
            z=row_coord[c("WN_TF_1", "WN_TF_2", "WN_TF_3"), 3],
            xlab = paste0(colnames(row_coord)[1] , " (" , substr(as.character(res_pca$eig[,2][1]), 1, 5) , "%)"),
            ylab = paste0(colnames(row_coord)[2] , " (" , substr(as.character(res_pca$eig[,2][2]), 1, 5) , "%)"),
            zlab = paste0(colnames(row_coord)[3] , " (" , substr(as.character(res_pca$eig[,2][3]), 1, 5) , "%)"),
            xlim = c(min(row_coord[, 1]), max(row_coord[, 1])+10), 
            ylim = c(min(row_coord[, 2]), max(row_coord[, 2])+10), 
            zlim = c(min(row_coord[, 3]), max(row_coord[, 3])+10),
            col = "darkcyan",
            size=10,
            main = "PCA D. hungarica")

rgl::text3d(x=row_coord[c("WN_TF_1", "WN_TF_2", "WN_TF_3"), 1]+5, 
            y=row_coord[c("WN_TF_1", "WN_TF_2", "WN_TF_3"), 2]+5, 
            z=row_coord[c("WN_TF_1", "WN_TF_2", "WN_TF_3"), 3]+5,
            text=c("WN_TF_1", "WN_TF_2", "WN_TF_3"),
            col = "darkcyan",
            size=10)


rgl::points3d(x=row_coord[c("SD_TF_1", "SD_TF_2", "SD_TF_3"), 1], 
              y=row_coord[c("SD_TF_1", "SD_TF_2", "SD_TF_3"), 2], 
              z=row_coord[c("SD_TF_1", "SD_TF_2", "SD_TF_3"), 3],
              col = "darkorange",
              size=10)

rgl::text3d(x=row_coord[c("SD_TF_1", "SD_TF_2", "SD_TF_3"), 1]+5, 
            y=row_coord[c("SD_TF_1", "SD_TF_2", "SD_TF_3"), 2]+5, 
            z=row_coord[c("SD_TF_1", "SD_TF_2", "SD_TF_3"), 3]+5,
            text=c("SD_TF_1", "SD_TF_2", "SD_TF_3"),
            col = "darkorange",
            size=10)

## Activate for or gif generation
#par3d(windowRect = c(20, 30, 800, 800))

#rgl::movie3d(
#  movie="3DAnimated_PCA_diohu", 
#  spin3d( axis = c(0, 0, 1), rpm = 3),
#  duration = 20, 
#  dir = "../figures",
#  type = "gif", 
#  clean = TRUE, webshot=FALSE, fps=10)
```

#### *Pseudomonas graminis*

```
res_pca <- PCA(t(psegr_clr), scale.unit = TRUE, graph = FALSE)
```

```
counts_data <- t(psegr_clr)
metadata_tmp <- data.frame(metadata_table, stringsAsFactors = TRUE)
permanova <- adonis2(formula = as.formula(paste0("counts_data~", "condition")), 
                     data = metadata_tmp, method="euclidean", # method="bray", #otu_data~TIME
                     permutations = 999, sqrt.dist = FALSE, add = FALSE, by = "terms")
pv = permanova$`Pr(>F)`[1]
# print(pv)

rm(counts_data, metadata_tmp)
```

```
pca_tmp <- rbind(res_pca$ind$coord,
                 res_pca$ind.sup$coord) %>% data.frame() %>% 
  rownames_to_column("sample")


pca_data <- left_join(pca_tmp, rownames_to_column(metadata_table), by=join_by("sample" == "rowname"))
rm(pca_tmp)

D1_text = paste0("Dim 1 (", 
                 round(data.frame(res_pca$eig)$percentage.of.variance[1], digits=2), 
                 "%)")
D2_text = paste0("Dim 2 (", 
                 round(data.frame(res_pca$eig)$percentage.of.variance[2], digits=2), 
                 "%)")
D3_text = paste0("Dim 3 (", 
                 round(data.frame(res_pca$eig)$percentage.of.variance[3], digits=2), 
                 "%)")

ggplot(pca_data) +
  geom_point(aes(x=Dim.1, y=Dim.2, colour=color, ), size=3) +
  scale_colour_identity("Condition", labels=c("Winter Night", "Summer Day"), guide = "legend") +
  ggrepel::geom_text_repel(aes(x=Dim.1, y=Dim.2, colour=color, label=sample), 
                           nudge_y = -2,  seed = 42, segment.size=0.1, show.legend = FALSE) +
  xlab(D1_text) + 
  ylab(D2_text) +
  ggtitle("PCA metatranscriptomics  \nP. graminis") +
  labs(caption =  paste0("PERMANOVA on condition: p-value: ", pv)) + 
  theme(panel.background= element_rect(fill="#f4f4f4"),
        plot.caption = element_text(size=7)) -> p1
 

ggplot(pca_data) +
  geom_point(aes(x=Dim.1, y=Dim.3, colour=color), size=3) +
  scale_colour_identity("Condition", labels=c("Winter Night", "Summer Day"), guide = "legend") +
  ggrepel::geom_text_repel(aes(x=Dim.1, y=Dim.3, colour=color, label=sample), 
                           nudge_y = -2,  seed = 42, segment.size=0.1, show.legend = FALSE) +
  xlab(D1_text) + 
  ylab(D3_text) +
  ggtitle("PCA metatranscriptomics  \nP. graminis") +
  labs(caption =  paste0("PERMANOVA on condition: p-value: ", pv)) + 
  theme(panel.background= element_rect(fill="#f4f4f4"),
        plot.caption = element_text(size=7)) -> p2

ggplot(pca_data) +
  geom_point(aes(x=Dim.2, y=Dim.3, colour=color), size=3) +
  scale_colour_identity("Condition", labels=c("Winter Night", "Summer Day"), guide = "legend") +
  ggrepel::geom_text_repel(aes(x=Dim.2, y=Dim.3, colour=color, label=sample), 
                           nudge_y = -2,  seed = 42, segment.size=0.1, show.legend = FALSE) +
  xlab(D2_text) + 
  ylab(D3_text) +
  ggtitle("PCA metatranscriptomics  \nP. graminis") +
  labs(caption =  paste0("PERMANOVA on condition: p-value: ", pv)) + 
  theme(panel.background= element_rect(fill="#f4f4f4"),
        plot.caption = element_text(size=7)) -> p3

plotgrid_PCA <- ggarrange(p1, p2, p3, labels = c("A", "B", "C"), common.legend=TRUE, legend="bottom", nrow=1) 

print(plotgrid_PCA)
```

```
ggsave(plot = plotgrid_PCA, filename = "../figures/PCA_metatranscriptomics_3_composantes_psegr.tiff", dpi = 300, width = 15, height = 5, bg="white")

ggsave(plot=p1, filename = "../figures/PCA_metatranscriptomics_psegr.tiff", dpi = 300, width = 6, height = 5, bg="white")
```

```
row_coord <- res_pca$ind$coord

invisible(rgl::open3d())

#rgl::bg3d("lightgray")
rgl::plot3d(x=row_coord[c("WN_TF_1", "WN_TF_2", "WN_TF_3"), 1], 
            y=row_coord[c("WN_TF_1", "WN_TF_2", "WN_TF_3"), 2], 
            z=row_coord[c("WN_TF_1", "WN_TF_2", "WN_TF_3"), 3],
            xlab = paste0(colnames(row_coord)[1] , " (" , substr(as.character(res_pca$eig[,2][1]), 1, 5) , "%)"),
            ylab = paste0(colnames(row_coord)[2] , " (" , substr(as.character(res_pca$eig[,2][2]), 1, 5) , "%)"),
            zlab = paste0(colnames(row_coord)[3] , " (" , substr(as.character(res_pca$eig[,2][3]), 1, 5) , "%)"),
            xlim = c(min(row_coord[, 1]), max(row_coord[, 1])+10), 
            ylim = c(min(row_coord[, 2]), max(row_coord[, 2])+10), 
            zlim = c(min(row_coord[, 3]), max(row_coord[, 3])+10),
            col = "darkcyan",
            size=10,
            main = "PCA P. graminis")

rgl::text3d(x=row_coord[c("WN_TF_1", "WN_TF_2", "WN_TF_3"), 1]+5, 
            y=row_coord[c("WN_TF_1", "WN_TF_2", "WN_TF_3"), 2]+5, 
            z=row_coord[c("WN_TF_1", "WN_TF_2", "WN_TF_3"), 3]+5,
            text=c("WN_TF_1", "WN_TF_2", "WN_TF_3"),
            col = "darkcyan",
            size=10)


rgl::points3d(x=row_coord[c("SD_TF_1", "SD_TF_2", "SD_TF_3"), 1], 
              y=row_coord[c("SD_TF_1", "SD_TF_2", "SD_TF_3"), 2], 
              z=row_coord[c("SD_TF_1", "SD_TF_2", "SD_TF_3"), 3],
              col = "darkorange",
              size=10)

rgl::text3d(x=row_coord[c("SD_TF_1", "SD_TF_2", "SD_TF_3"), 1]+3, 
            y=row_coord[c("SD_TF_1", "SD_TF_2", "SD_TF_3"), 2]+3, 
            z=row_coord[c("SD_TF_1", "SD_TF_2", "SD_TF_3"), 3]+3,
            text=c("SD_TF_1", "SD_TF_2", "SD_TF_3"),
            col = "darkorange",
            size=10)

## Activate for or gif generation
#par3d(windowRect = c(20, 30, 800, 800))

#rgl::movie3d(
#  movie="3DAnimated_PCA_psegr", 
#  spin3d( axis = c(0, 0, 1), rpm = 3),
#  duration = 20, 
#  dir = "../figures",
#  type = "gif", 
#  clean = TRUE, webshot=FALSE, fps=10)
```

#### *Pseudomonas syringae*

```
res_pca <- PCA(t(psesy_clr), scale.unit = TRUE, graph = FALSE)
```

```
counts_data <- t(psesy_clr)
metadata_tmp <- data.frame(metadata_table, stringsAsFactors = TRUE)
permanova <- adonis2(formula = as.formula(paste0("counts_data~", "condition")), 
                     data = metadata_tmp, method="euclidean", # method="bray", #otu_data~TIME
                     permutations = 999, sqrt.dist = FALSE, add = FALSE, by = "terms")
pv = permanova$`Pr(>F)`[1]
# print(pv)

rm(counts_data, metadata_tmp)
```

```
pca_tmp <- rbind(res_pca$ind$coord,
                 res_pca$ind.sup$coord) %>% data.frame() %>% 
  rownames_to_column("sample")


pca_data <- left_join(pca_tmp, rownames_to_column(metadata_table), by=join_by("sample" == "rowname"))
rm(pca_tmp)

D1_text = paste0("Dim 1 (", 
                 round(data.frame(res_pca$eig)$percentage.of.variance[1], digits=2), 
                 "%)")
D2_text = paste0("Dim 2 (", 
                 round(data.frame(res_pca$eig)$percentage.of.variance[2], digits=2), 
                 "%)")
D3_text = paste0("Dim 3 (", 
                 round(data.frame(res_pca$eig)$percentage.of.variance[3], digits=2), 
                 "%)")

ggplot(pca_data) +
  geom_point(aes(x=Dim.1, y=Dim.2, colour=color, ), size=3) +
  scale_colour_identity("Condition", labels=c("Winter Night", "Summer Day"), guide = "legend") +
  ggrepel::geom_text_repel(aes(x=Dim.1, y=Dim.2, colour=color, label=sample), 
                           nudge_y = -2,  seed = 42, segment.size=0.1, show.legend = FALSE) +
  xlab(D1_text) + 
  ylab(D2_text) +
  ggtitle("PCA metatranscriptomics  \nP. syringae") +
  labs(caption =  paste0("PERMANOVA on condition: p-value: ", pv)) + 
  theme(panel.background= element_rect(fill="#f4f4f4"),
        plot.caption = element_text(size=7)) -> p1
 

ggplot(pca_data) +
  geom_point(aes(x=Dim.1, y=Dim.3, colour=color), size=3) +
  scale_colour_identity("Condition", labels=c("Winter Night", "Summer Day"), guide = "legend") +
  ggrepel::geom_text_repel(aes(x=Dim.1, y=Dim.3, colour=color, label=sample), 
                           nudge_y = -2,  seed = 42, segment.size=0.1, show.legend = FALSE) +
  xlab(D1_text) + 
  ylab(D3_text) +
  ggtitle("PCA metatranscriptomics  \nP. syringae") +
  labs(caption =  paste0("PERMANOVA on condition: p-value: ", pv)) + 
  theme(panel.background= element_rect(fill="#f4f4f4"),
        plot.caption = element_text(size=7)) -> p2

ggplot(pca_data) +
  geom_point(aes(x=Dim.2, y=Dim.3, colour=color), size=3) +
  scale_colour_identity("Condition", labels=c("Winter Night", "Summer Day"), guide = "legend") +
  ggrepel::geom_text_repel(aes(x=Dim.2, y=Dim.3, colour=color, label=sample), 
                           nudge_y = -2,  seed = 42, segment.size=0.1, show.legend = FALSE) +
  xlab(D2_text) + 
  ylab(D3_text) +
  ggtitle("PCA metatranscriptomics  \nP. syringae") +
  labs(caption =  paste0("PERMANOVA on condition: p-value: ", pv)) + 
  theme(panel.background= element_rect(fill="#f4f4f4"),
        plot.caption = element_text(size=7)) -> p3

plotgrid_PCA <- ggarrange(p1, p2, p3, labels = c("A", "B", "C"), common.legend=TRUE, legend="bottom", nrow=1) 

print(plotgrid_PCA)
```

```
ggsave(plot = plotgrid_PCA, filename = "../figures/PCA_metatranscriptomics_3_composantes_psesy.tiff", dpi = 300, width = 15, height = 5, bg="white")

ggsave(plot=p1, filename = "../figures/PCA_metatranscriptomics_psesy.tiff", dpi = 300, width = 6, height = 5, bg="white")
```

```
row_coord <- res_pca$ind$coord

invisible(rgl::open3d())

#rgl::bg3d("lightgray")
rgl::plot3d(x=row_coord[c("WN_TF_1", "WN_TF_2", "WN_TF_3"), 1], 
            y=row_coord[c("WN_TF_1", "WN_TF_2", "WN_TF_3"), 2], 
            z=row_coord[c("WN_TF_1", "WN_TF_2", "WN_TF_3"), 3],
            xlab = paste0(colnames(row_coord)[1] , " (" , substr(as.character(res_pca$eig[,2][1]), 1, 5) , "%)"),
            ylab = paste0(colnames(row_coord)[2] , " (" , substr(as.character(res_pca$eig[,2][2]), 1, 5) , "%)"),
            zlab = paste0(colnames(row_coord)[3] , " (" , substr(as.character(res_pca$eig[,2][3]), 1, 5) , "%)"),
            xlim = c(min(row_coord[, 1]), max(row_coord[, 1])+10), 
            ylim = c(min(row_coord[, 2]), max(row_coord[, 2])+10), 
            zlim = c(min(row_coord[, 3]), max(row_coord[, 3])+10),
            col = "darkcyan",
            size=10,
            main = "PCA P. syringae")

rgl::text3d(x=row_coord[c("WN_TF_1", "WN_TF_2", "WN_TF_3"), 1]+3, 
            y=row_coord[c("WN_TF_1", "WN_TF_2", "WN_TF_3"), 2]+3, 
            z=row_coord[c("WN_TF_1", "WN_TF_2", "WN_TF_3"), 3]+3,
            text=c("WN_TF_1", "WN_TF_2", "WN_TF_3"),
            col = "darkcyan",
            size=10)


rgl::points3d(x=row_coord[c("SD_TF_1", "SD_TF_2", "SD_TF_3"), 1], 
              y=row_coord[c("SD_TF_1", "SD_TF_2", "SD_TF_3"), 2], 
              z=row_coord[c("SD_TF_1", "SD_TF_2", "SD_TF_3"), 3],
              col = "darkorange",
              size=10)

rgl::text3d(x=row_coord[c("SD_TF_1", "SD_TF_2", "SD_TF_3"), 1]+3, 
            y=row_coord[c("SD_TF_1", "SD_TF_2", "SD_TF_3"), 2]+3, 
            z=row_coord[c("SD_TF_1", "SD_TF_2", "SD_TF_3"), 3]+3,
            text=c("SD_TF_1", "SD_TF_2", "SD_TF_3"),
            col = "darkorange",
            size=10)


## Activate for or gif generation
#par3d(windowRect = c(20, 30, 800, 800))

#rgl::movie3d(
#  movie="3DAnimated_PCA_psesy", 
#  spin3d( axis = c(0, 0, 1), rpm = 3),
#  duration = 20, 
#  dir = "../figures",
#  type = "gif", 
#  clean = TRUE, webshot=FALSE, fps=10)
```

#### *Rhodococcus enclensis*

```
res_pca <- PCA(t(rhoen_clr), scale.unit = TRUE, graph = FALSE)
```

```
counts_data <- t(rhoen_clr)
metadata_tmp <- data.frame(metadata_table, stringsAsFactors = TRUE)
permanova <- adonis2(formula = as.formula(paste0("counts_data~", "condition")), 
                     data = metadata_tmp, method="euclidean", # method="bray", #otu_data~TIME
                     permutations = 999, sqrt.dist = FALSE, add = FALSE, by = "terms")
pv = permanova$`Pr(>F)`[1]
# print(pv)

rm(counts_data, metadata_tmp)
```

```
pca_tmp <- rbind(res_pca$ind$coord,
                 res_pca$ind.sup$coord) %>% data.frame() %>% 
  rownames_to_column("sample")


pca_data <- left_join(pca_tmp, rownames_to_column(metadata_table), by=join_by("sample" == "rowname"))
rm(pca_tmp)

D1_text = paste0("Dim 1 (", 
                 round(data.frame(res_pca$eig)$percentage.of.variance[1], digits=2), 
                 "%)")
D2_text = paste0("Dim 2 (", 
                 round(data.frame(res_pca$eig)$percentage.of.variance[2], digits=2), 
                 "%)")
D3_text = paste0("Dim 3 (", 
                 round(data.frame(res_pca$eig)$percentage.of.variance[3], digits=2), 
                 "%)")

ggplot(pca_data) +
  geom_point(aes(x=Dim.1, y=Dim.2, colour=color, ), size=3) +
  scale_colour_identity("Condition", labels=c("Winter Night", "Summer Day"), guide = "legend") +
  ggrepel::geom_text_repel(aes(x=Dim.1, y=Dim.2, colour=color, label=sample), 
                           nudge_y = -2,  seed = 42, segment.size=0.1, show.legend = FALSE) +
  xlab(D1_text) + 
  ylab(D2_text) +
  ggtitle("PCA metatranscriptomics  \nR. enclensis") +
  labs(caption =  paste0("PERMANOVA on condition: p-value: ", pv)) + 
  theme(panel.background= element_rect(fill="#f4f4f4"),
        plot.caption = element_text(size=7)) -> p1
 

ggplot(pca_data) +
  geom_point(aes(x=Dim.1, y=Dim.3, colour=color), size=3) +
  scale_colour_identity("Condition", labels=c("Winter Night", "Summer Day"), guide = "legend") +
  ggrepel::geom_text_repel(aes(x=Dim.1, y=Dim.3, colour=color, label=sample), 
                           nudge_y = -2,  seed = 42, segment.size=0.1, show.legend = FALSE) +
  xlab(D1_text) + 
  ylab(D3_text) +
  ggtitle("PCA metatranscriptomics  \nR. enclensis") +
  labs(caption =  paste0("PERMANOVA on condition: p-value: ", pv)) + 
  theme(panel.background= element_rect(fill="#f4f4f4"),
        plot.caption = element_text(size=7)) -> p2

ggplot(pca_data) +
  geom_point(aes(x=Dim.2, y=Dim.3, colour=color), size=3) +
  scale_colour_identity("Condition", labels=c("Winter Night", "Summer Day"), guide = "legend") +
  ggrepel::geom_text_repel(aes(x=Dim.2, y=Dim.3, colour=color, label=sample), 
                           nudge_y = -2,  seed = 42, segment.size=0.1, show.legend = FALSE) +
  xlab(D2_text) + 
  ylab(D3_text) +
  ggtitle("PCA metatranscriptomics  \nR. enclensis") +
  labs(caption =  paste0("PERMANOVA on condition: p-value: ", pv)) + 
  theme(panel.background= element_rect(fill="#f4f4f4"),
        plot.caption = element_text(size=7)) -> p3

plotgrid_PCA <- ggarrange(p1, p2, p3, labels = c("A", "B", "C"), common.legend=TRUE, legend="bottom", nrow=1) 

print(plotgrid_PCA)
```

```
ggsave(plot = plotgrid_PCA, filename = "../figures/PCA_metatranscriptomics_3_composantes_rhoen.tiff", dpi = 300, width = 15, height = 5, bg="white")

ggsave(plot=p1, filename = "../figures/PCA_metatranscriptomics_rhoen.tiff", dpi = 300, width = 6, height = 5, bg="white")
```

```
row_coord <- res_pca$ind$coord

invisible(rgl::open3d())

#rgl::bg3d("lightgray")
rgl::plot3d(x=row_coord[c("WN_TF_1", "WN_TF_2", "WN_TF_3"), 1], 
            y=row_coord[c("WN_TF_1", "WN_TF_2", "WN_TF_3"), 2], 
            z=row_coord[c("WN_TF_1", "WN_TF_2", "WN_TF_3"), 3],
            xlab = paste0(colnames(row_coord)[1] , " (" , substr(as.character(res_pca$eig[,2][1]), 1, 5) , "%)"),
            ylab = paste0(colnames(row_coord)[2] , " (" , substr(as.character(res_pca$eig[,2][2]), 1, 5) , "%)"),
            zlab = paste0(colnames(row_coord)[3] , " (" , substr(as.character(res_pca$eig[,2][3]), 1, 5) , "%)"),
            xlim = c(min(row_coord[, 1]), max(row_coord[, 1])+10), 
            ylim = c(min(row_coord[, 2]), max(row_coord[, 2])+10), 
            zlim = c(min(row_coord[, 3]), max(row_coord[, 3])+10),
            col = "darkcyan",
            size=10,
            main = "PCA R. enclensis")

rgl::text3d(x=row_coord[c("WN_TF_1", "WN_TF_2", "WN_TF_3"), 1]+3, 
            y=row_coord[c("WN_TF_1", "WN_TF_2", "WN_TF_3"), 2]+3, 
            z=row_coord[c("WN_TF_1", "WN_TF_2", "WN_TF_3"), 3]+3,
            text=c("WN_TF_1", "WN_TF_2", "WN_TF_3"),
            col = "darkcyan",
            size=10)


rgl::points3d(x=row_coord[c("SD_TF_1", "SD_TF_2", "SD_TF_3"), 1], 
              y=row_coord[c("SD_TF_1", "SD_TF_2", "SD_TF_3"), 2], 
              z=row_coord[c("SD_TF_1", "SD_TF_2", "SD_TF_3"), 3],
              col = "darkorange",
              size=10)

rgl::text3d(x=row_coord[c("SD_TF_1", "SD_TF_2", "SD_TF_3"), 1]+3, 
            y=row_coord[c("SD_TF_1", "SD_TF_2", "SD_TF_3"), 2]+3, 
            z=row_coord[c("SD_TF_1", "SD_TF_2", "SD_TF_3"), 3]+3,
            text=c("SD_TF_1", "SD_TF_2", "SD_TF_3"),
            col = "darkorange",
            size=10)


## Activate for or gif generation
#par3d(windowRect = c(20, 30, 800, 800))

#rgl::movie3d(
#  movie="3DAnimated_PCA_rhoen", 
#  spin3d( axis = c(0, 0, 1), rpm = 3),
#  duration = 20, 
#  dir = "../figures",
#  type = "gif", 
#  clean = TRUE, webshot=FALSE, fps=10)
```

### Samples hierarchical clustering by species

#### *Dioszegia hungarica*

```
#### Sample distances ####
message("Computing inter-sample distances")

## Pearson
dist_pearson_diohu <- as.dist(1 - cor(diohu_clr, use = "everything", 
                                method = "pearson"))
```

```
message("Sample clustering")

tree_pearson_diohu <- hclust(dist_pearson_diohu, method = "complete")
```

```
par(bg = "white", mfrow=c(1, 1))
plotColoredClusters(tree_pearson_diohu, labs = row.names(metadata_table),
                    ylab = NA, xlab = NA, cex = 1, las = 1,
                    cols = metadata_table$color, col = "black",
                    main = "Samples Pearson distance hierarchical clustering,
complete linkage, D. hungarica genes.")
```

Natural separation of samples according to their incubation condition
for *D. hungarica*.

#### *Pseudomonas graminis*

```
#### Sample distances ####
message("Computing inter-sample distances")

## Pearson
dist_pearson_psegr <- as.dist(1 - cor(psegr_clr, use = "everything", 
                                method = "pearson"))
```

```
message("Sample clustering")

tree_pearson_psegr <- hclust(dist_pearson_psegr, method = "complete")
```

```
par(bg = "white", mfrow=c(1, 1))
plotColoredClusters(tree_pearson_psegr, labs = row.names(metadata_table),
                    ylab = NA, xlab = NA, cex = 1, las = 1,
                    cols = metadata_table$color, col = "black",
                    main = "Samples Pearson distance hierarchical clustering,
complete linkage, P. graminis genes.")
```

For *P. graminis*, the clustering does not distinguish between
the two incubation conditions, probably because of the very low amounts
of counts attributed to this species.

#### *Pseudomonas syringae*

```
#### Sample distances ####
message("Computing inter-sample distances")

## Pearson
dist_pearson_psesy <- as.dist(1 - cor(psesy_clr, use = "everything", 
                                method = "pearson"))
```

```
message("Sample clustering")

tree_pearson_psesy <- hclust(dist_pearson_psesy, method = "complete")
```

```
par(bg = "white", mfrow=c(1, 1))
plotColoredClusters(tree_pearson_psesy, labs = row.names(metadata_table),
                    ylab = NA, xlab = NA, cex = 1, las = 1,
                    cols = metadata_table$color, col = "black",
                    main = "Samples Pearson distance hierarchical clustering,
complete linkage, P. syringae genes.")
```

Samples are separated according to their experimental condition,
which is encouraging for further analysis of *P. syringae*.

#### *Rhodococcus enclensis*

```
#### Sample distances ####
message("Computing inter-sample distances")

## Pearson
dist_pearson_rhoen <- as.dist(1 - cor(rhoen_clr, use = "everything", 
                                method = "pearson"))
```

```
message("Sample clustering")

tree_pearson_rhoen <- hclust(dist_pearson_rhoen, method = "complete")
```

```
par(bg = "white", mfrow=c(1, 1))
plotColoredClusters(tree_pearson_rhoen, labs = row.names(metadata_table),
                    ylab = NA, xlab = NA, cex = 1, las = 1,
                    cols = metadata_table$color, col = "black",
                    main = "Samples Pearson distance hierarchical clustering,
complete linkage, R. enclensis genes.")
```

Again, samples are naturally separated according to their incubation
condition for *R. enclensis*.

## Differential analyses with MTXmodel (whole assemblage metatranscriptomics data)

MTXmodel was used on the complete assemblage at once. As input,
filtered data (without the undetected genes) but untransformed was given
to MTXmodel, as it performs its own CLR normalisation.

`analysis_method = 'LM'`

`correction = 'BH'`,

```
fit_data <- MTXmodel(
    counts_standard, metadata_table, 'MTXmodel_output',
    cores = 2,
    fixed_effects = c('temperature'),
    reference = c("temperature,5"),
    min_abundance = 0,
    min_prevalence = 0,
    normalization = 'CLR',
    analysis_method = 'LM',
    correction = 'BH',
    standardize = FALSE,
    transform = 'NONE',
    plot_scatter = FALSE,
    plot_heatmap = TRUE)
```

```
# Loading MTXmodel results
res_mtx <- read.csv("../scripts/MTXmodel_output/all_results.tsv", sep="\t", row.names="feature")


tryCatch({
  inner_join(rownames_to_column(res_mtx), annotation_table_fig, by=c("rowname" = "gene")) -> res_mtx_fig
  res_mtx_fig %>% filter(abs(qval) <= 0.2) -> res_mtx_fig
  },
  error=function(e){str(e)
  }
)


## Data for final table
annotation_table_long <- read.csv("../data/annotations_final_community_long2.tsv", sep="\t", row.names = "Geneid")

tryCatch({
  annotation_table_long$gene <- row.names(annotation_table_long)
  inner_join(rownames_to_column(res_mtx), annotation_table_long, by=c("rowname" = "gene")) -> res_mtx_filt
  res_mtx_filt %>% filter(abs(qval) <= 0.2) -> res_mtx_filt
  },
  error=function(e){str(e)
  }
)
```

## Differential analyses with DESeq2 (metatranscriptomics data species by species)

As a complementary approach, separate differential analyses were
conducted for each species separately with DESeq2.

### DESeq2 analyses preparation

```
metadata_table$temperature <- factor(metadata_table$temperature)
```

We use filtered counts data (only genes detected in at least 70% of
biological samples). Zeroes are conserved as is. We round the counts
matrix before performing DESeq2 analysis.

```
diohu_df <- counts_filtered[diohu_geneid,]
psegr_df <- counts_filtered[psegr_geneid,]
psesy_df <- counts_filtered[psesy_geneid,]
rhoen_df <- counts_filtered[rhoen_geneid,]
```

```
res_df <- data.frame(gene=character(), 
                     baseMean=numeric(),
                     log2FoldChange=numeric(), 
                     lfcSE=numeric(), 
                     stat=numeric(), 
                     pvalue=numeric(), 
                     padj=numeric(), 
                     condition=factor(),
                     SAMPLE_COMPARISON=factor(),
                     organism=factor())
```

### *Dioszegia hungarica*

```
compute_deseq2_analysis(diohu_df, 
                        metadata_table,
                        #subset_var = "temperature", 
                        #select = "3.5", 
                        contrast_col="temperature", 
                        ref="5", 
                        tested="17") -> res

tryCatch({res$organism <- "D. hungarica"},
  error=function(e){str(e) # prints structure of exception
  })

res_df <- rbind(res_df, res)
```

### *Rhodococcus enclensis*

```
compute_deseq2_analysis(rhoen_df, 
                        metadata_table,
                        #subset_var = "temperature", 
                        #select = "3.5", 
                        contrast_col="temperature", 
                        ref="5", 
                        tested="17") -> res

tryCatch({res$organism <- "R. enclensis"},
  error=function(e){str(e)
  })

res_df <- rbind(res_df, res)
```

### *Pseudomonas syringae*

```
compute_deseq2_analysis(psesy_df, 
                        metadata_table,
                        #subset_var = "temperature", 
                        #select = "3.5", 
                        contrast_col="temperature", 
                        ref="5", 
                        tested="17") -> res

tryCatch({res$organism <- "P. syringae"},
  error=function(e){str(e)
  })

res_df <- rbind(res_df, res)
```

### *Pseudomonas graminis*

```
compute_deseq2_analysis(psegr_df, 
                        metadata_table,
                        #subset_var = "temperature", 
                        #select = "3.5", 
                        contrast_col="temperature", 
                        ref="5", 
                        tested="17") -> res

tryCatch({res$organism <- "P. graminis"},
  error=function(e){str(e)
  })

res_df <- rbind(res_df, res)
```

### Filter DESeq2 data

```
tryCatch({
  res_df %>% inner_join(annotation_table_fig, by="gene") -> tmp_res_df
  tmp_res_df %>% filter(padj <= 0.2) -> res_deseq_fig 
  },
  error=function(e){str(e)
  }
)

rm(tmp_res_df)

## data for final table
annotation_table_long <- read.csv("../data/annotations_final_community_long2.tsv", sep="\t", row.names = "Geneid")

tryCatch({
  annotation_table_long$gene <- row.names(annotation_table_long)
  res_df %>% inner_join(annotation_table_long, by="gene") -> res_df
  res_df %>% filter(padj <= 0.2) -> res_deseq_filt # & abs(log2FoldChange) >= 1
  },
  error=function(e){str(e)
  }
)
```

### Save differentially expressed genes (DEGs) all methods

```
temp_df <- right_join(rownames_to_column(res_mtx), annotation_table_long, join_by("rowname"=="gene"))
rename(temp_df, "gene" = "rowname") -> temp_df


all_genes <- full_join(res_df, temp_df,  by=c("gene", "locus_tag", "transcriptId", "Organism",
                                              "Chr", "Start", "End", "Strand", "Length",
                                              "product", "COG_process", "COG_category",
                                              "COGid", "GOs", "COG_cat", "COG_category_long",
                                              "ecNum" ),
                       suffix = c("", "w"))

rm(temp_df)
columns_to_remove <- grep(".w", names(all_genes))
all_genes %>% dplyr::select(-columns_to_remove) %>% filter(qval<=0.2 | padj<=0.2 ) -> all_genes_filtered

write.table(all_genes_filtered, "../results/DEG_all_methods_community.tsv", sep='\t', row.names = FALSE)

write.table(res_df, "../results/DEG_DESeq2_all_community.tsv", sep='\t', row.names = FALSE)
sign_deseq2_df <- res_df
```

## Plot transcript expression coefficient (SD vs WN)

```
#### Both methods DEGs


full_join(all_genes_filtered, annotation_table_fig, by="gene") -> data


data$title <- "Differentially expressed genes SD vs WN by strain (DESeq2 & MTXmodel)"
data$Organism <- factor(data$organism, levels=c("D. hungarica", "P. graminis", "P. syringae", "R. enclensis"))


custom_strips <- strip_nested(background_x = elem_list_rect(fill = c("lightgrey", 
                                                                     species_colours[["D.hungarica"]], 
                                                                     species_colours[["P.syringae"]], 
                                                                     species_colours[["R.enclensis"]])),
                              text_x = list(element_text(face = "plain", colour = "black", size = 17),
                                            element_text(face = "italic", colour = "white", size = 15),
                                            element_text(face = "italic", colour = "white", size = 15), 
                                            element_text(face = "italic", colour = "white", size = 15)),
                              by_layer_x = FALSE)


ggplot(data, aes(x = coef, y = COG_category.y,  color = after_scale(alpha(fill, 0.3)), fill=COG_category_long.y, alpha=0.7, label=name_figure)) + 
  geom_point(aes(alpha=0.7), position="dodge") +
  geom_violin(aes(alpha=0.3), show.legend = TRUE) +
  annotate("rect", xmin=-Inf, xmax=0, ymin=-Inf, ymax=Inf, fill="#184ca5", alpha=0.1) +
  annotate("rect", xmin=0, xmax=Inf, ymin=-Inf, ymax=Inf, fill="gold", alpha=0.1) +
  geom_vline(xintercept = 0, linetype="dashed") +
  ylab("COG category") +
  xlab("MTXmodel coefficient") +
  ggrepel::geom_text_repel(nudge_y = 0.5, segment.size=0.1, seed = 42) +
  guides(fill=guide_legend(ncol=4), alpha="none", color="none") + #color="none",
  scale_y_discrete(limits=rev(names(vect_COG_category_long))) +
  scale_colour_manual(limits=vect_COG_category_long, values=COG_colours, drop=FALSE) + 
  scale_fill_manual(limits=vect_COG_category_long, values=COG_colours, drop=FALSE) +
  theme(axis.title = element_text(size=13, "Differentially expressed genes SD vs WN"),
        axis.text = element_text(size=13),
        strip.text.y = element_text(size = 17),
        #strip.text.x = element_text(size = 17, face = "italic"),
        legend.text = element_text(size = 12),
        legend.title = element_blank(),
        legend.key.size = unit(0.5, "line"),
        legend.position = "bottom",
        panel.background = element_rect(fill = "#f4f4f4")) +
  facet_nested(~title + Organism, strip = custom_strips, drop=TRUE) -> plot_degs

# print(plot_degs)
# 
# ggsave(plot = plot_degs, filename = "../figures/DEG_day_vs_night_commu_both_methods_vertical.tiff", dpi = 300, width = 13, height = 13, bg="white")
```

```
columns_to_remove <- grep(".w", names(all_genes))
all_genes %>% dplyr::select(-columns_to_remove) %>% filter(!is.na(coef)) -> all_genes_unfiltered

full_join(all_genes_unfiltered, annotation_table_long, by="gene") %>% filter(!is.na(coef)) -> data
data[data$COG_category_long.y == "S: Function Unknown", "COG_category_long.y"] <- "S: Function unknown"
data$title <- "All detected genes"
data$subtitle <- "Assemblage"
data$Organism <- factor(data$organism, levels=c("D. hungarica", "P. graminis", "P. syringae", "R. enclensis"))
data$COG_category_fig <- substr(data$COG_category_long.y, 0, 1)


custom_strips <- strip_nested(background_x = elem_list_rect(fill = c("lightgrey", "black")),
                              text_x = list(element_text(face = "plain", colour = "black", size = 17),
                                            element_text(face = "italic", colour = "white", size = 15)),
                              by_layer_x = FALSE)

custom_strips_strains <- strip_nested(background_x = elem_list_rect(fill = c("lightgrey", 
                                                                     species_colours[["D.hungarica"]], 
                                                                     species_colours[["P.syringae"]], 
                                                                     species_colours[["R.enclensis"]])),
                              text_x = list(element_text(face = "plain", colour = "black", size = 17),
                                            element_text(face = "italic", colour = "white", size = 15),
                                            element_text(face = "italic", colour = "white", size = 15), 
                                            element_text(face = "italic", colour = "white", size = 15)),
                              by_layer_x = FALSE)

ggplot(data, aes(x = coef, y = COG_category_fig,color = after_scale(alpha(fill, 0.3)), fill=COG_category_long.y, alpha=0.9)) + #label=name_figure
  # geom_point(aes(alpha=0.7)) + #, position="dodge"
  geom_violin(aes(alpha=0.3), show.legend = TRUE) +
  geom_boxplot(outlier.colour = "black", width=0.2, color="white", outlier.alpha = 0.4, alpha=0.2, show.legend = FALSE) +
  annotate("rect", xmin=-Inf, xmax=0, ymin=-Inf, ymax=Inf, fill="#184ca5", alpha=0.1) +
  annotate("rect", xmin=0, xmax=Inf, ymin=-Inf, ymax=Inf, fill="gold", alpha=0.1) +
  geom_vline(xintercept = 0, linetype="dashed") +
  ylab("COG category") +
  xlab("MTXmodel coefficient") +
  # ggrepel::geom_text_repel(nudge_y = 0.5, segment.size=0.1, seed = 42) +
  guides(fill=guide_legend(ncol=4), alpha="none", color="none") + #color="none",
  scale_y_discrete(limits=rev(names(vect_COG_category_long))) +
  scale_colour_manual(limits=vect_COG_category_long, values=COG_colours, drop=FALSE) + 
  scale_fill_manual(limits=vect_COG_category_long, values=COG_colours, drop=FALSE) +
  theme(axis.title = element_text(size=13, "Expression coefficient (in SD vs WN) of all assemblage detected genes"),
        axis.text = element_text(size=13),
        strip.text.y = element_text(size = 17),
        #strip.text.x = element_text(size = 17, face = "italic"),
        legend.text = element_text(size = 12),
        legend.title = element_blank(),
        legend.key.size = unit(0.5, "line"),
        legend.position = "bottom",
        panel.background = element_rect(fill = "#f4f4f4")) -> p

  p + facet_nested(~title + subtitle, strip = custom_strips, drop=TRUE) -> plot_assemblage
  p + facet_nested(~title + Organism, strip = custom_strips_strains, drop=TRUE) + 
    guides(fill=guide_legend(ncol=3), alpha="none", color="none") + 
    theme(legend.text = element_text(size = 10))-> plot_strains

# print(plot_assemblage)
# 
ggsave(plot = plot_assemblage, filename = "../figures/expressed_genes_MTX_coeff_assemblage.tiff", dpi = 300, width = 13/3, height = 13, bg="white")

ggsave(plot = plot_strains, filename = "../figures/expressed_genes_MTX_coeff_strains.tiff", dpi = 300, width = 13, height = 13, bg="white")
```

```
plot_all <- ggarrange(plot_assemblage, plot_degs, 
                      labels = c("A", "B"), 
                      common.legend=TRUE,
                      legend="bottom",
                      nrow=1,
                      widths = c(1, 3))

print(plot_all)
```

```
ggsave(plot = plot_all, filename = "../figures/Figure_4.tiff", dpi = 300, width = 13+4.35, height = 13, bg="white")

print(plot_strains)
```

## Venn diagram of RNA differential expression results

```
list_venn <- list(res_deseq_filt[(res_deseq_filt$SAMPLE_COMPARISON == "17_VS_5"), "gene"],
                  res_mtx_filt[, "rowname"])

invisible(grid.newpage())   
draw.pairwise.venn(area1 = length(list_venn[[1]]),
                 area2 = length(list_venn[[2]]),
                 cross.area = length(intersect.Vector(list_venn[[1]], list_venn[[2]])),
                 fill = c("#D53F7F", "#039EBD"),
                 lty = "blank",
                 fontfamily = "Helvetica",
                 cex = rep(2, 3),
                 cat.cex = rep(1.5, 2),
                 cat.pos = c(-50, 50),
                 cat.dist = c(-0.05, -0.05),
                 cat.prompts = TRUE,
                 cat.col = c("#D53F7F", "#039EBD"),
                 cat.fontfamily = "Helvetica",
                 category = c("DESeq  \nalone", "MTXModel  \nassemblage"),
                 title = "Differentially abundant genes found by DESeq2 and MTXmodel",
                 margin = 0.1) -> venn_plot
```

```
# Writing to file

invisible(png(filename = "../figures/Venn_diagram_ALDEXe2_MTX_DESeq_community.png", 
     width = 1000, height = 1000))
invisible(grid.draw(venn_plot))
invisible(dev.off())
```

```
vect_degs <- all_genes_filtered$gene
counts_clr_degs <- as.matrix(counts_clr)[vect_degs,]
```

## Differential Metabolites intensity

```
identified_metabolites <- left_join(metabolomics_annotations[,c("metabolite identification", "ID")],
                                        rownames_to_column(metabolomics_all_times_filtered), by = join_by("ID" == "rowname"))  

identified_metabolites <- identified_metabolites[identified_metabolites$`metabolite identification` != "unknown",]

row.names(identified_metabolites) <- identified_metabolites$`metabolite identification`

identified_metabolites <- identified_metabolites %>% t() %>% data.frame()


identified_metabolites <- identified_metabolites[3:nrow(identified_metabolites),] 
identified_metabolites$sample <- row.names(identified_metabolites)

identified_metabolites <- left_join(identified_metabolites, rownames_to_column(metadata_table_metaB_all_times), by = join_by("sample" == "rowname"))

identified_metabolites %>% rename("2-Aminobenzoic acid" = "X2.aminobenzoic.acid",
                                      "DL-Methionine sulfoxide" = "DL.methionine.sulfoxide",
                                      "Pyridoxal" = "pyridoxal",
                                      "D-Pantothenic acid" = "D.pantothenic.acid" ,
                                      "N6-Acetyl-L-lysine" = "N6.acetyl.L.lysine",
                                      "L-Glutamic acid" = "L.glutamic.acid",
                                      "L-Isoleucine" = "L.isoleucine",
                                      "Butyryl-L-carnitine" = "butyryl.L.carnitine",
                                      "Acetyl-L-carnitine" = "acetyl.L.carnitine",
                                      "Isovaleryl-L-carnitine" = "isovaleryl.L.carnitine") -> identified_metabolites 

identified_metabolites %>%
  pivot_longer(cols = c("2-Aminobenzoic acid", "DL-Methionine sulfoxide", "Pyridoxal", 
                        "D-Pantothenic acid", "N6-Acetyl-L-lysine", "L-Glutamic acid", 
                        "L-Isoleucine", "Butyryl-L-carnitine", "Acetyl-L-carnitine", 
                        "Isovaleryl-L-carnitine"), 
               values_to = "intensity") -> identified_metabolites
 

  
identified_metabolites[identified_metabolites$temperature == 17 & identified_metabolites$time == 0, "condition"] <- "SD_T0"
identified_metabolites[identified_metabolites$temperature == 5 & identified_metabolites$time == 0, "condition"] <- "WN_T0"
identified_metabolites[identified_metabolites$temperature == 17 & identified_metabolites$time == 3, "condition"] <- "SD_TF"
identified_metabolites[identified_metabolites$temperature == 5 & identified_metabolites$time == 3, "condition"] <- "WN_TF"
identified_metabolites[identified_metabolites$temperature == 17, "fill"] <- "#F8AD18"
identified_metabolites[identified_metabolites$temperature == 5, "fill"] <- "#89DDF8"
identified_metabolites[identified_metabolites$temperature == 17, "colour"] <- "#b0790b"
identified_metabolites[identified_metabolites$temperature == 5, "colour"] <- "#1aa7d4"

identified_metabolites$intensity <- as.numeric(identified_metabolites$intensity)
```

### Differentially abundant metabolites between SD and WN

```
identified_metabolites %>% 
  filter(name %in% c("2-Aminobenzoic acid", "L-Glutamic acid",
                     "DL-Methionine sulfoxide", "N6-Acetyl-L-lysine",
                     "D-Pantothenic acid", "Pyridoxal" )) %>%
filter(condition %in% c("SD_TF", "WN_TF")) %>%
  # ggplot(aes(y=intensity, x=condition, group=condition)) +
  ggplot(aes(y=intensity, x=name, fill=condition)) +
  geom_boxplot(aes(fill=fill, colour=colour)) +
  scale_fill_identity() +
  scale_color_identity() +
  ylab(expression("intensity * 10"^" -2")) + 
  theme(strip.text = element_text(face = "bold", size=12),
        axis.title.x = element_blank(),
        axis.text.x = element_text(size=10)) +
  scale_x_discrete(labels = function(x) str_wrap(x, width = 14, whitespace_only = FALSE)) +
  # facet_wrap(~name, scale="free", ncol=2) +
  scale_y_continuous(labels = function(x) format(x * 100, scientific = FALSE), 
                     limits = c(0,0.055), breaks = extended_breaks(n=8)) -> plot_wn


identified_metabolites %>% 
  filter(name %in% c("L-Isoleucine", "Acetyl-L-carnitine",
                     "Butyryl-L-carnitine",
                     "Isovaleryl-L-carnitine")) %>%
filter(condition %in% c("SD_TF", "WN_TF")) %>%
  # ggplot(aes(y=intensity, x=condition, group=condition)) +
  ggplot(aes(y=intensity, x=name, fill=condition)) +
  geom_boxplot(aes(fill=fill, colour=colour)) +
  scale_fill_identity("Condition", labels=c("Winter Night", "Summer Day"), guide = "legend") +
  scale_color_identity("Condition", labels=c("Winter Night", "Summer Day"), guide = "legend") +
  ylab(expression("intensity * 10"^" -2")) + 
  theme(strip.text = element_text(face = "bold", size=12),
        axis.title.x = element_blank(),
        axis.text.x = element_text(size=10)) +
  scale_x_discrete(labels = function(x) str_wrap(x, width = 14, whitespace_only = FALSE)) +
  # facet_wrap(~name, scale="free", ncol=2) +
  scale_y_continuous(labels = function(x) format(x * 100, scientific = FALSE), 
                     limits = c(0,0.055), breaks = extended_breaks(n=8)) -> plot_sd

plot_all <- ggarrange(plot_sd, plot_wn,  
                      labels = c("A", "B"), 
                      common.legend=TRUE,
                      legend="bottom",
                      nrow=1,
                      widths = c(4, 6))

print(plot_all)
```

```
ggsave(plot = plot_all, filename = "../figures/Figure_3.tiff", dpi = 300, width = 12, height = 6, bg="white")
```

### Session information

```
sessionInfo()
```

```
R version 4.3.1 (2023-06-16 ucrt)
Platform: x86_64-w64-mingw32/x64 (64-bit)
Running under: Windows 11 x64 (build 26100)

Matrix products: default


locale:
[1] LC_COLLATE=French_France.utf8  LC_CTYPE=French_France.utf8    LC_MONETARY=French_France.utf8 LC_NUMERIC=C                   LC_TIME=French_France.utf8    

time zone: Europe/Paris
tzcode source: internal

attached base packages:
 [1] splines   stats4    grid      stats     graphics  grDevices utils     datasets  methods   base     

other attached packages:
 [1] ggridges_0.5.6              rlang_1.1.3                 lubridate_1.9.3             forcats_1.0.0               stringr_1.5.1               dplyr_1.1.4                 purrr_1.0.2                 readr_2.1.5                 tidyr_1.3.1                 tidyverse_2.0.0            
[11] ggtext_0.1.2                cplm_0.7-12                 Matrix_1.6-5                coda_0.19-4.1               compositions_2.0-8          ClassDiscovery_3.4.0        oompaBase_3.2.9             cluster_2.1.6               BiocManager_1.30.22         data.table_1.15.2          
[21] FactoMineR_2.10             ggh4x_0.2.8                 knitr_1.45                  pheatmap_1.0.12             rgl_1.3.1                   scales_1.3.0                tibble_3.2.1                vegan_2.6-4                 lattice_0.22-5              permute_0.9-7              
[31] VennDiagram_1.7.3           futile.logger_1.4.3         MTXmodel_1.2.4              DESeq2_1.40.2               SummarizedExperiment_1.30.2 Biobase_2.60.0              MatrixGenerics_1.12.3       matrixStats_1.2.0           GenomicRanges_1.52.0        GenomeInfoDb_1.36.4        
[41] IRanges_2.34.1              S4Vectors_0.38.2            BiocGenerics_0.46.0         ggpubr_0.6.0                ggplot2_3.4.3               devtools_2.4.5              usethis_2.2.3              

loaded via a namespace (and not attached):
  [1] later_1.3.2             bitops_1.0-7            cellranger_1.1.0        lpsymphony_1.30.0       lifecycle_1.0.4         rstatix_0.7.2           MASS_7.3-60.0.1         oompaData_3.1.3         flashClust_1.01-2       backports_1.4.1         magrittr_2.0.3          sass_0.4.8             
 [13] rmarkdown_2.26          jquerylib_0.1.4         yaml_2.3.8              remotes_2.4.2.1         httpuv_1.6.14           sessioninfo_1.2.2       pkgbuild_1.4.3          cowplot_1.1.3.9000      bayesm_3.1-6            DBI_1.2.2               minqa_1.2.6             RColorBrewer_1.1-3     
 [25] multcomp_1.4-25         abind_1.4-5             pkgload_1.3.4           zlibbioc_1.46.0         RCurl_1.98-1.14         TH.data_1.1-2           tensorA_0.36.2.1        sandwich_3.1-0          GenomeInfoDbData_1.2.11 ggrepel_0.9.5           tweedie_2.3.5           commonmark_1.9.1       
 [37] codetools_0.2-19        getopt_1.20.4           DelayedArray_0.26.7     DT_0.32                 xml2_1.3.6              tidyselect_1.2.1        farver_2.1.2            base64enc_0.1-3         jsonlite_1.8.8          ellipsis_0.3.2          survival_3.5-8          emmeans_1.10.0         
 [49] systemfonts_1.0.6       tools_4.3.1             ragg_1.3.0              Rcpp_1.0.12             glue_1.7.0              gridExtra_2.3           xfun_0.42               mgcv_1.9-1              withr_3.0.1             formatR_1.14            fastmap_1.1.1           fansi_1.0.6            
 [61] digest_0.6.35           timechange_0.3.0        R6_2.5.1                mime_0.12               estimability_1.5        textshaping_0.3.7       colorspace_2.1-0        markdown_1.12           utf8_1.2.4              generics_0.1.3          robustbase_0.99-2       htmlwidgets_1.6.4      
 [73] S4Arrays_1.0.6          scatterplot3d_0.3-44    pkgconfig_2.0.3         gtable_0.3.5            XVector_0.40.0          pcaPP_2.0-4             htmltools_0.5.7         carData_3.0-5           profvis_0.3.8           multcompView_0.1-10     leaps_3.1               optparse_1.7.4         
 [85] lambda.r_1.2.4          rstudioapi_0.15.0       tzdb_0.4.0              reshape2_1.4.4          nlme_3.1-164            cachem_1.0.8            zoo_1.8-12              parallel_4.3.1          miniUI_0.1.1.1          pillar_1.9.0            vctrs_0.6.5             urlchecker_1.0.1       
 [97] promises_1.2.1          car_3.1-2               xtable_1.8-4            evaluate_0.23           mvtnorm_1.2-4           cli_3.6.1               locfit_1.5-9.9          compiler_4.3.1          futile.options_1.0.1    crayon_1.5.2            ggsignif_0.6.4          labeling_0.4.3         
[109] mclust_6.1              plyr_1.8.9              fs_1.6.3                stringi_1.8.3           BiocParallel_1.34.2     munsell_0.5.1           hms_1.1.3               statmod_1.5.0           shiny_1.8.0             highr_0.10              gridtext_0.1.5          broom_1.0.5            
[121] memoise_2.0.1           bslib_0.6.1             biglm_0.9-2.1           DEoptimR_1.1-3          readxl_1.4.3
```

LS0tDQp0aXRsZTogIlN0YXRpc3RpY2FsIGFuYWx5c2lzIHJlcG9ydCBmb3IgJ011bHRpLWtpbmdkb20gbWljcm9iaWFsIGFzc2VtYmxhZ2UgbW9kdWxhdGVzIGl0cyBtZXRhYm9saXNtIHVuZGVyIGNvbnRyYXN0ZWQgY2xvdWQgY29uZGl0aW9ucycuIg0KYXV0aG9yOiAiRG9taXRpbGxlIEphcnJpZ2UiDQpkYXRlOiAnYHIgU3lzLkRhdGUoKWAnDQpvdXRwdXQ6DQogIGh0bWxfZG9jdW1lbnQ6DQogICAgc2VsZl9jb250YWluZWQ6IHllcw0KICAgIGNvZGVfZG93bmxvYWQ6IHRydWUNCiAgICBmaWdfY2FwdGlvbjogeWVzDQogICAgaGlnaGxpZ2h0OiB6ZW5idXJuDQogICAgdGhlbWU6IGNlcnVsZWFuDQogICAgdG9jOiB5ZXMNCiAgICB0b2NfZGVwdGg6IDMNCiAgICB0b2NfZmxvYXQ6IHllcw0KICAgIGNvZGVfZm9sZGluZzogImhpZGUiDQogIHBkZl9kb2N1bWVudDoNCiAgICBmaWdfY2FwdGlvbjogeWVzDQogICAgaGlnaGxpZ2h0OiB6ZW5idXJuDQogICAgdG9jOiB5ZXMNCiAgICB0b2NfZGVwdGg6IDMNCmVkaXRvcl9vcHRpb25zOiANCiAgY2h1bmtfb3V0cHV0X3R5cGU6IGNvbnNvbGUNCi0tLQ0KDQpgYGB7ciBzZXR0aW5ncywgaW5jbHVkZT1GQUxTRSwgZWNobz1GQUxTRSwgZXZhbD1UUlVFfQ0Kb3B0aW9ucyh3aWR0aCA9IDMwMCkNCmtuaXRyOjpvcHRzX2NodW5rJHNldCgNCiAgZmlnLndpZHRoID0gOSwgZmlnLmhlaWdodCA9IDksIA0KICBmaWcucGF0aCA9ICJDOi9Vc2Vycy9kamFycmlnZS9EZXNrdG9wL0RvbWl0aWxsZS9wcm9qZXRzL0FuY2llbnNfcHJvamV0cy9NRVRBQ0xPVUQvZmlndXJlcyIsDQogIGZpZy5hbGlnbiA9ICJjZW50ZXIiLCANCiAgc2l6ZSA9ICJzbWFsbCIsIA0KICBlY2hvID0gVFJVRSwgDQogIGV2YWwgPSBUUlVFLCANCiAgd2FybmluZyA9IEZBTFNFLCANCiAgbWVzc2FnZSA9IEZBTFNFLCANCiAgcmVzdWx0cyA9IFRSVUUsIA0KICBjb21tZW50ID0gIiIpDQoNCm9wdGlvbnMoc2NpcGVuID0gMTIpICMjIE1heCBudW1iZXIgb2YgZGlnaXRzIGZvciBub24tc2NpZW50aWZpYyBub3RhdGlvbg0KDQpgYGANCg0KYGBge3Igc2V0X3dvcmtpbmdfZGlyZWN0b3J5fQ0KDQpzZXR3ZCgiQzovVXNlcnMvZGphcnJpZ2UvRGVza3RvcC9Eb21pdGlsbGUvcHJvamV0cy9BbmNpZW5zX3Byb2pldHMvTUVUQUNMT1VEL3NjcmlwdHMvIikNCg0KYGBgDQoNCiMgTGlicmFyaWVzDQoNCmBgYHtyIGxpYnJhcmllcywgZWNobz1GQUxTRSwgZXZhbD1UUlVFfQ0KDQoNCmlmICghcmVxdWlyZU5hbWVzcGFjZSgiZGV2dG9vbHMiLCBxdWlldGx5ID0gVFJVRSkpDQogICAgaW5zdGFsbC5wYWNrYWdlcyhkZXZ0b29scykNCiAgbGlicmFyeSgiZGV2dG9vbHMiLCBjaGFyYWN0ZXIub25seSA9IFRSVUUpDQoNCg0KbGlzdF9saWJfdmVyIDwtIGxpc3QoYygic3RhdHMiKSwgIGMoImdyaWQiKSwgYygidXRpbHMiKSwgYygiZ3JhcGhpY3MiKSwgDQogICAgICAgICAgICAgICAgICAgICBjKCJnZ3Bsb3QyIiwgIjMuNC4zIiksIGMoImdncHViciIsICIwLjYuMCIpLA0KICAgICAgICAgICAgICAgICAgICAgYygiREVTZXEyIiwgIjEuNDAuMiIpLCBjKCJNVFhtb2RlbCIsICIxLjIuNCIpLCANCiAgICAgICAgICAgICAgICAgICAgIGMoIlZlbm5EaWFncmFtIiwgIjEuNy4zIiksIGMoInZlZ2FuIiwgIjIuNi00IiksDQogICAgICAgICAgICAgICAgICAgICBjKCJ0aWJibGUiLCAiMy4yLjEiKSwgYygic2NhbGVzIiwgIjEuMy4wIiksDQogICAgICAgICAgICAgICAgICAgICBjKCJTNFZlY3RvcnMiLCAiMC4zOC4yIiksIGMoInJnbCIsICIxLjMuMSIpLA0KICAgICAgICAgICAgICAgICAgICAgYygicGhlYXRtYXAiLCAiMS4wLjEyIiksIGMoImtuaXRyIiwgIjEuNDUiKSwNCiAgICAgICAgICAgICAgICAgICAgIGMoImdnaDR4IiwgIjAuMi44IiksIGMoIkZhY3RvTWluZVIiLCAiMi4xMCIpLA0KICAgICAgICAgICAgICAgICAgICAgYygiZGF0YS50YWJsZSIsICIxLjE1LjIiKSwgYygiQmlvY01hbmFnZXIiLCAiMS4zMC4yMiIpLA0KICAgICAgICAgICAgICAgICAgICAgYygiQ2xhc3NEaXNjb3ZlcnkiLCAiMy40LjAiKSwgYygiY29tcG9zaXRpb25zIiwgIjIuMC04IiksDQogICAgICAgICAgICAgICAgICAgICBjKCJjcGxtIiwgIjAuNy0xMiIpLCBjKCJnZ3RleHQiLCAiMC4xLjIiKSwNCiAgICAgICAgICAgICAgICAgICAgIGMoInRpZHl2ZXJzZSIsICIyLjAuMCIpLCBjKCJybGFuZyIsICIxLjEuMyIpLA0KICAgICAgICAgICAgICAgICAgICAgYygic3RyaW5nciIsICIxLjUuMSIpLCBjKCJnZ3JpZGdlcyIsICIwLjUuNiIpICMsIGMoImdndGV4dCIsICIwLjEuMiIpDQogICAgICAgICAgICAgICAgICAgICApDQoNCmZvciAobGliIGluIGxpc3RfbGliX3Zlcil7DQogIGlmICghcmVxdWlyZU5hbWVzcGFjZShsaWJbWzFdXSwgcXVpZXRseSA9IFRSVUUpKQ0KICAgICAgZGV2dG9vbHM6Omluc3RhbGxfdmVyc2lvbihsaWJbWzFdXSwgbGliW1syXV0pDQogICAgbGlicmFyeShsaWJbWzFdXSwgY2hhcmFjdGVyLm9ubHkgPSBUUlVFKQ0KfQ0KDQoNCmlmICghcmVxdWlyZSgiTVRYbW9kZWwiLCBjaGFyYWN0ZXIub25seSA9IFRSVUUpKSB7DQogICAgZGV2dG9vbHM6aW5zdGFsbF9naXRodWIoJ2Jpb2Jha2VyeS9NVFhfbW9kZWwnKQ0KfQ0KbGlicmFyeSgiTVRYbW9kZWwiKQ0KDQoNCiMgdG8gaW5jbHVkZSAzRCBwbG90cyBpbiBodG1sIHJlcG9ydA0Ka25pdHI6OmtuaXRfaG9va3Mkc2V0KHdlYmdsID0gaG9va193ZWJnbCkNCg0KYGBgDQoNCg0KIyBWYXJpYWJsZXMNCg0KYGBge3IgdmFyaWFibGVzfQ0KdmVjdF9DT0dfY2F0ZWdvcnlfbG9uZyA8LSBjKCJBIiA9ICJBOiBSTkEgcHJvY2Vzc2luZyBhbmQgbW9kaWZpY2F0aW9uIiwNCiAgICAgICAgICAgICAgICAgICAgICAgICAgICAiQiIgPSAiQjogQ2hyb21hdGluIFN0cnVjdHVyZSBhbmQgZHluYW1pY3MiLA0KICAgICAgICAgICAgICAgICAgICAgICAgICAgICJDIiA9ICJDOiBFbmVyZ3kgcHJvZHVjdGlvbiBhbmQgY29udmVyc2lvbiIsDQogICAgICAgICAgICAgICAgICAgICAgICAgICAgIkQiID0gIkQ6IENlbGwgY3ljbGUgY29udHJvbCwgY2VsbCBkaXZpc2lvbiwgY2hyb21vc29tZSBwYXJ0aXRpb25pbmciLA0KICAgICAgICAgICAgICAgICAgICAgICAgICAgICJFIiA9ICJFOiBBbWlubyBBY2lkIHRyYW5zcG9ydCBhbmQgbWV0YWJvbGlzbSIsDQogICAgICAgICAgICAgICAgICAgICAgICAgICAgIkYiID0gIkY6IE51Y2xlb3RpZGUgdHJhbnNwb3J0IGFuZCBtZXRhYm9saXNtIiwNCiAgICAgICAgICAgICAgICAgICAgICAgICAgICAiRyIgPSAiRzogQ2FyYm9oeWRyYXRlIHRyYW5zcG9ydCBhbmQgbWV0YWJvbGlzbSIsDQogICAgICAgICAgICAgICAgICAgICAgICAgICAgIkgiID0gIkg6IENvZW56eW1lIHRyYW5zcG9ydCBhbmQgbWV0YWJvbGlzbSIsDQogICAgICAgICAgICAgICAgICAgICAgICAgICAgIkkiID0gIkk6IExpcGlkIHRyYW5zcG9ydCBhbmQgbWV0YWJvbGlzbSIsDQogICAgICAgICAgICAgICAgICAgICAgICAgICAgIkoiID0gIko6IFRyYW5zbGF0aW9uLCByaWJvc29tYWwgc3RydWN0dXJlIGFuZCBiaW9nZW5lc2lzIiwNCiAgICAgICAgICAgICAgICAgICAgICAgICAgICAiSyIgPSAiSzogVHJhbnNjcmlwdGlvbiIsDQogICAgICAgICAgICAgICAgICAgICAgICAgICAgIkwiID0gIkw6IFJlcGxpY2F0aW9uLCByZWNvbWJpbmF0aW9uIGFuZCByZXBhaXIiLA0KICAgICAgICAgICAgICAgICAgICAgICAgICAgICJNIiA9ICJNOiBDZWxsIHdhbGwvbWVtYnJhbmUvZW52ZWxvcGUgYmlvZ2VuZXNpcyIsDQogICAgICAgICAgICAgICAgICAgICAgICAgICAgIk4iID0gIk46IENlbGwgbW90aWxpdHkiLA0KICAgICAgICAgICAgICAgICAgICAgICAgICAgICJPIiA9ICJPOiBQb3N0LXRyYW5zbGF0aW9uYWwgbW9kaWZpY2F0aW9uLCBwcm90ZWluIHR1cm5vdmVyLCBjaGFwZXJvbmVzIiwNCiAgICAgICAgICAgICAgICAgICAgICAgICAgICAiUCIgPSAiUDogSW5vcmdhbmljIGlvbiB0cmFuc3BvcnQgYW5kIG1ldGFib2xpc20iLA0KICAgICAgICAgICAgICAgICAgICAgICAgICAgICJRIiA9ICJROiBTZWNvbmRhcnkgbWV0YWJvbGl0ZXMgYmlvc3ludGhlc2lzLCB0cmFuc3BvcnQgYW5kIGNhdGFib2xpc20iLA0KICAgICAgICAgICAgICAgICAgICAgICAgICAgICJUIiA9ICJUOiBTaWduYWwgVHJhbnNkdWN0aW9uIG1lY2hhbmlzbXMiLA0KICAgICAgICAgICAgICAgICAgICAgICAgICAgICJVIiA9ICJVOiBJbnRyYWNlbGx1bGFyIHRyYWZmaWNraW5nLCBzZWNyZXRpb24gYW5kIHZlc2ljdWxhciB0cmFuc3BvcnQiLA0KICAgICAgICAgICAgICAgICAgICAgICAgICAgICJWIiA9ICJWOiBEZWZlbnNlIG1lY2hhbmlzbXMiLA0KICAgICAgICAgICAgICAgICAgICAgICAgICAgICJXIiA9ICJXOiBFeHRyYWNlbGx1bGFyIHN0cnVjdHVyZXMiLA0KICAgICAgICAgICAgICAgICAgICAgICAgICAgICJYIiA9ICJYOiBNb2JpbG9tZTogcHJvcGhhZ2VzLCB0cmFuc3Bvc29ucyIsDQogICAgICAgICAgICAgICAgICAgICAgICAgICAgIlkiID0gIlk6IE51Y2xlYXIgc3RydWN0dXJlIiwNCiAgICAgICAgICAgICAgICAgICAgICAgICAgICAiWiIgPSAiWjogQ3l0b3NrZWxldG9uIiwNCiAgICAgICAgICAgICAgICAgICAgICAgICAgICAiUiIgPSAiUjogR2VuZXJhbCBmdW5jdGlvbmFsIHByZWRpY3Rpb24gb25seSIsDQogICAgICAgICAgICAgICAgICAgICAgICAgICAgIlMiID0gIlM6IEZ1bmN0aW9uIHVua25vd24iICMsDQogICAgICAgICAgICAgICAgICAgICAgICAgICAgIyAiLSIgPSAiLTogT3RoZXIiDQogICAgICAgICAgICAgICAgICAgICAgICAgICAgKQ0KDQoNCkNPR19jb2xvdXJzIDwtIGh1ZV9wYWwoKShsZW5ndGgodmVjdF9DT0dfY2F0ZWdvcnlfbG9uZykpDQpuYW1lcyhDT0dfY29sb3VycykgPC0gdmVjdF9DT0dfY2F0ZWdvcnlfbG9uZw0KDQpzcGVjaWVzX2NvbG91cnMgPC0gYygiRC5odW5nYXJpY2EiID0gIiNlNjYxMDEiLCAiUC5ncmFtaW5pcyIgPSAiI2ZkYjg2MyIsIA0KICAgICAgICAgICAgICAgICAgICAgIlAuc3lyaW5nYWUiID0gIiNiMmFiZDIiLCAiUi5lbmNsZW5zaXMiID0gIiM1ZTNjOTkiKQ0KYGBgDQoNCiMgRnVuY3Rpb25zDQoNCmBgYHtyIGZ1bmN0aW9uc30NCg0KY29tcHV0ZV9kZXNlcTJfYW5hbHlzaXMgPSBmdW5jdGlvbihteURhdGEsIHNhbXBsZV9kYXRhLCBzdWJzZXRfdmFyPUZBTFNFLCBzZWxlY3Q9RkFMU0UsIA0KICAgICAgICAgICAgICAgICAgICAgICAgICAgICAgICAgICBjb250cmFzdF9jb2wsIHJlZiwgdGVzdGVkKXsNCiAgZGF0YSA8LSBteURhdGENCiAgc2FtcGxlIDwtIHNhbXBsZV9kYXRhDQogIGlmICgoc3Vic2V0X3ZhciAhPSBGQUxTRSkgJiAoc2VsZWN0ICE9IEZBTFNFKSl7DQogICAgc2FtcGxlICU+JSBmaWx0ZXIoISFhcy5uYW1lKHN1YnNldF92YXIpID09IHNlbGVjdCkgLT4gc2FtcGxlDQogIH0NCiAgDQogIHNhbXBsZVtbY29udHJhc3RfY29sXV0gPC0gZmFjdG9yKHNhbXBsZVtbY29udHJhc3RfY29sXV0pDQogIGRhdGEgPC0gZGF0YVtyb3cubmFtZXMoc2FtcGxlKV0NCiAgDQogIGRhdGFfbWF0cml4IDwtIHJvdW5kKGFzLm1hdHJpeChkYXRhKSkNCiAgZCA9IGZvcm11bGEocGFzdGUoIn4iLCAiICIsIGNvbnRyYXN0X2NvbCkpDQogIA0KICBkZHMgPC0gREVTZXFEYXRhU2V0RnJvbU1hdHJpeChjb3VudERhdGEgPSBkYXRhX21hdHJpeCwgDQogICAgICAgICAgICAgICAgICAgICAgICAgICAgICAgIGNvbERhdGEgPSBzYW1wbGUsDQogICAgICAgICAgICAgICAgICAgICAgICAgICAgICAgIGRlc2lnbiA9IGQpDQogIGRkcyA8LSBlc3RpbWF0ZVNpemVGYWN0b3JzKGRkcykNCiAgZGRzIDwtIERFU2VxKGRkcykNCiAgcmVzdWx0c05hbWVzKGRkcykNCiAgcmVzREVTZXEgPC0gcmVzdWx0cyhkZHMsIGNvbnRyYXN0ID0gYyhjb250cmFzdF9jb2wsIHRlc3RlZCwgcmVmKSwNCiAgICAgICAgICAgICAgICAgICAgICBpbmRlcGVuZGVudEZpbHRlcmluZyA9IFRSVUUsIGFscGhhPTAuMSkNCiAgDQogIHJlc0RFU2VxIDwtIHJlc0RFU2VxW29yZGVyKHJlc0RFU2VxJHBhZGopLF0NCiAgcmVzIDwtIGRhdGEuZnJhbWUocmVzREVTZXEpDQogIHJlcyRnZW5lID0gcm93Lm5hbWVzKHJlcykNCiAgcmVzJGNvbmRpdGlvbiA9IHNlbGVjdA0KICByZXMkU0FNUExFX0NPTVBBUklTT04gPSBwYXN0ZTAodGVzdGVkLCAiX1ZTXyIsIHJlZikgDQogIA0KICByZXR1cm4ocmVzKQ0KfQ0KYGBgDQoNCiMgRXhwZXJpbWVudGFsIHNldHVwDQoNCkNvbXBhcmlzb24gb2YgYW4gYXJ0aWZpY2lhbCBtaWNyb2JpYWwgYXNzZW1ibGFnZSBnZW5lIGV4cHJlc3Npb24gdW5kZXIgdHdvIGNsb3VkLWxpa2UgY29uZGl0aW9uczogDQoNCiAtICoqc3VtbWVyIGRheSAoU0QpOioqIGxpZ2h0LCAyNTDCtU0gSDJPMiwgMTfCsEMgDQoNCiAtICoqd2ludGVyIG5pZ2h0IChXTik6KiogZGFyaywgbm8gYWRkZWQgSDJPMiwgNcKwQw0KDQoNCiFbXSguLi9maWd1cmVzL2V4cGVyaW1lbnRhbF9zZXR1cC5wbmcpDQoNCg0KIyBCaW9pbmZvcm1hdGljIHdvcmtmbG93IGFuZCBtYXBwaW5nIG92ZXJ2aWV3DQoNClRoZSBtZXRhdHJhbnNjcmlwdG9taWNzIGRhdGEgd2FzIHByb2Nlc3NlZCB1c2luZyBhIGN1c3RvbSBtYWRlIFNuYWtlbWFrZSB3b3JrZmxvdy4NCg0KIVtdKC4uL2ZpZ3VyZXMvbWV0YWdlbm9taWNfYW5hbHlzZXNfY29tbXVuaXR5NS5kcmF3aW8ucG5nKSANCg0KVHdvIHNlcXVlbmNpbmcgcnVucyB3ZXJlIHBlcmZvcm1lZCBieSBHZW5vc2NyZWVuIChMaWxsZSwgRnJhbmNlKSBhcyB0aGUgZmlyc3QgcnVuIHByb2R1Y2VkIHJlbGF0aXZlbHkgbG93IHF1YWxpdHkgcmVhZHMuIEJvdGggcnVucyBhcmUgYWRkZWQgaW50byBhIHNpbmdsZSBkYXRhc2V0IGluIG91ciBhbmFseXNlcy4NCg0KQWZ0ZXIgcXVhbGl0eSBjb250cm9sIHN0ZXBzIGFuZCByZWFkIGNsZWFuaW5nIChbcmVwb3J0XShodHRwczovL3NlYWZpbGUudW5pc3RyYS5mci9mLzYxZTFjOWJkOTdmMDQ5YTJhNGY0Lz9kbD0xKSkgcmVtYWluaW5nIHJSTkEgKG5vdCBkZXBsZXRlZCBwcmlvciB0byBzZXF1ZW5jaW5nKSB3ZXJlIHJlbW92ZWQgd2l0aCBzb3J0bWVSTkEuIEFzIGZ1bmdhbCByUk5BIHdlcmUgbm90IGRlcGxldGVkIGluIG91ciBsaWJyYXJpZXMsIHRoZSB2YXN0IG1ham9yaXR5IG9mIG91ciByZWFkcyBjb3JyZXNwb25kIHRvICpEaW9zemVnaWEqICpodW5nYXJpY2EqIHJSTkFzLg0KDQpOb24gcmlib3NvbWFsIGFuZCByaWJvc29tYWwgUk5BcyB3ZXJlIHRoZW4gbWFwcGVkIHNlcGFyYXRlbHkgb24gb3VyIHJlZmVyZW5jZSBnZW5vbWVzIHVzaW5nIFNUQVIuDQoNCkZvciBub24gclJOQSByZWFkczogKipcfjkwLTk1JSBvZiByZWFkcyBtYXBwZWQgaW4gYmlvbG9naWNhbCBzYW1wbGVzKiouIEluIGJsYW5rIHNhbXBsZXMsIGFyb3VuZCA0MCUgb2YgcmVhZHMgd2VyZSBtYXBwZWQsIGFuZCBvbmx5IHBhcnRpYWxseS4NCg0KRmV3IHJlYWRzIHdlcmUgbWFwcGVkIG9uICpQLiBncmFtaW5pcyogUERELTEzYi0zIGdlbm9tZS4gSXQgd2lsbCBiZSBkaWZmaWN1bHQgdG8gZ2V0IHNpZ25pZmljYW50IHJlc3VsdHMgZm9yIHRoaXMgc3BlY2llcy4NCg0KTGFzdGx5LCBmb3IgZWFjaCBnZW5lIG9mIHRoZSBhc3NlbWJsYWdlLCByZWFkIGNvdW50cyB3ZXJlIG9idGFpbmVkIHdpdGggZmVhdHVyZUNvdW50cy4nLU0nIGFuZCAnLS1mcmFjdGlvbicgb3B0aW9ucyB3ZXJlIHVzZWQgdG8gY291bnQgbXVsdGktbWFwcGluZyByZWFkcyBmcmFjdGlvbmFsbHkgKGlmIGEgcmVhZCBtYXBzIG9uIHggZmVhdHVyZXM6IGVhY2ggZmVhdHVyZSBnZXRzIDEveCBjb3VudHMpLg0KDQoNCiMgQW5hbHlzZXMNCg0KDQojIyMjIyBMb2FkIGNvdW50cyBhbmQgYW5ub3RhdGlvbiBkYXRhDQoNCmBgYHtyIGxvYWRfZGF0YX0NCg0KY291bnRzX3RhYmxlIDwtIHJlYWQuY3N2KCIuLi9yZXN1bHRzL2FsbF9jb3VudHNfY29tbXVuaXR5X2FydGlmaWNpYWxfbm9fclJOQS50c3YiLCBzZXAgPSAiXHQiLCByb3cubmFtZXMgPSAiR2VuZWlkIikNCg0KbWV0YWRhdGFfdGFibGUgPC0gcmVhZC5jc3YoIi4uL2RhdGEvbWV0YWRhdGEudHh0Iiwgc2VwID0gIlx0Iiwgcm93Lm5hbWVzID0gIm5hbWUiKSAjIHJvdy5uYW1lcyA9ICJzYW1wbGUiDQoNCg0KY291bnRzX3RhYmxlICU+JSByZW5hbWUoIldOX1RGXzEiID0gIlNfNUMxIiwgDQogICAgICAgICAgICAgICAgICAgICAgICAiV05fVEZfMiIgPSAiU181QzIiLCANCiAgICAgICAgICAgICAgICAgICAgICAgICJXTl9URl8zIiA9ICJTXzVDMyIsDQogICAgICAgICAgICAgICAgICAgICAgICAiV05fQkxLIiA9ICJTXzVCTEsiLA0KICAgICAgICAgICAgICAgICAgICAgICAgIlNEX1RGXzEiID0gIlNfMTdDMSIsIA0KICAgICAgICAgICAgICAgICAgICAgICAgIlNEX1RGXzIiID0gIlNfMTdDMiIsIA0KICAgICAgICAgICAgICAgICAgICAgICAgIlNEX1RGXzMiID0gIlNfMTdDMyIsDQogICAgICAgICAgICAgICAgICAgICAgICAiU0RfQkxLIiA9ICJTXzE3QkxLIikgLT4gY291bnRzX3RhYmxlDQoNCmFubm90YXRpb25fdGFibGUgPC0gcmVhZC5jc3YoIi4uL2RhdGEvYW5ub3RhdGlvbnNfZmluYWxfY29tbXVuaXR5X3VwZGF0ZWQudHN2Iiwgc2VwPSJcdCIsIHJvdy5uYW1lcyA9ICJHZW5laWQiKQ0KYW5ub3RhdGlvbl9hbGwgPC0gcmVhZC5jc3YoIi4uL2RhdGEvYW5ub3RhdGlvbnNfZmluYWxfY29tbXVuaXR5X3VwZGF0ZWQudHN2Iiwgc2VwPSJcdCIsIHJvdy5uYW1lcyA9ICJHZW5laWQiKQ0KYW5ub3RhdGlvbl90YWJsZV9sb25nIDwtIHJlYWQuY3N2KCIuLi9kYXRhL2Fubm90YXRpb25zX2ZpbmFsX2NvbW11bml0eV9sb25nMi50c3YiLCBzZXA9Ilx0Iiwgcm93Lm5hbWVzID0gIkdlbmVpZCIpDQoNCiMjIGFkZGVkIGp1bHkgMjAyNA0KY291bnRzX3RhYmxlIDwtIGNvdW50c190YWJsZVtpbnRlcnNlY3Qocm93bmFtZXMoY291bnRzX3RhYmxlKSwgcm93bmFtZXMoYW5ub3RhdGlvbl9hbGwpKSxdDQoNCg0KDQphbm5vdGF0aW9uX2Rpb2h1IDwtIHJlYWQuY3N2KCIuLi9kYXRhL2Rpb3N6ZWdpYV9rZWdnLnRzdiIsIHNlcD0iXHQiLCByb3cubmFtZXMgPSAiR2VuZWlkIikNCmFubm90YXRpb25fcHNlZ3IgPC0gcmVhZC5jc3YoIi4uL2RhdGEvcHNldWRvbW9uYXNfZ3JhbWluaXNfa2VnZy50c3YiLCBzZXA9Ilx0Iiwgcm93Lm5hbWVzID0gIkdlbmVpZCIpDQphbm5vdGF0aW9uX3BzZXN5IDwtIHJlYWQuY3N2KCIuLi9kYXRhL3BzZXVkb21vbmFzX3N5cmluZ2FlX2tlZ2cudHN2Iiwgc2VwPSJcdCIsIHJvdy5uYW1lcyA9ICJHZW5laWQiKQ0KYW5ub3RhdGlvbl9yaG9lbiA8LSByZWFkLmNzdigiLi4vZGF0YS9yaG9kb2NvY2N1c19rZWdnLnRzdiIsIHNlcD0iXHQiLCByb3cubmFtZXMgPSAiR2VuZWlkIikNCg0KY2hlbV9kYXRhX21ldGFUIDwtIHJlYWQuY3N2KCIuLi9kYXRhL2Zvcm1hbGRlaHlkZV9ldm9sdXRpb25fdHJhbnNjcmlwdG9taWNzLnR4dCIsIHNlcD0iXHQiKQ0KY2hlbV9kYXRhX21ldGFCIDwtIHJlYWQuY3N2KCIuLi9kYXRhL2Zvcm1hbGRlaHlkZV9ldm9sdXRpb25fbWV0YWJvbG9taWNzLnR4dCIsIHNlcD0iXHQiKQ0KDQojIyBEYXRhIGZvciBmaWd1cmUNCg0KYW5ub3RhdGlvbl90YWJsZV9maWcgPC0gcmVhZHhsOjpyZWFkX2V4Y2VsKCIuLi9kYXRhL1RhYmxlX1MzX0RFR3NfYW5ub3RhdGlvbnMueGxzeCIpDQpyZW5hbWUoYW5ub3RhdGlvbl90YWJsZV9maWcsICJDT0dfY2F0ZWdvcnkiID0gIkNPRyBjYXRlZ29yeSIsIA0KICAgICAgICJDT0dfY2F0ZWdvcnlfbG9uZyIgPSAiQ09HIGNhdGVnb3J5IGxvbmciLA0KICAgICAgICJDT0dfcHJvY2VzcyIgPSAiQ09HIHByb2Nlc3MiKSAtPiBhbm5vdGF0aW9uX3RhYmxlX2ZpZw0KDQptZXRhYm9sb21pY3NfZGYgPC0gcmVhZC5jc3YoIi4uL2RhdGEvbWV0YWJvbG9taWNzLnR4dCIsIHNlcD0iXHQiLCByb3cubmFtZXMgPSAxKQ0KDQojIGtlZXAgYSB2ZXJzaW9uIG9mIHRoZSBtZXRhYm9sb21pY3MgZGF0YXNldCB3aXRoIGFsbCB0aW1lIHBvaW50cw0KbWV0YWJvbG9taWNzX2RmIC0+IG1ldGFib2xvbWljc19hbGxfdGltZXNfZGYNCm1ldGFib2xvbWljc19hbGxfdGltZXNfZGYgJT4lIHQoKSAlPiUgZGF0YS5mcmFtZSgpIC0+IG1ldGFib2xvbWljc19hbGxfdGltZXNfZGYNCg0KbWV0YWJvbG9taWNzX2FsbF90aW1lc19kZiAlPiUgcmVuYW1lKCJXTl9UMF8xIiA9ICJTNUNfVDBfMSIsIA0KICAgICAgICAgICAgICAgICAgICAgICAgICAgICAgICAgICAgICJXTl9UMF8yIiA9ICJTNUNfVDBfMiIsIA0KICAgICAgICAgICAgICAgICAgICAgICAgICAgICAgICAgICAgICJXTl9UMF8zIiA9ICJTNUNfVDBfMyIsDQogICAgICAgICAgICAgICAgICAgICAgICAgICAgICAgICAgICAgIlNEX1QwXzEiID0gIlMxN0NfVDBfMSIsIA0KICAgICAgICAgICAgICAgICAgICAgICAgICAgICAgICAgICAgICJTRF9UMF8yIiA9ICJTMTdDX1QwXzIiLCANCiAgICAgICAgICAgICAgICAgICAgICAgICAgICAgICAgICAgICAiU0RfVDBfMyIgPSAiUzE3Q19UMF8zIiwNCiAgICAgICAgICAgICAgICAgICAgICAgICAgICAgICAgICAgICAiV05fVEZfMSIgPSAiUzVDX1RGXzEiLCANCiAgICAgICAgICAgICAgICAgICAgICAgICAgICAgICAgICAgICAiV05fVEZfMiIgPSAiUzVDX1RGXzIiLCANCiAgICAgICAgICAgICAgICAgICAgICAgICAgICAgICAgICAgICAiV05fVEZfMyIgPSAiUzVDX1RGXzMiLA0KICAgICAgICAgICAgICAgICAgICAgICAgICAgICAgICAgICAgICJTRF9URl8xIiA9ICJTMTdDX1RGXzEiLCANCiAgICAgICAgICAgICAgICAgICAgICAgICAgICAgICAgICAgICAiU0RfVEZfMiIgPSAiUzE3Q19URl8yIiwgDQogICAgICAgICAgICAgICAgICAgICAgICAgICAgICAgICAgICAgIlNEX1RGXzMiID0gIlMxN0NfVEZfMyIpIC0+IG1ldGFib2xvbWljc19hbGxfdGltZXNfZGYNCg0KbWV0YWJvbG9taWNzX2FsbF90aW1lc19kZiA8LSBtZXRhYm9sb21pY3NfYWxsX3RpbWVzX2RmWyxjKCJXTl9UMF8xIiwgIldOX1QwXzIiLCAiV05fVDBfMyIsICJTRF9UMF8xIiwgIlNEX1QwXzIiLCAiU0RfVDBfMyIsDQogICAgICAgICAgICAgICAgICAgICAgICAgICAgICAgICAgICAgICAgICAgICAgICAgICAgICAgICAgIldOX1RGXzEiLCAiV05fVEZfMiIsICJXTl9URl8zIiwgIlNEX1RGXzEiLCAiU0RfVEZfMiIsICJTRF9URl8zIildDQoNCm1ldGFkYXRhX3RhYmxlX21ldGFCX2FsbF90aW1lcyA8LSByZWFkLmNzdigiLi4vZGF0YS9tZXRhZGF0YV9tZXRhQi50eHQiLCBzZXAgPSAiXHQiLCByb3cubmFtZXMgPSAibmFtZSIpICMgcm93Lm5hbWVzID0gInNhbXBsZSINCg0KIyBLZWVwIGZpbmFsIHRpbWUgcG9pbnRzIG9ubHksIGxpa2UgZm9yIG1ldGF0cmFuc2NyaXB0b21pY3MNCm1ldGFib2xvbWljc19kZiAgJT4lIHQoKSAlPiUgDQogIGRhdGEuZnJhbWUoKSAlPiUgc2VsZWN0KHN0YXJ0c193aXRoKGMoIlMxN0NfVEZfIiwgIlM1Q19URl8iKSkpIC0+IG1ldGFib2xvbWljc19kZg0KDQoNCm1ldGFib2xvbWljc19kZiAlPiUgcmVuYW1lKCJXTl9URl8xIiA9ICJTNUNfVEZfMSIsIA0KICAgICAgICAgICAgICAgICAgICAgICAgICAgIldOX1RGXzIiID0gIlM1Q19URl8yIiwgDQogICAgICAgICAgICAgICAgICAgICAgICAgICAiV05fVEZfMyIgPSAiUzVDX1RGXzMiLA0KICAgICAgICAgICAgICAgICAgICAgICAgICAgIlNEX1RGXzEiID0gIlMxN0NfVEZfMSIsIA0KICAgICAgICAgICAgICAgICAgICAgICAgICAgIlNEX1RGXzIiID0gIlMxN0NfVEZfMiIsIA0KICAgICAgICAgICAgICAgICAgICAgICAgICAgIlNEX1RGXzMiID0gIlMxN0NfVEZfMyIpIC0+IG1ldGFib2xvbWljc19kZg0KDQptZXRhYm9sb21pY3NfYm94X3Bsb3RfZGYgPC0gcmVhZHhsOjpyZWFkX2V4Y2VsKCIuLi9yZXN1bHRzL21ldGFib2xvbWljc19hbm5vdGF0aW9uX2JveHBsb3QueGxzeCIpDQoNCm1ldGFib2xvbWljc19hbm5vdGF0aW9ucyA8LSByZWFkeGw6OnJlYWRfZXhjZWwoIi4uL3Jlc3VsdHMvVGFibGVfUzJfTWV0YWJvbGl0ZXNfaWRlbnRpZmllZC54bHN4Iiwgbl9tYXggPSAyNSkNCmBgYA0KDQojIyMjIyBSZW1vdmFsIG9mIGJsYW5rIHNhbXBsZXMNCg0KYGBge3IgdGFraW5nX291dF9ibGt9DQoNCmNvdW50c190YWJsZSA8LSBjb3VudHNfdGFibGVbLGMoIldOX1RGXzEiLCAiV05fVEZfMiIsICJXTl9URl8zIiwgIlNEX1RGXzEiLCAiU0RfVEZfMiIsICJTRF9URl8zIildDQptZXRhZGF0YV90YWJsZSA8LSBtZXRhZGF0YV90YWJsZVtjKCJXTl9URl8xIiwgIldOX1RGXzIiLCAiV05fVEZfMyIsICJTRF9URl8xIiwgIlNEX1RGXzIiLCAiU0RfVEZfMyIpLF0NCiAgDQpgYGANCg0KIyMgQ2hlbWljYWwgcHJvcGVydGllcyBvZiB0aGUgc2FtcGxlcyB7LnRhYnNldH0NCg0KIyMjIEZvcm1hbGRlaHlkZSBldm9sdXRpb24NCg0KYGBge3IgY2hlbV9wbG90c19mb3JtYWxkZWh5ZGV9DQoNCmNoZW1fZGF0YV9tZXRhVCRleHBlcmltZW50IDwtICJtZXRhVCINCmNoZW1fZGF0YV9tZXRhQiRleHBlcmltZW50IDwtICJtZXRhQiINCg0KY2hlbV9kYXRhIDwtIHJiaW5kKGNoZW1fZGF0YV9tZXRhVCwgY2hlbV9kYXRhX21ldGFCKQ0KDQoNCg0KIyMgTWV0YXRyYW5zY3JpcHRvbWljcyBkYXRhDQoNCmNoZW1fZGF0YVtjaGVtX2RhdGEkY29sb3IgPT0gIiM4OURERjgiICYgY2hlbV9kYXRhJGV4cGVyaW1lbnQgPT0gIm1ldGFUIixdIC0+IHRtcDENCmdyb3VwX2J5KHRtcDEsIHNhbXBsaW5nX3RpbWUpICU+JSBzdW1tYXJpc2UoZm9ybWFsZGVoeWRlX21lYW49bWVhbihmb3JtYWxkZWh5ZGVfcmF0aW9fdG9faW5pdGlhbCksIA0KICAgICAgICAgICAgICAgICAgICAgICAgICAgICAgICAgICAgICAgICAgICBmb3JtYWxkZWh5ZGVfc2Q9c2QoZm9ybWFsZGVoeWRlX3JhdGlvX3RvX2luaXRpYWwpKSAtPiB0bXAxDQp0bXAxJGNvbG9yIDwtICIjODlEREY4Ig0KdG1wMSRjb25kaXRpb24gPC0gIldOIg0KdG1wMSRjYXRlZ29yeSA8LSAiYmlvdGljX1dOIg0KdG1wMSRleHBlcmltZW50IDwtICJtZXRhVCINCg0KY2hlbV9kYXRhW2NoZW1fZGF0YSRjb2xvciA9PSAiI0Y4QUQxOCIgJiBjaGVtX2RhdGEkZXhwZXJpbWVudCA9PSAibWV0YVQiLF0gLT4gdG1wMg0KZ3JvdXBfYnkodG1wMiwgc2FtcGxpbmdfdGltZSkgJT4lIHN1bW1hcmlzZShmb3JtYWxkZWh5ZGVfbWVhbj1tZWFuKGZvcm1hbGRlaHlkZV9yYXRpb190b19pbml0aWFsKSwgDQogICAgICAgICAgICAgICAgICAgICAgICAgICAgICAgICAgICAgICAgICAgIGZvcm1hbGRlaHlkZV9zZD1zZChmb3JtYWxkZWh5ZGVfcmF0aW9fdG9faW5pdGlhbCkpIC0+IHRtcDINCnRtcDIkY29sb3IgPC0gIiNGOEFEMTgiDQp0bXAyJGNvbmRpdGlvbiA8LSAiU0QiDQp0bXAyJGNhdGVnb3J5IDwtICJiaW90aWNfU0QiDQp0bXAyJGV4cGVyaW1lbnQgPC0gIm1ldGFUIg0KDQpjaGVtX2RhdGFbY2hlbV9kYXRhJGNvbG9yID09ICIjQTZDMUNBIiAmIGNoZW1fZGF0YSRleHBlcmltZW50ID09ICJtZXRhVCIsXSAtPiB0bXAzDQpncm91cF9ieSh0bXAzLCBzYW1wbGluZ190aW1lKSAlPiUgc3VtbWFyaXNlKGZvcm1hbGRlaHlkZV9tZWFuPW1lYW4oZm9ybWFsZGVoeWRlX3JhdGlvX3RvX2luaXRpYWwpLCANCiAgICAgICAgICAgICAgICAgICAgICAgICAgICAgICAgICAgICAgICAgICAgZm9ybWFsZGVoeWRlX3NkPXNkKGZvcm1hbGRlaHlkZV9yYXRpb190b19pbml0aWFsKSkgLT4gdG1wMw0KdG1wMyRjb2xvciA8LSAiI0E2QzFDQSINCnRtcDMkY29uZGl0aW9uIDwtICJXTiINCnRtcDMkY2F0ZWdvcnkgPC0gImFiaW90aWNfV04iDQp0bXAzJGV4cGVyaW1lbnQgPC0gIm1ldGFUIg0KDQpjaGVtX2RhdGFbY2hlbV9kYXRhJGNvbG9yID09ICIjQzRCNjlCIiAmIGNoZW1fZGF0YSRleHBlcmltZW50ID09ICJtZXRhVCIsXSAtPiB0bXA0DQpncm91cF9ieSh0bXA0LCBzYW1wbGluZ190aW1lKSAlPiUgc3VtbWFyaXNlKGZvcm1hbGRlaHlkZV9tZWFuPW1lYW4oZm9ybWFsZGVoeWRlX3JhdGlvX3RvX2luaXRpYWwpLCANCiAgICAgICAgICAgICAgICAgICAgICAgICAgICAgICAgICAgICAgICAgICAgZm9ybWFsZGVoeWRlX3NkPXNkKGZvcm1hbGRlaHlkZV9yYXRpb190b19pbml0aWFsKSkgLT4gdG1wNA0KdG1wNCRjb2xvciA8LSAiI0M0QjY5QiINCnRtcDQkY29uZGl0aW9uIDwtICJTRCINCnRtcDQkY2F0ZWdvcnkgPC0gImFiaW90aWNfU0QiDQp0bXA0JGV4cGVyaW1lbnQgPC0gIm1ldGFUIg0KDQojIyBNZXRhYm9sb21pY3MgZGF0YQ0KDQpjaGVtX2RhdGFbY2hlbV9kYXRhJGNvbG9yID09ICIjODlEREY4IiAmIGNoZW1fZGF0YSRleHBlcmltZW50ID09ICJtZXRhQiIsXSAtPiB0bXA1DQpncm91cF9ieSh0bXA1LCBzYW1wbGluZ190aW1lKSAlPiUgc3VtbWFyaXNlKGZvcm1hbGRlaHlkZV9tZWFuPW1lYW4oZm9ybWFsZGVoeWRlX3JhdGlvX3RvX2luaXRpYWwpLCANCiAgICAgICAgICAgICAgICAgICAgICAgICAgICAgICAgICAgICAgICAgICAgZm9ybWFsZGVoeWRlX3NkPXNkKGZvcm1hbGRlaHlkZV9yYXRpb190b19pbml0aWFsKSkgLT4gdG1wNQ0KdG1wNSRjb2xvciA8LSAiIzg5RERGOCINCnRtcDUkY29uZGl0aW9uIDwtICJXTiINCnRtcDUkY2F0ZWdvcnkgPC0gImJpb3RpY19XTiINCnRtcDUkZXhwZXJpbWVudCA8LSAibWV0YUIiDQoNCmNoZW1fZGF0YVtjaGVtX2RhdGEkY29sb3IgPT0gIiNGOEFEMTgiICYgY2hlbV9kYXRhJGV4cGVyaW1lbnQgPT0gIm1ldGFCIixdIC0+IHRtcDYNCmdyb3VwX2J5KHRtcDYsIHNhbXBsaW5nX3RpbWUpICU+JSBzdW1tYXJpc2UoZm9ybWFsZGVoeWRlX21lYW49bWVhbihmb3JtYWxkZWh5ZGVfcmF0aW9fdG9faW5pdGlhbCksIA0KICAgICAgICAgICAgICAgICAgICAgICAgICAgICAgICAgICAgICAgICAgICBmb3JtYWxkZWh5ZGVfc2Q9c2QoZm9ybWFsZGVoeWRlX3JhdGlvX3RvX2luaXRpYWwpKSAtPiB0bXA2DQp0bXA2JGNvbG9yIDwtICIjRjhBRDE4Ig0KdG1wNiRjb25kaXRpb24gPC0gIlNEIg0KdG1wNiRjYXRlZ29yeSA8LSAiYmlvdGljX1NEIg0KdG1wNiRleHBlcmltZW50IDwtICJtZXRhQiINCg0KY2hlbV9kYXRhW2NoZW1fZGF0YSRjb2xvciA9PSAiI0E2QzFDQSIgJiBjaGVtX2RhdGEkZXhwZXJpbWVudCA9PSAibWV0YUIiLF0gLT4gdG1wNw0KZ3JvdXBfYnkodG1wNywgc2FtcGxpbmdfdGltZSkgJT4lIHN1bW1hcmlzZShmb3JtYWxkZWh5ZGVfbWVhbj1tZWFuKGZvcm1hbGRlaHlkZV9yYXRpb190b19pbml0aWFsKSwgDQogICAgICAgICAgICAgICAgICAgICAgICAgICAgICAgICAgICAgICAgICAgIGZvcm1hbGRlaHlkZV9zZD1zZChmb3JtYWxkZWh5ZGVfcmF0aW9fdG9faW5pdGlhbCkpIC0+IHRtcDcNCnRtcDckY29sb3IgPC0gIiNBNkMxQ0EiDQp0bXA3JGNvbmRpdGlvbiA8LSAiV04iDQp0bXA3JGNhdGVnb3J5IDwtICJhYmlvdGljX1dOIg0KdG1wNyRleHBlcmltZW50IDwtICJtZXRhQiINCg0KY2hlbV9kYXRhW2NoZW1fZGF0YSRjb2xvciA9PSAiI0M0QjY5QiIgJiBjaGVtX2RhdGEkZXhwZXJpbWVudCA9PSAibWV0YUIiLF0gLT4gdG1wOA0KZ3JvdXBfYnkodG1wOCwgc2FtcGxpbmdfdGltZSkgJT4lIHN1bW1hcmlzZShmb3JtYWxkZWh5ZGVfbWVhbj1tZWFuKGZvcm1hbGRlaHlkZV9yYXRpb190b19pbml0aWFsKSwgDQogICAgICAgICAgICAgICAgICAgICAgICAgICAgICAgICAgICAgICAgICAgIGZvcm1hbGRlaHlkZV9zZD1zZChmb3JtYWxkZWh5ZGVfcmF0aW9fdG9faW5pdGlhbCkpIC0+IHRtcDgNCnRtcDgkY29sb3IgPC0gIiNDNEI2OUIiDQp0bXA4JGNvbmRpdGlvbiA8LSAiU0QiDQp0bXA4JGNhdGVnb3J5IDwtICJhYmlvdGljX1NEIg0KdG1wOCRleHBlcmltZW50IDwtICJtZXRhQiINCg0KDQojIyBNZXJnZSBkYXRhDQoNCmxpbmVfZGF0YSA8LSByYmluZCh0bXAxLCB0bXAyLCB0bXAzLCB0bXA0LCB0bXA1LCB0bXA2LCB0bXA3LCB0bXA4KQ0Kcm0odG1wMSwgdG1wMiwgdG1wMywgdG1wNCwgdG1wNSwgdG1wNiwgdG1wNywgdG1wOCkNCg0KDQojIyBUZXN0IGRpZmZlcmVuY2UgYmV0d2VlbiBiaW90aWMgYW5kIGFiaW90aWMNCg0KDQprcnVza2FsX2Zvcm1hbGRlaHlkZSA8LSBjKCIiLA0KICAgICAgICAgICAgICAgICAgICAgICAgICB0cnkoaWYoa3J1c2thbC50ZXN0KHggPSBjKGNoZW1fZGF0YV9tZXRhQlsoY2hlbV9kYXRhX21ldGFCJHNhbXBsaW5nX3RpbWUgPT0gMSAmDQogICAgICAgICAgICAgICAgICAgICAgICAgICAgICAgICAgICAgICAgICAgICAgICAgICAgICAgICAgICAgICAgICAgICBjaGVtX2RhdGFfbWV0YUIkY29sb3IgPT0gIiNGOEFEMTgiKSwNCiAgICAgICAgICAgICAgICAgICAgICAgICAgICAgICAgICAgICAgICAgICAgICAgICAgICAgICAgICAgICAgICAgICAgImZvcm1hbGRlaHlkZV9yYXRpb190b19pbml0aWFsIl0sDQogICAgICAgICAgICAgICAgICAgICAgICAgICAgICAgICAgICAgICAgICAgICAgICAgICAgY2hlbV9kYXRhX21ldGFCWyhjaGVtX2RhdGFfbWV0YUIkc2FtcGxpbmdfdGltZSA9PSAxICYNCiAgICAgICAgICAgICAgICAgICAgICAgICAgICAgICAgICAgICAgICAgICAgICAgICAgICAgICAgICAgICAgICAgICAgIGNoZW1fZGF0YV9tZXRhQiRjb2xvciA9PSAiI0M0QjY5QiIpLA0KICAgICAgICAgICAgICAgICAgICAgICAgICAgICAgICAgICAgICAgICAgICAgICAgICAgICAgICAgICAgICAgICAgICAiZm9ybWFsZGVoeWRlX3JhdGlvX3RvX2luaXRpYWwiXSksDQogICAgICAgICAgICAgICAgICAgICAgICAgICAgICAgICAgICAgICAgICAgICAgZyA9IGMocmVwKCJiaW90aWNfU0QiLCAzKSwgcmVwKCJhYmlvdGljX1NEIiwgMykpDQogICAgICAgICAgICAgICAgICAgICAgICAgIClbWyJwLnZhbHVlIl1dIDwgMC4wNSl7IioifWVsc2V7IiJ9LCBzaWxlbnQgPSBUUlVFKSAsDQogICAgICAgICAgICAgICAgICAgICAgICAgIHRyeShpZihrcnVza2FsLnRlc3QoeCA9IGMoY2hlbV9kYXRhX21ldGFCWyhjaGVtX2RhdGFfbWV0YUIkc2FtcGxpbmdfdGltZSA9PSAyICYNCiAgICAgICAgICAgICAgICAgICAgICAgICAgICAgICAgICAgICAgICAgICAgICAgICAgICAgICAgICAgICAgICAgICAgIGNoZW1fZGF0YV9tZXRhQiRjb2xvciA9PSAiI0Y4QUQxOCIpLA0KICAgICAgICAgICAgICAgICAgICAgICAgICAgICAgICAgICAgICAgICAgICAgICAgICAgICAgICAgICAgICAgICAgICAiZm9ybWFsZGVoeWRlX3JhdGlvX3RvX2luaXRpYWwiXSwNCiAgICAgICAgICAgICAgICAgICAgICAgICAgICAgICAgICAgICAgICAgICAgICAgICAgICBjaGVtX2RhdGFfbWV0YUJbKGNoZW1fZGF0YV9tZXRhQiRzYW1wbGluZ190aW1lID09IDIgJg0KICAgICAgICAgICAgICAgICAgICAgICAgICAgICAgICAgICAgICAgICAgICAgICAgICAgICAgICAgICAgICAgICAgICAgY2hlbV9kYXRhX21ldGFCJGNvbG9yID09ICIjQzRCNjlCIiksDQogICAgICAgICAgICAgICAgICAgICAgICAgICAgICAgICAgICAgICAgICAgICAgICAgICAgICAgICAgICAgICAgICAgICJmb3JtYWxkZWh5ZGVfcmF0aW9fdG9faW5pdGlhbCJdKSwNCiAgICAgICAgICAgICAgICAgICAgICAgICAgICAgICAgICAgICAgICAgICAgICBnID0gYyhyZXAoImJpb3RpY19TRCIsIDMpLCByZXAoImFiaW90aWNfU0QiLCAzKSkNCiAgICAgICAgICAgICAgICAgICAgICAgICAgKVtbInAudmFsdWUiXV0gPCAwLjA1KXsiKiJ9ZWxzZXsiIn0sIHNpbGVudCA9IFRSVUUpLA0KICAgICAgICAgICAgICAgICAgICAgICAgICB0cnkoaWYoa3J1c2thbC50ZXN0KHggPSBjKGNoZW1fZGF0YV9tZXRhQlsoY2hlbV9kYXRhX21ldGFCJHNhbXBsaW5nX3RpbWUgPT0gMyAmDQogICAgICAgICAgICAgICAgICAgICAgICAgICAgICAgICAgICAgICAgICAgICAgICAgICAgICAgICAgICAgICAgICAgICBjaGVtX2RhdGFfbWV0YUIkY29sb3IgPT0gIiNGOEFEMTgiKSwNCiAgICAgICAgICAgICAgICAgICAgICAgICAgICAgICAgICAgICAgICAgICAgICAgICAgICAgICAgICAgICAgICAgICAgImZvcm1hbGRlaHlkZV9yYXRpb190b19pbml0aWFsIl0sDQogICAgICAgICAgICAgICAgICAgICAgICAgICAgICAgICAgICAgICAgICAgICAgICAgICAgY2hlbV9kYXRhX21ldGFCWyhjaGVtX2RhdGFfbWV0YUIkc2FtcGxpbmdfdGltZSA9PSAzICYNCiAgICAgICAgICAgICAgICAgICAgICAgICAgICAgICAgICAgICAgICAgICAgICAgICAgICAgICAgICAgICAgICAgICAgIGNoZW1fZGF0YV9tZXRhQiRjb2xvciA9PSAiI0M0QjY5QiIpLA0KICAgICAgICAgICAgICAgICAgICAgICAgICAgICAgICAgICAgICAgICAgICAgICAgICAgICAgICAgICAgICAgICAgICAiZm9ybWFsZGVoeWRlX3JhdGlvX3RvX2luaXRpYWwiXSksDQogICAgICAgICAgICAgICAgICAgICAgICAgICAgICAgICAgICAgICAgICAgICAgZyA9IGMocmVwKCJiaW90aWNfU0QiLCAzKSwgcmVwKCJhYmlvdGljX1NEIiwgMykpDQogICAgICAgICAgICAgICAgICAgICAgICAgIClbWyJwLnZhbHVlIl1dIDwgMC4wNSl7IioifWVsc2V7IiJ9LCBzaWxlbnQgPSBUUlVFKSwNCiAgICAgICAgICAgICAgICAgICAgICAgICAgIiIsDQogICAgICAgICAgICAgICAgICAgICAgICAgIHRyeShpZihrcnVza2FsLnRlc3QoeCA9IGMoY2hlbV9kYXRhX21ldGFUWyhjaGVtX2RhdGFfbWV0YVQkc2FtcGxpbmdfdGltZSA9PSAxICYNCiAgICAgICAgICAgICAgICAgICAgICAgICAgICAgICAgICAgICAgICAgICAgICAgICAgICAgICAgICAgICAgICAgICAgIGNoZW1fZGF0YV9tZXRhVCRjb2xvciA9PSAiI0Y4QUQxOCIpLA0KICAgICAgICAgICAgICAgICAgICAgICAgICAgICAgICAgICAgICAgICAgICAgICAgICAgICAgICAgICAgICAgICAgICAiZm9ybWFsZGVoeWRlX3JhdGlvX3RvX2luaXRpYWwiXSwNCiAgICAgICAgICAgICAgICAgICAgICAgICAgICAgICAgICAgICAgICAgICAgICAgICAgICBjaGVtX2RhdGFfbWV0YVRbKGNoZW1fZGF0YV9tZXRhVCRzYW1wbGluZ190aW1lID09IDEgJg0KICAgICAgICAgICAgICAgICAgICAgICAgICAgICAgICAgICAgICAgICAgICAgICAgICAgICAgICAgICAgICAgICAgICAgY2hlbV9kYXRhX21ldGFUJGNvbG9yID09ICIjQzRCNjlCIiksDQogICAgICAgICAgICAgICAgICAgICAgICAgICAgICAgICAgICAgICAgICAgICAgICAgICAgICAgICAgICAgICAgICAgICJmb3JtYWxkZWh5ZGVfcmF0aW9fdG9faW5pdGlhbCJdKSwNCiAgICAgICAgICAgICAgICAgICAgICAgICAgICAgICAgICAgICAgICAgICAgICBnID0gYyhyZXAoImJpb3RpY19TRCIsIDMpLCByZXAoImFiaW90aWNfU0QiLCAzKSkNCiAgICAgICAgICAgICAgICAgICAgICAgICAgKVtbInAudmFsdWUiXV0gPCAwLjA1KXsiKiJ9ZWxzZXsiIn0sIHNpbGVudCA9IFRSVUUpICwNCiAgICAgICAgICAgICAgICAgICAgICAgICAgdHJ5KGlmKGtydXNrYWwudGVzdCh4ID0gYyhjaGVtX2RhdGFfbWV0YVRbKGNoZW1fZGF0YV9tZXRhVCRzYW1wbGluZ190aW1lID09IDIgJg0KICAgICAgICAgICAgICAgICAgICAgICAgICAgICAgICAgICAgICAgICAgICAgICAgICAgICAgICAgICAgICAgICAgICAgY2hlbV9kYXRhX21ldGFUJGNvbG9yID09ICIjRjhBRDE4IiksDQogICAgICAgICAgICAgICAgICAgICAgICAgICAgICAgICAgICAgICAgICAgICAgICAgICAgICAgICAgICAgICAgICAgICJmb3JtYWxkZWh5ZGVfcmF0aW9fdG9faW5pdGlhbCJdLA0KICAgICAgICAgICAgICAgICAgICAgICAgICAgICAgICAgICAgICAgICAgICAgICAgICAgIGNoZW1fZGF0YV9tZXRhVFsoY2hlbV9kYXRhX21ldGFUJHNhbXBsaW5nX3RpbWUgPT0gMiAmDQogICAgICAgICAgICAgICAgICAgICAgICAgICAgICAgICAgICAgICAgICAgICAgICAgICAgICAgICAgICAgICAgICAgICBjaGVtX2RhdGFfbWV0YVQkY29sb3IgPT0gIiNDNEI2OUIiKSwNCiAgICAgICAgICAgICAgICAgICAgICAgICAgICAgICAgICAgICAgICAgICAgICAgICAgICAgICAgICAgICAgICAgICAgImZvcm1hbGRlaHlkZV9yYXRpb190b19pbml0aWFsIl0pLA0KICAgICAgICAgICAgICAgICAgICAgICAgICAgICAgICAgICAgICAgICAgICAgIGcgPSBjKHJlcCgiYmlvdGljX1NEIiwgMyksIHJlcCgiYWJpb3RpY19TRCIsIDMpKQ0KICAgICAgICAgICAgICAgICAgICAgICAgICApW1sicC52YWx1ZSJdXSA8IDAuMDUpeyIqIn1lbHNleyIifSwgc2lsZW50ID0gVFJVRSksDQogICAgICAgICAgICAgICAgICAgICAgICAgIHRyeShpZihrcnVza2FsLnRlc3QoeCA9IGMoY2hlbV9kYXRhX21ldGFUWyhjaGVtX2RhdGFfbWV0YVQkc2FtcGxpbmdfdGltZSA9PSAzICYNCiAgICAgICAgICAgICAgICAgICAgICAgICAgICAgICAgICAgICAgICAgICAgICAgICAgICAgICAgICAgICAgICAgICAgIGNoZW1fZGF0YV9tZXRhVCRjb2xvciA9PSAiI0Y4QUQxOCIpLA0KICAgICAgICAgICAgICAgICAgICAgICAgICAgICAgICAgICAgICAgICAgICAgICAgICAgICAgICAgICAgICAgICAgICAiZm9ybWFsZGVoeWRlX3JhdGlvX3RvX2luaXRpYWwiXSwNCiAgICAgICAgICAgICAgICAgICAgICAgICAgICAgICAgICAgICAgICAgICAgICAgICAgICBjaGVtX2RhdGFfbWV0YVRbKGNoZW1fZGF0YV9tZXRhVCRzYW1wbGluZ190aW1lID09IDMgJg0KICAgICAgICAgICAgICAgICAgICAgICAgICAgICAgICAgICAgICAgICAgICAgICAgICAgICAgICAgICAgICAgICAgICAgY2hlbV9kYXRhX21ldGFUJGNvbG9yID09ICIjQzRCNjlCIiksDQogICAgICAgICAgICAgICAgICAgICAgICAgICAgICAgICAgICAgICAgICAgICAgICAgICAgICAgICAgICAgICAgICAgICJmb3JtYWxkZWh5ZGVfcmF0aW9fdG9faW5pdGlhbCJdKSwNCiAgICAgICAgICAgICAgICAgICAgICAgICAgICAgICAgICAgICAgICAgICAgICBnID0gYyhyZXAoImJpb3RpY19TRCIsIDMpLCByZXAoImFiaW90aWNfU0QiLCAzKSkNCiAgICAgICAgICAgICAgICAgICAgICAgICAgKVtbInAudmFsdWUiXV0gPCAwLjA1KXsiKiJ9ZWxzZXsiIn0sIHNpbGVudCA9IFRSVUUpLA0KICAgICAgICAgICAgICAgICAgICAgICAgICB0cnkoaWYoa3J1c2thbC50ZXN0KHggPSBjKGNoZW1fZGF0YV9tZXRhVFsoY2hlbV9kYXRhX21ldGFUJHNhbXBsaW5nX3RpbWUgPT0gNSAmDQogICAgICAgICAgICAgICAgICAgICAgICAgICAgICAgICAgICAgICAgICAgICAgICAgICAgICAgICAgICAgICAgICAgICBjaGVtX2RhdGFfbWV0YVQkY29sb3IgPT0gIiNGOEFEMTgiKSwNCiAgICAgICAgICAgICAgICAgICAgICAgICAgICAgICAgICAgICAgICAgICAgICAgICAgICAgICAgICAgICAgICAgICAgImZvcm1hbGRlaHlkZV9yYXRpb190b19pbml0aWFsIl0sDQogICAgICAgICAgICAgICAgICAgICAgICAgICAgICAgICAgICAgICAgICAgICAgICAgICAgY2hlbV9kYXRhX21ldGFUWyhjaGVtX2RhdGFfbWV0YVQkc2FtcGxpbmdfdGltZSA9PSA1ICYNCiAgICAgICAgICAgICAgICAgICAgICAgICAgICAgICAgICAgICAgICAgICAgICAgICAgICAgICAgICAgICAgICAgICAgIGNoZW1fZGF0YV9tZXRhVCRjb2xvciA9PSAiI0M0QjY5QiIpLA0KICAgICAgICAgICAgICAgICAgICAgICAgICAgICAgICAgICAgICAgICAgICAgICAgICAgICAgICAgICAgICAgICAgICAiZm9ybWFsZGVoeWRlX3JhdGlvX3RvX2luaXRpYWwiXSksDQogICAgICAgICAgICAgICAgICAgICAgICAgICAgICAgICAgICAgICAgICAgICAgZyA9IGMocmVwKCJiaW90aWNfU0QiLCAzKSwgcmVwKCJhYmlvdGljX1NEIiwgMykpDQogICAgICAgICAgICAgICAgICAgICAgICAgIClbWyJwLnZhbHVlIl1dIDwgMC4wNSl7IioifWVsc2V7IiJ9LCBzaWxlbnQgPSBUUlVFKSwNCiAgICAgICAgICAgICAgICAgICAgICAgICAgIiIsDQogICAgICAgICAgICAgICAgICAgICAgICAgIHRyeShpZihrcnVza2FsLnRlc3QoeCA9IGMoY2hlbV9kYXRhX21ldGFCWyhjaGVtX2RhdGFfbWV0YUIkc2FtcGxpbmdfdGltZSA9PSAxICYNCiAgICAgICAgICAgICAgICAgICAgICAgICAgICAgICAgICAgICAgICAgICAgICAgICAgICAgICAgICAgICAgICAgICAgIGNoZW1fZGF0YV9tZXRhQiRjb2xvciA9PSAiIzg5RERGOCIpLA0KICAgICAgICAgICAgICAgICAgICAgICAgICAgICAgICAgICAgICAgICAgICAgICAgICAgICAgICAgICAgICAgICAgICAiZm9ybWFsZGVoeWRlX3JhdGlvX3RvX2luaXRpYWwiXSwNCiAgICAgICAgICAgICAgICAgICAgICAgICAgICAgICAgICAgICAgICAgICAgICAgICAgICBjaGVtX2RhdGFfbWV0YUJbKGNoZW1fZGF0YV9tZXRhQiRzYW1wbGluZ190aW1lID09IDEgJg0KICAgICAgICAgICAgICAgICAgICAgICAgICAgICAgICAgICAgICAgICAgICAgICAgICAgICAgICAgICAgICAgICAgICAgY2hlbV9kYXRhX21ldGFCJGNvbG9yID09ICIjQTZDMUNBIiksDQogICAgICAgICAgICAgICAgICAgICAgICAgICAgICAgICAgICAgICAgICAgICAgICAgICAgICAgICAgICAgICAgICAgICJmb3JtYWxkZWh5ZGVfcmF0aW9fdG9faW5pdGlhbCJdKSwNCiAgICAgICAgICAgICAgICAgICAgICAgICAgICAgICAgICAgICAgICAgICAgICBnID0gYyhyZXAoImJpb3RpY19XTiIsIDMpLCByZXAoImFiaW90aWNfV04iLCAzKSkNCiAgICAgICAgICAgICAgICAgICAgICAgICAgKVtbInAudmFsdWUiXV0gPCAwLjA1KXsiKiJ9ZWxzZXsiIn0sIHNpbGVudCA9IFRSVUUpICwNCiAgICAgICAgICAgICAgICAgICAgICAgICAgdHJ5KGlmKGtydXNrYWwudGVzdCh4ID0gYyhjaGVtX2RhdGFfbWV0YUJbKGNoZW1fZGF0YV9tZXRhQiRzYW1wbGluZ190aW1lID09IDIgJg0KICAgICAgICAgICAgICAgICAgICAgICAgICAgICAgICAgICAgICAgICAgICAgICAgICAgICAgICAgICAgICAgICAgICAgY2hlbV9kYXRhX21ldGFCJGNvbG9yID09ICIjODlEREY4IiksDQogICAgICAgICAgICAgICAgICAgICAgICAgICAgICAgICAgICAgICAgICAgICAgICAgICAgICAgICAgICAgICAgICAgICJmb3JtYWxkZWh5ZGVfcmF0aW9fdG9faW5pdGlhbCJdLA0KICAgICAgICAgICAgICAgICAgICAgICAgICAgICAgICAgICAgICAgICAgICAgICAgICAgIGNoZW1fZGF0YV9tZXRhQlsoY2hlbV9kYXRhX21ldGFCJHNhbXBsaW5nX3RpbWUgPT0gMiAmDQogICAgICAgICAgICAgICAgICAgICAgICAgICAgICAgICAgICAgICAgICAgICAgICAgICAgICAgICAgICAgICAgICAgICBjaGVtX2RhdGFfbWV0YUIkY29sb3IgPT0gIiNBNkMxQ0EiKSwNCiAgICAgICAgICAgICAgICAgICAgICAgICAgICAgICAgICAgICAgICAgICAgICAgICAgICAgICAgICAgICAgICAgICAgImZvcm1hbGRlaHlkZV9yYXRpb190b19pbml0aWFsIl0pLA0KICAgICAgICAgICAgICAgICAgICAgICAgICAgICAgICAgICAgICAgICAgICAgIGcgPSBjKHJlcCgiYmlvdGljX1dOIiwgMyksIHJlcCgiYWJpb3RpY19XTiIsIDMpKQ0KICAgICAgICAgICAgICAgICAgICAgICAgICApW1sicC52YWx1ZSJdXSA8IDAuMDUpeyIqIn1lbHNleyIifSwgc2lsZW50ID0gVFJVRSksDQogICAgICAgICAgICAgICAgICAgICAgICAgIHRyeShpZihrcnVza2FsLnRlc3QoeCA9IGMoY2hlbV9kYXRhX21ldGFCWyhjaGVtX2RhdGFfbWV0YUIkc2FtcGxpbmdfdGltZSA9PSAzICYNCiAgICAgICAgICAgICAgICAgICAgICAgICAgICAgICAgICAgICAgICAgICAgICAgICAgICAgICAgICAgICAgICAgICAgIGNoZW1fZGF0YV9tZXRhQiRjb2xvciA9PSAiIzg5RERGOCIpLA0KICAgICAgICAgICAgICAgICAgICAgICAgICAgICAgICAgICAgICAgICAgICAgICAgICAgICAgICAgICAgICAgICAgICAiZm9ybWFsZGVoeWRlX3JhdGlvX3RvX2luaXRpYWwiXSwNCiAgICAgICAgICAgICAgICAgICAgICAgICAgICAgICAgICAgICAgICAgICAgICAgICAgICBjaGVtX2RhdGFfbWV0YUJbKGNoZW1fZGF0YV9tZXRhQiRzYW1wbGluZ190aW1lID09IDMgJg0KICAgICAgICAgICAgICAgICAgICAgICAgICAgICAgICAgICAgICAgICAgICAgICAgICAgICAgICAgICAgICAgICAgICAgY2hlbV9kYXRhX21ldGFCJGNvbG9yID09ICIjQTZDMUNBIiksDQogICAgICAgICAgICAgICAgICAgICAgICAgICAgICAgICAgICAgICAgICAgICAgICAgICAgICAgICAgICAgICAgICAgICJmb3JtYWxkZWh5ZGVfcmF0aW9fdG9faW5pdGlhbCJdKSwNCiAgICAgICAgICAgICAgICAgICAgICAgICAgICAgICAgICAgICAgICAgICAgICBnID0gYyhyZXAoImJpb3RpY19XTiIsIDMpLCByZXAoImFiaW90aWNfV04iLCAzKSkNCiAgICAgICAgICAgICAgICAgICAgICAgICAgKVtbInAudmFsdWUiXV0gPCAwLjA1KXsiKiJ9ZWxzZXsiIn0sIHNpbGVudCA9IFRSVUUpLA0KICAgICAgICAgICAgICAgICAgICAgICAgICAiIiwNCiAgICAgICAgICAgICAgICAgICAgICAgICAgdHJ5KGlmKGtydXNrYWwudGVzdCh4ID0gYyhjaGVtX2RhdGFfbWV0YVRbKGNoZW1fZGF0YV9tZXRhVCRzYW1wbGluZ190aW1lID09IDEgJg0KICAgICAgICAgICAgICAgICAgICAgICAgICAgICAgICAgICAgICAgICAgICAgICAgICAgICAgICAgICAgICAgICAgICAgY2hlbV9kYXRhX21ldGFUJGNvbG9yID09ICIjODlEREY4IiksDQogICAgICAgICAgICAgICAgICAgICAgICAgICAgICAgICAgICAgICAgICAgICAgICAgICAgICAgICAgICAgICAgICAgICJmb3JtYWxkZWh5ZGVfcmF0aW9fdG9faW5pdGlhbCJdLA0KICAgICAgICAgICAgICAgICAgICAgICAgICAgICAgICAgICAgICAgICAgICAgICAgICAgIGNoZW1fZGF0YV9tZXRhVFsoY2hlbV9kYXRhX21ldGFUJHNhbXBsaW5nX3RpbWUgPT0gMSAmDQogICAgICAgICAgICAgICAgICAgICAgICAgICAgICAgICAgICAgICAgICAgICAgICAgICAgICAgICAgICAgICAgICAgICBjaGVtX2RhdGFfbWV0YVQkY29sb3IgPT0gIiNBNkMxQ0EiKSwNCiAgICAgICAgICAgICAgICAgICAgICAgICAgICAgICAgICAgICAgICAgICAgICAgICAgICAgICAgICAgICAgICAgICAgImZvcm1hbGRlaHlkZV9yYXRpb190b19pbml0aWFsIl0pLA0KICAgICAgICAgICAgICAgICAgICAgICAgICAgICAgICAgICAgICAgICAgICAgIGcgPSBjKHJlcCgiYmlvdGljX1dOIiwgMyksIHJlcCgiYWJpb3RpY19XTiIsIDMpKQ0KICAgICAgICAgICAgICAgICAgICAgICAgICApW1sicC52YWx1ZSJdXSA8IDAuMDUpeyIqIn1lbHNleyIifSwgc2lsZW50ID0gVFJVRSkgLA0KICAgICAgICAgICAgICAgICAgICAgICAgICB0cnkoaWYoa3J1c2thbC50ZXN0KHggPSBjKGNoZW1fZGF0YV9tZXRhVFsoY2hlbV9kYXRhX21ldGFUJHNhbXBsaW5nX3RpbWUgPT0gMiAmDQogICAgICAgICAgICAgICAgICAgICAgICAgICAgICAgICAgICAgICAgICAgICAgICAgICAgICAgICAgICAgICAgICAgICBjaGVtX2RhdGFfbWV0YVQkY29sb3IgPT0gIiM4OURERjgiKSwNCiAgICAgICAgICAgICAgICAgICAgICAgICAgICAgICAgICAgICAgICAgICAgICAgICAgICAgICAgICAgICAgICAgICAgImZvcm1hbGRlaHlkZV9yYXRpb190b19pbml0aWFsIl0sDQogICAgICAgICAgICAgICAgICAgICAgICAgICAgICAgICAgICAgICAgICAgICAgICAgICAgY2hlbV9kYXRhX21ldGFUWyhjaGVtX2RhdGFfbWV0YVQkc2FtcGxpbmdfdGltZSA9PSAyICYNCiAgICAgICAgICAgICAgICAgICAgICAgICAgICAgICAgICAgICAgICAgICAgICAgICAgICAgICAgICAgICAgICAgICAgIGNoZW1fZGF0YV9tZXRhVCRjb2xvciA9PSAiI0E2QzFDQSIpLA0KICAgICAgICAgICAgICAgICAgICAgICAgICAgICAgICAgICAgICAgICAgICAgICAgICAgICAgICAgICAgICAgICAgICAiZm9ybWFsZGVoeWRlX3JhdGlvX3RvX2luaXRpYWwiXSksDQogICAgICAgICAgICAgICAgICAgICAgICAgICAgICAgICAgICAgICAgICAgICAgZyA9IGMocmVwKCJiaW90aWNfV04iLCAzKSwgcmVwKCJhYmlvdGljX1dOIiwgMykpDQogICAgICAgICAgICAgICAgICAgICAgICAgIClbWyJwLnZhbHVlIl1dIDwgMC4wNSl7IioifWVsc2V7IiJ9LCBzaWxlbnQgPSBUUlVFKSwNCiAgICAgICAgICAgICAgICAgICAgICAgICAgdHJ5KGlmKGtydXNrYWwudGVzdCh4ID0gYyhjaGVtX2RhdGFfbWV0YVRbKGNoZW1fZGF0YV9tZXRhVCRzYW1wbGluZ190aW1lID09IDMgJg0KICAgICAgICAgICAgICAgICAgICAgICAgICAgICAgICAgICAgICAgICAgICAgICAgICAgICAgICAgICAgICAgICAgICAgY2hlbV9kYXRhX21ldGFUJGNvbG9yID09ICIjODlEREY4IiksDQogICAgICAgICAgICAgICAgICAgICAgICAgICAgICAgICAgICAgICAgICAgICAgICAgICAgICAgICAgICAgICAgICAgICJmb3JtYWxkZWh5ZGVfcmF0aW9fdG9faW5pdGlhbCJdLA0KICAgICAgICAgICAgICAgICAgICAgICAgICAgICAgICAgICAgICAgICAgICAgICAgICAgIGNoZW1fZGF0YV9tZXRhVFsoY2hlbV9kYXRhX21ldGFUJHNhbXBsaW5nX3RpbWUgPT0gMyAmDQogICAgICAgICAgICAgICAgICAgICAgICAgICAgICAgICAgICAgICAgICAgICAgICAgICAgICAgICAgICAgICAgICAgICBjaGVtX2RhdGFfbWV0YVQkY29sb3IgPT0gIiNBNkMxQ0EiKSwNCiAgICAgICAgICAgICAgICAgICAgICAgICAgICAgICAgICAgICAgICAgICAgICAgICAgICAgICAgICAgICAgICAgICAgImZvcm1hbGRlaHlkZV9yYXRpb190b19pbml0aWFsIl0pLA0KICAgICAgICAgICAgICAgICAgICAgICAgICAgICAgICAgICAgICAgICAgICAgIGcgPSBjKHJlcCgiYmlvdGljX1dOIiwgMyksIHJlcCgiYWJpb3RpY19XTiIsIDMpKQ0KICAgICAgICAgICAgICAgICAgICAgICAgICApW1sicC52YWx1ZSJdXSA8IDAuMDUpeyIqIn1lbHNleyIifSwgc2lsZW50ID0gVFJVRSksDQogICAgICAgICAgICAgICAgICAgICAgICAgIHRyeShpZihrcnVza2FsLnRlc3QoeCA9IGMoY2hlbV9kYXRhX21ldGFUWyhjaGVtX2RhdGFfbWV0YVQkc2FtcGxpbmdfdGltZSA9PSA1ICYNCiAgICAgICAgICAgICAgICAgICAgICAgICAgICAgICAgICAgICAgICAgICAgICAgICAgICAgICAgICAgICAgICAgICAgIGNoZW1fZGF0YV9tZXRhVCRjb2xvciA9PSAiIzg5RERGOCIpLA0KICAgICAgICAgICAgICAgICAgICAgICAgICAgICAgICAgICAgICAgICAgICAgICAgICAgICAgICAgICAgICAgICAgICAiZm9ybWFsZGVoeWRlX3JhdGlvX3RvX2luaXRpYWwiXSwNCiAgICAgICAgICAgICAgICAgICAgICAgICAgICAgICAgICAgICAgICAgICAgICAgICAgICBjaGVtX2RhdGFfbWV0YVRbKGNoZW1fZGF0YV9tZXRhVCRzYW1wbGluZ190aW1lID09IDUgJg0KICAgICAgICAgICAgICAgICAgICAgICAgICAgICAgICAgICAgICAgICAgICAgICAgICAgICAgICAgICAgICAgICAgICAgY2hlbV9kYXRhX21ldGFUJGNvbG9yID09ICIjQTZDMUNBIiksDQogICAgICAgICAgICAgICAgICAgICAgICAgICAgICAgICAgICAgICAgICAgICAgICAgICAgICAgICAgICAgICAgICAgICJmb3JtYWxkZWh5ZGVfcmF0aW9fdG9faW5pdGlhbCJdKSwNCiAgICAgICAgICAgICAgICAgICAgICAgICAgICAgICAgICAgICAgICAgICAgICBnID0gYyhyZXAoImJpb3RpY19XTiIsIDMpLCByZXAoImFiaW90aWNfV04iLCAzKSkNCiAgICAgICAgICAgICAgICAgICAgICAgICAgKVtbInAudmFsdWUiXV0gPCAwLjA1KXsiKiJ9ZWxzZXsiIn0sIHNpbGVudCA9IFRSVUUpDQopDQoNCiMjIFRlc3QgZGlmZmVyZW5jZSBiZXR3ZWVuIHRpbWUgMCBhbiB0aGUgb3RoZXIgdGltZSBwb2ludHMNCg0Ka3J1c2thbF9kZiA8LSBkYXRhLmZyYW1lKHRpbWUgPSByZXAoYygwLCAxLCAyLCAzLCAwLCAxLCAyLCAzLCA1KSwgMiksDQogICAgICAgICAgICAgICAgICAgICAgICAgaGVpZ2h0ID0gcmVwKDAsIDE4KSwNCiAgICAgICAgICAgICAgICAgICAgICAgICBleHBlcmltZW50ID0gcmVwKGMocmVwKCJtZXRhQiIsIDQpLCByZXAoIm1ldGFUIiwgNSkpLCAyKSwNCiAgICAgICAgICAgICAgICAgICAgICAgICBjb25kaXRpb24gPSBjKHJlcCgiU0QiLCA5KSwgcmVwKCJXTiIsIDkpKSwNCiAgICAgICAgICAgICAgICAgICAgICAgICBjYXRlZ29yeSA9IGMocmVwKCJjb21tdW5pdHkgU0QiLCA5KSwgcmVwKCJjb21tdW5pdHkgV04iLCA5KSksDQogICAgICAgICAgICAgICAgICAgICAgICAgY29sb3IgPSBjKHJlcCgiI0Y4QUQxOCIsIDkpLCByZXAoIiM4OURERjgiLCA5KSksDQogICAgICAgICAgICAgICAgICAgICAgICAgcHZhbHVlID0ga3J1c2thbF9mb3JtYWxkZWh5ZGUpDQoNCg0Ka3J1c2sgPC0ga3J1c2thbF9kZg0KDQoNCiMjIFNlZ21lbnQgZGF0YSBmb3Igc2FtcGxpbmcgdGltZQ0KDQpzZWdtZW50X2RhdGEgPC0gZGF0YS5mcmFtZSh4ID0gYyg0LCAwLCAzLjUpLCB4ZW5kID0gYyg0LCAwLCAzLjUpLCANCiAgICAgICAgICAgICAgICAgICAgICAgICAgIHkgPSBjKC1JbmYsIC1JbmYsIC1JbmYpLCB5ZW5kID0gYyhJbmYsIEluZiwgSW5mKSwNCiAgICAgICAgICAgICAgICAgICAgICAgICAgIGV4cGVyaW1lbnQgPSBjKCJtZXRhVCIsICJtZXRhQiIsICJtZXRhQiIpKQ0KICANCmNoZW1fZGF0YSAlPiUgZ2dwbG90KCkgKw0KICBnZW9tX3BvaW50KGFlcyh4PXNhbXBsaW5nX3RpbWUsIHk9Zm9ybWFsZGVoeWRlX3JhdGlvX3RvX2luaXRpYWwsIGNvbG9yPWNvbG9yKSkgKw0KICB5bGltKGMoLTAuMSwgMS4yKSkgKw0KICB4bGltKGMoMCwgNSkpICsNCiAgc2NhbGVfY29sb3VyX2lkZW50aXR5KCJDYXRlZ29yeSIsIGxhYmVscyA9IGMoIkFzc2VtYmxhZ2UgV2ludGVyIE5pZ2h0IiwgIkFiaW90aWMgV2ludGVyIE5pZ2h0IiwNCiAgICAgICAgICAgICAgICAgICAgICAgICAgICAgICAgICAgICAgICAgICAgICAgIkFiaW90aWMgU3VtbWVyIERheSIsICJBc3NlbWJsYWdlIFN1bW1lciBEYXkiKSwgDQogICAgICAgICAgICAgICAgICAgICAgICBndWlkZT0ibGVnZW5kIikgKw0KICBzY2FsZV9maWxsX2lkZW50aXR5KCkgKw0KICB5bGFiKGxhYmVsID0gIlJlbGF0aXZlIGZvcm1hbGRlaHlkZSBldm9sdXRpb24iKSArDQogIHhsYWIobGFiZWwgPSAiSW5jdWJhdGlvbiB0aW1lIChoKSIpICsNCiAgZmFjZXRfZ3JpZChyb3dzID0gdmFycyhjb25kaXRpb24pLCBjb2xzID0gdmFycyhleHBlcmltZW50KSwNCiAgICAgICAgICAgICBsYWJlbGxlciA9IGFzX2xhYmVsbGVyKGMoIm1ldGFUIiA9ICJNZXRhdHJhbnNjcmlwdG9taWNzIiwgDQogICAgICAgICAgICAgICAgICAgICAgICAgICAgICAgICAgICAgICJtZXRhQiIgPSAiTWV0YS1tZXRhYm9sb21pY3MiLA0KICAgICAgICAgICAgICAgICAgICAgICAgICAgICAgICAgICAgICAiU0QiID0gIlN1bW1lciBEYXkiLA0KICAgICAgICAgICAgICAgICAgICAgICAgICAgICAgICAgICAgICAiV04iID0gIldpbnRlciBOaWdodCIpKSkgKyANCiAgdGhlbWUobGVnZW5kLnBvc2l0aW9uID0gImJvdHRvbSIsDQogICAgICAgIHBhbmVsLmJhY2tncm91bmQgPSBlbGVtZW50X3JlY3QoZmlsbD0iI2Y0ZjRmNCIpLA0KICAgICAgICBzdHJpcC50ZXh0ID0gZWxlbWVudF90ZXh0KHNpemU9MTIpKSArDQogIA0KICANCiAgZ2VvbV9wYXRoKGRhdGEgPSBsaW5lX2RhdGFbbGluZV9kYXRhJGNhdGVnb3J5ID09ICJiaW90aWNfU0QiLF0sIA0KICAgICAgICAgICAgYWVzKHggPSBzYW1wbGluZ190aW1lLCB5ID0gZm9ybWFsZGVoeWRlX21lYW4sIGNvbG9yID0gY29sb3IpLCANCiAgICAgICAgICAgIGxpbmV3aWR0aCA9IDEpICsgDQogIGdlb21fcmliYm9uKGRhdGEgPSBsaW5lX2RhdGFbbGluZV9kYXRhJGNhdGVnb3J5ID09ICJiaW90aWNfU0QiLF0sIA0KICAgICAgICAgICAgICBhZXMoeCA9IHNhbXBsaW5nX3RpbWUsIA0KICAgICAgICAgICAgICAgICAgeW1pbiA9IGZvcm1hbGRlaHlkZV9tZWFuIC0gZm9ybWFsZGVoeWRlX3NkLA0KICAgICAgICAgICAgICAgICAgeW1heCA9IGZvcm1hbGRlaHlkZV9tZWFuICsgZm9ybWFsZGVoeWRlX3NkLA0KICAgICAgICAgICAgICAgICAgZmlsbCA9IGNvbG9yKSwgYWxwaGE9MC4yNSwgbGluZXdpZHRoPTAuMDUpICsNCiAgDQogIGdlb21fcGF0aChkYXRhID0gbGluZV9kYXRhW2xpbmVfZGF0YSRjYXRlZ29yeSA9PSAiYWJpb3RpY19TRCIsXSwgDQogICAgICAgICAgICBhZXMoeCA9IHNhbXBsaW5nX3RpbWUsIHkgPSBmb3JtYWxkZWh5ZGVfbWVhbiwgY29sb3IgPSBjb2xvciksIA0KICAgICAgICAgICAgbGluZXdpZHRoID0gMSkgKyANCiAgZ2VvbV9yaWJib24oZGF0YSA9IGxpbmVfZGF0YVtsaW5lX2RhdGEkY2F0ZWdvcnkgPT0gImFiaW90aWNfU0QiLF0sIA0KICAgICAgICAgICAgICBhZXMoeCA9IHNhbXBsaW5nX3RpbWUsIA0KICAgICAgICAgICAgICAgICAgeW1pbiA9IGZvcm1hbGRlaHlkZV9tZWFuIC0gZm9ybWFsZGVoeWRlX3NkLA0KICAgICAgICAgICAgICAgICAgeW1heCA9IGZvcm1hbGRlaHlkZV9tZWFuICsgZm9ybWFsZGVoeWRlX3NkLA0KICAgICAgICAgICAgICAgICAgZmlsbCA9IGNvbG9yKSwgYWxwaGE9MC4yNSwgbGluZXdpZHRoPTAuMDUpICsNCiAgDQogIGdlb21fcGF0aChkYXRhID0gbGluZV9kYXRhW2xpbmVfZGF0YSRjYXRlZ29yeSA9PSAiYmlvdGljX1dOIixdLCANCiAgICAgICAgICAgIGFlcyh4ID0gc2FtcGxpbmdfdGltZSwgeSA9IGZvcm1hbGRlaHlkZV9tZWFuLCBjb2xvciA9IGNvbG9yKSwgDQogICAgICAgICAgICBsaW5ld2lkdGggPSAxKSArIA0KICBnZW9tX3JpYmJvbihkYXRhID0gbGluZV9kYXRhW2xpbmVfZGF0YSRjYXRlZ29yeSA9PSAiYmlvdGljX1dOIixdLCANCiAgICAgICAgICAgICAgYWVzKHggPSBzYW1wbGluZ190aW1lLCANCiAgICAgICAgICAgICAgICAgIHltaW4gPSBmb3JtYWxkZWh5ZGVfbWVhbiAtIGZvcm1hbGRlaHlkZV9zZCwNCiAgICAgICAgICAgICAgICAgIHltYXggPSBmb3JtYWxkZWh5ZGVfbWVhbiArIGZvcm1hbGRlaHlkZV9zZCwNCiAgICAgICAgICAgICAgICAgIGZpbGwgPSBjb2xvciksIGFscGhhPTAuMjUsIGxpbmV3aWR0aD0wLjA1KSArIA0KICANCiAgZ2VvbV9wYXRoKGRhdGEgPSBsaW5lX2RhdGFbbGluZV9kYXRhJGNhdGVnb3J5ID09ICJhYmlvdGljX1dOIixdLCANCiAgICAgICAgICAgIGFlcyh4ID0gc2FtcGxpbmdfdGltZSwgeSA9IGZvcm1hbGRlaHlkZV9tZWFuLCBjb2xvciA9IGNvbG9yKSwgDQogICAgICAgICAgICBsaW5ld2lkdGggPSAxKSArIA0KICBnZW9tX3JpYmJvbihkYXRhID0gbGluZV9kYXRhW2xpbmVfZGF0YSRjYXRlZ29yeSA9PSAiYWJpb3RpY19XTiIsXSwgDQogICAgICAgICAgICAgIGFlcyh4ID0gc2FtcGxpbmdfdGltZSwgDQogICAgICAgICAgICAgICAgICB5bWluID0gZm9ybWFsZGVoeWRlX21lYW4gLSBmb3JtYWxkZWh5ZGVfc2QsDQogICAgICAgICAgICAgICAgICB5bWF4ID0gZm9ybWFsZGVoeWRlX21lYW4gKyBmb3JtYWxkZWh5ZGVfc2QsDQogICAgICAgICAgICAgICAgICBmaWxsID0gY29sb3IpLCBhbHBoYT0wLjI1LCBsaW5ld2lkdGg9MC4wNSkgKyANCiAgDQogICMgcGxvdCBzaWduaWZpY2FuY2Ugc3RhcnMNCiAgZ2VvbV90ZXh0KGRhdGE9a3J1c2tba3J1c2skY2F0ZWdvcnkgPT0gImNvbW11bml0eSBTRCIsXSwgDQogICAgICAgICAgICBhZXMoeCA9IHRpbWUsIHkgPSBoZWlnaHQsIGxhYmVsID0gcHZhbHVlLCBjb2xvdXI9Y29sb3IpLCANCiAgICAgICAgICAgIHNpemUgPSA3LCBzaG93LmxlZ2VuZCA9IEZBTFNFLCBudWRnZV95ID0gLTAuMDcpICsNCiAgZ2VvbV90ZXh0KGRhdGE9a3J1c2tba3J1c2skY2F0ZWdvcnkgPT0gImNvbW11bml0eSBXTiIsXSwgDQogICAgICAgICAgICBhZXMoeCA9IHRpbWUsIHkgPSBoZWlnaHQsIGxhYmVsID0gcHZhbHVlLCBjb2xvdXI9Y29sb3IpLCANCiAgICAgICAgICAgIHNpemUgPSA3LCBzaG93LmxlZ2VuZCA9IEZBTFNFLCBudWRnZV95ID0gLTAuMDcpICsNCiAgZ2VvbV90ZXh0KGRhdGE9a3J1c2tba3J1c2skY2F0ZWdvcnkgPT0gImFiaW90aWMgU0QiLF0sDQogICAgICAgICAgICBhZXMoeCA9IHRpbWUsIHkgPSBoZWlnaHQsIGxhYmVsID0gcHZhbHVlLCBjb2xvdXI9Y29sb3IpLA0KICAgICAgICAgICAgc2l6ZSA9IDcsIHNob3cubGVnZW5kID0gRkFMU0UpICsNCiAgZ2VvbV90ZXh0KGRhdGE9a3J1c2tba3J1c2skY2F0ZWdvcnkgPT0gImFiaW90aWMgV04iLF0sDQogICAgICAgICAgICBhZXMoeCA9IHRpbWUsIHkgPSBoZWlnaHQsIGxhYmVsID0gcHZhbHVlLCBjb2xvdXI9Y29sb3IpLA0KICAgICAgICAgICAgc2l6ZSA9IDcsIHNob3cubGVnZW5kID0gRkFMU0UpICsNCiAgDQogIGdlb21fc2VnbWVudChkYXRhPXNlZ21lbnRfZGF0YSwgYWVzKHggPSB4LCB4ZW5kID0geGVuZCwgeSA9IHksIHllbmQgPSB5ZW5kKSkgKw0KICBnZW9tX3RleHQoZGF0YT1zZWdtZW50X2RhdGEsIGFlcyh4ID0geCArIDAuMiwgeSA9IDAuMjAsIGxhYmVsID0gIkNlbGwgc2FtcGxpbmciKSwgYW5nbGU9OTAsIHNpemU9MykgLT4gcGxvdF9mb3JtYWxkZWh5ZGUNCg0KcHJpbnQocGxvdF9mb3JtYWxkZWh5ZGUpDQoNCiMgZ2dzYXZlKHBsb3QgPSBwbG90X2Zvcm1hbGRlaHlkZSwgZmlsZW5hbWUgPSAiLi4vZmlndXJlcy9mb3JtYWxkZWh5ZGVfZXZvbHV0aW9uLnRpZmYiLCBkcGkgPSAzMDAsIHdpZHRoID0gMTAsIGhlaWdodCA9IDYsIGJnPSJ3aGl0ZSIpDQogIA0KYGBgDQoNCg0KIyMjIEgyTzIgZXZvbHV0aW9uDQoNCmBgYHtyIGNoZW1fcGxvdHNfSDJPMn0NCg0KY2hlbV9kYXRhX21ldGFUJGV4cGVyaW1lbnQgPC0gIm1ldGFUIg0KY2hlbV9kYXRhX21ldGFCJGV4cGVyaW1lbnQgPC0gIm1ldGFCIg0KDQpjaGVtX2RhdGEgPC0gcmJpbmQoY2hlbV9kYXRhX21ldGFULCBjaGVtX2RhdGFfbWV0YUIpDQoNCg0KDQojIyBNZXRhdHJhbnNjcmlwdG9taWNzIGRhdGENCg0KY2hlbV9kYXRhW2NoZW1fZGF0YSRjb2xvciA9PSAiI0Y4QUQxOCIgJiBjaGVtX2RhdGEkZXhwZXJpbWVudCA9PSAibWV0YVQiLF0gLT4gdG1wMg0KZ3JvdXBfYnkodG1wMiwgc2FtcGxpbmdfdGltZSkgJT4lIHN1bW1hcmlzZShIMk8yX21lYW49bWVhbihIMk8yX3JhdGlvX3RvX2luaXRpYWwpLCANCiAgICAgICAgICAgICAgICAgICAgICAgICAgICAgICAgICAgICAgICAgICAgSDJPMl9zZD1zZChIMk8yX3JhdGlvX3RvX2luaXRpYWwpKSAtPiB0bXAyDQp0bXAyJGNvbG9yIDwtICIjRjhBRDE4Ig0KdG1wMiRjb25kaXRpb24gPC0gIlNEIg0KdG1wMiRjYXRlZ29yeSA8LSAiYmlvdGljX1NEIg0KdG1wMiRleHBlcmltZW50IDwtICJtZXRhVCINCg0KDQpjaGVtX2RhdGFbY2hlbV9kYXRhJGNvbG9yID09ICIjQzRCNjlCIiAmIGNoZW1fZGF0YSRleHBlcmltZW50ID09ICJtZXRhVCIsXSAtPiB0bXA0DQpncm91cF9ieSh0bXA0LCBzYW1wbGluZ190aW1lKSAlPiUgc3VtbWFyaXNlKEgyTzJfbWVhbj1tZWFuKEgyTzJfcmF0aW9fdG9faW5pdGlhbCksIA0KICAgICAgICAgICAgICAgICAgICAgICAgICAgICAgICAgICAgICAgICAgICBIMk8yX3NkPXNkKEgyTzJfcmF0aW9fdG9faW5pdGlhbCkpIC0+IHRtcDQNCnRtcDQkY29sb3IgPC0gIiNDNEI2OUIiDQp0bXA0JGNvbmRpdGlvbiA8LSAiU0QiDQp0bXA0JGNhdGVnb3J5IDwtICJhYmlvdGljX1NEIg0KdG1wNCRleHBlcmltZW50IDwtICJtZXRhVCINCg0KDQojIyBNZXRhYm9sb21pY3MgZGF0YQ0KDQpjaGVtX2RhdGFbY2hlbV9kYXRhJGNvbG9yID09ICIjRjhBRDE4IiAmIGNoZW1fZGF0YSRleHBlcmltZW50ID09ICJtZXRhQiIsXSAtPiB0bXA2DQpncm91cF9ieSh0bXA2LCBzYW1wbGluZ190aW1lKSAlPiUgc3VtbWFyaXNlKEgyTzJfbWVhbj1tZWFuKEgyTzJfcmF0aW9fdG9faW5pdGlhbCksIA0KICAgICAgICAgICAgICAgICAgICAgICAgICAgICAgICAgICAgICAgICAgICBIMk8yX3NkPXNkKEgyTzJfcmF0aW9fdG9faW5pdGlhbCkpIC0+IHRtcDYNCnRtcDYkY29sb3IgPC0gIiNGOEFEMTgiDQp0bXA2JGNvbmRpdGlvbiA8LSAiU0QiDQp0bXA2JGNhdGVnb3J5IDwtICJiaW90aWNfU0QiDQp0bXA2JGV4cGVyaW1lbnQgPC0gIm1ldGFCIg0KDQoNCmNoZW1fZGF0YVtjaGVtX2RhdGEkY29sb3IgPT0gIiNDNEI2OUIiICYgY2hlbV9kYXRhJGV4cGVyaW1lbnQgPT0gIm1ldGFCIixdIC0+IHRtcDgNCmdyb3VwX2J5KHRtcDgsIHNhbXBsaW5nX3RpbWUpICU+JSBzdW1tYXJpc2UoSDJPMl9tZWFuPW1lYW4oSDJPMl9yYXRpb190b19pbml0aWFsKSwgDQogICAgICAgICAgICAgICAgICAgICAgICAgICAgICAgICAgICAgICAgICAgIEgyTzJfc2Q9c2QoSDJPMl9yYXRpb190b19pbml0aWFsKSkgLT4gdG1wOA0KdG1wOCRjb2xvciA8LSAiI0M0QjY5QiINCnRtcDgkY29uZGl0aW9uIDwtICJTRCINCnRtcDgkY2F0ZWdvcnkgPC0gImFiaW90aWNfU0QiDQp0bXA4JGV4cGVyaW1lbnQgPC0gIm1ldGFCIg0KDQpjaGVtX2RhdGEkY29sb3IgPC0gYXMuZmFjdG9yKGNoZW1fZGF0YSRjb2xvcikNCiMjIE1lcmdlIGRhdGENCg0KbGluZV9kYXRhIDwtIHJiaW5kKHRtcDIsIHRtcDQsIHRtcDYsIHRtcDgpDQpybSh0bXAyLCB0bXA0LCB0bXA2LCB0bXA4KQ0KDQojIyBUZXN0IGRpZmZlcmVuY2UgYmV0d2VlbiB0aW1lIDAgYW4gdGhlIG90aGVyIHRpbWUgcG9pbnRzDQoNCmtydXNrYWxfaDJvMiA8LSAgYygiIiwNCiAgICAgICAgICAgICAgICAgICAgICAgICAgdHJ5KGlmKGtydXNrYWwudGVzdCh4ID0gYyhjaGVtX2RhdGFfbWV0YUJbKGNoZW1fZGF0YV9tZXRhQiRzYW1wbGluZ190aW1lID09IDEgJg0KICAgICAgICAgICAgICAgICAgICAgICAgICAgICAgICAgICAgICAgICAgICAgICAgICAgICAgICAgICAgICAgICAgICAgY2hlbV9kYXRhX21ldGFCJGNvbG9yID09ICIjRjhBRDE4IiksDQogICAgICAgICAgICAgICAgICAgICAgICAgICAgICAgICAgICAgICAgICAgICAgICAgICAgICAgICAgICAgICAgICAgICJIMk8yX3JhdGlvX3RvX2luaXRpYWwiXSwNCiAgICAgICAgICAgICAgICAgICAgICAgICAgICAgICAgICAgICAgICAgICAgICAgICAgICBjaGVtX2RhdGFfbWV0YUJbKGNoZW1fZGF0YV9tZXRhQiRzYW1wbGluZ190aW1lID09IDEgJg0KICAgICAgICAgICAgICAgICAgICAgICAgICAgICAgICAgICAgICAgICAgICAgICAgICAgICAgICAgICAgICAgICAgICAgY2hlbV9kYXRhX21ldGFCJGNvbG9yID09ICIjQzRCNjlCIiksDQogICAgICAgICAgICAgICAgICAgICAgICAgICAgICAgICAgICAgICAgICAgICAgICAgICAgICAgICAgICAgICAgICAgICJIMk8yX3JhdGlvX3RvX2luaXRpYWwiXSksDQogICAgICAgICAgICAgICAgICAgICAgICAgICAgICAgICAgICAgICAgICAgICAgZyA9IGMocmVwKCJiaW90aWNfU0QiLCAzKSwgcmVwKCJhYmlvdGljX1NEIiwgMykpDQogICAgICAgICAgICAgICAgICAgICAgICAgIClbWyJwLnZhbHVlIl1dIDwgMC4wNSl7IioifWVsc2V7IiJ9LCBzaWxlbnQgPSBUUlVFKSAsDQogICAgICAgICAgICAgICAgICAgICAgICAgIHRyeShpZihrcnVza2FsLnRlc3QoeCA9IGMoY2hlbV9kYXRhX21ldGFCWyhjaGVtX2RhdGFfbWV0YUIkc2FtcGxpbmdfdGltZSA9PSAyICYNCiAgICAgICAgICAgICAgICAgICAgICAgICAgICAgICAgICAgICAgICAgICAgICAgICAgICAgICAgICAgICAgICAgICAgIGNoZW1fZGF0YV9tZXRhQiRjb2xvciA9PSAiI0Y4QUQxOCIpLA0KICAgICAgICAgICAgICAgICAgICAgICAgICAgICAgICAgICAgICAgICAgICAgICAgICAgICAgICAgICAgICAgICAgICAiSDJPMl9yYXRpb190b19pbml0aWFsIl0sDQogICAgICAgICAgICAgICAgICAgICAgICAgICAgICAgICAgICAgICAgICAgICAgICAgICAgY2hlbV9kYXRhX21ldGFCWyhjaGVtX2RhdGFfbWV0YUIkc2FtcGxpbmdfdGltZSA9PSAyICYNCiAgICAgICAgICAgICAgICAgICAgICAgICAgICAgICAgICAgICAgICAgICAgICAgICAgICAgICAgICAgICAgICAgICAgIGNoZW1fZGF0YV9tZXRhQiRjb2xvciA9PSAiI0M0QjY5QiIpLA0KICAgICAgICAgICAgICAgICAgICAgICAgICAgICAgICAgICAgICAgICAgICAgICAgICAgICAgICAgICAgICAgICAgICAiSDJPMl9yYXRpb190b19pbml0aWFsIl0pLA0KICAgICAgICAgICAgICAgICAgICAgICAgICAgICAgICAgICAgICAgICAgICAgIGcgPSBjKHJlcCgiYmlvdGljX1NEIiwgMyksIHJlcCgiYWJpb3RpY19TRCIsIDMpKQ0KICAgICAgICAgICAgICAgICAgICAgICAgICApW1sicC52YWx1ZSJdXSA8IDAuMDUpeyIqIn1lbHNleyIifSwgc2lsZW50ID0gVFJVRSksDQogICAgICAgICAgICAgICAgICAgICAgICAgIHRyeShpZihrcnVza2FsLnRlc3QoeCA9IGMoY2hlbV9kYXRhX21ldGFCWyhjaGVtX2RhdGFfbWV0YUIkc2FtcGxpbmdfdGltZSA9PSAzICYNCiAgICAgICAgICAgICAgICAgICAgICAgICAgICAgICAgICAgICAgICAgICAgICAgICAgICAgICAgICAgICAgICAgICAgIGNoZW1fZGF0YV9tZXRhQiRjb2xvciA9PSAiI0Y4QUQxOCIpLA0KICAgICAgICAgICAgICAgICAgICAgICAgICAgICAgICAgICAgICAgICAgICAgICAgICAgICAgICAgICAgICAgICAgICAiSDJPMl9yYXRpb190b19pbml0aWFsIl0sDQogICAgICAgICAgICAgICAgICAgICAgICAgICAgICAgICAgICAgICAgICAgICAgICAgICAgY2hlbV9kYXRhX21ldGFCWyhjaGVtX2RhdGFfbWV0YUIkc2FtcGxpbmdfdGltZSA9PSAzICYNCiAgICAgICAgICAgICAgICAgICAgICAgICAgICAgICAgICAgICAgICAgICAgICAgICAgICAgICAgICAgICAgICAgICAgIGNoZW1fZGF0YV9tZXRhQiRjb2xvciA9PSAiI0M0QjY5QiIpLA0KICAgICAgICAgICAgICAgICAgICAgICAgICAgICAgICAgICAgICAgICAgICAgICAgICAgICAgICAgICAgICAgICAgICAiSDJPMl9yYXRpb190b19pbml0aWFsIl0pLA0KICAgICAgICAgICAgICAgICAgICAgICAgICAgICAgICAgICAgICAgICAgICAgIGcgPSBjKHJlcCgiYmlvdGljX1NEIiwgMyksIHJlcCgiYWJpb3RpY19TRCIsIDMpKQ0KICAgICAgICAgICAgICAgICAgICAgICAgICApW1sicC52YWx1ZSJdXSA8IDAuMDUpeyIqIn1lbHNleyIifSwgc2lsZW50ID0gVFJVRSksDQogICAgICAgICAgICAgICAgICAgICAgICAgICIiLA0KICAgICAgICAgICAgICAgICAgICAgICAgICB0cnkoaWYoa3J1c2thbC50ZXN0KHggPSBjKGNoZW1fZGF0YV9tZXRhVFsoY2hlbV9kYXRhX21ldGFUJHNhbXBsaW5nX3RpbWUgPT0gMSAmDQogICAgICAgICAgICAgICAgICAgICAgICAgICAgICAgICAgICAgICAgICAgICAgICAgICAgICAgICAgICAgICAgICAgICBjaGVtX2RhdGFfbWV0YVQkY29sb3IgPT0gIiNGOEFEMTgiKSwNCiAgICAgICAgICAgICAgICAgICAgICAgICAgICAgICAgICAgICAgICAgICAgICAgICAgICAgICAgICAgICAgICAgICAgIkgyTzJfcmF0aW9fdG9faW5pdGlhbCJdLA0KICAgICAgICAgICAgICAgICAgICAgICAgICAgICAgICAgICAgICAgICAgICAgICAgICAgIGNoZW1fZGF0YV9tZXRhVFsoY2hlbV9kYXRhX21ldGFUJHNhbXBsaW5nX3RpbWUgPT0gMSAmDQogICAgICAgICAgICAgICAgICAgICAgICAgICAgICAgICAgICAgICAgICAgICAgICAgICAgICAgICAgICAgICAgICAgICBjaGVtX2RhdGFfbWV0YVQkY29sb3IgPT0gIiNDNEI2OUIiKSwNCiAgICAgICAgICAgICAgICAgICAgICAgICAgICAgICAgICAgICAgICAgICAgICAgICAgICAgICAgICAgICAgICAgICAgIkgyTzJfcmF0aW9fdG9faW5pdGlhbCJdKSwNCiAgICAgICAgICAgICAgICAgICAgICAgICAgICAgICAgICAgICAgICAgICAgICBnID0gYyhyZXAoImJpb3RpY19TRCIsIDMpLCByZXAoImFiaW90aWNfU0QiLCAzKSkNCiAgICAgICAgICAgICAgICAgICAgICAgICAgKVtbInAudmFsdWUiXV0gPCAwLjA1KXsiKiJ9ZWxzZXsiIn0sIHNpbGVudCA9IFRSVUUpICwNCiAgICAgICAgICAgICAgICAgICAgICAgICAgdHJ5KGlmKGtydXNrYWwudGVzdCh4ID0gYyhjaGVtX2RhdGFfbWV0YVRbKGNoZW1fZGF0YV9tZXRhVCRzYW1wbGluZ190aW1lID09IDIgJg0KICAgICAgICAgICAgICAgICAgICAgICAgICAgICAgICAgICAgICAgICAgICAgICAgICAgICAgICAgICAgICAgICAgICAgY2hlbV9kYXRhX21ldGFUJGNvbG9yID09ICIjRjhBRDE4IiksDQogICAgICAgICAgICAgICAgICAgICAgICAgICAgICAgICAgICAgICAgICAgICAgICAgICAgICAgICAgICAgICAgICAgICJIMk8yX3JhdGlvX3RvX2luaXRpYWwiXSwNCiAgICAgICAgICAgICAgICAgICAgICAgICAgICAgICAgICAgICAgICAgICAgICAgICAgICBjaGVtX2RhdGFfbWV0YVRbKGNoZW1fZGF0YV9tZXRhVCRzYW1wbGluZ190aW1lID09IDIgJg0KICAgICAgICAgICAgICAgICAgICAgICAgICAgICAgICAgICAgICAgICAgICAgICAgICAgICAgICAgICAgICAgICAgICAgY2hlbV9kYXRhX21ldGFUJGNvbG9yID09ICIjQzRCNjlCIiksDQogICAgICAgICAgICAgICAgICAgICAgICAgICAgICAgICAgICAgICAgICAgICAgICAgICAgICAgICAgICAgICAgICAgICJIMk8yX3JhdGlvX3RvX2luaXRpYWwiXSksDQogICAgICAgICAgICAgICAgICAgICAgICAgICAgICAgICAgICAgICAgICAgICAgZyA9IGMocmVwKCJiaW90aWNfU0QiLCAzKSwgcmVwKCJhYmlvdGljX1NEIiwgMykpDQogICAgICAgICAgICAgICAgICAgICAgICAgIClbWyJwLnZhbHVlIl1dIDwgMC4wNSl7IioifWVsc2V7IiJ9LCBzaWxlbnQgPSBUUlVFKSwNCiAgICAgICAgICAgICAgICAgICAgICAgICAgdHJ5KGlmKGtydXNrYWwudGVzdCh4ID0gYyhjaGVtX2RhdGFfbWV0YVRbKGNoZW1fZGF0YV9tZXRhVCRzYW1wbGluZ190aW1lID09IDMgJg0KICAgICAgICAgICAgICAgICAgICAgICAgICAgICAgICAgICAgICAgICAgICAgICAgICAgICAgICAgICAgICAgICAgICAgY2hlbV9kYXRhX21ldGFUJGNvbG9yID09ICIjRjhBRDE4IiksDQogICAgICAgICAgICAgICAgICAgICAgICAgICAgICAgICAgICAgICAgICAgICAgICAgICAgICAgICAgICAgICAgICAgICJIMk8yX3JhdGlvX3RvX2luaXRpYWwiXSwNCiAgICAgICAgICAgICAgICAgICAgICAgICAgICAgICAgICAgICAgICAgICAgICAgICAgICBjaGVtX2RhdGFfbWV0YVRbKGNoZW1fZGF0YV9tZXRhVCRzYW1wbGluZ190aW1lID09IDMgJg0KICAgICAgICAgICAgICAgICAgICAgICAgICAgICAgICAgICAgICAgICAgICAgICAgICAgICAgICAgICAgICAgICAgICAgY2hlbV9kYXRhX21ldGFUJGNvbG9yID09ICIjQzRCNjlCIiksDQogICAgICAgICAgICAgICAgICAgICAgICAgICAgICAgICAgICAgICAgICAgICAgICAgICAgICAgICAgICAgICAgICAgICJIMk8yX3JhdGlvX3RvX2luaXRpYWwiXSksDQogICAgICAgICAgICAgICAgICAgICAgICAgICAgICAgICAgICAgICAgICAgICAgZyA9IGMocmVwKCJiaW90aWNfU0QiLCAzKSwgcmVwKCJhYmlvdGljX1NEIiwgMykpDQogICAgICAgICAgICAgICAgICAgICAgICAgIClbWyJwLnZhbHVlIl1dIDwgMC4wNSl7IioifWVsc2V7IiJ9LCBzaWxlbnQgPSBUUlVFKSwNCiAgICAgICAgICAgICAgICAgICAgICAgICAgdHJ5KGlmKGtydXNrYWwudGVzdCh4ID0gYyhjaGVtX2RhdGFfbWV0YVRbKGNoZW1fZGF0YV9tZXRhVCRzYW1wbGluZ190aW1lID09IDUgJg0KICAgICAgICAgICAgICAgICAgICAgICAgICAgICAgICAgICAgICAgICAgICAgICAgICAgICAgICAgICAgICAgICAgICAgY2hlbV9kYXRhX21ldGFUJGNvbG9yID09ICIjRjhBRDE4IiksDQogICAgICAgICAgICAgICAgICAgICAgICAgICAgICAgICAgICAgICAgICAgICAgICAgICAgICAgICAgICAgICAgICAgICJIMk8yX3JhdGlvX3RvX2luaXRpYWwiXSwNCiAgICAgICAgICAgICAgICAgICAgICAgICAgICAgICAgICAgICAgICAgICAgICAgICAgICBjaGVtX2RhdGFfbWV0YVRbKGNoZW1fZGF0YV9tZXRhVCRzYW1wbGluZ190aW1lID09IDUgJg0KICAgICAgICAgICAgICAgICAgICAgICAgICAgICAgICAgICAgICAgICAgICAgICAgICAgICAgICAgICAgICAgICAgICAgY2hlbV9kYXRhX21ldGFUJGNvbG9yID09ICIjQzRCNjlCIiksDQogICAgICAgICAgICAgICAgICAgICAgICAgICAgICAgICAgICAgICAgICAgICAgICAgICAgICAgICAgICAgICAgICAgICJIMk8yX3JhdGlvX3RvX2luaXRpYWwiXSksDQogICAgICAgICAgICAgICAgICAgICAgICAgICAgICAgICAgICAgICAgICAgICAgZyA9IGMocmVwKCJiaW90aWNfU0QiLCAzKSwgcmVwKCJhYmlvdGljX1NEIiwgMykpDQogICAgICAgICAgICAgICAgICAgICAgICAgIClbWyJwLnZhbHVlIl1dIDwgMC4wNSl7IioifWVsc2V7IiJ9LCBzaWxlbnQgPSBUUlVFKQ0KICAgICAgICAgICAgICAgICAgICAgKQ0KDQprcnVza2FsX2RmIDwtIGRhdGEuZnJhbWUodGltZSA9IGMoMCwgMSwgMiwgMywgMCwgMSwgMiwgMywgNSksDQogICAgICAgICAgICAgICAgICAgICAgICAgaGVpZ2h0ID0gcmVwKDAsIDkpLA0KICAgICAgICAgICAgICAgICAgICAgICAgIGV4cGVyaW1lbnQgPSBjKHJlcCgibWV0YUIiLCA0KSwgcmVwKCJtZXRhVCIsIDUpKSwNCiAgICAgICAgICAgICAgICAgICAgICAgICBjb25kaXRpb24gPSByZXAoIlNEIiwgOSksDQogICAgICAgICAgICAgICAgICAgICAgICAgY2F0ZWdvcnkgPSByZXAoImNvbW11bml0eSBTRCIsIDkpLA0KICAgICAgICAgICAgICAgICAgICAgICAgIGNvbG9yID0gcmVwKCIjRjhBRDE4IiwgOSksDQogICAgICAgICAgICAgICAgICAgICAgICAgcHZhbHVlID0ga3J1c2thbF9oMm8yKQ0KDQoNCmtydXNrIDwtIGtydXNrYWxfZGYNCg0KDQojIyBTZWdtZW50IGRhdGEgZm9yIHNhbXBsaW5nIHRpbWUNCg0Kc2VnbWVudF9kYXRhIDwtIGRhdGEuZnJhbWUoeCA9IGMoNCwgMCwgMy41KSwgeGVuZCA9IGMoNCwgMCwgMy41KSwgDQogICAgICAgICAgICAgICAgICAgICAgICAgICB5ID0gYygtSW5mLCAtSW5mLCAtSW5mKSwgeWVuZCA9IGMoSW5mLCBJbmYsIEluZiksDQogICAgICAgICAgICAgICAgICAgICAgICAgICBleHBlcmltZW50ID0gYygibWV0YVQiLCAibWV0YUIiLCAibWV0YUIiKSkNCiAgDQpjaGVtX2RhdGFbY2hlbV9kYXRhJGNvbmRpdGlvbiA9PSAiU0QiLF0gJT4lIGdncGxvdCgpICsNCiAgZ2VvbV9wb2ludChhZXMoeD1zYW1wbGluZ190aW1lLCB5PUgyTzJfcmF0aW9fdG9faW5pdGlhbCwgY29sb3I9Y29sb3IpKSArDQogIHlsaW0oYygtMC4xLCAxLjIpKSArDQogIHhsaW0oYygwLCA1KSkgKw0KICBzY2FsZV9jb2xvdXJfaWRlbnRpdHkoIkNhdGVnb3J5IiwgbGFiZWxzID0gYygiQXNzZW1ibGFnZSBXaW50ZXIgTmlnaHQiLCAiQWJpb3RpYyBXaW50ZXIgTmlnaHQiLA0KICAgICAgICAgICAgICAgICAgICAgICAgICAgICAgICAgICAgICAgICAgICAgICAiQWJpb3RpYyBTdW1tZXIgRGF5IiwgIkFzc2VtYmxhZ2UgU3VtbWVyIERheSIpLCANCiAgICAgICAgICAgICAgICAgICAgICAgIGd1aWRlPSJsZWdlbmQiLCBkcm9wID0gRkFMU0UpICsNCiAgc2NhbGVfZmlsbF9pZGVudGl0eSgpICsNCg0KICB5bGFiKCJSZWxhdGl2ZSBIPHN1Yj4yPC9zdWI+TzxzdWI+Mjwvc3ViPiBldm9sdXRpb24iKSArDQogIHhsYWIobGFiZWwgPSAiSW5jdWJhdGlvbiB0aW1lIChoKSIpICsNCg0KICBmYWNldF9ncmlkKHJvd3MgPSB2YXJzKGNvbmRpdGlvbiksIGNvbHMgPSB2YXJzKGV4cGVyaW1lbnQpLA0KICAgICAgICAgICAgIGxhYmVsbGVyID0gYXNfbGFiZWxsZXIoYygibWV0YVQiID0gIk1ldGF0cmFuc2NyaXB0b21pY3MiLCANCiAgICAgICAgICAgICAgICAgICAgICAgICAgICAgICAgICAgICAgIm1ldGFCIiA9ICJNZXRhLW1ldGFib2xvbWljcyIsDQogICAgICAgICAgICAgICAgICAgICAgICAgICAgICAgICAgICAgICJTRCIgPSAiU3VtbWVyIERheSIsDQogICAgICAgICAgICAgICAgICAgICAgICAgICAgICAgICAgICAgICJXTiIgPSAiV2ludGVyIE5pZ2h0IikpKSArIA0KICB0aGVtZShsZWdlbmQucG9zaXRpb24gPSAiYm90dG9tIiwNCiAgICAgICAgcGFuZWwuYmFja2dyb3VuZCA9IGVsZW1lbnRfcmVjdChmaWxsPSIjZjRmNGY0IiksDQogICAgICAgIGF4aXMudGl0bGUueSA9IGVsZW1lbnRfbWFya2Rvd24oKSwNCiAgICAgICAgc3RyaXAudGV4dCA9IGVsZW1lbnRfdGV4dChzaXplPTEyKSkgKw0KICANCiAgDQogIGdlb21fcGF0aChkYXRhID0gbGluZV9kYXRhW2xpbmVfZGF0YSRjYXRlZ29yeSA9PSAiYmlvdGljX1NEIixdLCANCiAgICAgICAgICAgIGFlcyh4ID0gc2FtcGxpbmdfdGltZSwgeSA9IEgyTzJfbWVhbiwgY29sb3IgPSBjb2xvciksIA0KICAgICAgICAgICAgbGluZXdpZHRoID0gMSkgKyANCiAgZ2VvbV9yaWJib24oZGF0YSA9IGxpbmVfZGF0YVtsaW5lX2RhdGEkY2F0ZWdvcnkgPT0gImJpb3RpY19TRCIsXSwgDQogICAgICAgICAgICAgIGFlcyh4ID0gc2FtcGxpbmdfdGltZSwgDQogICAgICAgICAgICAgICAgICB5bWluID0gSDJPMl9tZWFuIC0gSDJPMl9zZCwNCiAgICAgICAgICAgICAgICAgIHltYXggPSBIMk8yX21lYW4gKyBIMk8yX3NkLA0KICAgICAgICAgICAgICAgICAgZmlsbCA9IGNvbG9yKSwgYWxwaGE9MC4yNSwgbGluZXdpZHRoPTAuMDUpICsNCiAgDQogIGdlb21fcGF0aChkYXRhID0gbGluZV9kYXRhW2xpbmVfZGF0YSRjYXRlZ29yeSA9PSAiYWJpb3RpY19TRCIsXSwgDQogICAgICAgICAgICBhZXMoeCA9IHNhbXBsaW5nX3RpbWUseSA9SDJPMl9tZWFuLCBjb2xvciA9IGNvbG9yKSwgDQogICAgICAgICAgICBsaW5ld2lkdGggPSAxKSArIA0KICBnZW9tX3JpYmJvbihkYXRhID0gbGluZV9kYXRhW2xpbmVfZGF0YSRjYXRlZ29yeSA9PSAiYWJpb3RpY19TRCIsXSwgDQogICAgICAgICAgICAgIGFlcyh4ID0gc2FtcGxpbmdfdGltZSwgDQogICAgICAgICAgICAgICAgICB5bWluID0gSDJPMl9tZWFuIC0gSDJPMl9zZCwNCiAgICAgICAgICAgICAgICAgIHltYXggPSBIMk8yX21lYW4gKyBIMk8yX3NkLA0KICAgICAgICAgICAgICAgICAgZmlsbCA9IGNvbG9yKSwgYWxwaGE9MC4yNSwgbGluZXdpZHRoPTAuMDUpICsNCiAgDQogICMgcGxvdCBzaWduaWZpY2FuY2Ugc3RhcnMNCiAgZ2VvbV90ZXh0KGRhdGE9a3J1c2tba3J1c2skY2F0ZWdvcnkgPT0gImNvbW11bml0eSBTRCIsXSwgDQogICAgICAgICAgICBhZXMoeCA9IHRpbWUsIHkgPSBoZWlnaHQsIGxhYmVsID0gcHZhbHVlLCBjb2xvdXI9Y29sb3IpLCANCiAgICAgICAgICAgIHNpemUgPSA3LCBzaG93LmxlZ2VuZCA9IEZBTFNFLCBudWRnZV95ID0gLTAuMDcpICsNCiAgZ2VvbV90ZXh0KGRhdGE9a3J1c2tba3J1c2skY2F0ZWdvcnkgPT0gImFiaW90aWMgU0QiLF0sDQogICAgICAgICAgICBhZXMoeCA9IHRpbWUsIHkgPSBoZWlnaHQsIGxhYmVsID0gcHZhbHVlLCBjb2xvdXI9Y29sb3IpLA0KICAgICAgICAgICAgc2l6ZSA9IDcsIHNob3cubGVnZW5kID0gRkFMU0UpICsNCg0KICANCiAgIyBnZW9tX3NlZ21lbnQoYWVzKHg9NCwgeGVuZD00LCB5PS1JbmYsIHllbmQ9SW5mKSkgKw0KICBnZW9tX3NlZ21lbnQoZGF0YT1zZWdtZW50X2RhdGEsIGFlcyh4ID0geCwgeGVuZCA9IHhlbmQsIHkgPSB5LCB5ZW5kID0geWVuZCkpICsNCiAgZ2VvbV9zZWdtZW50KGFlcyh4ID0gLUluZiwgeGVuZCA9IEluZiwgeSA9IDAuMiwgeWVuZCA9IDAuMiksIGNvbG91ciA9ICJkYXJrcmVkIiwgbGluZXR5cGUgPSAyKSArDQogIGdlb21fdGV4dChkYXRhPXNlZ21lbnRfZGF0YSwgYWVzKHggPSB4ICsgMC4yLCB5ID0gMC40MiwgbGFiZWwgPSAiQ2VsbCBzYW1wbGluZyIpLCBhbmdsZT05MCwgc2l6ZT0zKSAtPiBwbG90X2gybzINCg0KcHJpbnQocGxvdF9oMm8yKQ0KDQojIGdnc2F2ZShwbG90ID0gcGxvdF9oMm8yLCBmaWxlbmFtZSA9ICIuLi9maWd1cmVzL0gyTzJfZXZvbHV0aW9uLnRpZmYiLCBkcGkgPSAzMDAsIHdpZHRoID0gMTAsIGhlaWdodCA9IDMuNiwgYmc9IndoaXRlIikNCiAgDQpgYGANCg0KDQoNCmBgYHtyIHBsb3RfRmlndXJlUzEsIGZpZy5oZWlnaHQ9MTUsIGZpZy5kcGk9MjAwLCBmaWcud2lkdGg9MTV9DQpwbG90X2NoZW1pY2FsIDwtIGdnYXJyYW5nZShwbG90X2gybzIsIHBsb3RfZm9ybWFsZGVoeWRlLCAgDQogICAgICAgICAgICAgICAgICAgICAgbGFiZWxzID0gYygiQSIsICJCIiksIA0KICAgICAgICAgICAgICAgICAgICAgIGNvbW1vbi5sZWdlbmQ9VFJVRSwNCiAgICAgICAgICAgICAgICAgICAgICBsZWdlbmQ9ImJvdHRvbSIsDQogICAgICAgICAgICAgICAgICAgICAgbnJvdz0yLA0KICAgICAgICAgICAgICAgICAgICAgIGhlaWdodHMgPSBjKDEuMSwgMS45KSkNCg0KDQpnZ3NhdmUocGxvdCA9IHBsb3RfY2hlbWljYWwsIGZpbGVuYW1lID0gIi4uL2ZpZ3VyZXMvRmlndXJlX1MxLnRpZmYiLCBkcGkgPSAzMDAsIHdpZHRoID0gMTAsIGhlaWdodCA9IDEwLCBiZz0id2hpdGUiKQ0KDQojIHByaW50KHBsb3RfY2hlbWljYWwpDQpgYGANCg0KDQojIyBEZXNjcmlwdGl2ZSBzdGF0aXN0aWNzIG9uIGNvbXBsZXRlIGFzc2VtYmxhZ2UgbWV0YXRyYW5zY3JpcHRvbWljcyBhbmQgbWV0YWJvbG9taWNzIGRhdGENCg0KIyMjIE1ldGF0cmFuc2NyaXB0b21pY3MNCg0KIyMjIyBTYW1wbGUtd2lzZSBzdGF0aXN0aWNzIChyYXcgbWV0YXRyYW5zY3JpcHRvbWljcyBjb3VudHMpDQoNCmBgYHtyIHNhbXBsZS13aXNlX3N0YXRzfQ0KDQptZXNzYWdlKCJDb21wdXRpbmcgc2FtcGxlLXdpc2Ugc3RhdGlzdGljcyBvbiByYXcgY291bnRzIikNCnNhbXBsZV9zdGF0X3ByZW5vcm0gPC0gZGF0YS5mcmFtZSgNCiAgbWVhbiA9IGFwcGx5KGNvdW50c190YWJsZSwgMiwgbWVhbiwgbmEucm0gPSBUUlVFKSwNCiAgc2QgPSBhcHBseShjb3VudHNfdGFibGUsIDIsIHNkLCBuYS5ybSA9IFRSVUUpLA0KICBpcXIgPSBhcHBseShjb3VudHNfdGFibGUsIDIsIElRUiwgbmEucm0gPSBUUlVFKSwNCiAgUTEgPSBhcHBseShjb3VudHNfdGFibGUsIDIsIHF1YW50aWxlLCBwID0gMC4yNSwgbmEucm0gPSBUUlVFKSwNCiAgbWVkaWFuID0gYXBwbHkoY291bnRzX3RhYmxlLCAyLCBtZWRpYW4sIG5hLnJtID0gVFJVRSksDQogIFEzID0gYXBwbHkoY291bnRzX3RhYmxlLCAyLCBxdWFudGlsZSwgcCA9IDAuNzUsIG5hLnJtID0gVFJVRSksDQogIG1heCA9IGFwcGx5KGNvdW50c190YWJsZSwgMiwgbWF4LCBuYS5ybSA9IFRSVUUpLA0KICBudWxsID0gYXBwbHkoY291bnRzX3RhYmxlID09IDAsIDIsIHN1bSwgbmEucm0gPSBUUlVFKQ0KKQ0KYGBgDQoNCiMjIyMgR2VuZS13aXNlIHN0YXRpc3RpY3MgKHJhdyBtZXRhdHJhbnNjcmlwdG9taWNzIGNvdW50cykNCg0KYGBge3IgZ2VuZS13aXNlX3N0YXRzfQ0KDQptZXNzYWdlKCJDb21wdXRpbmcgZ2VuZS13aXNlIHN0YXRpc3RpY3Mgb24gcmF3IGNvdW50cyIpDQpnZW5lX3N0YXRfcHJlbm9ybSA8LSBkYXRhLmZyYW1lKA0KICBtZWFuID0gYXBwbHkoY291bnRzX3RhYmxlLCAxLCBtZWFuLCBuYS5ybSA9IFRSVUUpLA0KICBzZCA9IGFwcGx5KGNvdW50c190YWJsZSwgMSwgc2QsIG5hLnJtID0gVFJVRSksDQogIGlxciA9IGFwcGx5KGNvdW50c190YWJsZSwgMSwgSVFSLCBuYS5ybSA9IFRSVUUpLA0KICBRMSA9IGFwcGx5KGNvdW50c190YWJsZSwgMSwgcXVhbnRpbGUsIHAgPSAwLjI1LCBuYS5ybSA9IFRSVUUpLA0KICBtZWRpYW4gPSBhcHBseShjb3VudHNfdGFibGUsIDEsIG1lZGlhbiwgbmEucm0gPSBUUlVFKSwNCiAgUTMgPSBhcHBseShjb3VudHNfdGFibGUsIDEsIHF1YW50aWxlLCBwID0gMC43NSwgbmEucm0gPSBUUlVFKSwNCiAgbWF4ID0gYXBwbHkoY291bnRzX3RhYmxlLCAxLCBtYXgsIG5hLnJtID0gVFJVRSksDQogIG51bGwgPSBhcHBseShjb3VudHNfdGFibGUgPT0gMCwgMSwgc3VtLCBuYS5ybSA9IFRSVUUpDQopDQoNCmBgYA0KDQojIyMjIFplcm9lcyBmaWx0ZXJpbmcgYW5kIHNtb290aGluZyBpbnNwaXJlZCBieSBNVFhtb2RlbCBhcnRpY2xlIFsoWmhhbmcgZXQgYWwuIDIwMjEpXShodHRwczovL2RvaS5vcmcvMTAuMTA5My9iaW9pbmZvcm1hdGljcy9idGFiMzI3KQ0KDQpgYGB7ciBsaXN0c30NCnNwZWNpZXMgPC0gdW5saXN0KHVuaXF1ZShhbm5vdGF0aW9uX3RhYmxlWywiT3JnYW5pc20iXSkpDQpzYW1wbGVzIDwtIHJvdy5uYW1lcyhtZXRhZGF0YV90YWJsZSkNCmBgYA0KDQpHZW5lIHdpdGggMCBjb3VudHMgaW4gbW9yZSB0aGFuIDcwJSBvZiBzYW1wbGVzIGFyZSBkZWVtZWQgdW5leHByZXNzZWQgYW5kIGFyZSByZW1vdmVkIGZyb20gdGhlIGRhdGFzZXQuICANCg0KYGBge3IgTVRYX3plcm9fZmlsdGVyaW5nLCByZXN1bHRzPSdhc2lzJ30NCm1lc3NhZ2UoIkZpbHRlcmluZyB1bmRldGVjdGVkIGdlbmVzIikNCnVuZGV0ZWN0ZWRfZ2VuZXMgPC0gZ2VuZV9zdGF0X3ByZW5vcm0kbnVsbCA+PSBuY29sKGNvdW50c190YWJsZSkgKiAwLjcwDQpwcmludChwYXN0ZTAoIlVuZGV0ZWN0ZWQgZ2VuZXMgKG51bGwgaW4gPj0gNzAlIHNhbXBsZXMpOiAiLCBzdW0odW5kZXRlY3RlZF9nZW5lcykpKQ0KDQprZXB0X2dlbmVzIDwtICF1bmRldGVjdGVkX2dlbmVzDQpwcmludChwYXN0ZTAoIktlcHQgZ2VuZXM6ICIsIHN1bShrZXB0X2dlbmVzKSkpDQoNCiMjIEdlbmVzIGFmdGVyIGZpbHRlcmluZw0KY291bnRzX2ZpbHRlcmVkIDwtIGNvdW50c190YWJsZVtrZXB0X2dlbmVzLCBdDQphbm5vdGF0aW9uX3RhYmxlIDwtIGFubm90YXRpb25fYWxsW3Jvd25hbWVzKGNvdW50c19maWx0ZXJlZCksXSAjcm93Lm5hbWVzKCkNCg0KIyMgU3BlY2llcyBhYnVuZGFuY2UgKHN1bSBvZiBhbGwgY291bnRzKQ0KdG90YWxzX2RmIDwtIGRhdGEuZnJhbWUoKQ0KZm9yIChzcGUgaW4gc3BlY2llcykgew0KICBzcGVfZ2VuZWlkIDwtIGFubm90YXRpb25fdGFibGUkT3JnYW5pc20gPT0gc3BlDQogIGZvciAoc2FtcCBpbiBzYW1wbGVzKSB7DQogICAgdG90YWxzX2RmW3NwZSwgc2FtcF0gPC0gc3VtKGNvdW50c19maWx0ZXJlZFtzcGVfZ2VuZWlkLCBzYW1wXSwgbmEucm09VFJVRSkNCiAgfQ0KfQ0KDQpgYGANCg0KYGBge3Igbm9fc3RhbmRhcmRpc2F0aW9ufQ0KIyBubyBzdGFuZGFyZGlzYXRpb24gd2FzIHVzZWQgYWZ0ZXJhbGwNCmNvdW50c19zdGFuZGFyZCA8LSBjb3VudHNfZmlsdGVyZWQNCnRhYmxlX2dlbmVfZXhwciA8LSBjb3VudHNfdGFibGUNCnRhYmxlX2dlbmVfZXhwciRkZXRlY3RlZCA8LSAiTm8iDQp0YWJsZV9nZW5lX2V4cHJba2VwdF9nZW5lcywgImRldGVjdGVkIl0gPC0gInllcyINCnRhYmxlX2dlbmVfZXhwciA8LSByb3duYW1lc190b19jb2x1bW4odGFibGVfZ2VuZV9leHByKQ0KdGFibGVfZ2VuZV9leHByIDwtIGxlZnRfam9pbih0YWJsZV9nZW5lX2V4cHIsIHJvd25hbWVzX3RvX2NvbHVtbihhbm5vdGF0aW9uX3RhYmxlX2xvbmcpWyxjKCJyb3duYW1lIiwgImxvY3VzX3RhZyIsICJ0cmFuc2NyaXB0SWQiKV0pDQoNCmBgYA0KDQpgYGB7ciB6ZXJvZXNfc21vb3RoaW5nX21ldGF0cmFuc2NyaXB0b21pY3N9DQojIyByZW1haW5pbmcgemVyb2VzIHNtb290aGluZyAoYXMgaW4gWmhhbmcgZXQgYWwuIDIwMjEpDQoNCm1pbl92YWwgPC0gYXBwbHkoY291bnRzX3N0YW5kYXJkLCAxLCBmdW5jdGlvbih4KSAobWluKHhbeD4wXSkvMikpDQoNCg0KZm9yIChnZW5lIGluIHJvdy5uYW1lcyhjb3VudHNfc3RhbmRhcmQpKSB7DQogIGNvdW50c19zdGFuZGFyZFtnZW5lLF1bY291bnRzX3N0YW5kYXJkW2dlbmUsXSA9PSAwXSA8LSBtaW5fdmFsW2dlbmVdDQp9DQoNCmBgYA0KDQpgYGB7ciBzYW1wbGVfd2lzZV9zdGF0c19maWx0ZXJlZH0NCm1lc3NhZ2UoIkNvbXB1dGluZyBzYW1wbGUtd2lzZSBzdGF0aXN0aWNzIG9uIGZpbHRlcmVkIGNvdW50cyIpDQpzYW1wbGVfc3RhdF9maWx0IDwtIGRhdGEuZnJhbWUoDQogIG1lYW4gPSBhcHBseShjb3VudHNfc3RhbmRhcmQsIDIsIG1lYW4sIG5hLnJtID0gVFJVRSksDQogIHNkID0gYXBwbHkoY291bnRzX3N0YW5kYXJkLCAyLCBzZCwgbmEucm0gPSBUUlVFKSwNCiAgaXFyID0gYXBwbHkoY291bnRzX3N0YW5kYXJkLCAyLCBJUVIsIG5hLnJtID0gVFJVRSksDQogIFExID0gYXBwbHkoY291bnRzX3N0YW5kYXJkLCAyLCBxdWFudGlsZSwgcCA9IDAuMjUsIG5hLnJtID0gVFJVRSksDQogIG1lZGlhbiA9IGFwcGx5KGNvdW50c19zdGFuZGFyZCwgMiwgbWVkaWFuLCBuYS5ybSA9IFRSVUUpLA0KICBRMyA9IGFwcGx5KGNvdW50c19zdGFuZGFyZCwgMiwgcXVhbnRpbGUsIHAgPSAwLjc1LCBuYS5ybSA9IFRSVUUpLA0KICBtYXggPSBhcHBseShjb3VudHNfc3RhbmRhcmQsIDIsIG1heCwgbmEucm0gPSBUUlVFKSwNCiAgbnVsbCA9IGFwcGx5KGNvdW50c19zdGFuZGFyZCA9PSAwLCAyLCBzdW0sIG5hLnJtID0gVFJVRSkNCikNCg0Ka2FibGUoc2FtcGxlX3N0YXRfZmlsdFswOjYsIF0sIGNhcHRpb24gPSAiU2FtcGxlLXdpc2Ugc3RhdGlzdGljcyBhZnRlciBmaWx0ZXJpbmciKQ0KYGBgDQoNCiMjIyMgQ0xSIChDZW50ZXJlZCBMb2cgcmF0aW8pIHRyYW5zZm9ybWF0aW9uIG9mIHdob2xlIGFzc2VtYmxhZ2UgbWV0YXRyYW5zY3JpcHRvbWljcyBjb3VudHMNCg0KYGBge3IgQ0xSX3RyYW5zZm9ybWF0aW9ufQ0KDQpjb3VudHNfY2xyIDwtIGNvdW50c19zdGFuZGFyZA0KDQpmb3IgKHNhbXAgaW4gc2FtcGxlcykgew0KICBjb3VudHNfY2xyWyxzYW1wXSA8LSBjbHIoY291bnRzX3N0YW5kYXJkWyxzYW1wXSkNCn0NCmBgYA0KDQpgYGB7ciBzYW1wbGVfd2lzZV9zdGF0c19jbHJ9DQptZXNzYWdlKCJDb21wdXRpbmcgc2FtcGxlLXdpc2Ugc3RhdGlzdGljcyBvbiBmaWx0ZXJlZCBjb3VudHMiKQ0Kc2FtcGxlX3N0YXRfY2xyIDwtIGRhdGEuZnJhbWUoDQogIG1lYW4gPSBhcHBseShjb3VudHNfY2xyLCAyLCBtZWFuLCBuYS5ybSA9IFRSVUUpLA0KICBzZCA9IGFwcGx5KGNvdW50c19jbHIsIDIsIHNkLCBuYS5ybSA9IFRSVUUpLA0KICBpcXIgPSBhcHBseShjb3VudHNfY2xyLCAyLCBJUVIsIG5hLnJtID0gVFJVRSksDQogIFExID0gYXBwbHkoY291bnRzX2NsciwgMiwgcXVhbnRpbGUsIHAgPSAwLjI1LCBuYS5ybSA9IFRSVUUpLA0KICBtZWRpYW4gPSBhcHBseShjb3VudHNfY2xyLCAyLCBtZWRpYW4sIG5hLnJtID0gVFJVRSksDQogIFEzID0gYXBwbHkoY291bnRzX2NsciwgMiwgcXVhbnRpbGUsIHAgPSAwLjc1LCBuYS5ybSA9IFRSVUUpLA0KICBtYXggPSBhcHBseShjb3VudHNfY2xyLCAyLCBtYXgsIG5hLnJtID0gVFJVRSksDQogIG51bGwgPSBhcHBseShjb3VudHNfY2xyID09IDAsIDIsIHN1bSwgbmEucm0gPSBUUlVFKQ0KKQ0KDQprYWJsZShzYW1wbGVfc3RhdF9jbHJbMDo2LCBdLCBjYXB0aW9uID0gIlNhbXBsZS13aXNlIHN0YXRpc3RpY3MgYWZ0ZXIgQ0xSIHRyYW5zZm9ybWF0aW9uIikNCmBgYA0KDQpgYGB7ciBnZW5lX3N0YXRzX25vcm19DQojIyBHZW5lLXdpc2Ugc3RhdGlzdGljcyBhZnRlciBub3JtYWxpc2F0aW9uDQptZXNzYWdlKCJDb21wdXRpbmcgZ2VuZS13aXNlIHN0YXRpc3RpY3Mgb24gbG9nLXRyYW5zZm9ybWVkIGFuZCBub3JtYWxpc2VkIGNvdW50cyIpDQpnZW5lX3N0YXRfbm9ybSA8LSBkYXRhLmZyYW1lKG1lYW4gPSBhcHBseShjb3VudHNfY2xyLCAxLCBtZWFuLCBuYS5ybT1UUlVFKSwNCiAgICAgICAgICAgICAgICAgICAgICAgICAgICAgdmFyID0gYXBwbHkoY291bnRzX2NsciwgMSwgdmFyLCBuYS5ybT1UUlVFKSwNCiAgICAgICAgICAgICAgICAgICAgICAgICAgICAgc2QgPSBhcHBseShjb3VudHNfY2xyLCAxLCBzZCwgbmEucm09VFJVRSksDQogICAgICAgICAgICAgICAgICAgICAgICAgICAgIGlxciA9IGFwcGx5KGNvdW50c19jbHIsIDEsIElRUiwgbmEucm09VFJVRSksDQogICAgICAgICAgICAgICAgICAgICAgICAgICAgIG1pbiA9IGFwcGx5KGNvdW50c19jbHIsIDEsIG1pbiwgbmEucm09VFJVRSksDQogICAgICAgICAgICAgICAgICAgICAgICAgICAgIG1lZCA9IGFwcGx5KGNvdW50c19jbHIsIDEsIG1lZGlhbiwgbmEucm09VFJVRSksDQogICAgICAgICAgICAgICAgICAgICAgICAgICAgIG1heCA9IGFwcGx5KGNvdW50c19jbHIsIDEsIG1heCwgbmEucm09VFJVRSkpDQoNCiMgQWpvdXQgZHUgY29lZmZpY2llbnQgZGUgdmFyaWF0aW9uDQpnZW5lX3N0YXRfbm9ybSRjb2VmX3ZhciA8LSAoZ2VuZV9zdGF0X25vcm0kc2QgLyBnZW5lX3N0YXRfbm9ybSRtZWFuKQ0KDQoNCmBgYA0KDQojIyMjIE1ldGF0cmFuc2NyaXB0b21pY3MgY291bnRzIGRpc3RyaWJ1dGlvbg0KDQpgYGB7ciBoaXN0X3JhdywgZmlnLmhlaWdodD03LCBmaWcud2lkdGg9N30NCnBhcihtZnJvdz1jKDEsMSkpDQpoaXN0KHVubGlzdChjb3VudHNfdGFibGUpLA0KICAgICBicmVha3MgPSAyMDAsDQogICAgIGNleC5heGlzID0gMC43LA0KICAgICBsYXMgPSAxLA0KICAgICBjb2wgPSAic2t5Ymx1ZSIsDQogICAgIHhsYWIgPSAicmF3IGNvdW50cyIsDQogICAgIG1haW4gPSAiRGlzdHJpYnV0aW9uIG9mIHJhdyBjb3VudHMiKQ0KYGBgDQoNCmBgYHtyIGhpc3RfY2xyLCBmaWcuaGVpZ2h0PTcsIGZpZy53aWR0aD03fQ0KcGFyKG1mcm93PWMoMSwxKSkNCmhpc3QodW5saXN0KGNvdW50c19jbHIpLA0KICAgICBicmVha3MgPSAyMDAsDQogICAgIGNleC5heGlzID0gMC43LA0KICAgICBsYXMgPSAxLA0KICAgICBjb2wgPSAic2t5Ymx1ZSIsDQogICAgIHhsYWIgPSAiY2xyLXRyYW5zZm9ybWVkIGNvdW50cyIsDQogICAgIG1haW4gPSAiRGlzdHJpYnV0aW9uIG9mIGNsci10cmFuc2Zvcm1lZCBjb3VudHMiKQ0KYGBgDQoNClRoZSBkaXN0cmlidXRpb24gb2YgdGhlIHdob2xlIGFzc2VtYmxhZ2UgbWV0YXRyYW5zY3JpcHRvbWljcyBjb3VudHMgc2VlbXMgY2xvc2UgdG8gbm9ybWFsLg0KDQpgYGB7ciBib3hfcGxvdF9yYXcsIGZpZy5oZWlnaHQ9NywgZmlnLndpZHRoPTd9DQojIFJhdyBkYXRhDQpib3hwbG90KGNvdW50c190YWJsZSwNCiAgICAgICAgbWFpbiA9ICJSYXcgZXhwcmVzc2lvbiIsDQogICAgICAgIGhvcml6b250YWwgPSBUUlVFLA0KICAgICAgICBjb2wgPSBtZXRhZGF0YV90YWJsZSRjb2xvciwNCiAgICAgICAgY2V4ID0gMC41LA0KICAgICAgICBjZXguYXhpcyA9IDAuOCwNCiAgICAgICAgbGFzID0gMSkNCmBgYA0KDQpgYGB7ciBib3hfcGxvdF9jbHIsIGZpZy5oZWlnaHQ9NywgZmlnLndpZHRoPTd9DQpib3hwbG90KGNvdW50c19jbHIsDQogICAgICAgIG1haW4gPSAiY2xyLXRyYW5zZm9ybWVkIGV4cHJlc3Npb24iLA0KICAgICAgICBob3Jpem9udGFsID0gVFJVRSwNCiAgICAgICAgY29sID0gbWV0YWRhdGFfdGFibGUkY29sb3IsDQogICAgICAgIGNleCA9IDAuNSwNCiAgICAgICAgY2V4LmF4aXMgPSAwLjgsDQogICAgICAgIGxhcyA9IDEpDQpgYGANCg0KDQpUaGUgQ0xSIHRyYW5zZm9ybWF0aW9uIGNlbnRyZWQgb3VyIGRhdGEuDQoNCg0KIyMjIyBBc3NlbWJsYWdlIG1ldGF0cmFuc2NyaXB0b21pY3MgY291bnRzIHJlcGFydGl0aW9uIGJ5IHNwZWNpZXMNCg0KYGBge3Igc3BlY2llc19yZXBhcnRpdGlvbl90YWJsZX0NCnRvdGFsc19kZiRzcGVjaWVzIDwtIHJvdy5uYW1lcygodG90YWxzX2RmKSkNCnRvdGFsc19kZiAlPiUNCiAgICBwaXZvdF9sb25nZXIoIXNwZWNpZXMsIA0KICAgICAgICAgICAgICAgIG5hbWVzX3RvPWMoInNhbXBsZSIpLA0KICAgICAgICAgICAgICAgIHZhbHVlc190bz0idG90YWxfY291bnRzIikgJT4lDQogIG11dGF0ZShzYW1wbGUgPSBjYXNlX3doZW4oc2FtcGxlID09ICJTRF9URl8xIiB+ICJTRF9URl8xIiwNCiAgICAgICAgICAgICAgICAgICAgICAgICAgICBzYW1wbGUgPT0gIlNEX1RGXzIiIH4gIlNEX1RGXzIiLA0KICAgICAgICAgICAgICAgICAgICAgICAgICAgIHNhbXBsZSA9PSAiU0RfVEZfMyIgfiAiU0RfVEZfMyIsDQogICAgICAgICAgICAgICAgICAgICAgICAgICAgc2FtcGxlID09ICJXTl9URl8xIiB+ICJXTl9URl8xIiwNCiAgICAgICAgICAgICAgICAgICAgICAgICAgICBzYW1wbGUgPT0gIldOX1RGXzIiIH4gIldOX1RGXzIiLA0KICAgICAgICAgICAgICAgICAgICAgICAgICAgIHNhbXBsZSA9PSAiV05fVEZfMyIgfiAiV05fVEZfMyIpKSAtPiB0b3RhbHNfZGYNCmBgYA0KDQpgYGB7ciBzcGVjaWVzX3JlcGFydGl0aW9uX3Bsb3QsIGZpZy5oZWlnaHQ9NywgZmlnLndpZHRoPTd9DQoNCg0KZ2dwbG90KGRhdGE9dG90YWxzX2RmLCBtYXBwaW5nPWFlcyh4PXNhbXBsZSwgeT10b3RhbF9jb3VudHMsIGZpbGw9c3BlY2llcykpICsgDQogIGdlb21fYmFyKHBvc2l0aW9uPSJmaWxsIiwgc3RhdD0iaWRlbnRpdHkiKSArIA0KICBzY2FsZV9maWxsX2JyZXdlcihwYWxldHRlID0gIlB1T3IiKSArDQogIGxhYnMoeSA9ICJQZXJjZW50YWdlIG9mIHRvdGFsIGNvdW50cyIpICsNCiAgc2NhbGVfeV9jb250aW51b3VzKGxhYmVscyA9IGFzX2Z1bmN0aW9uKH4gMTAwICogLikpICsgDQogIHRoZW1lKGF4aXMudGV4dC54ID0gZWxlbWVudF90ZXh0KGNvbG91ciA9IGMoIlNEX1RGXzEiID0gImRhcmtvcmFuZ2UiLA0KICAgICAgICAgICAgICAgICAgICAgICAgICAgICAgICAgICAgICAgICAgICAgICJTRF9URl8yIiA9ICJkYXJrb3JhbmdlIiwNCiAgICAgICAgICAgICAgICAgICAgICAgICAgICAgICAgICAgICAgICAgICAgICAiU0RfVEZfMyIgPSAiZGFya29yYW5nZSIsDQogICAgICAgICAgICAgICAgICAgICAgICAgICAgICAgICAgICAgICAgICAgICAgIldOX1RGXzEiID0gImRhcmtjeWFuIiwNCiAgICAgICAgICAgICAgICAgICAgICAgICAgICAgICAgICAgICAgICAgICAgICAiV05fVEZfMiIgPSAiZGFya2N5YW4iLA0KICAgICAgICAgICAgICAgICAgICAgICAgICAgICAgICAgICAgICAgICAgICAgICJXTl9URl8zIiA9ICJkYXJrY3lhbiIpKSkgKyANCiAgZ2d0aXRsZSgiVG90YWwgbWV0YXRyYW5zY3JpcHRvbWljcyBjb3VudHMgcGVyIHNwZWNpZXMiKSAtPiBwIA0KDQpwcmludChwKQ0KDQpnZ3NhdmUoIi4uL2ZpZ3VyZXMvRmlndXJlX1MzX2NvdW50c19yZXBhcnRpdGlvbi5wbmciLCBwLCBkcGkgPSAzMDApDQpnZ3NhdmUoIi4uL2ZpZ3VyZXMvRmlndXJlX1MzX2NvdW50c19yZXBhcnRpdGlvbi50aWZmIiwgcCwgaGVpZ2h0ID0gNywgd2lkdGggPSA3LCBkcGkgPSAzMDApDQpgYGANCg0KQXMgcHJldmlvdXNseSBub3RlZCwgKkRpb3N6ZWdpYSBodW5nYXJpY2EqIGNvdW50cyBhcmUgbGFyZ2VseSBkb21pbmFudCBpbiBvdXIgZGF0YS4gKlBzZXVkb21vbmFzIGdyYW1pbmlzKiBhc3NvY2lhdGVkIGNvdW50cyBhcmUgdmVyeSByYXJlLg0KDQojIyMjIyAqRC4gaHVuZ2FyaWNhKiBmaWx0ZXJlZCBjb3VudHMgcmVwYXJ0aXRpb24gYnkgbnVjbGV1cyBvciBtaXRvY2hvbmRyaWFsIG9yaWdpbg0KDQpgYGB7ciBkaW9zemVnaWFfZ2Vub21lX29yaWdpbl90cmFuc2NyaXB0cywgZmlnLmhlaWdodD03LCBmaWcud2lkdGg9N30NCg0Kb3JnYW5lbGxlX2RmIDwtIGRhdGEuZnJhbWUoKQ0KDQptaXRvX2dlbmVpZCA8LSBhbm5vdGF0aW9uX3RhYmxlJENociA9PSAiRGlvc3plZ2lhX2h1bmdhcmljYV9QREQtMjRiLTJfY29udGlnXzM1Ig0KbnVjbF9nZW5laWQgPC0gKGFubm90YXRpb25fdGFibGUkT3JnYW5pc20gPT0gIkQuaHVuZ2FyaWNhIiAmIGFubm90YXRpb25fdGFibGUkQ2hyICE9ICJEaW9zemVnaWFfaHVuZ2FyaWNhX1BERC0yNGItMl9jb250aWdfMzUiKQ0KDQpmb3IgKHNhbXAgaW4gc2FtcGxlcykgew0KICAgIG9yZ2FuZWxsZV9kZlsibWl0b2Nob25kcmlhIiwgc2FtcF0gPC0gc3VtKGNvdW50c19maWx0ZXJlZFttaXRvX2dlbmVpZCwgc2FtcF0sIG5hLnJtPVRSVUUpDQogICAgb3JnYW5lbGxlX2RmWyJudWNsZXVzIiwgc2FtcF0gPC0gc3VtKGNvdW50c19maWx0ZXJlZFtudWNsX2dlbmVpZCwgc2FtcF0sIG5hLnJtPVRSVUUpDQp9DQoNCm9yZ2FuZWxsZV9kZiRvcmdhbmVsbGUgPC0gcm93Lm5hbWVzKChvcmdhbmVsbGVfZGYpKQ0Kb3JnYW5lbGxlX2RmICU+JQ0KICAgIHBpdm90X2xvbmdlcighb3JnYW5lbGxlLCANCiAgICAgICAgICAgICAgICBuYW1lc190bz1jKCJzYW1wbGUiKSwNCiAgICAgICAgICAgICAgICB2YWx1ZXNfdG89InRvdGFsX2NvdW50cyIpICU+JQ0KICBtdXRhdGUoc2FtcGxlID0gY2FzZV93aGVuKHNhbXBsZSA9PSAiU0RfVEZfMSIgfiAiU0RfVEZfMSIsDQogICAgICAgICAgICAgICAgICAgICAgICAgICAgc2FtcGxlID09ICJTRF9URl8yIiB+ICJTRF9URl8yIiwNCiAgICAgICAgICAgICAgICAgICAgICAgICAgICBzYW1wbGUgPT0gIlNEX1RGXzMiIH4gIlNEX1RGXzMiLA0KICAgICAgICAgICAgICAgICAgICAgICAgICAgIHNhbXBsZSA9PSAiV05fVEZfMSIgfiAiV05fVEZfMSIsDQogICAgICAgICAgICAgICAgICAgICAgICAgICAgc2FtcGxlID09ICJXTl9URl8yIiB+ICJXTl9URl8yIiwNCiAgICAgICAgICAgICAgICAgICAgICAgICAgICBzYW1wbGUgPT0gIldOX1RGXzMiIH4gIldOX1RGXzMiKSkgLT4gb3JnYW5lbGxlX2RmDQoNCmdncGxvdChkYXRhPW9yZ2FuZWxsZV9kZiwgbWFwcGluZz1hZXMoeD1zYW1wbGUsIHk9dG90YWxfY291bnRzLCBmaWxsPW9yZ2FuZWxsZSkpICsgDQogIGdlb21fYmFyKHBvc2l0aW9uPSJmaWxsIiwgc3RhdD0iaWRlbnRpdHkiKSArIA0KICBzY2FsZV9maWxsX2JyZXdlcihwYWxldHRlID0gIlB1T3IiKSArDQogIGxhYnMoeSA9ICJQZXJjZW50YWdlIG9mIHRvdGFsIGNvdW50cyIpICsNCiAgc2NhbGVfeV9jb250aW51b3VzKGxhYmVscyA9IGFzX2Z1bmN0aW9uKH4gMTAwICogLikpICsgDQogIHRoZW1lKGF4aXMudGV4dC54ID0gZWxlbWVudF90ZXh0KGNvbG91ciA9IGMoIlNEX1RGXzEiID0gImRhcmtvcmFuZ2UiLA0KICAgICAgICAgICAgICAgICAgICAgICAgICAgICAgICAgICAgICAgICAgICAgICJTRF9URl8yIiA9ICJkYXJrb3JhbmdlIiwNCiAgICAgICAgICAgICAgICAgICAgICAgICAgICAgICAgICAgICAgICAgICAgICAiU0RfVEZfMyIgPSAiZGFya29yYW5nZSIsDQogICAgICAgICAgICAgICAgICAgICAgICAgICAgICAgICAgICAgICAgICAgICAgIldOX1RGXzEiID0gImRhcmtjeWFuIiwNCiAgICAgICAgICAgICAgICAgICAgICAgICAgICAgICAgICAgICAgICAgICAgICAiV05fVEZfMiIgPSAiZGFya2N5YW4iLA0KICAgICAgICAgICAgICAgICAgICAgICAgICAgICAgICAgICAgICAgICAgICAgICJXTl9URl8zIiA9ICJkYXJrY3lhbiIpKSkgKyANCiAgZ2d0aXRsZSgiVG90YWwgdHJhbnNjcmlwdCBjb3VudHMgcGVyIEQuIGh1bmdhcmljYSBjb21wYXJ0bWVudCIpIC0+IHAgDQoNCnByaW50KHApDQoNCmdnc2F2ZSgiLi4vZmlndXJlcy9EaW9odV9jb3VudHNfcmVwYXJ0aXRpb24ucG5nIiwgcCwgZHBpID0gMzAwKQ0KZ2dzYXZlKCIuLi9maWd1cmVzL0Rpb2h1X2NvdW50c19yZXBhcnRpdGlvbi50aWZmIiwgcCwgaGVpZ2h0ID0gNywgd2lkdGggPSA3LCBkcGkgPSAzMDApDQoNCmBgYA0KDQoNCioqQW1vbmcgKkRpb3N6ZWdpYSBodW5nYXJpY2EqIGNvdW50cywgYSBsYXJnZSBwb3J0aW9uIGNvbWUgZnJvbSB0cmFuc2NyaXB0cyBwcm9kdWNlZCBpbiB0aGUgbWl0b2Nob25kcmlhLCBlc3BlY2lhbGx5IGluIFNEIGNvbmRpdGlvbnMuKioNCg0KDQojIyMgTWV0YWJvbG9taWNzDQoNCiMjIyMgTWV0YWJvbGl0ZS13aXNlIHN0YXRpc3RpY3MgKHJhdyBkYXRhKQ0KDQpgYGB7ciBtZXRhYm9saXRlLXdpc2Vfc3RhdHN9DQoNCiMgbWVzc2FnZSgiQ29tcHV0aW5nIG1ldGFib2xpdGUtd2lzZSBzdGF0aXN0aWNzIG9uIHJhdyBjb3VudHMiKQ0KDQptZXRhYm9saXRlX3N0YXRfcHJlbm9ybSA8LSBkYXRhLmZyYW1lKA0KICBtZWFuID0gYXBwbHkobWV0YWJvbG9taWNzX2FsbF90aW1lc19kZiwgMSwgbWVhbiwgbmEucm0gPSBUUlVFKSwNCiAgc2QgPSBhcHBseShtZXRhYm9sb21pY3NfYWxsX3RpbWVzX2RmLCAxLCBzZCwgbmEucm0gPSBUUlVFKSwNCiAgaXFyID0gYXBwbHkobWV0YWJvbG9taWNzX2FsbF90aW1lc19kZiwgMSwgSVFSLCBuYS5ybSA9IFRSVUUpLA0KICBRMSA9IGFwcGx5KG1ldGFib2xvbWljc19hbGxfdGltZXNfZGYsIDEsIHF1YW50aWxlLCBwID0gMC4yNSwgbmEucm0gPSBUUlVFKSwNCiAgbWVkaWFuID0gYXBwbHkobWV0YWJvbG9taWNzX2FsbF90aW1lc19kZiwgMSwgbWVkaWFuLCBuYS5ybSA9IFRSVUUpLA0KICBRMyA9IGFwcGx5KG1ldGFib2xvbWljc19hbGxfdGltZXNfZGYsIDEsIHF1YW50aWxlLCBwID0gMC43NSwgbmEucm0gPSBUUlVFKSwNCiAgbWF4ID0gYXBwbHkobWV0YWJvbG9taWNzX2FsbF90aW1lc19kZiwgMSwgbWF4LCBuYS5ybSA9IFRSVUUpLA0KICBudWxsID0gYXBwbHkobWV0YWJvbG9taWNzX2FsbF90aW1lc19kZiA9PSAwLCAxLCBzdW0sIG5hLnJtID0gVFJVRSkNCikNCg0Ka2FibGUobWV0YWJvbGl0ZV9zdGF0X3ByZW5vcm1bMTAwOjEwOSwgXSwgY2FwdGlvbiA9ICJHZW5lLXdpc2Ugc3RhdGlzdGljcyBiZWZvcmUgbm9ybWFsaXNhdGlvbiIpDQpgYGANCg0KIyMjIyBaZXJvIGZpbHRlcmluZyBhbmQgc21vb3RoaW5nIG1ldGFib2xvbWljcw0KDQpNZXRhYm9saXRlcyB3aXRoIDAgY291bnRzIGluIG1vcmUgdGhhbiA3MCUgb2Ygc2FtcGxlcyBhcmUgZGVlbWVkIHVuZXhwcmVzc2VkIGFuZCBhcmUgcmVtb3ZlZCBmcm9tIHRoZSBkYXRhc2V0LiAgDQoNCg0KDQpgYGB7ciBNZXRhYm9sb21pY3NfYWxsX3RpbWVzX3plcm9fZmlsdGVyaW5nLCByZXN1bHRzPSdhc2lzJ30NCg0KbWVzc2FnZSgiRmlsdGVyaW5nIHVuZGV0ZWN0ZWQgbWV0YWJvbGl0ZXMiKQ0KdW5kZXRlY3RlZF9tZXRhYm9saXRlcyA8LSBtZXRhYm9saXRlX3N0YXRfcHJlbm9ybSRudWxsID49IG5jb2wobWV0YWJvbG9taWNzX2FsbF90aW1lc19kZikgKiAwLjcwDQpwcmludChwYXN0ZTAoInVuZGV0ZWN0ZWRfbWV0YWJvbGl0ZXMgKG51bGwgaW4gPj0gNzAlIHNhbXBsZXMpOiAiLCBzdW0odW5kZXRlY3RlZF9tZXRhYm9saXRlcykpKQ0KDQprZXB0X21ldGFib2xpdGVzIDwtICF1bmRldGVjdGVkX21ldGFib2xpdGVzDQpwcmludChwYXN0ZTAoIktlcHQgbWV0YWJvbGl0ZXM6ICIsIHN1bShrZXB0X21ldGFib2xpdGVzKSkpDQoNCiMjIG1ldGFib2xpdGVzIGFmdGVyIGZpbHRlcmluZw0KbWV0YWJvbG9taWNzX2FsbF90aW1lc19maWx0ZXJlZCA8LSBtZXRhYm9sb21pY3NfYWxsX3RpbWVzX2RmW2tlcHRfbWV0YWJvbGl0ZXMsIF0NCg0KYGBgDQoNCg0KYGBge3IgTWV0YWJvbG9taWNzX2FsbF90aW1lc196ZXJvZXNfc21vb3RoaW5nfQ0KIyMgcmVtYWluaW5nIHplcm9lcyBzbW9vdGhpbmcgKGFzIGluIFpoYW5nIGV0IGFsLiAyMDIxKQ0KDQptaW5fdmFsIDwtIGFwcGx5KG1ldGFib2xvbWljc19hbGxfdGltZXNfZmlsdGVyZWQsIDEsIGZ1bmN0aW9uKHgpIChtaW4oeFt4PjBdKS8yKSkNCg0KDQpmb3IgKG1ldGFib2xpdGUgaW4gcm93Lm5hbWVzKG1ldGFib2xvbWljc19hbGxfdGltZXNfZmlsdGVyZWQpKSB7DQogIG1ldGFib2xvbWljc19hbGxfdGltZXNfZmlsdGVyZWRbbWV0YWJvbGl0ZSxdW21ldGFib2xvbWljc19hbGxfdGltZXNfZmlsdGVyZWRbbWV0YWJvbGl0ZSxdID09IDBdIDwtIG1pbl92YWxbbWV0YWJvbGl0ZV0NCn0NCg0KYGBgDQoNCiMjIyMgQ0xSIHRyYW5zZm9ybWF0aW9uIG1ldGFib2xvbWljcw0KDQpgYGB7ciBtZXRhYm9sb21pY3NfYWxsX3RpbWVzX0NMUn0NCm1ldGFib2xvbWljc19hbGxfdGltZXNfY2xyIDwtIG1ldGFib2xvbWljc19hbGxfdGltZXNfZmlsdGVyZWQNCg0KZm9yIChzYW1wIGluIG5hbWVzKG1ldGFib2xvbWljc19hbGxfdGltZXNfZmlsdGVyZWQgKSl7DQogIG1ldGFib2xvbWljc19hbGxfdGltZXNfY2xyWyxzYW1wXSA8LSBhcy52ZWN0b3IoY2xyKG1ldGFib2xvbWljc19hbGxfdGltZXNfZmlsdGVyZWRbLHNhbXBdKSkNCn0NCmBgYA0KDQoNCg0KIyMjIyBNZXRhYm9sb21pY3MgZGF0YSByZXBhcnRpdGlvbg0KDQpgYGB7ciBoaXN0X21ldGFCX3JhdywgZmlnLmhlaWdodD03LCBmaWcud2lkdGg9N30NCnBhcihtZnJvdz1jKDEsMSkpDQpoaXN0KHVubGlzdChtZXRhYm9sb21pY3NfYWxsX3RpbWVzX2RmKSwNCiAgICAgYnJlYWtzID0gMjAwLA0KICAgICBjZXguYXhpcyA9IDAuNywNCiAgICAgbGFzID0gMSwNCiAgICAgY29sID0gInNreWJsdWUiLA0KICAgICB4bGFiID0gInJhdyBtZXRhYm9sb21pY3MgZGF0YSIsDQogICAgIG1haW4gPSAiRGlzdHJpYnV0aW9uIG9mIHJhdyBtZXRhYm9sb21pY3MgZGF0YSIpDQpgYGANCg0KDQoNCmBgYHtyIGhpc3RfbWV0YUJfYWxsX3RpbWVzX2NsciwgZmlnLmhlaWdodD03LCBmaWcud2lkdGg9N30NCnBhcihtZnJvdz1jKDEsMSkpDQpoaXN0KHVubGlzdChtZXRhYm9sb21pY3NfYWxsX3RpbWVzX2NsciksDQogICAgIGJyZWFrcyA9IDIwMCwNCiAgICAgY2V4LmF4aXMgPSAwLjcsDQogICAgIGxhcyA9IDEsDQogICAgIGNvbCA9ICJza3libHVlIiwNCiAgICAgeGxhYiA9ICJjbHItdHJhbnNmb3JtZWQgbWV0YWJvbG9taWNzIGRhdGEsIGFsbCB0aW1lcyIsDQogICAgIG1haW4gPSAiRGlzdHJpYnV0aW9uIG9mIGNsci10cmFuc2Zvcm1lZCBtZXRhYm9sb21pY3MgZGF0YSwgYWxsIHRpbWVzIikNCmBgYA0KDQpUaGUgZGlzdHJpYnV0aW9uIG9mIG1ldGFib2xvbWljcyBkYXRhIGlzIGNsb3NlIHRvIG5vcm1hbC4NCg0KDQpgYGB7ciBib3hfcGxvdF9tZXRhQl9yYXcsIGZpZy5oZWlnaHQ9NywgZmlnLndpZHRoPTd9DQpib3hwbG90KG1ldGFib2xvbWljc19hbGxfdGltZXNfZGYsDQogICAgICAgIG1haW4gPSAibWV0YWJvbG9taWNzIHJhdyBkYXRhIiwNCiAgICAgICAgaG9yaXpvbnRhbCA9IFRSVUUsDQogICAgICAgIGNvbCA9IG1ldGFkYXRhX3RhYmxlX21ldGFCX2FsbF90aW1lcyRjb2xvciwNCiAgICAgICAgY2V4ID0gMC41LA0KICAgICAgICBjZXguYXhpcyA9IDAuOCwNCiAgICAgICAgbGFzID0gMSkNCmBgYA0KDQpgYGB7ciBib3hfcGxvdF9tZXRhQl9jbHIsIGZpZy5oZWlnaHQ9NywgZmlnLndpZHRoPTd9DQpib3hwbG90KG1ldGFib2xvbWljc19hbGxfdGltZXNfY2xyLA0KICAgICAgICBtYWluID0gIm1ldGFib2xvbWljcyBjbHItdHJhbnNmb3JtZWQgZXhwcmVzc2lvbiIsDQogICAgICAgIGhvcml6b250YWwgPSBUUlVFLA0KICAgICAgICBjb2wgPSBtZXRhZGF0YV90YWJsZV9tZXRhQl9hbGxfdGltZXMkY29sb3IsDQogICAgICAgIGNleCA9IDAuNSwNCiAgICAgICAgY2V4LmF4aXMgPSAwLjgsDQogICAgICAgIGxhcyA9IDEpDQpgYGANCg0KDQpUaGUgQ0xSIHRyYW5zZm9ybWF0aW9uIGNlbnRyZWQgb3VyIGRhdGEuDQoNCg0KDQojIyBFeHBsb3JhdG9yeSBzdGF0aXN0aWNhbCBhbmFseXNlcyBvZiB0aGUgd2hvbGUgYXNzZW1ibGFnZQ0KDQojIyMgUHJpbmNpcGFsIENvbXBvbmVudCBBbmFseXNlcyAoUENBKSBtZXRhdHJhbnNjcmlwdG9taWMNCg0KYGBge3IgUENBX2NsciwgZmlnLmhlaWdodD03LCBmaWcud2lkdGg9N30NCnBhcihtZnJvdyA9IGMoMiwyKSkNCnJlc19wY2EgPC0gUENBKHQoY291bnRzX2NsciksIHNjYWxlLnVuaXQgPSBUUlVFLCBncmFwaCA9IEZBTFNFKQ0KYGBgDQoNCmBgYHtyIFBFUk1BTk9WQV9hc3NlbWJsYWdlfQ0KY291bnRzX2RhdGEgPC0gdChjb3VudHNfY2xyKQ0KbWV0YWRhdGFfdG1wIDwtIGRhdGEuZnJhbWUobWV0YWRhdGFfdGFibGUsIHN0cmluZ3NBc0ZhY3RvcnMgPSBUUlVFKQ0KcGVybWFub3ZhIDwtIGFkb25pczIoZm9ybXVsYSA9IGFzLmZvcm11bGEocGFzdGUwKCJjb3VudHNfZGF0YX4iLCAiY29uZGl0aW9uIikpLCANCiAgICAgICAgICAgICAgICAgICAgIGRhdGEgPSBtZXRhZGF0YV90bXAsIG1ldGhvZD0iZXVjbGlkZWFuIiwgIyBtZXRob2Q9ImJyYXkiLCAjb3R1X2RhdGF+VElNRQ0KICAgICAgICAgICAgICAgICAgICAgcGVybXV0YXRpb25zID0gOTk5LCBzcXJ0LmRpc3QgPSBGQUxTRSwgYWRkID0gRkFMU0UsIGJ5ID0gInRlcm1zIikNCnB2ID0gcGVybWFub3ZhJGBQcig+RilgWzFdDQojIHByaW50KHB2KQ0KDQpybShjb3VudHNfZGF0YSwgbWV0YWRhdGFfdG1wKQ0KYGBgDQoNCmBgYHtyIFBDQV8yRF9wbG90c19hc3NlbWJsYWdlLCBmaWcuaGVpZ2h0PTQsIGZpZy53aWR0aD05fQ0KDQpwY2FfdG1wIDwtIHJiaW5kKHJlc19wY2EkaW5kJGNvb3JkLA0KICAgICAgICAgICAgICAgICByZXNfcGNhJGluZC5zdXAkY29vcmQpICU+JSBkYXRhLmZyYW1lKCkgJT4lIA0KICByb3duYW1lc190b19jb2x1bW4oInNhbXBsZSIpDQoNCg0KcGNhX2RhdGEgPC0gbGVmdF9qb2luKHBjYV90bXAsIHJvd25hbWVzX3RvX2NvbHVtbihtZXRhZGF0YV90YWJsZSksIGJ5PWpvaW5fYnkoInNhbXBsZSIgPT0gInJvd25hbWUiKSkNCnJtKHBjYV90bXApDQoNCkQxX3RleHQgPSBwYXN0ZTAoIkRpbSAxICgiLCANCiAgICAgICAgICAgICAgICAgcm91bmQoZGF0YS5mcmFtZShyZXNfcGNhJGVpZykkcGVyY2VudGFnZS5vZi52YXJpYW5jZVsxXSwgZGlnaXRzPTIpLCANCiAgICAgICAgICAgICAgICAgIiUpIikNCkQyX3RleHQgPSBwYXN0ZTAoIkRpbSAyICgiLCANCiAgICAgICAgICAgICAgICAgcm91bmQoZGF0YS5mcmFtZShyZXNfcGNhJGVpZykkcGVyY2VudGFnZS5vZi52YXJpYW5jZVsyXSwgZGlnaXRzPTIpLCANCiAgICAgICAgICAgICAgICAgIiUpIikNCkQzX3RleHQgPSBwYXN0ZTAoIkRpbSAzICgiLCANCiAgICAgICAgICAgICAgICAgcm91bmQoZGF0YS5mcmFtZShyZXNfcGNhJGVpZykkcGVyY2VudGFnZS5vZi52YXJpYW5jZVszXSwgZGlnaXRzPTIpLCANCiAgICAgICAgICAgICAgICAgIiUpIikNCg0KZ2dwbG90KHBjYV9kYXRhKSArDQogIGdlb21fcG9pbnQoYWVzKHg9RGltLjEsIHk9RGltLjIsIGNvbG91cj1jb2xvciwgKSwgc2l6ZT0zKSArDQogIHNjYWxlX2NvbG91cl9pZGVudGl0eSgiQ29uZGl0aW9uIiwgbGFiZWxzPWMoIldpbnRlciBOaWdodCIsICJTdW1tZXIgRGF5IiksIGd1aWRlID0gImxlZ2VuZCIpICsNCiAgZ2dyZXBlbDo6Z2VvbV90ZXh0X3JlcGVsKGFlcyh4PURpbS4xLCB5PURpbS4yLCBjb2xvdXI9Y29sb3IsIGxhYmVsPXNhbXBsZSksIA0KICAgICAgICAgICAgICAgICAgICAgICAgICAgbnVkZ2VfeSA9IC0yLCAgc2VlZCA9IDQyLCBzZWdtZW50LnNpemU9MC4xLCBzaG93LmxlZ2VuZCA9IEZBTFNFKSArDQogIHhsYWIoRDFfdGV4dCkgKyANCiAgeWxhYihEMl90ZXh0KSArDQogIGdndGl0bGUoIlBDQSBtZXRhdHJhbnNjcmlwdG9taWNzIikgKw0KICBsYWJzKGNhcHRpb24gPSAgcGFzdGUwKCJQRVJNQU5PVkEgb24gY29uZGl0aW9uOiBwLXZhbHVlOiAiLCBwdikpICsgDQogIHRoZW1lKHBhbmVsLmJhY2tncm91bmQ9IGVsZW1lbnRfcmVjdChmaWxsPSIjZjRmNGY0IiksDQogICAgICAgIHBsb3QuY2FwdGlvbiA9IGVsZW1lbnRfdGV4dChzaXplPTcpKSAtPiBwMV9tZXRhVA0KIA0KDQpnZ3Bsb3QocGNhX2RhdGEpICsNCiAgZ2VvbV9wb2ludChhZXMoeD1EaW0uMSwgeT1EaW0uMywgY29sb3VyPWNvbG9yKSwgc2l6ZT0zKSArDQogIHNjYWxlX2NvbG91cl9pZGVudGl0eSgiQ29uZGl0aW9uIiwgbGFiZWxzPWMoIldpbnRlciBOaWdodCIsICJTdW1tZXIgRGF5IiksIGd1aWRlID0gImxlZ2VuZCIpICsNCiAgZ2dyZXBlbDo6Z2VvbV90ZXh0X3JlcGVsKGFlcyh4PURpbS4xLCB5PURpbS4zLCBjb2xvdXI9Y29sb3IsIGxhYmVsPXNhbXBsZSksIA0KICAgICAgICAgICAgICAgICAgICAgICAgICAgbnVkZ2VfeSA9IC0yLCAgc2VlZCA9IDQyLCBzZWdtZW50LnNpemU9MC4xLCBzaG93LmxlZ2VuZCA9IEZBTFNFKSArDQogIHhsYWIoRDFfdGV4dCkgKyANCiAgeWxhYihEM190ZXh0KSArDQogIGdndGl0bGUoIlBDQSBtZXRhdHJhbnNjcmlwdG9taWNzIikgKw0KICBsYWJzKGNhcHRpb24gPSAgcGFzdGUwKCJQRVJNQU5PVkEgb24gY29uZGl0aW9uOiBwLXZhbHVlOiAiLCBwdikpICsgDQogIHRoZW1lKHBhbmVsLmJhY2tncm91bmQ9IGVsZW1lbnRfcmVjdChmaWxsPSIjZjRmNGY0IiksDQogICAgICAgIHBsb3QuY2FwdGlvbiA9IGVsZW1lbnRfdGV4dChzaXplPTcpKSAtPiBwMg0KDQpnZ3Bsb3QocGNhX2RhdGEpICsNCiAgZ2VvbV9wb2ludChhZXMoeD1EaW0uMiwgeT1EaW0uMywgY29sb3VyPWNvbG9yKSwgc2l6ZT0zKSArDQogIHNjYWxlX2NvbG91cl9pZGVudGl0eSgiQ29uZGl0aW9uIiwgbGFiZWxzPWMoIldpbnRlciBOaWdodCIsICJTdW1tZXIgRGF5IiksIGd1aWRlID0gImxlZ2VuZCIpICsNCiAgZ2dyZXBlbDo6Z2VvbV90ZXh0X3JlcGVsKGFlcyh4PURpbS4yLCB5PURpbS4zLCBjb2xvdXI9Y29sb3IsIGxhYmVsPXNhbXBsZSksIA0KICAgICAgICAgICAgICAgICAgICAgICAgICAgbnVkZ2VfeSA9IC0yLCAgc2VlZCA9IDQyLCBzZWdtZW50LnNpemU9MC4xLCBzaG93LmxlZ2VuZCA9IEZBTFNFKSArDQogIHhsYWIoRDJfdGV4dCkgKyANCiAgeWxhYihEM190ZXh0KSArDQogIGdndGl0bGUoIlBDQSBtZXRhdHJhbnNjcmlwdG9taWNzIikgKw0KICBsYWJzKGNhcHRpb24gPSAgcGFzdGUwKCJQRVJNQU5PVkEgb24gY29uZGl0aW9uOiBwLXZhbHVlOiAiLCBwdikpICsgDQogIHRoZW1lKHBhbmVsLmJhY2tncm91bmQ9IGVsZW1lbnRfcmVjdChmaWxsPSIjZjRmNGY0IiksDQogICAgICAgIHBsb3QuY2FwdGlvbiA9IGVsZW1lbnRfdGV4dChzaXplPTcpKSAtPiBwMw0KDQpwbG90Z3JpZF9QQ0EgPC0gZ2dhcnJhbmdlKHAxX21ldGFULCBwMiwgcDMsIGxhYmVscyA9IGMoIkEiLCAiQiIsICJDIiksIGNvbW1vbi5sZWdlbmQ9VFJVRSwgbGVnZW5kPSJib3R0b20iLCBucm93PTEpIA0KDQpwcmludChwbG90Z3JpZF9QQ0EpDQoNCmdnc2F2ZShwbG90ID0gcGxvdGdyaWRfUENBLCBmaWxlbmFtZSA9ICIuLi9maWd1cmVzL1BDQV9tZXRhdHJhbnNjcmlwdG9taWNzXzNfY29tcG9zYW50ZXMudGlmZiIsIGRwaSA9IDMwMCwgd2lkdGggPSAxNSwgaGVpZ2h0ID0gNSwgYmc9IndoaXRlIikNCg0KZ2dzYXZlKHBsb3Q9cDFfbWV0YVQsIGZpbGVuYW1lID0gIi4uL2ZpZ3VyZXMvUENBX21ldGF0cmFuc2NyaXB0b21pY3MudGlmZiIsIGRwaSA9IDMwMCwgd2lkdGggPSA2LCBoZWlnaHQgPSA1LCBiZz0id2hpdGUiKQ0KYGBgDQoNCg0KYGBge3IgM0RfUENBX2Nsciwgd2ViZ2w9VFJVRSwgZmlnLmhlaWdodD03LCBmaWcud2lkdGg9NywgbWVzc2FnZT1GQUxTRSwgZXZhbD1UUlVFfQ0KDQpyb3dfY29vcmQgPC0gcmVzX3BjYSRpbmQkY29vcmQNCg0KaW52aXNpYmxlKHJnbDo6b3BlbjNkKCkpDQoNCiNyZ2w6OmJnM2QoImxpZ2h0Z3JheSIpDQpyZ2w6OnBsb3QzZCh4PXJvd19jb29yZFtjKCJXTl9URl8xIiwgIldOX1RGXzIiLCAiV05fVEZfMyIpLCAxXSwgDQogICAgICAgICAgICB5PXJvd19jb29yZFtjKCJXTl9URl8xIiwgIldOX1RGXzIiLCAiV05fVEZfMyIpLCAyXSwgDQogICAgICAgICAgICB6PXJvd19jb29yZFtjKCJXTl9URl8xIiwgIldOX1RGXzIiLCAiV05fVEZfMyIpLCAzXSwNCiAgICAgICAgICAgIHhsYWIgPSBwYXN0ZTAoY29sbmFtZXMocm93X2Nvb3JkKVsxXSAsICIgKCIgLCBzdWJzdHIoYXMuY2hhcmFjdGVyKHJlc19wY2EkZWlnWywyXVsxXSksIDEsIDUpICwgIiUpIiksDQogICAgICAgICAgICB5bGFiID0gcGFzdGUwKGNvbG5hbWVzKHJvd19jb29yZClbMl0gLCAiICgiICwgc3Vic3RyKGFzLmNoYXJhY3RlcihyZXNfcGNhJGVpZ1ssMl1bMl0pLCAxLCA1KSAsICIlKSIpLA0KICAgICAgICAgICAgemxhYiA9IHBhc3RlMChjb2xuYW1lcyhyb3dfY29vcmQpWzNdICwgIiAoIiAsIHN1YnN0cihhcy5jaGFyYWN0ZXIocmVzX3BjYSRlaWdbLDJdWzNdKSwgMSwgNSkgLCAiJSkiKSwNCiAgICAgICAgICAgIHhsaW0gPSBjKG1pbihyb3dfY29vcmRbLCAxXS0xMCksIG1heChyb3dfY29vcmRbLCAxXSkrMTApLCANCiAgICAgICAgICAgIHlsaW0gPSBjKG1pbihyb3dfY29vcmRbLCAyXS0xMCksIG1heChyb3dfY29vcmRbLCAyXSkrMTApLCANCiAgICAgICAgICAgIHpsaW0gPSBjKG1pbihyb3dfY29vcmRbLCAzXS0xMCksIG1heChyb3dfY29vcmRbLCAzXSkrMTApLA0KICAgICAgICAgICAgY29sID0gImRhcmtjeWFuIiwNCiAgICAgICAgICAgIHNpemU9MTAsDQogICAgICAgICAgICBtYWluID0gIlBDQSBBc3NlbWJsYWdlIikNCg0KcmdsOjp0ZXh0M2QoeD1yb3dfY29vcmRbYygiV05fVEZfMSIsICJXTl9URl8yIiwgIldOX1RGXzMiKSwgMV0rNSwgDQogICAgICAgICAgICB5PXJvd19jb29yZFtjKCJXTl9URl8xIiwgIldOX1RGXzIiLCAiV05fVEZfMyIpLCAyXSs1LCANCiAgICAgICAgICAgIHo9cm93X2Nvb3JkW2MoIldOX1RGXzEiLCAiV05fVEZfMiIsICJXTl9URl8zIiksIDNdKzUsDQogICAgICAgICAgICB0ZXh0PWMoIldOX1RGXzEiLCAiV05fVEZfMiIsICJXTl9URl8zIiksDQogICAgICAgICAgICBjb2wgPSAiZGFya2N5YW4iLA0KICAgICAgICAgICAgc2l6ZT0xMCkNCg0KDQpyZ2w6OnBvaW50czNkKHg9cm93X2Nvb3JkW2MoIlNEX1RGXzEiLCAiU0RfVEZfMiIsICJTRF9URl8zIiksIDFdLCANCiAgICAgICAgICAgICAgeT1yb3dfY29vcmRbYygiU0RfVEZfMSIsICJTRF9URl8yIiwgIlNEX1RGXzMiKSwgMl0sIA0KICAgICAgICAgICAgICB6PXJvd19jb29yZFtjKCJTRF9URl8xIiwgIlNEX1RGXzIiLCAiU0RfVEZfMyIpLCAzXSwNCiAgICAgICAgICAgICAgY29sID0gImRhcmtvcmFuZ2UiLA0KICAgICAgICAgICAgICBzaXplPTEwKQ0KDQpyZ2w6OnRleHQzZCh4PXJvd19jb29yZFtjKCJTRF9URl8xIiwgIlNEX1RGXzIiLCAiU0RfVEZfMyIpLCAxXSs1LCANCiAgICAgICAgICAgIHk9cm93X2Nvb3JkW2MoIlNEX1RGXzEiLCAiU0RfVEZfMiIsICJTRF9URl8zIiksIDJdKzUsIA0KICAgICAgICAgICAgej1yb3dfY29vcmRbYygiU0RfVEZfMSIsICJTRF9URl8yIiwgIlNEX1RGXzMiKSwgM10rNSwNCiAgICAgICAgICAgIHRleHQ9YygiU0RfVEZfMSIsICJTRF9URl8yIiwgIlNEX1RGXzMiKSwNCiAgICAgICAgICAgIGNvbCA9ICJkYXJrb3JhbmdlIiwNCiAgICAgICAgICAgIHNpemU9MTApDQoNCiMgQWN0aXZhdGUgZm9yIG9yIGdpZiBnZW5lcmF0aW9uDQpwYXIzZCh3aW5kb3dSZWN0ID0gYygyMCwgMzAsIDgwMCwgODAwKSkNCg0KIyByZ2w6Om1vdmllM2QoDQojICBtb3ZpZT0iM2RBbmltYXRlZF9QQ0FfbWV0YVRfYXNzZW1ibGFnZSIsDQojICBzcGluM2QoIGF4aXMgPSBjKDAsIDAsIDEpLCBycG0gPSAzKSwNCiMgIGR1cmF0aW9uID0gMjAsDQojICBkaXIgPSAiLi4vZmlndXJlcyIsDQojICB0eXBlID0gImdpZiIsDQojICBjbGVhbiA9IFRSVUUsIHdlYnNob3Q9RkFMU0UsIGZwcz0yMCkNCmBgYA0KDQojIyMgUENBIG1ldGFib2xvbWljcw0KDQpgYGB7ciBQQ0FfY2xyX2FsbF90aW1lcywgZmlnLmhlaWdodD03LCBmaWcud2lkdGg9N30NCnBhcihtZnJvdyA9IGMoMiwyKSkNCnJlc19wY2FfbWV0YUJfYWxsX3RpbWVzIDwtIFBDQSh0KG1ldGFib2xvbWljc19hbGxfdGltZXNfY2xyKSwgc2NhbGUudW5pdCA9IFRSVUUsIGdyYXBoID0gRkFMU0UpDQoNCmBgYA0KDQoNCmBgYHtyIFBFUk1BTk9WQV9hc3NlbWJsYWdlX21ldGFCX2FsbF90aW1lc30NCg0KbWV0YWJvbG9taWNzX2RhdGEgPC0gdChtZXRhYm9sb21pY3NfYWxsX3RpbWVzX2NscikNCm1ldGFib2xvbWljc190bXAgPC0gZGF0YS5mcmFtZShtZXRhZGF0YV90YWJsZV9tZXRhQl9hbGxfdGltZXMsIHN0cmluZ3NBc0ZhY3RvcnMgPSBUUlVFKQ0KcGVybWFub3ZhIDwtIGFkb25pczIoZm9ybXVsYSA9IGFzLmZvcm11bGEocGFzdGUwKCJtZXRhYm9sb21pY3NfZGF0YX4iLCAiY29uZGl0aW9uIikpLA0KICAgICAgICAgICAgICAgICAgICAgZGF0YSA9IG1ldGFib2xvbWljc190bXAsIG1ldGhvZD0iZXVjbGlkZWFuIiwgDQogICAgICAgICAgICAgICAgICAgICBwZXJtdXRhdGlvbnMgPSA5OTksIHNxcnQuZGlzdCA9IEZBTFNFLCBhZGQgPSBGQUxTRSwgYnkgPSAidGVybXMiKQ0KcHYgPSBwZXJtYW5vdmEkYFByKD5GKWBbMV0NCg0Kcm0obWV0YWJvbG9taWNzX2RhdGEsIG1ldGFib2xvbWljc190bXApDQpgYGANCg0KDQpgYGB7ciBQQ0FfMkRfcGxvdHNfYXNzZW1ibGFnZV9tZXRhYm9sb21pY3NfYmlzLCBmaWcuaGVpZ2h0PTQsIGZpZy53aWR0aD05fQ0KDQpwY2FfdG1wX21ldGFCIDwtIHJiaW5kKHJlc19wY2FfbWV0YUJfYWxsX3RpbWVzJGluZCRjb29yZCwNCiAgICAgICAgICAgICAgICAgcmVzX3BjYV9tZXRhQl9hbGxfdGltZXMkaW5kLnN1cCRjb29yZCkgJT4lIGRhdGEuZnJhbWUoKSAlPiUgDQogIHJvd25hbWVzX3RvX2NvbHVtbigic2FtcGxlIikNCg0KDQpwY2FfZGF0YV9tZXRhQiA8LSBsZWZ0X2pvaW4ocGNhX3RtcF9tZXRhQiwgcm93bmFtZXNfdG9fY29sdW1uKG1ldGFkYXRhX3RhYmxlX21ldGFCX2FsbF90aW1lcyksIGJ5PWpvaW5fYnkoInNhbXBsZSIgPT0gInJvd25hbWUiKSkNCnJtKHBjYV90bXBfbWV0YUIpDQoNCkQxX3RleHQgPSBwYXN0ZTAoIkRpbSAxICgiLCANCiAgICAgICAgICAgICAgICAgcm91bmQoZGF0YS5mcmFtZShyZXNfcGNhX21ldGFCX2FsbF90aW1lcyRlaWcpJHBlcmNlbnRhZ2Uub2YudmFyaWFuY2VbMV0sIGRpZ2l0cz0yKSwgDQogICAgICAgICAgICAgICAgICIlKSIpDQpEMl90ZXh0ID0gcGFzdGUwKCJEaW0gMiAoIiwgDQogICAgICAgICAgICAgICAgIHJvdW5kKGRhdGEuZnJhbWUocmVzX3BjYV9tZXRhQl9hbGxfdGltZXMkZWlnKSRwZXJjZW50YWdlLm9mLnZhcmlhbmNlWzJdLCBkaWdpdHM9MiksIA0KICAgICAgICAgICAgICAgICAiJSkiKQ0KRDNfdGV4dCA9IHBhc3RlMCgiRGltIDMgKCIsIA0KICAgICAgICAgICAgICAgICByb3VuZChkYXRhLmZyYW1lKHJlc19wY2FfbWV0YUJfYWxsX3RpbWVzJGVpZykkcGVyY2VudGFnZS5vZi52YXJpYW5jZVszXSwgZGlnaXRzPTIpLCANCiAgICAgICAgICAgICAgICAgIiUpIikNCg0KZ2dwbG90KHBjYV9kYXRhX21ldGFCKSArDQogIGdlb21fcG9pbnQoYWVzKHg9RGltLjEsIHk9RGltLjIsIGNvbG91cj1jb2xvciwgKSwgc2l6ZT0zKSArDQogIHNjYWxlX2NvbG91cl9pZGVudGl0eSgiQ29uZGl0aW9uIiwgbGFiZWxzPWMoIldpbnRlciBOaWdodCIsICJTdW1tZXIgRGF5IiksIGd1aWRlID0gImxlZ2VuZCIpICsNCiAgZ2dyZXBlbDo6Z2VvbV90ZXh0X3JlcGVsKGFlcyh4PURpbS4xLCB5PURpbS4yLCBjb2xvdXI9Y29sb3IsIGxhYmVsPXNhbXBsZSksIA0KICAgICAgICAgICAgICAgICAgICAgICAgICAgbnVkZ2VfeSA9IC0yLCAgc2VlZCA9IDQyLCBzZWdtZW50LnNpemU9MC4xLCBzaG93LmxlZ2VuZCA9IEZBTFNFKSArDQogIHhsYWIoRDFfdGV4dCkgKyANCiAgeWxhYihEMl90ZXh0KSArDQogIGdndGl0bGUoIlBDQSBtZXRhLW1ldGFib2xvbWljcyIpICsNCiAgbGFicyhjYXB0aW9uID0gIHBhc3RlMCgiUEVSTUFOT1ZBIG9uIGNvbmRpdGlvbjogcC12YWx1ZTogIiwgcHYpKSArIA0KICB0aGVtZShwYW5lbC5iYWNrZ3JvdW5kPSBlbGVtZW50X3JlY3QoZmlsbD0iI2Y0ZjRmNCIpLA0KICAgICAgICBwbG90LmNhcHRpb24gPSBlbGVtZW50X3RleHQoc2l6ZT03KSkgLT4gcDFfbWV0YUINCiANCg0KZ2dwbG90KHBjYV9kYXRhX21ldGFCKSArDQogIGdlb21fcG9pbnQoYWVzKHg9RGltLjEsIHk9RGltLjMsIGNvbG91cj1jb2xvciksIHNpemU9MykgKw0KICBzY2FsZV9jb2xvdXJfaWRlbnRpdHkoIkNvbmRpdGlvbiIsIGxhYmVscz1jKCJXaW50ZXIgTmlnaHQiLCAiU3VtbWVyIERheSIpLCBndWlkZSA9ICJsZWdlbmQiKSArDQogIGdncmVwZWw6Omdlb21fdGV4dF9yZXBlbChhZXMoeD1EaW0uMSwgeT1EaW0uMywgY29sb3VyPWNvbG9yLCBsYWJlbD1zYW1wbGUpLCANCiAgICAgICAgICAgICAgICAgICAgICAgICAgIG51ZGdlX3kgPSAtMiwgIHNlZWQgPSA0Miwgc2VnbWVudC5zaXplPTAuMSwgc2hvdy5sZWdlbmQgPSBGQUxTRSkgKw0KICB4bGFiKEQxX3RleHQpICsgDQogIHlsYWIoRDNfdGV4dCkgKw0KICBnZ3RpdGxlKCJQQ0EgbWV0YS1tZXRhYm9sb21pY3MiKSArDQogIGxhYnMoY2FwdGlvbiA9ICBwYXN0ZTAoIlBFUk1BTk9WQSBvbiBjb25kaXRpb246IHAtdmFsdWU6ICIsIHB2KSkgKyANCiAgdGhlbWUocGFuZWwuYmFja2dyb3VuZD0gZWxlbWVudF9yZWN0KGZpbGw9IiNmNGY0ZjQiKSwNCiAgICAgICAgcGxvdC5jYXB0aW9uID0gZWxlbWVudF90ZXh0KHNpemU9NykpIC0+IHAyX21ldGFCDQoNCmdncGxvdChwY2FfZGF0YV9tZXRhQikgKw0KICBnZW9tX3BvaW50KGFlcyh4PURpbS4yLCB5PURpbS4zLCBjb2xvdXI9Y29sb3IpLCBzaXplPTMpICsNCiAgc2NhbGVfY29sb3VyX2lkZW50aXR5KCJDb25kaXRpb24iLCBsYWJlbHM9YygiV2ludGVyIE5pZ2h0IiwgIlN1bW1lciBEYXkiKSwgZ3VpZGUgPSAibGVnZW5kIikgKw0KICBnZ3JlcGVsOjpnZW9tX3RleHRfcmVwZWwoYWVzKHg9RGltLjIsIHk9RGltLjMsIGNvbG91cj1jb2xvciwgbGFiZWw9c2FtcGxlKSwgDQogICAgICAgICAgICAgICAgICAgICAgICAgICBudWRnZV95ID0gLTIsICBzZWVkID0gNDIsIHNlZ21lbnQuc2l6ZT0wLjEsIHNob3cubGVnZW5kID0gRkFMU0UpICsNCiAgeGxhYihEMl90ZXh0KSArIA0KICB5bGFiKEQzX3RleHQpICsNCiAgZ2d0aXRsZSgiUENBIG1ldGEtbWV0YWJvbG9taWNzIikgKw0KICBsYWJzKGNhcHRpb24gPSAgcGFzdGUwKCJQRVJNQU5PVkEgb24gY29uZGl0aW9uOiBwLXZhbHVlOiAiLCBwdikpICsgDQogIHRoZW1lKHBhbmVsLmJhY2tncm91bmQ9IGVsZW1lbnRfcmVjdChmaWxsPSIjZjRmNGY0IiksDQogICAgICAgIHBsb3QuY2FwdGlvbiA9IGVsZW1lbnRfdGV4dChzaXplPTcpKSAtPiBwM19tZXRhQg0KDQpwbG90Z3JpZF9QQ0FfbWV0YUIgPC0gZ2dhcnJhbmdlKHAxX21ldGFCLCBwMl9tZXRhQiwgcDNfbWV0YUIsIGxhYmVscyA9IGMoIkEiLCAiQiIsICJDIiksIGNvbW1vbi5sZWdlbmQ9VFJVRSwgbGVnZW5kPSJib3R0b20iLCBucm93PTEpIA0KDQpwcmludChwbG90Z3JpZF9QQ0FfbWV0YUIpDQoNCmdnc2F2ZShwbG90ID0gcDFfbWV0YUIsIGZpbGVuYW1lID0gIi4uL2ZpZ3VyZXMvUENBX21ldGFib2xvbWljc19hbGxfdGltZXMudGlmZiIsIGRwaSA9IDMwMCwgd2lkdGggPSA1LCBoZWlnaHQgPSA1LCBiZz0id2hpdGUiKQ0KDQpnZ3NhdmUocGxvdCA9IHBsb3RncmlkX1BDQV9tZXRhQiwgZmlsZW5hbWUgPSAiLi4vZmlndXJlcy9QQ0FfbWV0YWJvbG9taWNzXzNfY29tcG9zYW50ZXNfYWxsX3RpbWVzLnRpZmYiLCBkcGkgPSAzMDAsIHdpZHRoID0gMTUsIGhlaWdodCA9IDUsIGJnPSJ3aGl0ZSIpDQoNCmdnc2F2ZShwbG90PXAxX21ldGFCLCBmaWxlbmFtZSA9ICIuLi9maWd1cmVzL1BDQV9tZXRhYm9sb21pY3NfYWxsX3RpbWVzLnRpZmYiLCBkcGkgPSAzMDAsIHdpZHRoID0gNiwgaGVpZ2h0ID0gNSwgYmc9IndoaXRlIikNCmBgYA0KDQpgYGB7ciAzRF9QQ0FfbWV0YUJfY2xyX2FsbF90aW1lcG9pbnRzLCB3ZWJnbD1UUlVFLCBmaWcuaGVpZ2h0PTcsIGZpZy53aWR0aD03LCBtZXNzYWdlPUZBTFNFfQ0KDQpyb3dfY29vcmQgPC0gcmVzX3BjYV9tZXRhQl9hbGxfdGltZXMkaW5kJGNvb3JkDQoNCmludmlzaWJsZShyZ2w6Om9wZW4zZCgpKQ0KDQojcmdsOjpiZzNkKCJsaWdodGdyYXkiKQ0KcmdsOjpwbG90M2QoeD1yb3dfY29vcmRbYygiV05fVEZfMSIsICJXTl9URl8yIiwgIldOX1RGXzMiKSwgMV0sIA0KICAgICAgICAgICAgeT1yb3dfY29vcmRbYygiV05fVEZfMSIsICJXTl9URl8yIiwgIldOX1RGXzMiKSwgMl0sIA0KICAgICAgICAgICAgej1yb3dfY29vcmRbYygiV05fVEZfMSIsICJXTl9URl8yIiwgIldOX1RGXzMiKSwgM10sDQogICAgICAgICAgICB4bGFiID0gcGFzdGUwKGNvbG5hbWVzKHJvd19jb29yZClbMV0gLCAiICgiICwgc3Vic3RyKGFzLmNoYXJhY3RlcihyZXNfcGNhJGVpZ1ssMl1bMV0pLCAxLCA1KSAsICIlKSIpLA0KICAgICAgICAgICAgeWxhYiA9IHBhc3RlMChjb2xuYW1lcyhyb3dfY29vcmQpWzJdICwgIiAoIiAsIHN1YnN0cihhcy5jaGFyYWN0ZXIocmVzX3BjYSRlaWdbLDJdWzJdKSwgMSwgNSkgLCAiJSkiKSwNCiAgICAgICAgICAgIHpsYWIgPSBwYXN0ZTAoY29sbmFtZXMocm93X2Nvb3JkKVszXSAsICIgKCIgLCBzdWJzdHIoYXMuY2hhcmFjdGVyKHJlc19wY2EkZWlnWywyXVszXSksIDEsIDUpICwgIiUpIiksDQogICAgICAgICAgICB4bGltID0gYyhtaW4ocm93X2Nvb3JkWywgMV0tMTApLCBtYXgocm93X2Nvb3JkWywgMV0pKzEwKSwgDQogICAgICAgICAgICB5bGltID0gYyhtaW4ocm93X2Nvb3JkWywgMl0tMTApLCBtYXgocm93X2Nvb3JkWywgMl0pKzEwKSwgDQogICAgICAgICAgICB6bGltID0gYyhtaW4ocm93X2Nvb3JkWywgM10tMTApLCBtYXgocm93X2Nvb3JkWywgM10pKzEwKSwNCiAgICAgICAgICAgIGNvbCA9ICJkYXJrY3lhbiIsDQogICAgICAgICAgICBzaXplPTEwLA0KICAgICAgICAgICAgbWFpbiA9ICJQQ0EgbWV0YS1tZXRhYm9sb21pY3MgYXNzZW1ibGFnZSIpDQoNCnJnbDo6dGV4dDNkKHg9cm93X2Nvb3JkW2MoIldOX1RGXzEiLCAiV05fVEZfMiIsICJXTl9URl8zIiksIDFdKzUsIA0KICAgICAgICAgICAgeT1yb3dfY29vcmRbYygiV05fVEZfMSIsICJXTl9URl8yIiwgIldOX1RGXzMiKSwgMl0rNSwgDQogICAgICAgICAgICB6PXJvd19jb29yZFtjKCJXTl9URl8xIiwgIldOX1RGXzIiLCAiV05fVEZfMyIpLCAzXSs1LA0KICAgICAgICAgICAgdGV4dD1jKCJXTl9URl8xIiwgIldOX1RGXzIiLCAiV05fVEZfMyIpLA0KICAgICAgICAgICAgY29sID0gImRhcmtjeWFuIiwNCiAgICAgICAgICAgIHNpemU9MTApDQoNCg0KcmdsOjpwb2ludHMzZCh4PXJvd19jb29yZFtjKCJTRF9URl8xIiwgIlNEX1RGXzIiLCAiU0RfVEZfMyIpLCAxXSwgDQogICAgICAgICAgICAgIHk9cm93X2Nvb3JkW2MoIlNEX1RGXzEiLCAiU0RfVEZfMiIsICJTRF9URl8zIiksIDJdLCANCiAgICAgICAgICAgICAgej1yb3dfY29vcmRbYygiU0RfVEZfMSIsICJTRF9URl8yIiwgIlNEX1RGXzMiKSwgM10sDQogICAgICAgICAgICAgIGNvbCA9ICJkYXJrb3JhbmdlIiwNCiAgICAgICAgICAgICAgc2l6ZT0xMCkNCg0KcmdsOjp0ZXh0M2QoeD1yb3dfY29vcmRbYygiU0RfVEZfMSIsICJTRF9URl8yIiwgIlNEX1RGXzMiKSwgMV0rNSwgDQogICAgICAgICAgICB5PXJvd19jb29yZFtjKCJTRF9URl8xIiwgIlNEX1RGXzIiLCAiU0RfVEZfMyIpLCAyXSs1LCANCiAgICAgICAgICAgIHo9cm93X2Nvb3JkW2MoIlNEX1RGXzEiLCAiU0RfVEZfMiIsICJTRF9URl8zIiksIDNdKzUsDQogICAgICAgICAgICB0ZXh0PWMoIlNEX1RGXzEiLCAiU0RfVEZfMiIsICJTRF9URl8zIiksDQogICAgICAgICAgICBjb2wgPSAiZGFya29yYW5nZSIsDQogICAgICAgICAgICBzaXplPTEwKQ0KDQoNCnJnbDo6cG9pbnRzM2QoeD1yb3dfY29vcmRbYygiV05fVDBfMSIsICJXTl9UMF8yIiwgIldOX1QwXzMiKSwgMV0sIA0KICAgICAgICAgICAgICB5PXJvd19jb29yZFtjKCJXTl9UMF8xIiwgIldOX1QwXzIiLCAiV05fVDBfMyIpLCAyXSwgDQogICAgICAgICAgICAgIHo9cm93X2Nvb3JkW2MoIldOX1QwXzEiLCAiV05fVDBfMiIsICJXTl9UMF8zIiksIDNdLA0KICAgICAgICAgICAgICBjb2wgPSAiZGFya2N5YW4iLA0KICAgICAgICAgICAgICBzaXplPTEwKQ0KDQpyZ2w6OnRleHQzZCh4PXJvd19jb29yZFtjKCJXTl9UMF8xIiwgIldOX1QwXzIiLCAiV05fVDBfMyIpLCAxXSs1LCANCiAgICAgICAgICAgIHk9cm93X2Nvb3JkW2MoIldOX1QwXzEiLCAiV05fVDBfMiIsICJXTl9UMF8zIiksIDJdKzUsIA0KICAgICAgICAgICAgej1yb3dfY29vcmRbYygiV05fVDBfMSIsICJXTl9UMF8yIiwgIldOX1QwXzMiKSwgM10rNSwNCiAgICAgICAgICAgIHRleHQ9YygiV05fVDBfMSIsICJXTl9UMF8yIiwgIldOX1QwXzMiKSwNCiAgICAgICAgICAgIGNvbCA9ICJkYXJrY3lhbiIsDQogICAgICAgICAgICBzaXplPTEwKQ0KDQoNCnJnbDo6cG9pbnRzM2QoeD1yb3dfY29vcmRbYygiU0RfVDBfMSIsICJTRF9UMF8yIiwgIlNEX1QwXzMiKSwgMV0sIA0KICAgICAgICAgICAgICB5PXJvd19jb29yZFtjKCJTRF9UMF8xIiwgIlNEX1QwXzIiLCAiU0RfVDBfMyIpLCAyXSwgDQogICAgICAgICAgICAgIHo9cm93X2Nvb3JkW2MoIlNEX1QwXzEiLCAiU0RfVDBfMiIsICJTRF9UMF8zIiksIDNdLA0KICAgICAgICAgICAgICBjb2wgPSAiZGFya29yYW5nZSIsDQogICAgICAgICAgICAgIHNpemU9MTApDQoNCnJnbDo6dGV4dDNkKHg9cm93X2Nvb3JkW2MoIlNEX1QwXzEiLCAiU0RfVDBfMiIsICJTRF9UMF8zIiksIDFdKzUsIA0KICAgICAgICAgICAgeT1yb3dfY29vcmRbYygiU0RfVDBfMSIsICJTRF9UMF8yIiwgIlNEX1QwXzMiKSwgMl0rNSwgDQogICAgICAgICAgICB6PXJvd19jb29yZFtjKCJTRF9UMF8xIiwgIlNEX1QwXzIiLCAiU0RfVDBfMyIpLCAzXSs1LA0KICAgICAgICAgICAgdGV4dD1jKCJTRF9UMF8xIiwgIlNEX1QwXzIiLCAiU0RfVDBfMyIpLA0KICAgICAgICAgICAgY29sID0gImRhcmtvcmFuZ2UiLA0KICAgICAgICAgICAgc2l6ZT0xMCkNCg0KDQoNCiMgQWN0aXZhdGUgZm9yIG9yIGdpZiBnZW5lcmF0aW9uDQojIHBhcjNkKHdpbmRvd1JlY3QgPSBjKDIwLCAzMCwgODAwLCA4MDApKQ0KIyANCiMgcmdsOjptb3ZpZTNkKA0KIyAgbW92aWU9IjNkQW5pbWF0ZWRfUENBX21ldGFCX2Fzc2VtYmxhZ2UiLA0KIyAgc3BpbjNkKCBheGlzID0gYygwLCAwLCAxKSwgcnBtID0gMyksDQojICBkdXJhdGlvbiA9IDIwLA0KIyAgZGlyID0gIi4uL2ZpZ3VyZXMiLA0KIyAgdHlwZSA9ICJnaWYiLA0KIyAgY2xlYW4gPSBUUlVFLCB3ZWJzaG90PUZBTFNFLCBmcHM9MjApDQpgYGANCg0KYGBge3IgcGxvdF9GaWd1cmUyfQ0KDQpwbG90X2FsbCA8LSBnZ2FycmFuZ2UocDFfbWV0YUIsIHAxX21ldGFULCANCiAgICAgICAgICAgICAgICAgICAgICBsYWJlbHMgPSBjKCJBIiwgIkIiKSwgDQogICAgICAgICAgICAgICAgICAgICAgY29tbW9uLmxlZ2VuZD1UUlVFLA0KICAgICAgICAgICAgICAgICAgICAgIGxlZ2VuZD0iYm90dG9tIiwNCiAgICAgICAgICAgICAgICAgICAgICBucm93PTEsDQogICAgICAgICAgICAgICAgICAgICAgd2lkdGhzID0gYygxLCAxKSkNCg0KZ2dzYXZlKHBsb3QgPSBwbG90X2FsbCwgZmlsZW5hbWUgPSAiLi4vZmlndXJlcy9GaWd1cmVfMi50aWZmIiwgZHBpID0gMzAwLCB3aWR0aCA9IDExLCBoZWlnaHQgPSA2LCBiZz0id2hpdGUiKQ0KDQpgYGANCg0KIyMgSW5kaXZpZHVhbCBleHBsb3JhdG9yeSBhbmFseXNpcyBvZiBtZXRhdHJhbnNjcmlwdG9taWNzIGRhdGEgYnkgc3BlY2llcw0KDQojIyMjIERhdGEgcHJlcGFyYXRpb24NCg0KIyMjIyBMb2FkIGRhdGEgYnkgc3BlY2llcw0KDQpgYGB7ciByZWNvdmVyX3NwZWNpZXNfZGF0YX0NCg0KZGlvaHVfZ2VuZWlkIDwtIGFubm90YXRpb25fdGFibGUkT3JnYW5pc20gPT0gIkQuaHVuZ2FyaWNhIg0KZGlvaHVfZGYgPC0gY291bnRzX3N0YW5kYXJkW3N0YXJ0c1dpdGgocm93bmFtZXNfdG9fY29sdW1uKGNvdW50c19zdGFuZGFyZCkkcm93bmFtZSwgIkQuaHVuZ2FyaWNhIiksXQ0KDQpwc2VzeV9nZW5laWQgPC0gYW5ub3RhdGlvbl90YWJsZSRPcmdhbmlzbSA9PSAiUC5zeXJpbmdhZSINCnBzZXN5X2RmIDwtIGNvdW50c19zdGFuZGFyZFtzdGFydHNXaXRoKHJvd25hbWVzX3RvX2NvbHVtbihjb3VudHNfc3RhbmRhcmQpJHJvd25hbWUsICJQLnN5cmluZ2FlIiksXQ0KDQpwc2Vncl9nZW5laWQgPC0gYW5ub3RhdGlvbl90YWJsZSRPcmdhbmlzbSA9PSAiUC5ncmFtaW5pcyINCnBzZWdyX2RmIDwtIGNvdW50c19zdGFuZGFyZFtzdGFydHNXaXRoKHJvd25hbWVzX3RvX2NvbHVtbihjb3VudHNfc3RhbmRhcmQpJHJvd25hbWUsICJQLmdyYW1pbmlzIiksXQ0KDQpyaG9lbl9nZW5laWQgPC0gYW5ub3RhdGlvbl90YWJsZSRPcmdhbmlzbSA9PSAiUi5lbmNsZW5zaXMiDQpyaG9lbl9kZiA8LSBjb3VudHNfc3RhbmRhcmRbc3RhcnRzV2l0aChyb3duYW1lc190b19jb2x1bW4oY291bnRzX3N0YW5kYXJkKSRyb3duYW1lLCAiUi5lbmNsZW5zaXMiKSxdDQoNCmBgYA0KDQojIyMjIFplcm9lcyBzbW9vdGhpbmcgYnkgc3BlY2llcw0KDQpgYGB7ciB6ZXJvZXNfc21vb3RoaW5nX3NwZV9kYXRhfQ0KDQojIyByZW1haW5pbmcgemVyb2VzIHNtb290aGluZyAoYXMgaW4gWmhhbmcgZXQgYWwuIDIwMjEpDQptaW5fdmFsIDwtIGFwcGx5KGRpb2h1X2RmLCAxLCBmdW5jdGlvbih4KSAobWluKHhbeD4wXSkvMikpDQpmb3IgKGdlbmUgaW4gcm93Lm5hbWVzKGRpb2h1X2RmKSkgew0KICBkaW9odV9kZltnZW5lLF1bZGlvaHVfZGZbZ2VuZSxdID09IDBdIDwtIG1pbl92YWxbZ2VuZV0NCn0NCg0KbWluX3ZhbCA8LSBhcHBseShwc2VzeV9kZiwgMSwgZnVuY3Rpb24oeCkgKG1pbih4W3g+MF0pLzIpKQ0KZm9yIChnZW5lIGluIHJvdy5uYW1lcyhwc2VzeV9kZikpIHsNCiAgcHNlc3lfZGZbZ2VuZSxdW3BzZXN5X2RmW2dlbmUsXSA9PSAwXSA8LSBtaW5fdmFsW2dlbmVdDQp9DQoNCm1pbl92YWwgPC0gYXBwbHkocHNlZ3JfZGYsIDEsIGZ1bmN0aW9uKHgpIChtaW4oeFt4PjBdKS8yKSkNCmZvciAoZ2VuZSBpbiByb3cubmFtZXMocHNlZ3JfZGYpKSB7DQogIHBzZWdyX2RmW2dlbmUsXVtwc2Vncl9kZltnZW5lLF0gPT0gMF0gPC0gbWluX3ZhbFtnZW5lXQ0KfQ0KDQptaW5fdmFsIDwtIGFwcGx5KHJob2VuX2RmLCAxLCBmdW5jdGlvbih4KSAobWluKHhbeD4wXSkvMikpDQpmb3IgKGdlbmUgaW4gcm93Lm5hbWVzKHJob2VuX2RmKSkgew0KICByaG9lbl9kZltnZW5lLF1bcmhvZW5fZGZbZ2VuZSxdID09IDBdIDwtIG1pbl92YWxbZ2VuZV0NCn0NCmBgYA0KDQojIyMjIENMUiBUcmFuc2Zvcm1hdGlvbiBvZiBjb3VudHMgYnkgc3BlY2llcyANCg0KYGBge3IgY2xyX3RyYW5zZm9ybWF0aW9uX29mX3NwZV9kYXRhfQ0KZGlvaHVfY2xyIDwtIGRpb2h1X2RmDQpwc2VzeV9jbHIgPC0gcHNlc3lfZGYNCnBzZWdyX2NsciA8LSBwc2Vncl9kZg0KcmhvZW5fY2xyIDwtIHJob2VuX2RmDQoNCmZvciAoc2FtcCBpbiBzYW1wbGVzKSB7DQogICAgZGlvaHVfY2xyWyxzYW1wXSA8LSBhcy52ZWN0b3IoY2xyKGRpb2h1X2RmWyxzYW1wXSkpDQogICAgcHNlc3lfY2xyWyxzYW1wXSA8LSBhcy52ZWN0b3IoY2xyKHBzZXN5X2RmWyxzYW1wXSkpDQogICAgcHNlZ3JfY2xyWyxzYW1wXSA8LSBhcy52ZWN0b3IoY2xyKHBzZWdyX2RmWyxzYW1wXSkpDQogICAgcmhvZW5fY2xyWyxzYW1wXSA8LSBhcy52ZWN0b3IoY2xyKHJob2VuX2RmWyxzYW1wXSkpDQp9DQoNCmBgYA0KDQoNCiMjIyBUcmFuc2NyaXB0b21pY3MgY291bnRzIGhpc3RvZ3JhbXMgYnkgc3BlY2llcyB7LnRhYnNldH0NCg0KIyMjIyAqRGlvc3plZ2lhIGh1bmdhcmljYSoNCg0KYGBge3IgaGlzdF9jbHJfZGlvaHUsIGZpZy5oZWlnaHQ9NywgZmlnLndpZHRoPTd9DQpwYXIobWZyb3c9YygxLDEpKQ0KaGlzdCh1bmxpc3QoZGlvaHVfY2xyKSwNCiAgICAgYnJlYWtzID0gMjAwLA0KICAgICBjZXguYXhpcyA9IDAuNywNCiAgICAgbGFzID0gMSwNCiAgICAgY29sID0gInNreWJsdWUiLA0KICAgICB5bGltID0gYygwLDE4MDApLA0KICAgICB4bGltID0gYygtNSwxNSksDQogICAgIHhsYWIgPSAiY2xyLXRyYW5zZm9ybWVkIGNvdW50cyIsDQogICAgIG1haW4gPSAiRGlzdHJpYnV0aW9uIG9mIEQuIGh1bmdhcmljYSBjbHItdHJhbnNmb3JtZWQgY291bnRzIikNCmBgYA0KDQojIyMjICpQc2V1ZG9tb25hcyBncmFtaW5pcyoNCg0KYGBge3IgaGlzdF9jbHJfcHNlZ3IsIGZpZy5oZWlnaHQ9NywgZmlnLndpZHRoPTd9DQpwYXIobWZyb3c9YygxLDEpKQ0KaGlzdCh1bmxpc3QocHNlZ3JfY2xyKSwNCiAgICAgYnJlYWtzID0gMjAwLA0KICAgICBjZXguYXhpcyA9IDAuNywNCiAgICAgbGFzID0gMSwNCiAgICAgY29sID0gInNreWJsdWUiLA0KICAgICB5bGltID0gYygwLDE4MDApLA0KICAgICB4bGltID0gYygtNSwxNSksDQogICAgIHhsYWIgPSAiY2xyLXRyYW5zZm9ybWVkIGNvdW50cyIsDQogICAgIG1haW4gPSAiRGlzdHJpYnV0aW9uIG9mIFAuIGdyYW1pbmlzIGNsci10cmFuc2Zvcm1lZCBjb3VudHMiKQ0KYGBgDQoNClZlcnkgZmV3ICpQLiBncmFtaW5pcyogY291bnRzLCBwcm9iYWJseSBub3QgZXhwbG9pdGFibGUuDQoNCiMjIyMgKlBzZXVkb21vbmFzIHN5cmluZ2FlKg0KDQpgYGB7ciBoaXN0X2Nscl9wc2VzeSwgZmlnLmhlaWdodD03LCBmaWcud2lkdGg9N30NCnBhcihtZnJvdz1jKDEsMSkpDQpoaXN0KHVubGlzdChwc2VzeV9jbHIpLA0KICAgICBicmVha3MgPSAyMDAsDQogICAgIGNleC5heGlzID0gMC43LA0KICAgICBsYXMgPSAxLA0KICAgICBjb2wgPSAic2t5Ymx1ZSIsDQogICAgIHlsaW0gPSBjKDAsMTgwMCksDQogICAgIHhsaW0gPSBjKC01LDE1KSwNCiAgICAgeGxhYiA9ICJjbHItdHJhbnNmb3JtZWQgY291bnRzIiwNCiAgICAgbWFpbiA9ICJEaXN0cmlidXRpb24gb2YgUC4gc3lyaW5nYWUgY2xyLXRyYW5zZm9ybWVkIGNvdW50cyIpDQpgYGANCg0KIyMjIyAqUmhvZG9jb2NjdXMgZW5jbGVuc2lzKg0KDQpgYGB7ciBoaXN0X2Nscl9yaG9lbiwgZmlnLmhlaWdodD03LCBmaWcud2lkdGg9N30NCnBhcihtZnJvdz1jKDEsMSkpDQpoaXN0KHVubGlzdChyaG9lbl9jbHIpLA0KICAgICBicmVha3MgPSAyMDAsDQogICAgIGNleC5heGlzID0gMC43LA0KICAgICBsYXMgPSAxLA0KICAgICBjb2wgPSAic2t5Ymx1ZSIsDQogICAgIHlsaW0gPSBjKDAsMTgwMCksDQogICAgIHhsaW0gPSBjKC01LDE1KSwNCiAgICAgeGxhYiA9ICJjbHItdHJhbnNmb3JtZWQgY291bnRzIiwNCiAgICAgbWFpbiA9ICJEaXN0cmlidXRpb24gb2YgUi4gZW5jbGVuc2lzIGNsci10cmFuc2Zvcm1lZCBjb3VudHMiKQ0KYGBgDQoNCiMjIyBUcmFuc2NyaXB0b21pY3MgY291bnRzIGJveC1wbG90cyBieSBzcGVjaWVzIHsudGFic2V0fQ0KDQojIyMjICpEaW9zemVnaWEgaHVuZ2FyaWNhKg0KDQpgYGB7ciBib3hfcGxvdF9jbHJfZGlvaHUsIGZpZy5oZWlnaHQ9NywgZmlnLndpZHRoPTd9DQpib3hwbG90KGRpb2h1X2NsciwNCiAgICAgICAgbWFpbiA9ICJELiBodW5nYXJpY2EgY2xyLXRyYW5zZm9ybWVkIGV4cHJlc3Npb24iLA0KICAgICAgICBob3Jpem9udGFsID0gVFJVRSwNCiAgICAgICAgY29sID0gbWV0YWRhdGFfdGFibGUkY29sb3IsDQogICAgICAgIGNleCA9IDAuNSwNCiAgICAgICAgY2V4LmF4aXMgPSAwLjgsDQogICAgICAgIGxhcyA9IDEpDQoNCmBgYA0KDQojIyMjICpQc2V1ZG9tb25hcyBncmFtaW5pcyoNCg0KYGBge3IgYm94X3Bsb3RfY2xyX3BzZWdyLCBmaWcuaGVpZ2h0PTcsIGZpZy53aWR0aD03fQ0KYm94cGxvdChwc2Vncl9jbHIsDQogICAgICAgIG1haW4gPSAiUC4gZ3JhbWluaXMgY2xyLXRyYW5zZm9ybWVkIGV4cHJlc3Npb24iLA0KICAgICAgICBob3Jpem9udGFsID0gVFJVRSwNCiAgICAgICAgY29sID0gbWV0YWRhdGFfdGFibGUkY29sb3IsDQogICAgICAgIGNleCA9IDAuNSwNCiAgICAgICAgY2V4LmF4aXMgPSAwLjgsDQogICAgICAgIGxhcyA9IDEpDQoNCmBgYA0KDQojIyMjICpQc2V1ZG9tb25hcyBzeXJpbmdhZSoNCg0KYGBge3IgYm94X3Bsb3RfY2xyX3BzZXN5LCBmaWcuaGVpZ2h0PTcsIGZpZy53aWR0aD03fQ0KYm94cGxvdChwc2VzeV9jbHIsDQogICAgICAgIG1haW4gPSAiUC4gc3lyaW5nYWUgY2xyLXRyYW5zZm9ybWVkIGV4cHJlc3Npb24iLA0KICAgICAgICBob3Jpem9udGFsID0gVFJVRSwNCiAgICAgICAgY29sID0gbWV0YWRhdGFfdGFibGUkY29sb3IsDQogICAgICAgIGNleCA9IDAuNSwNCiAgICAgICAgY2V4LmF4aXMgPSAwLjgsDQogICAgICAgIGxhcyA9IDEpDQoNCmBgYA0KDQojIyMjICpSaG9kb2NvY2N1cyBlbmNsZW5zaXMqDQoNCmBgYHtyIGJveF9wbG90X2Nscl9yaG9lbiwgZmlnLmhlaWdodD03LCBmaWcud2lkdGg9N30NCmJveHBsb3QocmhvZW5fY2xyLA0KICAgICAgICBtYWluID0gIlIuIGVuY2xlbnNpcyBjbHItdHJhbnNmb3JtZWQgZXhwcmVzc2lvbiIsDQogICAgICAgIGhvcml6b250YWwgPSBUUlVFLA0KICAgICAgICBjb2wgPSBtZXRhZGF0YV90YWJsZSRjb2xvciwNCiAgICAgICAgY2V4ID0gMC41LA0KICAgICAgICBjZXguYXhpcyA9IDAuOCwNCiAgICAgICAgbGFzID0gMSkNCg0KYGBgDQoNCiMjIyBUcmFuc2NyaXB0b21pY3MgY291bnRzIFBDQSBieSBzcGVjaWVzIHsudGFic2V0fQ0KDQojIyMjICpEaW9zemVnaWEgaHVuZ2FyaWNhKg0KDQpgYGB7ciBQQ0FfY2xyX2Rpb2h1fQ0KDQpyZXNfcGNhIDwtIFBDQSh0KGRpb2h1X2NsciksIHNjYWxlLnVuaXQgPSBUUlVFLCBncmFwaCA9IEZBTFNFKQ0KDQpgYGANCg0KYGBge3IgUEVSTUFOT1ZBX2Rpb2h1fQ0KY291bnRzX2RhdGEgPC0gdChkaW9odV9jbHIpDQptZXRhZGF0YV90bXAgPC0gZGF0YS5mcmFtZShtZXRhZGF0YV90YWJsZSwgc3RyaW5nc0FzRmFjdG9ycyA9IFRSVUUpDQpwZXJtYW5vdmEgPC0gYWRvbmlzMihmb3JtdWxhID0gYXMuZm9ybXVsYShwYXN0ZTAoImNvdW50c19kYXRhfiIsICJjb25kaXRpb24iKSksIA0KICAgICAgICAgICAgICAgICAgICAgZGF0YSA9IG1ldGFkYXRhX3RtcCwgbWV0aG9kPSJldWNsaWRlYW4iLCAjIG1ldGhvZD0iYnJheSIsICNvdHVfZGF0YX5USU1FDQogICAgICAgICAgICAgICAgICAgICBwZXJtdXRhdGlvbnMgPSA5OTksIHNxcnQuZGlzdCA9IEZBTFNFLCBhZGQgPSBGQUxTRSwgYnkgPSAidGVybXMiKQ0KcHYgPSBwZXJtYW5vdmEkYFByKD5GKWBbMV0NCg0Kcm0oY291bnRzX2RhdGEsIG1ldGFkYXRhX3RtcCkNCmBgYA0KDQpgYGB7ciBQQ0FfMkRfcGxvdHNfZGlvaHUsIGZpZy5oZWlnaHQ9NCwgZmlnLndpZHRoPTl9DQoNCnBjYV90bXAgPC0gcmJpbmQocmVzX3BjYSRpbmQkY29vcmQsDQogICAgICAgICAgICAgICAgIHJlc19wY2EkaW5kLnN1cCRjb29yZCkgJT4lIGRhdGEuZnJhbWUoKSAlPiUgDQogIHJvd25hbWVzX3RvX2NvbHVtbigic2FtcGxlIikNCg0KDQpwY2FfZGF0YSA8LSBsZWZ0X2pvaW4ocGNhX3RtcCwgcm93bmFtZXNfdG9fY29sdW1uKG1ldGFkYXRhX3RhYmxlKSwgYnk9am9pbl9ieSgic2FtcGxlIiA9PSAicm93bmFtZSIpKQ0Kcm0ocGNhX3RtcCkNCg0KRDFfdGV4dCA9IHBhc3RlMCgiRGltIDEgKCIsIA0KICAgICAgICAgICAgICAgICByb3VuZChkYXRhLmZyYW1lKHJlc19wY2EkZWlnKSRwZXJjZW50YWdlLm9mLnZhcmlhbmNlWzFdLCBkaWdpdHM9MiksIA0KICAgICAgICAgICAgICAgICAiJSkiKQ0KRDJfdGV4dCA9IHBhc3RlMCgiRGltIDIgKCIsIA0KICAgICAgICAgICAgICAgICByb3VuZChkYXRhLmZyYW1lKHJlc19wY2EkZWlnKSRwZXJjZW50YWdlLm9mLnZhcmlhbmNlWzJdLCBkaWdpdHM9MiksIA0KICAgICAgICAgICAgICAgICAiJSkiKQ0KRDNfdGV4dCA9IHBhc3RlMCgiRGltIDMgKCIsIA0KICAgICAgICAgICAgICAgICByb3VuZChkYXRhLmZyYW1lKHJlc19wY2EkZWlnKSRwZXJjZW50YWdlLm9mLnZhcmlhbmNlWzNdLCBkaWdpdHM9MiksIA0KICAgICAgICAgICAgICAgICAiJSkiKQ0KDQpnZ3Bsb3QocGNhX2RhdGEpICsNCiAgZ2VvbV9wb2ludChhZXMoeD1EaW0uMSwgeT1EaW0uMiwgY29sb3VyPWNvbG9yLCApLCBzaXplPTMpICsNCiAgc2NhbGVfY29sb3VyX2lkZW50aXR5KCJDb25kaXRpb24iLCBsYWJlbHM9YygiV2ludGVyIE5pZ2h0IiwgIlN1bW1lciBEYXkiKSwgZ3VpZGUgPSAibGVnZW5kIikgKw0KICBnZ3JlcGVsOjpnZW9tX3RleHRfcmVwZWwoYWVzKHg9RGltLjEsIHk9RGltLjIsIGNvbG91cj1jb2xvciwgbGFiZWw9c2FtcGxlKSwgDQogICAgICAgICAgICAgICAgICAgICAgICAgICBudWRnZV95ID0gLTIsICBzZWVkID0gNDIsIHNlZ21lbnQuc2l6ZT0wLjEsIHNob3cubGVnZW5kID0gRkFMU0UpICsNCiAgeGxhYihEMV90ZXh0KSArIA0KICB5bGFiKEQyX3RleHQpICsNCiAgZ2d0aXRsZSgiUENBIG1ldGF0cmFuc2NyaXB0b21pY3MgIFxuRC4gaHVuZ2FyaWNhIikgKw0KICBsYWJzKGNhcHRpb24gPSAgcGFzdGUwKCJQRVJNQU5PVkEgb24gY29uZGl0aW9uOiBwLXZhbHVlOiAiLCBwdikpICsgDQogIHRoZW1lKHBhbmVsLmJhY2tncm91bmQ9IGVsZW1lbnRfcmVjdChmaWxsPSIjZjRmNGY0IiksDQogICAgICAgIHBsb3QuY2FwdGlvbiA9IGVsZW1lbnRfdGV4dChzaXplPTcpKSAtPiBwMQ0KIA0KDQpnZ3Bsb3QocGNhX2RhdGEpICsNCiAgZ2VvbV9wb2ludChhZXMoeD1EaW0uMSwgeT1EaW0uMywgY29sb3VyPWNvbG9yKSwgc2l6ZT0zKSArDQogIHNjYWxlX2NvbG91cl9pZGVudGl0eSgiQ29uZGl0aW9uIiwgbGFiZWxzPWMoIldpbnRlciBOaWdodCIsICJTdW1tZXIgRGF5IiksIGd1aWRlID0gImxlZ2VuZCIpICsNCiAgZ2dyZXBlbDo6Z2VvbV90ZXh0X3JlcGVsKGFlcyh4PURpbS4xLCB5PURpbS4zLCBjb2xvdXI9Y29sb3IsIGxhYmVsPXNhbXBsZSksIA0KICAgICAgICAgICAgICAgICAgICAgICAgICAgbnVkZ2VfeSA9IC0yLCAgc2VlZCA9IDQyLCBzZWdtZW50LnNpemU9MC4xLCBzaG93LmxlZ2VuZCA9IEZBTFNFKSArDQogIHhsYWIoRDFfdGV4dCkgKyANCiAgeWxhYihEM190ZXh0KSArDQogIGdndGl0bGUoIlBDQSBtZXRhdHJhbnNjcmlwdG9taWNzICBcbkQuIGh1bmdhcmljYSIpICsNCiAgbGFicyhjYXB0aW9uID0gIHBhc3RlMCgiUEVSTUFOT1ZBIG9uIGNvbmRpdGlvbjogcC12YWx1ZTogIiwgcHYpKSArIA0KICB0aGVtZShwYW5lbC5iYWNrZ3JvdW5kPSBlbGVtZW50X3JlY3QoZmlsbD0iI2Y0ZjRmNCIpLA0KICAgICAgICBwbG90LmNhcHRpb24gPSBlbGVtZW50X3RleHQoc2l6ZT03KSkgLT4gcDINCg0KZ2dwbG90KHBjYV9kYXRhKSArDQogIGdlb21fcG9pbnQoYWVzKHg9RGltLjIsIHk9RGltLjMsIGNvbG91cj1jb2xvciksIHNpemU9MykgKw0KICBzY2FsZV9jb2xvdXJfaWRlbnRpdHkoIkNvbmRpdGlvbiIsIGxhYmVscz1jKCJXaW50ZXIgTmlnaHQiLCAiU3VtbWVyIERheSIpLCBndWlkZSA9ICJsZWdlbmQiKSArDQogIGdncmVwZWw6Omdlb21fdGV4dF9yZXBlbChhZXMoeD1EaW0uMiwgeT1EaW0uMywgY29sb3VyPWNvbG9yLCBsYWJlbD1zYW1wbGUpLCANCiAgICAgICAgICAgICAgICAgICAgICAgICAgIG51ZGdlX3kgPSAtMiwgIHNlZWQgPSA0Miwgc2VnbWVudC5zaXplPTAuMSwgc2hvdy5sZWdlbmQgPSBGQUxTRSkgKw0KICB4bGFiKEQyX3RleHQpICsgDQogIHlsYWIoRDNfdGV4dCkgKw0KICBnZ3RpdGxlKCJQQ0EgbWV0YXRyYW5zY3JpcHRvbWljcyAgXG5ELiBodW5nYXJpY2EiKSArDQogIGxhYnMoY2FwdGlvbiA9ICBwYXN0ZTAoIlBFUk1BTk9WQSBvbiBjb25kaXRpb246IHAtdmFsdWU6ICIsIHB2KSkgKyANCiAgdGhlbWUocGFuZWwuYmFja2dyb3VuZD0gZWxlbWVudF9yZWN0KGZpbGw9IiNmNGY0ZjQiKSwNCiAgICAgICAgcGxvdC5jYXB0aW9uID0gZWxlbWVudF90ZXh0KHNpemU9NykpIC0+IHAzDQoNCnBsb3RncmlkX1BDQSA8LSBnZ2FycmFuZ2UocDEsIHAyLCBwMywgbGFiZWxzID0gYygiQSIsICJCIiwgIkMiKSwgY29tbW9uLmxlZ2VuZD1UUlVFLCBsZWdlbmQ9ImJvdHRvbSIsIG5yb3c9MSkgDQoNCnByaW50KHBsb3RncmlkX1BDQSkNCg0KZ2dzYXZlKHBsb3QgPSBwbG90Z3JpZF9QQ0EsIGZpbGVuYW1lID0gIi4uL2ZpZ3VyZXMvUENBX21ldGF0cmFuc2NyaXB0b21pY3NfM19jb21wb3NhbnRlc19kaW9odS50aWZmIiwgZHBpID0gMzAwLCB3aWR0aCA9IDE1LCBoZWlnaHQgPSA1LCBiZz0id2hpdGUiKQ0KDQpnZ3NhdmUocGxvdD1wMSwgZmlsZW5hbWUgPSAiLi4vZmlndXJlcy9QQ0FfbWV0YXRyYW5zY3JpcHRvbWljc19kaW9odS50aWZmIiwgZHBpID0gMzAwLCB3aWR0aCA9IDYsIGhlaWdodCA9IDUsIGJnPSJ3aGl0ZSIpDQpgYGANCg0KYGBge3IgM0RfUENBX2Nscl9kaW9odSwgd2ViZ2w9VFJVRSwgbWVzc2FnZT1GQUxTRSwgd2FybmluZz1GQUxTRSwgZmlnLmhlaWdodD03LCBmaWcud2lkdGg9N30NCg0Kcm93X2Nvb3JkIDwtIHJlc19wY2EkaW5kJGNvb3JkDQoNCg0KaW52aXNpYmxlKHJnbDo6b3BlbjNkKCkpDQoNCiNyZ2w6OmJnM2QoImxpZ2h0Z3JheSIpDQpyZ2w6OnBsb3QzZCh4PXJvd19jb29yZFtjKCJXTl9URl8xIiwgIldOX1RGXzIiLCAiV05fVEZfMyIpLCAxXSwgDQogICAgICAgICAgICB5PXJvd19jb29yZFtjKCJXTl9URl8xIiwgIldOX1RGXzIiLCAiV05fVEZfMyIpLCAyXSwgDQogICAgICAgICAgICB6PXJvd19jb29yZFtjKCJXTl9URl8xIiwgIldOX1RGXzIiLCAiV05fVEZfMyIpLCAzXSwNCiAgICAgICAgICAgIHhsYWIgPSBwYXN0ZTAoY29sbmFtZXMocm93X2Nvb3JkKVsxXSAsICIgKCIgLCBzdWJzdHIoYXMuY2hhcmFjdGVyKHJlc19wY2EkZWlnWywyXVsxXSksIDEsIDUpICwgIiUpIiksDQogICAgICAgICAgICB5bGFiID0gcGFzdGUwKGNvbG5hbWVzKHJvd19jb29yZClbMl0gLCAiICgiICwgc3Vic3RyKGFzLmNoYXJhY3RlcihyZXNfcGNhJGVpZ1ssMl1bMl0pLCAxLCA1KSAsICIlKSIpLA0KICAgICAgICAgICAgemxhYiA9IHBhc3RlMChjb2xuYW1lcyhyb3dfY29vcmQpWzNdICwgIiAoIiAsIHN1YnN0cihhcy5jaGFyYWN0ZXIocmVzX3BjYSRlaWdbLDJdWzNdKSwgMSwgNSkgLCAiJSkiKSwNCiAgICAgICAgICAgIHhsaW0gPSBjKG1pbihyb3dfY29vcmRbLCAxXSksIG1heChyb3dfY29vcmRbLCAxXSkrMTApLCANCiAgICAgICAgICAgIHlsaW0gPSBjKG1pbihyb3dfY29vcmRbLCAyXSksIG1heChyb3dfY29vcmRbLCAyXSkrMTApLCANCiAgICAgICAgICAgIHpsaW0gPSBjKG1pbihyb3dfY29vcmRbLCAzXSksIG1heChyb3dfY29vcmRbLCAzXSkrMTApLA0KICAgICAgICAgICAgY29sID0gImRhcmtjeWFuIiwNCiAgICAgICAgICAgIHNpemU9MTAsDQogICAgICAgICAgICBtYWluID0gIlBDQSBELiBodW5nYXJpY2EiKQ0KDQpyZ2w6OnRleHQzZCh4PXJvd19jb29yZFtjKCJXTl9URl8xIiwgIldOX1RGXzIiLCAiV05fVEZfMyIpLCAxXSs1LCANCiAgICAgICAgICAgIHk9cm93X2Nvb3JkW2MoIldOX1RGXzEiLCAiV05fVEZfMiIsICJXTl9URl8zIiksIDJdKzUsIA0KICAgICAgICAgICAgej1yb3dfY29vcmRbYygiV05fVEZfMSIsICJXTl9URl8yIiwgIldOX1RGXzMiKSwgM10rNSwNCiAgICAgICAgICAgIHRleHQ9YygiV05fVEZfMSIsICJXTl9URl8yIiwgIldOX1RGXzMiKSwNCiAgICAgICAgICAgIGNvbCA9ICJkYXJrY3lhbiIsDQogICAgICAgICAgICBzaXplPTEwKQ0KDQoNCnJnbDo6cG9pbnRzM2QoeD1yb3dfY29vcmRbYygiU0RfVEZfMSIsICJTRF9URl8yIiwgIlNEX1RGXzMiKSwgMV0sIA0KICAgICAgICAgICAgICB5PXJvd19jb29yZFtjKCJTRF9URl8xIiwgIlNEX1RGXzIiLCAiU0RfVEZfMyIpLCAyXSwgDQogICAgICAgICAgICAgIHo9cm93X2Nvb3JkW2MoIlNEX1RGXzEiLCAiU0RfVEZfMiIsICJTRF9URl8zIiksIDNdLA0KICAgICAgICAgICAgICBjb2wgPSAiZGFya29yYW5nZSIsDQogICAgICAgICAgICAgIHNpemU9MTApDQoNCnJnbDo6dGV4dDNkKHg9cm93X2Nvb3JkW2MoIlNEX1RGXzEiLCAiU0RfVEZfMiIsICJTRF9URl8zIiksIDFdKzUsIA0KICAgICAgICAgICAgeT1yb3dfY29vcmRbYygiU0RfVEZfMSIsICJTRF9URl8yIiwgIlNEX1RGXzMiKSwgMl0rNSwgDQogICAgICAgICAgICB6PXJvd19jb29yZFtjKCJTRF9URl8xIiwgIlNEX1RGXzIiLCAiU0RfVEZfMyIpLCAzXSs1LA0KICAgICAgICAgICAgdGV4dD1jKCJTRF9URl8xIiwgIlNEX1RGXzIiLCAiU0RfVEZfMyIpLA0KICAgICAgICAgICAgY29sID0gImRhcmtvcmFuZ2UiLA0KICAgICAgICAgICAgc2l6ZT0xMCkNCg0KIyMgQWN0aXZhdGUgZm9yIG9yIGdpZiBnZW5lcmF0aW9uDQojcGFyM2Qod2luZG93UmVjdCA9IGMoMjAsIDMwLCA4MDAsIDgwMCkpDQoNCiNyZ2w6Om1vdmllM2QoDQojICBtb3ZpZT0iM0RBbmltYXRlZF9QQ0FfZGlvaHUiLCANCiMgIHNwaW4zZCggYXhpcyA9IGMoMCwgMCwgMSksIHJwbSA9IDMpLA0KIyAgZHVyYXRpb24gPSAyMCwgDQojICBkaXIgPSAiLi4vZmlndXJlcyIsDQojICB0eXBlID0gImdpZiIsIA0KIyAgY2xlYW4gPSBUUlVFLCB3ZWJzaG90PUZBTFNFLCBmcHM9MTApDQpgYGANCg0KIyMjIyAqUHNldWRvbW9uYXMgZ3JhbWluaXMqDQoNCmBgYHtyIFBDQV9jbHJfcHNlZ3J9DQoNCnJlc19wY2EgPC0gUENBKHQocHNlZ3JfY2xyKSwgc2NhbGUudW5pdCA9IFRSVUUsIGdyYXBoID0gRkFMU0UpDQoNCmBgYA0KDQpgYGB7ciBQRVJNQU5PVkFfcHNlZ3J9DQpjb3VudHNfZGF0YSA8LSB0KHBzZWdyX2NscikNCm1ldGFkYXRhX3RtcCA8LSBkYXRhLmZyYW1lKG1ldGFkYXRhX3RhYmxlLCBzdHJpbmdzQXNGYWN0b3JzID0gVFJVRSkNCnBlcm1hbm92YSA8LSBhZG9uaXMyKGZvcm11bGEgPSBhcy5mb3JtdWxhKHBhc3RlMCgiY291bnRzX2RhdGF+IiwgImNvbmRpdGlvbiIpKSwgDQogICAgICAgICAgICAgICAgICAgICBkYXRhID0gbWV0YWRhdGFfdG1wLCBtZXRob2Q9ImV1Y2xpZGVhbiIsICMgbWV0aG9kPSJicmF5IiwgI290dV9kYXRhflRJTUUNCiAgICAgICAgICAgICAgICAgICAgIHBlcm11dGF0aW9ucyA9IDk5OSwgc3FydC5kaXN0ID0gRkFMU0UsIGFkZCA9IEZBTFNFLCBieSA9ICJ0ZXJtcyIpDQpwdiA9IHBlcm1hbm92YSRgUHIoPkYpYFsxXQ0KIyBwcmludChwdikNCg0Kcm0oY291bnRzX2RhdGEsIG1ldGFkYXRhX3RtcCkNCmBgYA0KDQpgYGB7ciBQQ0FfMkRfcGxvdHNfcHNlZ3IsIGZpZy5oZWlnaHQ9NCwgZmlnLndpZHRoPTl9DQoNCnBjYV90bXAgPC0gcmJpbmQocmVzX3BjYSRpbmQkY29vcmQsDQogICAgICAgICAgICAgICAgIHJlc19wY2EkaW5kLnN1cCRjb29yZCkgJT4lIGRhdGEuZnJhbWUoKSAlPiUgDQogIHJvd25hbWVzX3RvX2NvbHVtbigic2FtcGxlIikNCg0KDQpwY2FfZGF0YSA8LSBsZWZ0X2pvaW4ocGNhX3RtcCwgcm93bmFtZXNfdG9fY29sdW1uKG1ldGFkYXRhX3RhYmxlKSwgYnk9am9pbl9ieSgic2FtcGxlIiA9PSAicm93bmFtZSIpKQ0Kcm0ocGNhX3RtcCkNCg0KRDFfdGV4dCA9IHBhc3RlMCgiRGltIDEgKCIsIA0KICAgICAgICAgICAgICAgICByb3VuZChkYXRhLmZyYW1lKHJlc19wY2EkZWlnKSRwZXJjZW50YWdlLm9mLnZhcmlhbmNlWzFdLCBkaWdpdHM9MiksIA0KICAgICAgICAgICAgICAgICAiJSkiKQ0KRDJfdGV4dCA9IHBhc3RlMCgiRGltIDIgKCIsIA0KICAgICAgICAgICAgICAgICByb3VuZChkYXRhLmZyYW1lKHJlc19wY2EkZWlnKSRwZXJjZW50YWdlLm9mLnZhcmlhbmNlWzJdLCBkaWdpdHM9MiksIA0KICAgICAgICAgICAgICAgICAiJSkiKQ0KRDNfdGV4dCA9IHBhc3RlMCgiRGltIDMgKCIsIA0KICAgICAgICAgICAgICAgICByb3VuZChkYXRhLmZyYW1lKHJlc19wY2EkZWlnKSRwZXJjZW50YWdlLm9mLnZhcmlhbmNlWzNdLCBkaWdpdHM9MiksIA0KICAgICAgICAgICAgICAgICAiJSkiKQ0KDQpnZ3Bsb3QocGNhX2RhdGEpICsNCiAgZ2VvbV9wb2ludChhZXMoeD1EaW0uMSwgeT1EaW0uMiwgY29sb3VyPWNvbG9yLCApLCBzaXplPTMpICsNCiAgc2NhbGVfY29sb3VyX2lkZW50aXR5KCJDb25kaXRpb24iLCBsYWJlbHM9YygiV2ludGVyIE5pZ2h0IiwgIlN1bW1lciBEYXkiKSwgZ3VpZGUgPSAibGVnZW5kIikgKw0KICBnZ3JlcGVsOjpnZW9tX3RleHRfcmVwZWwoYWVzKHg9RGltLjEsIHk9RGltLjIsIGNvbG91cj1jb2xvciwgbGFiZWw9c2FtcGxlKSwgDQogICAgICAgICAgICAgICAgICAgICAgICAgICBudWRnZV95ID0gLTIsICBzZWVkID0gNDIsIHNlZ21lbnQuc2l6ZT0wLjEsIHNob3cubGVnZW5kID0gRkFMU0UpICsNCiAgeGxhYihEMV90ZXh0KSArIA0KICB5bGFiKEQyX3RleHQpICsNCiAgZ2d0aXRsZSgiUENBIG1ldGF0cmFuc2NyaXB0b21pY3MgIFxuUC4gZ3JhbWluaXMiKSArDQogIGxhYnMoY2FwdGlvbiA9ICBwYXN0ZTAoIlBFUk1BTk9WQSBvbiBjb25kaXRpb246IHAtdmFsdWU6ICIsIHB2KSkgKyANCiAgdGhlbWUocGFuZWwuYmFja2dyb3VuZD0gZWxlbWVudF9yZWN0KGZpbGw9IiNmNGY0ZjQiKSwNCiAgICAgICAgcGxvdC5jYXB0aW9uID0gZWxlbWVudF90ZXh0KHNpemU9NykpIC0+IHAxDQogDQoNCmdncGxvdChwY2FfZGF0YSkgKw0KICBnZW9tX3BvaW50KGFlcyh4PURpbS4xLCB5PURpbS4zLCBjb2xvdXI9Y29sb3IpLCBzaXplPTMpICsNCiAgc2NhbGVfY29sb3VyX2lkZW50aXR5KCJDb25kaXRpb24iLCBsYWJlbHM9YygiV2ludGVyIE5pZ2h0IiwgIlN1bW1lciBEYXkiKSwgZ3VpZGUgPSAibGVnZW5kIikgKw0KICBnZ3JlcGVsOjpnZW9tX3RleHRfcmVwZWwoYWVzKHg9RGltLjEsIHk9RGltLjMsIGNvbG91cj1jb2xvciwgbGFiZWw9c2FtcGxlKSwgDQogICAgICAgICAgICAgICAgICAgICAgICAgICBudWRnZV95ID0gLTIsICBzZWVkID0gNDIsIHNlZ21lbnQuc2l6ZT0wLjEsIHNob3cubGVnZW5kID0gRkFMU0UpICsNCiAgeGxhYihEMV90ZXh0KSArIA0KICB5bGFiKEQzX3RleHQpICsNCiAgZ2d0aXRsZSgiUENBIG1ldGF0cmFuc2NyaXB0b21pY3MgIFxuUC4gZ3JhbWluaXMiKSArDQogIGxhYnMoY2FwdGlvbiA9ICBwYXN0ZTAoIlBFUk1BTk9WQSBvbiBjb25kaXRpb246IHAtdmFsdWU6ICIsIHB2KSkgKyANCiAgdGhlbWUocGFuZWwuYmFja2dyb3VuZD0gZWxlbWVudF9yZWN0KGZpbGw9IiNmNGY0ZjQiKSwNCiAgICAgICAgcGxvdC5jYXB0aW9uID0gZWxlbWVudF90ZXh0KHNpemU9NykpIC0+IHAyDQoNCmdncGxvdChwY2FfZGF0YSkgKw0KICBnZW9tX3BvaW50KGFlcyh4PURpbS4yLCB5PURpbS4zLCBjb2xvdXI9Y29sb3IpLCBzaXplPTMpICsNCiAgc2NhbGVfY29sb3VyX2lkZW50aXR5KCJDb25kaXRpb24iLCBsYWJlbHM9YygiV2ludGVyIE5pZ2h0IiwgIlN1bW1lciBEYXkiKSwgZ3VpZGUgPSAibGVnZW5kIikgKw0KICBnZ3JlcGVsOjpnZW9tX3RleHRfcmVwZWwoYWVzKHg9RGltLjIsIHk9RGltLjMsIGNvbG91cj1jb2xvciwgbGFiZWw9c2FtcGxlKSwgDQogICAgICAgICAgICAgICAgICAgICAgICAgICBudWRnZV95ID0gLTIsICBzZWVkID0gNDIsIHNlZ21lbnQuc2l6ZT0wLjEsIHNob3cubGVnZW5kID0gRkFMU0UpICsNCiAgeGxhYihEMl90ZXh0KSArIA0KICB5bGFiKEQzX3RleHQpICsNCiAgZ2d0aXRsZSgiUENBIG1ldGF0cmFuc2NyaXB0b21pY3MgIFxuUC4gZ3JhbWluaXMiKSArDQogIGxhYnMoY2FwdGlvbiA9ICBwYXN0ZTAoIlBFUk1BTk9WQSBvbiBjb25kaXRpb246IHAtdmFsdWU6ICIsIHB2KSkgKyANCiAgdGhlbWUocGFuZWwuYmFja2dyb3VuZD0gZWxlbWVudF9yZWN0KGZpbGw9IiNmNGY0ZjQiKSwNCiAgICAgICAgcGxvdC5jYXB0aW9uID0gZWxlbWVudF90ZXh0KHNpemU9NykpIC0+IHAzDQoNCnBsb3RncmlkX1BDQSA8LSBnZ2FycmFuZ2UocDEsIHAyLCBwMywgbGFiZWxzID0gYygiQSIsICJCIiwgIkMiKSwgY29tbW9uLmxlZ2VuZD1UUlVFLCBsZWdlbmQ9ImJvdHRvbSIsIG5yb3c9MSkgDQoNCnByaW50KHBsb3RncmlkX1BDQSkNCg0KZ2dzYXZlKHBsb3QgPSBwbG90Z3JpZF9QQ0EsIGZpbGVuYW1lID0gIi4uL2ZpZ3VyZXMvUENBX21ldGF0cmFuc2NyaXB0b21pY3NfM19jb21wb3NhbnRlc19wc2Vnci50aWZmIiwgZHBpID0gMzAwLCB3aWR0aCA9IDE1LCBoZWlnaHQgPSA1LCBiZz0id2hpdGUiKQ0KDQpnZ3NhdmUocGxvdD1wMSwgZmlsZW5hbWUgPSAiLi4vZmlndXJlcy9QQ0FfbWV0YXRyYW5zY3JpcHRvbWljc19wc2Vnci50aWZmIiwgZHBpID0gMzAwLCB3aWR0aCA9IDYsIGhlaWdodCA9IDUsIGJnPSJ3aGl0ZSIpDQpgYGANCg0KYGBge3IgM0RfUENBX2Nscl9wc2Vnciwgd2ViZ2w9VFJVRSwgbWVzc2FnZT1GQUxTRSwgd2FybmluZz1GQUxTRSwgZmlnLmhlaWdodD03LCBmaWcud2lkdGg9N30NCg0Kcm93X2Nvb3JkIDwtIHJlc19wY2EkaW5kJGNvb3JkDQoNCmludmlzaWJsZShyZ2w6Om9wZW4zZCgpKQ0KDQojcmdsOjpiZzNkKCJsaWdodGdyYXkiKQ0KcmdsOjpwbG90M2QoeD1yb3dfY29vcmRbYygiV05fVEZfMSIsICJXTl9URl8yIiwgIldOX1RGXzMiKSwgMV0sIA0KICAgICAgICAgICAgeT1yb3dfY29vcmRbYygiV05fVEZfMSIsICJXTl9URl8yIiwgIldOX1RGXzMiKSwgMl0sIA0KICAgICAgICAgICAgej1yb3dfY29vcmRbYygiV05fVEZfMSIsICJXTl9URl8yIiwgIldOX1RGXzMiKSwgM10sDQogICAgICAgICAgICB4bGFiID0gcGFzdGUwKGNvbG5hbWVzKHJvd19jb29yZClbMV0gLCAiICgiICwgc3Vic3RyKGFzLmNoYXJhY3RlcihyZXNfcGNhJGVpZ1ssMl1bMV0pLCAxLCA1KSAsICIlKSIpLA0KICAgICAgICAgICAgeWxhYiA9IHBhc3RlMChjb2xuYW1lcyhyb3dfY29vcmQpWzJdICwgIiAoIiAsIHN1YnN0cihhcy5jaGFyYWN0ZXIocmVzX3BjYSRlaWdbLDJdWzJdKSwgMSwgNSkgLCAiJSkiKSwNCiAgICAgICAgICAgIHpsYWIgPSBwYXN0ZTAoY29sbmFtZXMocm93X2Nvb3JkKVszXSAsICIgKCIgLCBzdWJzdHIoYXMuY2hhcmFjdGVyKHJlc19wY2EkZWlnWywyXVszXSksIDEsIDUpICwgIiUpIiksDQogICAgICAgICAgICB4bGltID0gYyhtaW4ocm93X2Nvb3JkWywgMV0pLCBtYXgocm93X2Nvb3JkWywgMV0pKzEwKSwgDQogICAgICAgICAgICB5bGltID0gYyhtaW4ocm93X2Nvb3JkWywgMl0pLCBtYXgocm93X2Nvb3JkWywgMl0pKzEwKSwgDQogICAgICAgICAgICB6bGltID0gYyhtaW4ocm93X2Nvb3JkWywgM10pLCBtYXgocm93X2Nvb3JkWywgM10pKzEwKSwNCiAgICAgICAgICAgIGNvbCA9ICJkYXJrY3lhbiIsDQogICAgICAgICAgICBzaXplPTEwLA0KICAgICAgICAgICAgbWFpbiA9ICJQQ0EgUC4gZ3JhbWluaXMiKQ0KDQpyZ2w6OnRleHQzZCh4PXJvd19jb29yZFtjKCJXTl9URl8xIiwgIldOX1RGXzIiLCAiV05fVEZfMyIpLCAxXSs1LCANCiAgICAgICAgICAgIHk9cm93X2Nvb3JkW2MoIldOX1RGXzEiLCAiV05fVEZfMiIsICJXTl9URl8zIiksIDJdKzUsIA0KICAgICAgICAgICAgej1yb3dfY29vcmRbYygiV05fVEZfMSIsICJXTl9URl8yIiwgIldOX1RGXzMiKSwgM10rNSwNCiAgICAgICAgICAgIHRleHQ9YygiV05fVEZfMSIsICJXTl9URl8yIiwgIldOX1RGXzMiKSwNCiAgICAgICAgICAgIGNvbCA9ICJkYXJrY3lhbiIsDQogICAgICAgICAgICBzaXplPTEwKQ0KDQoNCnJnbDo6cG9pbnRzM2QoeD1yb3dfY29vcmRbYygiU0RfVEZfMSIsICJTRF9URl8yIiwgIlNEX1RGXzMiKSwgMV0sIA0KICAgICAgICAgICAgICB5PXJvd19jb29yZFtjKCJTRF9URl8xIiwgIlNEX1RGXzIiLCAiU0RfVEZfMyIpLCAyXSwgDQogICAgICAgICAgICAgIHo9cm93X2Nvb3JkW2MoIlNEX1RGXzEiLCAiU0RfVEZfMiIsICJTRF9URl8zIiksIDNdLA0KICAgICAgICAgICAgICBjb2wgPSAiZGFya29yYW5nZSIsDQogICAgICAgICAgICAgIHNpemU9MTApDQoNCnJnbDo6dGV4dDNkKHg9cm93X2Nvb3JkW2MoIlNEX1RGXzEiLCAiU0RfVEZfMiIsICJTRF9URl8zIiksIDFdKzMsIA0KICAgICAgICAgICAgeT1yb3dfY29vcmRbYygiU0RfVEZfMSIsICJTRF9URl8yIiwgIlNEX1RGXzMiKSwgMl0rMywgDQogICAgICAgICAgICB6PXJvd19jb29yZFtjKCJTRF9URl8xIiwgIlNEX1RGXzIiLCAiU0RfVEZfMyIpLCAzXSszLA0KICAgICAgICAgICAgdGV4dD1jKCJTRF9URl8xIiwgIlNEX1RGXzIiLCAiU0RfVEZfMyIpLA0KICAgICAgICAgICAgY29sID0gImRhcmtvcmFuZ2UiLA0KICAgICAgICAgICAgc2l6ZT0xMCkNCg0KIyMgQWN0aXZhdGUgZm9yIG9yIGdpZiBnZW5lcmF0aW9uDQojcGFyM2Qod2luZG93UmVjdCA9IGMoMjAsIDMwLCA4MDAsIDgwMCkpDQoNCiNyZ2w6Om1vdmllM2QoDQojICBtb3ZpZT0iM0RBbmltYXRlZF9QQ0FfcHNlZ3IiLCANCiMgIHNwaW4zZCggYXhpcyA9IGMoMCwgMCwgMSksIHJwbSA9IDMpLA0KIyAgZHVyYXRpb24gPSAyMCwgDQojICBkaXIgPSAiLi4vZmlndXJlcyIsDQojICB0eXBlID0gImdpZiIsIA0KIyAgY2xlYW4gPSBUUlVFLCB3ZWJzaG90PUZBTFNFLCBmcHM9MTApDQpgYGANCg0KIyMjIyAqUHNldWRvbW9uYXMgc3lyaW5nYWUqDQoNCmBgYHtyIFBDQV9jbHJfcHNlc3l9DQoNCnJlc19wY2EgPC0gUENBKHQocHNlc3lfY2xyKSwgc2NhbGUudW5pdCA9IFRSVUUsIGdyYXBoID0gRkFMU0UpDQpgYGANCg0KYGBge3IgUEVSTUFOT1ZBX3BzZXN5fQ0KY291bnRzX2RhdGEgPC0gdChwc2VzeV9jbHIpDQptZXRhZGF0YV90bXAgPC0gZGF0YS5mcmFtZShtZXRhZGF0YV90YWJsZSwgc3RyaW5nc0FzRmFjdG9ycyA9IFRSVUUpDQpwZXJtYW5vdmEgPC0gYWRvbmlzMihmb3JtdWxhID0gYXMuZm9ybXVsYShwYXN0ZTAoImNvdW50c19kYXRhfiIsICJjb25kaXRpb24iKSksIA0KICAgICAgICAgICAgICAgICAgICAgZGF0YSA9IG1ldGFkYXRhX3RtcCwgbWV0aG9kPSJldWNsaWRlYW4iLCAjIG1ldGhvZD0iYnJheSIsICNvdHVfZGF0YX5USU1FDQogICAgICAgICAgICAgICAgICAgICBwZXJtdXRhdGlvbnMgPSA5OTksIHNxcnQuZGlzdCA9IEZBTFNFLCBhZGQgPSBGQUxTRSwgYnkgPSAidGVybXMiKQ0KcHYgPSBwZXJtYW5vdmEkYFByKD5GKWBbMV0NCiMgcHJpbnQocHYpDQoNCnJtKGNvdW50c19kYXRhLCBtZXRhZGF0YV90bXApDQpgYGANCg0KYGBge3IgUENBXzJEX3Bsb3RzX3BzZXN5LCBmaWcuaGVpZ2h0PTQsIGZpZy53aWR0aD05fQ0KDQpwY2FfdG1wIDwtIHJiaW5kKHJlc19wY2EkaW5kJGNvb3JkLA0KICAgICAgICAgICAgICAgICByZXNfcGNhJGluZC5zdXAkY29vcmQpICU+JSBkYXRhLmZyYW1lKCkgJT4lIA0KICByb3duYW1lc190b19jb2x1bW4oInNhbXBsZSIpDQoNCg0KcGNhX2RhdGEgPC0gbGVmdF9qb2luKHBjYV90bXAsIHJvd25hbWVzX3RvX2NvbHVtbihtZXRhZGF0YV90YWJsZSksIGJ5PWpvaW5fYnkoInNhbXBsZSIgPT0gInJvd25hbWUiKSkNCnJtKHBjYV90bXApDQoNCkQxX3RleHQgPSBwYXN0ZTAoIkRpbSAxICgiLCANCiAgICAgICAgICAgICAgICAgcm91bmQoZGF0YS5mcmFtZShyZXNfcGNhJGVpZykkcGVyY2VudGFnZS5vZi52YXJpYW5jZVsxXSwgZGlnaXRzPTIpLCANCiAgICAgICAgICAgICAgICAgIiUpIikNCkQyX3RleHQgPSBwYXN0ZTAoIkRpbSAyICgiLCANCiAgICAgICAgICAgICAgICAgcm91bmQoZGF0YS5mcmFtZShyZXNfcGNhJGVpZykkcGVyY2VudGFnZS5vZi52YXJpYW5jZVsyXSwgZGlnaXRzPTIpLCANCiAgICAgICAgICAgICAgICAgIiUpIikNCkQzX3RleHQgPSBwYXN0ZTAoIkRpbSAzICgiLCANCiAgICAgICAgICAgICAgICAgcm91bmQoZGF0YS5mcmFtZShyZXNfcGNhJGVpZykkcGVyY2VudGFnZS5vZi52YXJpYW5jZVszXSwgZGlnaXRzPTIpLCANCiAgICAgICAgICAgICAgICAgIiUpIikNCg0KZ2dwbG90KHBjYV9kYXRhKSArDQogIGdlb21fcG9pbnQoYWVzKHg9RGltLjEsIHk9RGltLjIsIGNvbG91cj1jb2xvciwgKSwgc2l6ZT0zKSArDQogIHNjYWxlX2NvbG91cl9pZGVudGl0eSgiQ29uZGl0aW9uIiwgbGFiZWxzPWMoIldpbnRlciBOaWdodCIsICJTdW1tZXIgRGF5IiksIGd1aWRlID0gImxlZ2VuZCIpICsNCiAgZ2dyZXBlbDo6Z2VvbV90ZXh0X3JlcGVsKGFlcyh4PURpbS4xLCB5PURpbS4yLCBjb2xvdXI9Y29sb3IsIGxhYmVsPXNhbXBsZSksIA0KICAgICAgICAgICAgICAgICAgICAgICAgICAgbnVkZ2VfeSA9IC0yLCAgc2VlZCA9IDQyLCBzZWdtZW50LnNpemU9MC4xLCBzaG93LmxlZ2VuZCA9IEZBTFNFKSArDQogIHhsYWIoRDFfdGV4dCkgKyANCiAgeWxhYihEMl90ZXh0KSArDQogIGdndGl0bGUoIlBDQSBtZXRhdHJhbnNjcmlwdG9taWNzICBcblAuIHN5cmluZ2FlIikgKw0KICBsYWJzKGNhcHRpb24gPSAgcGFzdGUwKCJQRVJNQU5PVkEgb24gY29uZGl0aW9uOiBwLXZhbHVlOiAiLCBwdikpICsgDQogIHRoZW1lKHBhbmVsLmJhY2tncm91bmQ9IGVsZW1lbnRfcmVjdChmaWxsPSIjZjRmNGY0IiksDQogICAgICAgIHBsb3QuY2FwdGlvbiA9IGVsZW1lbnRfdGV4dChzaXplPTcpKSAtPiBwMQ0KIA0KDQpnZ3Bsb3QocGNhX2RhdGEpICsNCiAgZ2VvbV9wb2ludChhZXMoeD1EaW0uMSwgeT1EaW0uMywgY29sb3VyPWNvbG9yKSwgc2l6ZT0zKSArDQogIHNjYWxlX2NvbG91cl9pZGVudGl0eSgiQ29uZGl0aW9uIiwgbGFiZWxzPWMoIldpbnRlciBOaWdodCIsICJTdW1tZXIgRGF5IiksIGd1aWRlID0gImxlZ2VuZCIpICsNCiAgZ2dyZXBlbDo6Z2VvbV90ZXh0X3JlcGVsKGFlcyh4PURpbS4xLCB5PURpbS4zLCBjb2xvdXI9Y29sb3IsIGxhYmVsPXNhbXBsZSksIA0KICAgICAgICAgICAgICAgICAgICAgICAgICAgbnVkZ2VfeSA9IC0yLCAgc2VlZCA9IDQyLCBzZWdtZW50LnNpemU9MC4xLCBzaG93LmxlZ2VuZCA9IEZBTFNFKSArDQogIHhsYWIoRDFfdGV4dCkgKyANCiAgeWxhYihEM190ZXh0KSArDQogIGdndGl0bGUoIlBDQSBtZXRhdHJhbnNjcmlwdG9taWNzICBcblAuIHN5cmluZ2FlIikgKw0KICBsYWJzKGNhcHRpb24gPSAgcGFzdGUwKCJQRVJNQU5PVkEgb24gY29uZGl0aW9uOiBwLXZhbHVlOiAiLCBwdikpICsgDQogIHRoZW1lKHBhbmVsLmJhY2tncm91bmQ9IGVsZW1lbnRfcmVjdChmaWxsPSIjZjRmNGY0IiksDQogICAgICAgIHBsb3QuY2FwdGlvbiA9IGVsZW1lbnRfdGV4dChzaXplPTcpKSAtPiBwMg0KDQpnZ3Bsb3QocGNhX2RhdGEpICsNCiAgZ2VvbV9wb2ludChhZXMoeD1EaW0uMiwgeT1EaW0uMywgY29sb3VyPWNvbG9yKSwgc2l6ZT0zKSArDQogIHNjYWxlX2NvbG91cl9pZGVudGl0eSgiQ29uZGl0aW9uIiwgbGFiZWxzPWMoIldpbnRlciBOaWdodCIsICJTdW1tZXIgRGF5IiksIGd1aWRlID0gImxlZ2VuZCIpICsNCiAgZ2dyZXBlbDo6Z2VvbV90ZXh0X3JlcGVsKGFlcyh4PURpbS4yLCB5PURpbS4zLCBjb2xvdXI9Y29sb3IsIGxhYmVsPXNhbXBsZSksIA0KICAgICAgICAgICAgICAgICAgICAgICAgICAgbnVkZ2VfeSA9IC0yLCAgc2VlZCA9IDQyLCBzZWdtZW50LnNpemU9MC4xLCBzaG93LmxlZ2VuZCA9IEZBTFNFKSArDQogIHhsYWIoRDJfdGV4dCkgKyANCiAgeWxhYihEM190ZXh0KSArDQogIGdndGl0bGUoIlBDQSBtZXRhdHJhbnNjcmlwdG9taWNzICBcblAuIHN5cmluZ2FlIikgKw0KICBsYWJzKGNhcHRpb24gPSAgcGFzdGUwKCJQRVJNQU5PVkEgb24gY29uZGl0aW9uOiBwLXZhbHVlOiAiLCBwdikpICsgDQogIHRoZW1lKHBhbmVsLmJhY2tncm91bmQ9IGVsZW1lbnRfcmVjdChmaWxsPSIjZjRmNGY0IiksDQogICAgICAgIHBsb3QuY2FwdGlvbiA9IGVsZW1lbnRfdGV4dChzaXplPTcpKSAtPiBwMw0KDQpwbG90Z3JpZF9QQ0EgPC0gZ2dhcnJhbmdlKHAxLCBwMiwgcDMsIGxhYmVscyA9IGMoIkEiLCAiQiIsICJDIiksIGNvbW1vbi5sZWdlbmQ9VFJVRSwgbGVnZW5kPSJib3R0b20iLCBucm93PTEpIA0KDQpwcmludChwbG90Z3JpZF9QQ0EpDQoNCmdnc2F2ZShwbG90ID0gcGxvdGdyaWRfUENBLCBmaWxlbmFtZSA9ICIuLi9maWd1cmVzL1BDQV9tZXRhdHJhbnNjcmlwdG9taWNzXzNfY29tcG9zYW50ZXNfcHNlc3kudGlmZiIsIGRwaSA9IDMwMCwgd2lkdGggPSAxNSwgaGVpZ2h0ID0gNSwgYmc9IndoaXRlIikNCg0KZ2dzYXZlKHBsb3Q9cDEsIGZpbGVuYW1lID0gIi4uL2ZpZ3VyZXMvUENBX21ldGF0cmFuc2NyaXB0b21pY3NfcHNlc3kudGlmZiIsIGRwaSA9IDMwMCwgd2lkdGggPSA2LCBoZWlnaHQgPSA1LCBiZz0id2hpdGUiKQ0KYGBgDQoNCmBgYHtyIDNEX1BDQV9jbHJfcHNlc3ksIHdlYmdsPVRSVUUsIG1lc3NhZ2U9RkFMU0UsIHdhcm5pbmc9RkFMU0UsIGZpZy5oZWlnaHQ9NywgZmlnLndpZHRoPTd9DQoNCnJvd19jb29yZCA8LSByZXNfcGNhJGluZCRjb29yZA0KDQppbnZpc2libGUocmdsOjpvcGVuM2QoKSkNCg0KI3JnbDo6YmczZCgibGlnaHRncmF5IikNCnJnbDo6cGxvdDNkKHg9cm93X2Nvb3JkW2MoIldOX1RGXzEiLCAiV05fVEZfMiIsICJXTl9URl8zIiksIDFdLCANCiAgICAgICAgICAgIHk9cm93X2Nvb3JkW2MoIldOX1RGXzEiLCAiV05fVEZfMiIsICJXTl9URl8zIiksIDJdLCANCiAgICAgICAgICAgIHo9cm93X2Nvb3JkW2MoIldOX1RGXzEiLCAiV05fVEZfMiIsICJXTl9URl8zIiksIDNdLA0KICAgICAgICAgICAgeGxhYiA9IHBhc3RlMChjb2xuYW1lcyhyb3dfY29vcmQpWzFdICwgIiAoIiAsIHN1YnN0cihhcy5jaGFyYWN0ZXIocmVzX3BjYSRlaWdbLDJdWzFdKSwgMSwgNSkgLCAiJSkiKSwNCiAgICAgICAgICAgIHlsYWIgPSBwYXN0ZTAoY29sbmFtZXMocm93X2Nvb3JkKVsyXSAsICIgKCIgLCBzdWJzdHIoYXMuY2hhcmFjdGVyKHJlc19wY2EkZWlnWywyXVsyXSksIDEsIDUpICwgIiUpIiksDQogICAgICAgICAgICB6bGFiID0gcGFzdGUwKGNvbG5hbWVzKHJvd19jb29yZClbM10gLCAiICgiICwgc3Vic3RyKGFzLmNoYXJhY3RlcihyZXNfcGNhJGVpZ1ssMl1bM10pLCAxLCA1KSAsICIlKSIpLA0KICAgICAgICAgICAgeGxpbSA9IGMobWluKHJvd19jb29yZFssIDFdKSwgbWF4KHJvd19jb29yZFssIDFdKSsxMCksIA0KICAgICAgICAgICAgeWxpbSA9IGMobWluKHJvd19jb29yZFssIDJdKSwgbWF4KHJvd19jb29yZFssIDJdKSsxMCksIA0KICAgICAgICAgICAgemxpbSA9IGMobWluKHJvd19jb29yZFssIDNdKSwgbWF4KHJvd19jb29yZFssIDNdKSsxMCksDQogICAgICAgICAgICBjb2wgPSAiZGFya2N5YW4iLA0KICAgICAgICAgICAgc2l6ZT0xMCwNCiAgICAgICAgICAgIG1haW4gPSAiUENBIFAuIHN5cmluZ2FlIikNCg0KcmdsOjp0ZXh0M2QoeD1yb3dfY29vcmRbYygiV05fVEZfMSIsICJXTl9URl8yIiwgIldOX1RGXzMiKSwgMV0rMywgDQogICAgICAgICAgICB5PXJvd19jb29yZFtjKCJXTl9URl8xIiwgIldOX1RGXzIiLCAiV05fVEZfMyIpLCAyXSszLCANCiAgICAgICAgICAgIHo9cm93X2Nvb3JkW2MoIldOX1RGXzEiLCAiV05fVEZfMiIsICJXTl9URl8zIiksIDNdKzMsDQogICAgICAgICAgICB0ZXh0PWMoIldOX1RGXzEiLCAiV05fVEZfMiIsICJXTl9URl8zIiksDQogICAgICAgICAgICBjb2wgPSAiZGFya2N5YW4iLA0KICAgICAgICAgICAgc2l6ZT0xMCkNCg0KDQpyZ2w6OnBvaW50czNkKHg9cm93X2Nvb3JkW2MoIlNEX1RGXzEiLCAiU0RfVEZfMiIsICJTRF9URl8zIiksIDFdLCANCiAgICAgICAgICAgICAgeT1yb3dfY29vcmRbYygiU0RfVEZfMSIsICJTRF9URl8yIiwgIlNEX1RGXzMiKSwgMl0sIA0KICAgICAgICAgICAgICB6PXJvd19jb29yZFtjKCJTRF9URl8xIiwgIlNEX1RGXzIiLCAiU0RfVEZfMyIpLCAzXSwNCiAgICAgICAgICAgICAgY29sID0gImRhcmtvcmFuZ2UiLA0KICAgICAgICAgICAgICBzaXplPTEwKQ0KDQpyZ2w6OnRleHQzZCh4PXJvd19jb29yZFtjKCJTRF9URl8xIiwgIlNEX1RGXzIiLCAiU0RfVEZfMyIpLCAxXSszLCANCiAgICAgICAgICAgIHk9cm93X2Nvb3JkW2MoIlNEX1RGXzEiLCAiU0RfVEZfMiIsICJTRF9URl8zIiksIDJdKzMsIA0KICAgICAgICAgICAgej1yb3dfY29vcmRbYygiU0RfVEZfMSIsICJTRF9URl8yIiwgIlNEX1RGXzMiKSwgM10rMywNCiAgICAgICAgICAgIHRleHQ9YygiU0RfVEZfMSIsICJTRF9URl8yIiwgIlNEX1RGXzMiKSwNCiAgICAgICAgICAgIGNvbCA9ICJkYXJrb3JhbmdlIiwNCiAgICAgICAgICAgIHNpemU9MTApDQoNCg0KIyMgQWN0aXZhdGUgZm9yIG9yIGdpZiBnZW5lcmF0aW9uDQojcGFyM2Qod2luZG93UmVjdCA9IGMoMjAsIDMwLCA4MDAsIDgwMCkpDQoNCiNyZ2w6Om1vdmllM2QoDQojICBtb3ZpZT0iM0RBbmltYXRlZF9QQ0FfcHNlc3kiLCANCiMgIHNwaW4zZCggYXhpcyA9IGMoMCwgMCwgMSksIHJwbSA9IDMpLA0KIyAgZHVyYXRpb24gPSAyMCwgDQojICBkaXIgPSAiLi4vZmlndXJlcyIsDQojICB0eXBlID0gImdpZiIsIA0KIyAgY2xlYW4gPSBUUlVFLCB3ZWJzaG90PUZBTFNFLCBmcHM9MTApDQpgYGANCg0KIyMjIyAqUmhvZG9jb2NjdXMgZW5jbGVuc2lzKg0KDQpgYGB7ciBQQ0FfY2xyX3Job2VufQ0KDQpyZXNfcGNhIDwtIFBDQSh0KHJob2VuX2NsciksIHNjYWxlLnVuaXQgPSBUUlVFLCBncmFwaCA9IEZBTFNFKQ0KYGBgDQoNCg0KYGBge3IgUEVSTUFOT1ZBX3Job2VufQ0KY291bnRzX2RhdGEgPC0gdChyaG9lbl9jbHIpDQptZXRhZGF0YV90bXAgPC0gZGF0YS5mcmFtZShtZXRhZGF0YV90YWJsZSwgc3RyaW5nc0FzRmFjdG9ycyA9IFRSVUUpDQpwZXJtYW5vdmEgPC0gYWRvbmlzMihmb3JtdWxhID0gYXMuZm9ybXVsYShwYXN0ZTAoImNvdW50c19kYXRhfiIsICJjb25kaXRpb24iKSksIA0KICAgICAgICAgICAgICAgICAgICAgZGF0YSA9IG1ldGFkYXRhX3RtcCwgbWV0aG9kPSJldWNsaWRlYW4iLCAjIG1ldGhvZD0iYnJheSIsICNvdHVfZGF0YX5USU1FDQogICAgICAgICAgICAgICAgICAgICBwZXJtdXRhdGlvbnMgPSA5OTksIHNxcnQuZGlzdCA9IEZBTFNFLCBhZGQgPSBGQUxTRSwgYnkgPSAidGVybXMiKQ0KcHYgPSBwZXJtYW5vdmEkYFByKD5GKWBbMV0NCiMgcHJpbnQocHYpDQoNCnJtKGNvdW50c19kYXRhLCBtZXRhZGF0YV90bXApDQpgYGANCg0KYGBge3IgUENBXzJEX3Bsb3RzX3Job2VuLCBmaWcuaGVpZ2h0PTQsIGZpZy53aWR0aD05fQ0KDQpwY2FfdG1wIDwtIHJiaW5kKHJlc19wY2EkaW5kJGNvb3JkLA0KICAgICAgICAgICAgICAgICByZXNfcGNhJGluZC5zdXAkY29vcmQpICU+JSBkYXRhLmZyYW1lKCkgJT4lIA0KICByb3duYW1lc190b19jb2x1bW4oInNhbXBsZSIpDQoNCg0KcGNhX2RhdGEgPC0gbGVmdF9qb2luKHBjYV90bXAsIHJvd25hbWVzX3RvX2NvbHVtbihtZXRhZGF0YV90YWJsZSksIGJ5PWpvaW5fYnkoInNhbXBsZSIgPT0gInJvd25hbWUiKSkNCnJtKHBjYV90bXApDQoNCkQxX3RleHQgPSBwYXN0ZTAoIkRpbSAxICgiLCANCiAgICAgICAgICAgICAgICAgcm91bmQoZGF0YS5mcmFtZShyZXNfcGNhJGVpZykkcGVyY2VudGFnZS5vZi52YXJpYW5jZVsxXSwgZGlnaXRzPTIpLCANCiAgICAgICAgICAgICAgICAgIiUpIikNCkQyX3RleHQgPSBwYXN0ZTAoIkRpbSAyICgiLCANCiAgICAgICAgICAgICAgICAgcm91bmQoZGF0YS5mcmFtZShyZXNfcGNhJGVpZykkcGVyY2VudGFnZS5vZi52YXJpYW5jZVsyXSwgZGlnaXRzPTIpLCANCiAgICAgICAgICAgICAgICAgIiUpIikNCkQzX3RleHQgPSBwYXN0ZTAoIkRpbSAzICgiLCANCiAgICAgICAgICAgICAgICAgcm91bmQoZGF0YS5mcmFtZShyZXNfcGNhJGVpZykkcGVyY2VudGFnZS5vZi52YXJpYW5jZVszXSwgZGlnaXRzPTIpLCANCiAgICAgICAgICAgICAgICAgIiUpIikNCg0KZ2dwbG90KHBjYV9kYXRhKSArDQogIGdlb21fcG9pbnQoYWVzKHg9RGltLjEsIHk9RGltLjIsIGNvbG91cj1jb2xvciwgKSwgc2l6ZT0zKSArDQogIHNjYWxlX2NvbG91cl9pZGVudGl0eSgiQ29uZGl0aW9uIiwgbGFiZWxzPWMoIldpbnRlciBOaWdodCIsICJTdW1tZXIgRGF5IiksIGd1aWRlID0gImxlZ2VuZCIpICsNCiAgZ2dyZXBlbDo6Z2VvbV90ZXh0X3JlcGVsKGFlcyh4PURpbS4xLCB5PURpbS4yLCBjb2xvdXI9Y29sb3IsIGxhYmVsPXNhbXBsZSksIA0KICAgICAgICAgICAgICAgICAgICAgICAgICAgbnVkZ2VfeSA9IC0yLCAgc2VlZCA9IDQyLCBzZWdtZW50LnNpemU9MC4xLCBzaG93LmxlZ2VuZCA9IEZBTFNFKSArDQogIHhsYWIoRDFfdGV4dCkgKyANCiAgeWxhYihEMl90ZXh0KSArDQogIGdndGl0bGUoIlBDQSBtZXRhdHJhbnNjcmlwdG9taWNzICBcblIuIGVuY2xlbnNpcyIpICsNCiAgbGFicyhjYXB0aW9uID0gIHBhc3RlMCgiUEVSTUFOT1ZBIG9uIGNvbmRpdGlvbjogcC12YWx1ZTogIiwgcHYpKSArIA0KICB0aGVtZShwYW5lbC5iYWNrZ3JvdW5kPSBlbGVtZW50X3JlY3QoZmlsbD0iI2Y0ZjRmNCIpLA0KICAgICAgICBwbG90LmNhcHRpb24gPSBlbGVtZW50X3RleHQoc2l6ZT03KSkgLT4gcDENCiANCg0KZ2dwbG90KHBjYV9kYXRhKSArDQogIGdlb21fcG9pbnQoYWVzKHg9RGltLjEsIHk9RGltLjMsIGNvbG91cj1jb2xvciksIHNpemU9MykgKw0KICBzY2FsZV9jb2xvdXJfaWRlbnRpdHkoIkNvbmRpdGlvbiIsIGxhYmVscz1jKCJXaW50ZXIgTmlnaHQiLCAiU3VtbWVyIERheSIpLCBndWlkZSA9ICJsZWdlbmQiKSArDQogIGdncmVwZWw6Omdlb21fdGV4dF9yZXBlbChhZXMoeD1EaW0uMSwgeT1EaW0uMywgY29sb3VyPWNvbG9yLCBsYWJlbD1zYW1wbGUpLCANCiAgICAgICAgICAgICAgICAgICAgICAgICAgIG51ZGdlX3kgPSAtMiwgIHNlZWQgPSA0Miwgc2VnbWVudC5zaXplPTAuMSwgc2hvdy5sZWdlbmQgPSBGQUxTRSkgKw0KICB4bGFiKEQxX3RleHQpICsgDQogIHlsYWIoRDNfdGV4dCkgKw0KICBnZ3RpdGxlKCJQQ0EgbWV0YXRyYW5zY3JpcHRvbWljcyAgXG5SLiBlbmNsZW5zaXMiKSArDQogIGxhYnMoY2FwdGlvbiA9ICBwYXN0ZTAoIlBFUk1BTk9WQSBvbiBjb25kaXRpb246IHAtdmFsdWU6ICIsIHB2KSkgKyANCiAgdGhlbWUocGFuZWwuYmFja2dyb3VuZD0gZWxlbWVudF9yZWN0KGZpbGw9IiNmNGY0ZjQiKSwNCiAgICAgICAgcGxvdC5jYXB0aW9uID0gZWxlbWVudF90ZXh0KHNpemU9NykpIC0+IHAyDQoNCmdncGxvdChwY2FfZGF0YSkgKw0KICBnZW9tX3BvaW50KGFlcyh4PURpbS4yLCB5PURpbS4zLCBjb2xvdXI9Y29sb3IpLCBzaXplPTMpICsNCiAgc2NhbGVfY29sb3VyX2lkZW50aXR5KCJDb25kaXRpb24iLCBsYWJlbHM9YygiV2ludGVyIE5pZ2h0IiwgIlN1bW1lciBEYXkiKSwgZ3VpZGUgPSAibGVnZW5kIikgKw0KICBnZ3JlcGVsOjpnZW9tX3RleHRfcmVwZWwoYWVzKHg9RGltLjIsIHk9RGltLjMsIGNvbG91cj1jb2xvciwgbGFiZWw9c2FtcGxlKSwgDQogICAgICAgICAgICAgICAgICAgICAgICAgICBudWRnZV95ID0gLTIsICBzZWVkID0gNDIsIHNlZ21lbnQuc2l6ZT0wLjEsIHNob3cubGVnZW5kID0gRkFMU0UpICsNCiAgeGxhYihEMl90ZXh0KSArIA0KICB5bGFiKEQzX3RleHQpICsNCiAgZ2d0aXRsZSgiUENBIG1ldGF0cmFuc2NyaXB0b21pY3MgIFxuUi4gZW5jbGVuc2lzIikgKw0KICBsYWJzKGNhcHRpb24gPSAgcGFzdGUwKCJQRVJNQU5PVkEgb24gY29uZGl0aW9uOiBwLXZhbHVlOiAiLCBwdikpICsgDQogIHRoZW1lKHBhbmVsLmJhY2tncm91bmQ9IGVsZW1lbnRfcmVjdChmaWxsPSIjZjRmNGY0IiksDQogICAgICAgIHBsb3QuY2FwdGlvbiA9IGVsZW1lbnRfdGV4dChzaXplPTcpKSAtPiBwMw0KDQpwbG90Z3JpZF9QQ0EgPC0gZ2dhcnJhbmdlKHAxLCBwMiwgcDMsIGxhYmVscyA9IGMoIkEiLCAiQiIsICJDIiksIGNvbW1vbi5sZWdlbmQ9VFJVRSwgbGVnZW5kPSJib3R0b20iLCBucm93PTEpIA0KDQpwcmludChwbG90Z3JpZF9QQ0EpDQoNCmdnc2F2ZShwbG90ID0gcGxvdGdyaWRfUENBLCBmaWxlbmFtZSA9ICIuLi9maWd1cmVzL1BDQV9tZXRhdHJhbnNjcmlwdG9taWNzXzNfY29tcG9zYW50ZXNfcmhvZW4udGlmZiIsIGRwaSA9IDMwMCwgd2lkdGggPSAxNSwgaGVpZ2h0ID0gNSwgYmc9IndoaXRlIikNCg0KZ2dzYXZlKHBsb3Q9cDEsIGZpbGVuYW1lID0gIi4uL2ZpZ3VyZXMvUENBX21ldGF0cmFuc2NyaXB0b21pY3NfcmhvZW4udGlmZiIsIGRwaSA9IDMwMCwgd2lkdGggPSA2LCBoZWlnaHQgPSA1LCBiZz0id2hpdGUiKQ0KYGBgDQoNCmBgYHtyIDNEX1BDQV9jbHJfcmhvZW4sIHdlYmdsPVRSVUUsIG1lc3NhZ2U9RkFMU0UsIHdhcm5pbmc9RkFMU0UsIGZpZy5oZWlnaHQ9NywgZmlnLndpZHRoPTd9DQoNCnJvd19jb29yZCA8LSByZXNfcGNhJGluZCRjb29yZA0KDQppbnZpc2libGUocmdsOjpvcGVuM2QoKSkNCg0KI3JnbDo6YmczZCgibGlnaHRncmF5IikNCnJnbDo6cGxvdDNkKHg9cm93X2Nvb3JkW2MoIldOX1RGXzEiLCAiV05fVEZfMiIsICJXTl9URl8zIiksIDFdLCANCiAgICAgICAgICAgIHk9cm93X2Nvb3JkW2MoIldOX1RGXzEiLCAiV05fVEZfMiIsICJXTl9URl8zIiksIDJdLCANCiAgICAgICAgICAgIHo9cm93X2Nvb3JkW2MoIldOX1RGXzEiLCAiV05fVEZfMiIsICJXTl9URl8zIiksIDNdLA0KICAgICAgICAgICAgeGxhYiA9IHBhc3RlMChjb2xuYW1lcyhyb3dfY29vcmQpWzFdICwgIiAoIiAsIHN1YnN0cihhcy5jaGFyYWN0ZXIocmVzX3BjYSRlaWdbLDJdWzFdKSwgMSwgNSkgLCAiJSkiKSwNCiAgICAgICAgICAgIHlsYWIgPSBwYXN0ZTAoY29sbmFtZXMocm93X2Nvb3JkKVsyXSAsICIgKCIgLCBzdWJzdHIoYXMuY2hhcmFjdGVyKHJlc19wY2EkZWlnWywyXVsyXSksIDEsIDUpICwgIiUpIiksDQogICAgICAgICAgICB6bGFiID0gcGFzdGUwKGNvbG5hbWVzKHJvd19jb29yZClbM10gLCAiICgiICwgc3Vic3RyKGFzLmNoYXJhY3RlcihyZXNfcGNhJGVpZ1ssMl1bM10pLCAxLCA1KSAsICIlKSIpLA0KICAgICAgICAgICAgeGxpbSA9IGMobWluKHJvd19jb29yZFssIDFdKSwgbWF4KHJvd19jb29yZFssIDFdKSsxMCksIA0KICAgICAgICAgICAgeWxpbSA9IGMobWluKHJvd19jb29yZFssIDJdKSwgbWF4KHJvd19jb29yZFssIDJdKSsxMCksIA0KICAgICAgICAgICAgemxpbSA9IGMobWluKHJvd19jb29yZFssIDNdKSwgbWF4KHJvd19jb29yZFssIDNdKSsxMCksDQogICAgICAgICAgICBjb2wgPSAiZGFya2N5YW4iLA0KICAgICAgICAgICAgc2l6ZT0xMCwNCiAgICAgICAgICAgIG1haW4gPSAiUENBIFIuIGVuY2xlbnNpcyIpDQoNCnJnbDo6dGV4dDNkKHg9cm93X2Nvb3JkW2MoIldOX1RGXzEiLCAiV05fVEZfMiIsICJXTl9URl8zIiksIDFdKzMsIA0KICAgICAgICAgICAgeT1yb3dfY29vcmRbYygiV05fVEZfMSIsICJXTl9URl8yIiwgIldOX1RGXzMiKSwgMl0rMywgDQogICAgICAgICAgICB6PXJvd19jb29yZFtjKCJXTl9URl8xIiwgIldOX1RGXzIiLCAiV05fVEZfMyIpLCAzXSszLA0KICAgICAgICAgICAgdGV4dD1jKCJXTl9URl8xIiwgIldOX1RGXzIiLCAiV05fVEZfMyIpLA0KICAgICAgICAgICAgY29sID0gImRhcmtjeWFuIiwNCiAgICAgICAgICAgIHNpemU9MTApDQoNCg0KcmdsOjpwb2ludHMzZCh4PXJvd19jb29yZFtjKCJTRF9URl8xIiwgIlNEX1RGXzIiLCAiU0RfVEZfMyIpLCAxXSwgDQogICAgICAgICAgICAgIHk9cm93X2Nvb3JkW2MoIlNEX1RGXzEiLCAiU0RfVEZfMiIsICJTRF9URl8zIiksIDJdLCANCiAgICAgICAgICAgICAgej1yb3dfY29vcmRbYygiU0RfVEZfMSIsICJTRF9URl8yIiwgIlNEX1RGXzMiKSwgM10sDQogICAgICAgICAgICAgIGNvbCA9ICJkYXJrb3JhbmdlIiwNCiAgICAgICAgICAgICAgc2l6ZT0xMCkNCg0KcmdsOjp0ZXh0M2QoeD1yb3dfY29vcmRbYygiU0RfVEZfMSIsICJTRF9URl8yIiwgIlNEX1RGXzMiKSwgMV0rMywgDQogICAgICAgICAgICB5PXJvd19jb29yZFtjKCJTRF9URl8xIiwgIlNEX1RGXzIiLCAiU0RfVEZfMyIpLCAyXSszLCANCiAgICAgICAgICAgIHo9cm93X2Nvb3JkW2MoIlNEX1RGXzEiLCAiU0RfVEZfMiIsICJTRF9URl8zIiksIDNdKzMsDQogICAgICAgICAgICB0ZXh0PWMoIlNEX1RGXzEiLCAiU0RfVEZfMiIsICJTRF9URl8zIiksDQogICAgICAgICAgICBjb2wgPSAiZGFya29yYW5nZSIsDQogICAgICAgICAgICBzaXplPTEwKQ0KDQoNCiMjIEFjdGl2YXRlIGZvciBvciBnaWYgZ2VuZXJhdGlvbg0KI3BhcjNkKHdpbmRvd1JlY3QgPSBjKDIwLCAzMCwgODAwLCA4MDApKQ0KDQojcmdsOjptb3ZpZTNkKA0KIyAgbW92aWU9IjNEQW5pbWF0ZWRfUENBX3Job2VuIiwgDQojICBzcGluM2QoIGF4aXMgPSBjKDAsIDAsIDEpLCBycG0gPSAzKSwNCiMgIGR1cmF0aW9uID0gMjAsIA0KIyAgZGlyID0gIi4uL2ZpZ3VyZXMiLA0KIyAgdHlwZSA9ICJnaWYiLCANCiMgIGNsZWFuID0gVFJVRSwgd2Vic2hvdD1GQUxTRSwgZnBzPTEwKQ0KYGBgDQoNCg0KIyMjIFNhbXBsZXMgaGllcmFyY2hpY2FsIGNsdXN0ZXJpbmcgYnkgc3BlY2llcyB7LnRhYnNldH0NCiMjIyMgKkRpb3N6ZWdpYSBodW5nYXJpY2EqDQoNCmBgYHtyIGRpc3RhbmNlX21hdHJpY2VzX2Rpb2h1fQ0KIyMjIyBTYW1wbGUgZGlzdGFuY2VzICMjIyMNCm1lc3NhZ2UoIkNvbXB1dGluZyBpbnRlci1zYW1wbGUgZGlzdGFuY2VzIikNCg0KIyMgUGVhcnNvbg0KZGlzdF9wZWFyc29uX2Rpb2h1IDwtIGFzLmRpc3QoMSAtIGNvcihkaW9odV9jbHIsIHVzZSA9ICJldmVyeXRoaW5nIiwgDQogICAgICAgICAgICAgICAgICAgICAgICAgICAgICAgIG1ldGhvZCA9ICJwZWFyc29uIikpDQpgYGANCg0KYGBge3Igc2FtcGxlX3RyZWVfZGlvaHV9DQptZXNzYWdlKCJTYW1wbGUgY2x1c3RlcmluZyIpDQoNCnRyZWVfcGVhcnNvbl9kaW9odSA8LSBoY2x1c3QoZGlzdF9wZWFyc29uX2Rpb2h1LCBtZXRob2QgPSAiY29tcGxldGUiKQ0KDQpgYGANCg0KYGBge3IgcGVhcnNvbl90cmVlX2Rpb2h1LCBmaWcuaGVpZ2h0PTd9DQpwYXIoYmcgPSAid2hpdGUiLCBtZnJvdz1jKDEsIDEpKQ0KcGxvdENvbG9yZWRDbHVzdGVycyh0cmVlX3BlYXJzb25fZGlvaHUsIGxhYnMgPSByb3cubmFtZXMobWV0YWRhdGFfdGFibGUpLA0KICAgICAgICAgICAgICAgICAgICB5bGFiID0gTkEsIHhsYWIgPSBOQSwgY2V4ID0gMSwgbGFzID0gMSwNCiAgICAgICAgICAgICAgICAgICAgY29scyA9IG1ldGFkYXRhX3RhYmxlJGNvbG9yLCBjb2wgPSAiYmxhY2siLA0KICAgICAgICAgICAgICAgICAgICBtYWluID0gIlNhbXBsZXMgUGVhcnNvbiBkaXN0YW5jZSBoaWVyYXJjaGljYWwgY2x1c3RlcmluZywNCmNvbXBsZXRlIGxpbmthZ2UsIEQuIGh1bmdhcmljYSBnZW5lcy4iKQ0KYGBgDQoNCk5hdHVyYWwgc2VwYXJhdGlvbiBvZiBzYW1wbGVzIGFjY29yZGluZyB0byB0aGVpciBpbmN1YmF0aW9uIGNvbmRpdGlvbiBmb3IgKkQuIGh1bmdhcmljYSouDQoNCiMjIyMgKlBzZXVkb21vbmFzIGdyYW1pbmlzKg0KDQpgYGB7ciBkaXN0YW5jZV9tYXRyaWNlc19wc2Vncn0NCiMjIyMgU2FtcGxlIGRpc3RhbmNlcyAjIyMjDQptZXNzYWdlKCJDb21wdXRpbmcgaW50ZXItc2FtcGxlIGRpc3RhbmNlcyIpDQoNCiMjIFBlYXJzb24NCmRpc3RfcGVhcnNvbl9wc2VnciA8LSBhcy5kaXN0KDEgLSBjb3IocHNlZ3JfY2xyLCB1c2UgPSAiZXZlcnl0aGluZyIsIA0KICAgICAgICAgICAgICAgICAgICAgICAgICAgICAgICBtZXRob2QgPSAicGVhcnNvbiIpKQ0KYGBgDQoNCmBgYHtyIHNhbXBsZV90cmVlX3BzZWdyfQ0KbWVzc2FnZSgiU2FtcGxlIGNsdXN0ZXJpbmciKQ0KDQp0cmVlX3BlYXJzb25fcHNlZ3IgPC0gaGNsdXN0KGRpc3RfcGVhcnNvbl9wc2VnciwgbWV0aG9kID0gImNvbXBsZXRlIikNCmBgYA0KDQoNCmBgYHtyIHBlYXJzb25fdHJlZV9wc2VnciwgZmlnLmhlaWdodD03fQ0KcGFyKGJnID0gIndoaXRlIiwgbWZyb3c9YygxLCAxKSkNCnBsb3RDb2xvcmVkQ2x1c3RlcnModHJlZV9wZWFyc29uX3BzZWdyLCBsYWJzID0gcm93Lm5hbWVzKG1ldGFkYXRhX3RhYmxlKSwNCiAgICAgICAgICAgICAgICAgICAgeWxhYiA9IE5BLCB4bGFiID0gTkEsIGNleCA9IDEsIGxhcyA9IDEsDQogICAgICAgICAgICAgICAgICAgIGNvbHMgPSBtZXRhZGF0YV90YWJsZSRjb2xvciwgY29sID0gImJsYWNrIiwNCiAgICAgICAgICAgICAgICAgICAgbWFpbiA9ICJTYW1wbGVzIFBlYXJzb24gZGlzdGFuY2UgaGllcmFyY2hpY2FsIGNsdXN0ZXJpbmcsDQpjb21wbGV0ZSBsaW5rYWdlLCBQLiBncmFtaW5pcyBnZW5lcy4iKQ0KYGBgDQoNCkZvciAqUC4gZ3JhbWluaXMqLCB0aGUgY2x1c3RlcmluZyBkb2VzIG5vdCBkaXN0aW5ndWlzaCBiZXR3ZWVuIHRoZSB0d28gaW5jdWJhdGlvbiBjb25kaXRpb25zLCBwcm9iYWJseSBiZWNhdXNlIG9mIHRoZSB2ZXJ5IGxvdyBhbW91bnRzIG9mIGNvdW50cyBhdHRyaWJ1dGVkIHRvIHRoaXMgc3BlY2llcy4NCg0KDQojIyMjICpQc2V1ZG9tb25hcyBzeXJpbmdhZSoNCg0KYGBge3IgZGlzdGFuY2VfbWF0cmljZXNfcHNlc3l9DQojIyMjIFNhbXBsZSBkaXN0YW5jZXMgIyMjIw0KbWVzc2FnZSgiQ29tcHV0aW5nIGludGVyLXNhbXBsZSBkaXN0YW5jZXMiKQ0KDQojIyBQZWFyc29uDQpkaXN0X3BlYXJzb25fcHNlc3kgPC0gYXMuZGlzdCgxIC0gY29yKHBzZXN5X2NsciwgdXNlID0gImV2ZXJ5dGhpbmciLCANCiAgICAgICAgICAgICAgICAgICAgICAgICAgICAgICAgbWV0aG9kID0gInBlYXJzb24iKSkNCmBgYA0KDQpgYGB7ciBzYW1wbGVfdHJlZV9wc2VzeX0NCm1lc3NhZ2UoIlNhbXBsZSBjbHVzdGVyaW5nIikNCg0KdHJlZV9wZWFyc29uX3BzZXN5IDwtIGhjbHVzdChkaXN0X3BlYXJzb25fcHNlc3ksIG1ldGhvZCA9ICJjb21wbGV0ZSIpDQpgYGANCg0KYGBge3IgcGVhcnNvbl90cmVlX3BzZXN5LCBmaWcuaGVpZ2h0PTd9DQpwYXIoYmcgPSAid2hpdGUiLCBtZnJvdz1jKDEsIDEpKQ0KcGxvdENvbG9yZWRDbHVzdGVycyh0cmVlX3BlYXJzb25fcHNlc3ksIGxhYnMgPSByb3cubmFtZXMobWV0YWRhdGFfdGFibGUpLA0KICAgICAgICAgICAgICAgICAgICB5bGFiID0gTkEsIHhsYWIgPSBOQSwgY2V4ID0gMSwgbGFzID0gMSwNCiAgICAgICAgICAgICAgICAgICAgY29scyA9IG1ldGFkYXRhX3RhYmxlJGNvbG9yLCBjb2wgPSAiYmxhY2siLA0KICAgICAgICAgICAgICAgICAgICBtYWluID0gIlNhbXBsZXMgUGVhcnNvbiBkaXN0YW5jZSBoaWVyYXJjaGljYWwgY2x1c3RlcmluZywNCmNvbXBsZXRlIGxpbmthZ2UsIFAuIHN5cmluZ2FlIGdlbmVzLiIpDQpgYGANCg0KU2FtcGxlcyBhcmUgc2VwYXJhdGVkIGFjY29yZGluZyB0byB0aGVpciBleHBlcmltZW50YWwgY29uZGl0aW9uLCB3aGljaCBpcyBlbmNvdXJhZ2luZyBmb3IgZnVydGhlciBhbmFseXNpcyBvZiAqUC4gc3lyaW5nYWUqLg0KDQoNCg0KIyMjIyAqUmhvZG9jb2NjdXMgZW5jbGVuc2lzKg0KDQpgYGB7ciBkaXN0YW5jZV9tYXRyaWNlc19yaG9lbn0NCiMjIyMgU2FtcGxlIGRpc3RhbmNlcyAjIyMjDQptZXNzYWdlKCJDb21wdXRpbmcgaW50ZXItc2FtcGxlIGRpc3RhbmNlcyIpDQoNCiMjIFBlYXJzb24NCmRpc3RfcGVhcnNvbl9yaG9lbiA8LSBhcy5kaXN0KDEgLSBjb3IocmhvZW5fY2xyLCB1c2UgPSAiZXZlcnl0aGluZyIsIA0KICAgICAgICAgICAgICAgICAgICAgICAgICAgICAgICBtZXRob2QgPSAicGVhcnNvbiIpKQ0KYGBgDQoNCmBgYHtyIHNhbXBsZV90cmVlX3Job2VufQ0KbWVzc2FnZSgiU2FtcGxlIGNsdXN0ZXJpbmciKQ0KDQp0cmVlX3BlYXJzb25fcmhvZW4gPC0gaGNsdXN0KGRpc3RfcGVhcnNvbl9yaG9lbiwgbWV0aG9kID0gImNvbXBsZXRlIikNCmBgYA0KDQpgYGB7ciBwZWFyc29uX3RyZWVfcmhvZW4sIGZpZy5oZWlnaHQ9N30NCnBhcihiZyA9ICJ3aGl0ZSIsIG1mcm93PWMoMSwgMSkpDQpwbG90Q29sb3JlZENsdXN0ZXJzKHRyZWVfcGVhcnNvbl9yaG9lbiwgbGFicyA9IHJvdy5uYW1lcyhtZXRhZGF0YV90YWJsZSksDQogICAgICAgICAgICAgICAgICAgIHlsYWIgPSBOQSwgeGxhYiA9IE5BLCBjZXggPSAxLCBsYXMgPSAxLA0KICAgICAgICAgICAgICAgICAgICBjb2xzID0gbWV0YWRhdGFfdGFibGUkY29sb3IsIGNvbCA9ICJibGFjayIsDQogICAgICAgICAgICAgICAgICAgIG1haW4gPSAiU2FtcGxlcyBQZWFyc29uIGRpc3RhbmNlIGhpZXJhcmNoaWNhbCBjbHVzdGVyaW5nLA0KY29tcGxldGUgbGlua2FnZSwgUi4gZW5jbGVuc2lzIGdlbmVzLiIpDQpgYGANCg0KQWdhaW4sIHNhbXBsZXMgYXJlIG5hdHVyYWxseSBzZXBhcmF0ZWQgYWNjb3JkaW5nIHRvIHRoZWlyIGluY3ViYXRpb24gY29uZGl0aW9uIGZvciAqUi4gZW5jbGVuc2lzKi4NCg0KDQpgYGB7ciBnZW5lX3RyZWVfZGlvaHUsIGluY2x1ZGU9RkFMU0UsIGVjaG89RkFMU0UsIGV2YWw9RkFMU0V9DQoNCiMgR2VuZSB0cmVlIHdpdGggUGVhcnNvbiBjb3JyZWxhdGlvbiAjIyMjDQptZXNzYWdlKCJEcmF3aW5nIGdlbmUgdHJlZSIpDQoNCiMgUGVhcnNvbiBkaXN0YW5jZSBnZW5lDQpkaXN0X2dlbmVfcGVhcnNvbl9kaW9odSA8LSBhcy5kaXN0KDEgLSBjb3IodChkaW9odV9jbHIpLA0KICAgICAgICAgICAgICAgICAgICAgICAgICAgICAgICB1c2UgPSAiZXZlcnl0aGluZyIsIG1ldGhvZCA9ICJwZWFyc29uIikpDQoNCiMgdHJlZQ0KdHJlZV9nZW5lX3BlYXJzb25fZGlvaHUgPC0gaGNsdXN0KGRpc3RfZ2VuZV9wZWFyc29uX2Rpb2h1LCBtZXRob2QgPSAiY29tcGxldGUiKQ0KYGBgDQoNCg0KYGBge3IgY3V0X3RyZWVfZGlvaHUsIGluY2x1ZGU9RkFMU0UsIGVjaG89RkFMU0UsIGV2YWw9RkFMU0V9DQpjbHVzdGVyc19nZW5lX3BlYXJzb25fZGlvaHUgPC0gY3V0cmVlKHRyZWVfZ2VuZV9wZWFyc29uX2Rpb2h1LCBoPTEuOTcpDQpgYGANCg0KYGBge3Igc2F2ZV9nZW5lX2NsdXN0ZXJzX2Rpb2h1LCBpbmNsdWRlPUZBTFNFLCBlY2hvPUZBTFNFLCBldmFsPUZBTFNFfQ0KIyBTYXZpbmcgY2x1c3RlcmluZyByZXN1bHRzIGluIGdlbmUgZGF0YWZyYW1lIA0KZ2VuZV9zdGF0X25vcm1fZGlvaHUgPC0gZ2VuZV9zdGF0X25vcm1bZGlvaHVfZ2VuZWlkLF0NCmdlbmVfc3RhdF9ub3JtX2Rpb2h1JGNsdXN0ZXIgPC0gY2x1c3RlcnNfZ2VuZV9wZWFyc29uX2Rpb2h1DQoNCg0KIyBXZSBhZGQgdGhlIGNsdXN0ZXIgaW5mb3JtYXRpb24gaW4gdGhlIG1vc3RfZXhwcmVzc2VkX2dlbmVzX3N0YXQNCm15X2NvbG9ycyA9IGMoIm9yYW5nZSIsICJibHVlIiwgImRhcmtyZWQiLCAic2t5Ymx1ZSIsICJkYXJrZ3JlZW4iLCAicGluayIsICJncmVlbiIsICJyZWQiKQ0KDQpgYGANCg0KDQpgYGB7ciBnZW5lX2NsdXN0ZXJpbmdfcHNlZ3IsIGluY2x1ZGU9RkFMU0UsIGVjaG89RkFMU0UsIGV2YWw9RkFMU0V9DQojIyMjIEdlbmUgdHJlZSB3aXRoIFBlYXJzb24gY29ycmVsYXRpb24gIyMjIw0KbWVzc2FnZSgiRHJhd2luZyBnZW5lIHRyZWUiKQ0KDQojIFBlYXJzb24gZGlzdGFuY2UgZ2VuZQ0KZGlzdF9nZW5lX3BlYXJzb25fcHNlZ3IgPC0gYXMuZGlzdCgxIC0gY29yKHQocHNlZ3JfY2xyKSwgDQogICAgICAgICAgICAgICAgICAgICAgICAgICAgICAgIHVzZSA9ICJldmVyeXRoaW5nIiwgbWV0aG9kID0gInBlYXJzb24iKSkNCg0KIyB0cmVlDQp0cmVlX2dlbmVfcGVhcnNvbl9wc2VnciA8LSBoY2x1c3QoZGlzdF9nZW5lX3BlYXJzb25fcHNlZ3IsIG1ldGhvZCA9ICJjb21wbGV0ZSIpDQpgYGANCg0KYGBge3IgY3V0X3RyZWVfcHNlZ3IsIGluY2x1ZGU9RkFMU0UsIGVjaG89RkFMU0UsIGV2YWw9RkFMU0V9DQpjbHVzdGVyc19nZW5lX3BlYXJzb25fcHNlZ3IgPC0gY3V0cmVlKHRyZWVfZ2VuZV9wZWFyc29uX3BzZWdyLCBoPTEuOTIpDQpgYGANCg0KYGBge3Igc2F2ZV9nZW5lX2NsdXN0ZXJzX3BzZWdyLCBpbmNsdWRlPUZBTFNFLCBlY2hvPUZBTFNFLCBldmFsPUZBTFNFfQ0KIyBTYXZpbmcgY2x1c3RlcmluZyByZXN1bHRzIGluIGdlbmUgZGF0YWZyYW1lIA0KZ2VuZV9zdGF0X25vcm1fcHNlZ3IgPC0gZ2VuZV9zdGF0X25vcm1bcHNlZ3JfZ2VuZWlkLF0NCmdlbmVfc3RhdF9ub3JtX3BzZWdyJGNsdXN0ZXIgPC0gY2x1c3RlcnNfZ2VuZV9wZWFyc29uX3BzZWdyDQoNCg0KIyBXZSBhZGQgdGhlIGNsdXN0ZXIgaW5mb3JtYXRpb24gaW4gdGhlIG1vc3RfZXhwcmVzc2VkX2dlbmVzX3N0YXQNCm15X2NvbG9ycyA9IGMoIm9yYW5nZSIsICJibHVlIiwgImRhcmtyZWQiLCAic2t5Ymx1ZSIsICJkYXJrZ3JlZW4iLCAicGluayIsICJncmVlbiIsICJyZWQiKQ0KDQpgYGANCg0KDQpgYGB7ciBnZW5lX2NsdXN0ZXJpbmdfcHNlc3ksIGluY2x1ZGU9RkFMU0UsIGVjaG89RkFMU0UsIGV2YWw9RkFMU0V9DQojIyMjIEdlbmUgdHJlZSB3aXRoIFBlYXJzb24gY29ycmVsYXRpb24gIyMjIw0KbWVzc2FnZSgiRHJhd2luZyBnZW5lIHRyZWUiKQ0KDQojIFBlYXJzb24gZGlzdGFuY2UgZ2VuZQ0KZGlzdF9nZW5lX3BlYXJzb25fcHNlc3kgPC0gYXMuZGlzdCgxIC0gY29yKHQocHNlc3lfY2xyKSwgDQogICAgICAgICAgICAgICAgICAgICAgICAgICAgICAgIHVzZSA9ICJldmVyeXRoaW5nIiwgbWV0aG9kID0gInBlYXJzb24iKSkNCg0KIyB0cmVlDQp0cmVlX2dlbmVfcGVhcnNvbl9wc2VzeSA8LSBoY2x1c3QoZGlzdF9nZW5lX3BlYXJzb25fcHNlc3ksIG1ldGhvZCA9ICJjb21wbGV0ZSIpDQpgYGANCg0KYGBge3IgY3V0X3RyZWVfcHNlc3ksIGluY2x1ZGU9RkFMU0UsIGVjaG89RkFMU0UsIGV2YWw9RkFMU0V9DQpjbHVzdGVyc19nZW5lX3BlYXJzb25fcHNlc3kgPC0gY3V0cmVlKHRyZWVfZ2VuZV9wZWFyc29uX3BzZXN5LCBoPTEuOTEpDQpgYGANCg0KYGBge3Igc2F2ZV9nZW5lX2NsdXN0ZXJzX3BzZXN5LCBpbmNsdWRlPUZBTFNFLCBlY2hvPUZBTFNFLCBldmFsPUZBTFNFfQ0KIyBTYXZpbmcgY2x1c3RlcmluZyByZXN1bHRzIGluIGdlbmUgZGF0YWZyYW1lIA0KZ2VuZV9zdGF0X25vcm1fcHNlc3kgPC0gZ2VuZV9zdGF0X25vcm1bcHNlc3lfZ2VuZWlkLF0NCmdlbmVfc3RhdF9ub3JtX3BzZXN5JGNsdXN0ZXIgPC0gY2x1c3RlcnNfZ2VuZV9wZWFyc29uX3BzZXN5DQoNCg0KIyBXZSBhZGQgdGhlIGNsdXN0ZXIgaW5mb3JtYXRpb24gaW4gdGhlIG1vc3RfZXhwcmVzc2VkX2dlbmVzX3N0YXQNCm15X2NvbG9ycyA9IGMoIm9yYW5nZSIsICJibHVlIiwgImRhcmtyZWQiLCAic2t5Ymx1ZSIsICJkYXJrZ3JlZW4iLCAicGluayIsICJncmVlbiIsICJyZWQiKQ0KDQpgYGANCg0KDQpgYGB7ciBnZW5lX2NsdXN0ZXJpbmdfcmhvZW4sIGluY2x1ZGU9RkFMU0UsIGVjaG89RkFMU0UsIGV2YWw9RkFMU0V9DQojIyMjIEdlbmUgdHJlZSB3aXRoIFBlYXJzb24gY29ycmVsYXRpb24gIyMjIw0KbWVzc2FnZSgiRHJhd2luZyBnZW5lIHRyZWUiKQ0KDQojIFBlYXJzb24gZGlzdGFuY2UgZ2VuZQ0KZGlzdF9nZW5lX3BlYXJzb25fcmhvZW4gPC0gYXMuZGlzdCgxIC0gY29yKHQocmhvZW5fY2xyKSwgDQogICAgICAgICAgICAgICAgICAgICAgICAgICAgICAgIHVzZSA9ICJldmVyeXRoaW5nIiwgbWV0aG9kID0gInBlYXJzb24iKSkNCg0KIyB0cmVlDQp0cmVlX2dlbmVfcGVhcnNvbl9yaG9lbiA8LSBoY2x1c3QoZGlzdF9nZW5lX3BlYXJzb25fcmhvZW4sIG1ldGhvZCA9ICJjb21wbGV0ZSIpDQpgYGANCg0KYGBge3IgY3V0X3RyZWVfcmhvZW4sIGluY2x1ZGU9RkFMU0UsIGVjaG89RkFMU0UsIGV2YWw9RkFMU0V9DQpjbHVzdGVyc19nZW5lX3BlYXJzb25fcmhvZW4gPC0gY3V0cmVlKHRyZWVfZ2VuZV9wZWFyc29uX3Job2VuLCBoPTEuOCkNCg0KYGBgDQoNCmBgYHtyIHNhdmVfZ2VuZV9jbHVzdGVyc19yaG9lbiwgaW5jbHVkZT1GQUxTRSwgZWNobz1GQUxTRSwgZXZhbD1GQUxTRX0NCiMgU2F2aW5nIGNsdXN0ZXJpbmcgcmVzdWx0cyBpbiBnZW5lIGRhdGFmcmFtZSANCmdlbmVfc3RhdF9ub3JtX3Job2VuIDwtIGdlbmVfc3RhdF9ub3JtW3Job2VuX2dlbmVpZCxdDQpnZW5lX3N0YXRfbm9ybV9yaG9lbiRjbHVzdGVyIDwtIGNsdXN0ZXJzX2dlbmVfcGVhcnNvbl9yaG9lbg0KDQoNCiMgV2UgYWRkIHRoZSBjbHVzdGVyIGluZm9ybWF0aW9uIGluIHRoZSBtb3N0X2V4cHJlc3NlZF9nZW5lc19zdGF0DQpteV9jb2xvcnMgPSBjKCJvcmFuZ2UiLCAiYmx1ZSIsICJkYXJrcmVkIiwgInNreWJsdWUiLCAiZGFya2dyZWVuIiwgInBpbmsiLCAiZ3JlZW4iLCAicmVkIikNCg0KDQpgYGANCg0KDQoNCmBgYHtyIGhlYXRtYXBfZGlvaHUsIGZpZy5oZWlnaHQ9NywgZmlnLndpZHRoPTcsIGluY2x1ZGU9RkFMU0UsIGVjaG89RkFMU0UsIGV2YWw9RkFMU0V9DQoNCiMjIyBCaWNsdXN0ZXJpbmcgey50YWJzZXR9DQojIyMjICpEaW9zemVnaWEgaHVuZ2FyaWNhKg0KDQojIyMjIEhlYXRtYXAgd2l0aCBiaWNsdXN0ZXJpbmcgIyMjIw0KbWVzc2FnZSgiaGVhdG1hcCB3aXRoIGJpY2x1c3RlcmluZyIpDQoNCmFubm90X2NsdXN0X2Rpb2h1ID0gZGF0YS5mcmFtZShhcy5mYWN0b3IoZ2VuZV9zdGF0X25vcm1fZGlvaHUkY2x1c3RlcikpDQpjb2xuYW1lcyhhbm5vdF9jbHVzdF9kaW9odSkgPC0gImNsdXN0ZXIiDQpyb3duYW1lcyhhbm5vdF9jbHVzdF9kaW9odSkgPC0gcm93bmFtZXMoZGlvaHVfY2xyKQ0KDQpwaGVhdG1hcChkaW9odV9jbHIsDQogICAgICAgICBjbHVzdGVyX3Jvd3MgPSBUUlVFLA0KICAgICAgICAgY2x1c3Rlcl9jb2xzID0gVFJVRSwNCiAgICAgICAgIGNsdXN0ZXJpbmdfZGlzdGFuY2Vfcm93cyA9IGRpc3RfZ2VuZV9wZWFyc29uX2Rpb2h1LA0KICAgICAgICAgY2x1c3RlcmluZ19kaXN0YW5jZV9jb2xzID0gZGlzdF9wZWFyc29uX2Rpb2h1LA0KICAgICAgICAgYm9yZGVyX2NvbG9yID0gTkEsDQogICAgICAgICBzaG93X3Jvd25hbWVzID0gRkFMU0UsDQogICAgICAgICBhbm5vdGF0aW9uX3JvdyA9IGFubm90X2NsdXN0X2Rpb2h1LA0KICAgICAgICAgYW5ub3RhdGlvbl9uYW1lc19yb3cgPSBGQUxTRSwNCiAgICAgICAgIGN1dHJlZV9yb3dzID0gOCwNCiAgICAgICAgIGN1dHJlZV9jb2xzID0gMiwNCiAgICAgICAgIHNjYWxlID0gInJvdyIsDQogICAgICAgICB1c2VfcmFzdGVyID0gVFJVRSwNCiAgICAgICAgIGFuZ2xlX2NvbCA9ICIwIiwNCiAgICAgICAgIG1haW4gPSAiQmljbHVzdGVyaW5nIG9mIEQuIGh1bmdhcmljYSBnZW5lcyAoUGVhcnNvbiBkaXN0YW5jZSkgDQphbmQgc2FtcGxlcyAoUGVhcnNvbiBkaXN0YW5jZSkiKQ0KYGBgDQoNCg0KDQpgYGB7ciBoZWF0bWFwX3BzZWdyLCBmaWcuaGVpZ2h0PTcsIGZpZy53aWR0aD03LCBpbmNsdWRlPUZBTFNFLCBlY2hvPUZBTFNFLCBldmFsPUZBTFNFfQ0KIyMjIyAqUHNldWRvbW9uYXMgZ3JhbWluaXMqDQoNCiMjIyMgSGVhdG1hcCB3aXRoIGJpY2x1c3RlcmluZyAjIyMjDQptZXNzYWdlKCJoZWF0bWFwIHdpdGggYmljbHVzdGVyaW5nIikNCg0KYW5ub3RfY2x1c3RfcHNlZ3IgPSBkYXRhLmZyYW1lKGFzLmZhY3RvcihnZW5lX3N0YXRfbm9ybV9wc2VnciRjbHVzdGVyKSkNCmNvbG5hbWVzKGFubm90X2NsdXN0X3BzZWdyKSA8LSAiY2x1c3RlciINCnJvd25hbWVzKGFubm90X2NsdXN0X3BzZWdyKSA8LSByb3duYW1lcyhwc2Vncl9jbHIpDQoNCnBoZWF0bWFwKHBzZWdyX2NsciwNCiAgICAgICAgIGNsdXN0ZXJfcm93cyA9IFRSVUUsDQogICAgICAgICBjbHVzdGVyX2NvbHMgPSBUUlVFLA0KICAgICAgICAgY2x1c3RlcmluZ19kaXN0YW5jZV9yb3dzID0gZGlzdF9nZW5lX3BlYXJzb25fcHNlZ3IsDQogICAgICAgICBjbHVzdGVyaW5nX2Rpc3RhbmNlX2NvbHMgPSBkaXN0X3BlYXJzb25fcHNlZ3IsDQogICAgICAgICBib3JkZXJfY29sb3IgPSBOQSwNCiAgICAgICAgIHNob3dfcm93bmFtZXMgPSBGQUxTRSwNCiAgICAgICAgIGFubm90YXRpb25fcm93ID0gYW5ub3RfY2x1c3RfcHNlZ3IsDQogICAgICAgICBhbm5vdGF0aW9uX25hbWVzX3JvdyA9IEZBTFNFLA0KICAgICAgICAgY3V0cmVlX3Jvd3MgPSAzLA0KICAgICAgICAgY3V0cmVlX2NvbHMgPSAyLA0KICAgICAgICAgc2NhbGUgPSAicm93IiwNCiAgICAgICAgIHVzZV9yYXN0ZXIgPSBUUlVFLA0KICAgICAgICAgYW5nbGVfY29sID0gIjAiLA0KICAgICAgICAgbWFpbiA9ICJCaWNsdXN0ZXJpbmcgb2YgUC4gZ3JhbWluaXMgZ2VuZXMgKFBlYXJzb24gZGlzdGFuY2UpIA0KYW5kIHNhbXBsZXMgKFBlYXJzb24gZGlzdGFuY2UpIikNCg0KYGBgDQoNCg0KDQpgYGB7ciBoZWF0bWFwX3BzZXN5LCBmaWcuaGVpZ2h0PTcsIGZpZy53aWR0aD03LCBpbmNsdWRlPUZBTFNFLCBlY2hvPUZBTFNFLCBldmFsPUZBTFNFfQ0KIyMjIyAqUHNldWRvbW9uYXMgc3lyaW5nYWUqDQoNCiMjIyMgSGVhdG1hcCB3aXRoIGJpY2x1c3RlcmluZyAjIyMjDQptZXNzYWdlKCJoZWF0bWFwIHdpdGggYmljbHVzdGVyaW5nIikNCg0KYW5ub3RfY2x1c3RfcHNlc3kgPSBkYXRhLmZyYW1lKGFzLmZhY3RvcihnZW5lX3N0YXRfbm9ybV9wc2VzeSRjbHVzdGVyKSkNCmNvbG5hbWVzKGFubm90X2NsdXN0X3BzZXN5KSA8LSAiY2x1c3RlciINCnJvd25hbWVzKGFubm90X2NsdXN0X3BzZXN5KSA8LSByb3duYW1lcyhwc2VzeV9jbHIpDQoNCnBoZWF0bWFwKHBzZXN5X2NsciwNCiAgICAgICAgIGNsdXN0ZXJfcm93cyA9IFRSVUUsDQogICAgICAgICBjbHVzdGVyX2NvbHMgPSBUUlVFLA0KICAgICAgICAgY2x1c3RlcmluZ19kaXN0YW5jZV9yb3dzID0gZGlzdF9nZW5lX3BlYXJzb25fcHNlc3ksDQogICAgICAgICBjbHVzdGVyaW5nX2Rpc3RhbmNlX2NvbHMgPSBkaXN0X3BlYXJzb25fcHNlc3ksDQogICAgICAgICBib3JkZXJfY29sb3IgPSBOQSwNCiAgICAgICAgIHNob3dfcm93bmFtZXMgPSBGQUxTRSwNCiAgICAgICAgIGFubm90YXRpb25fcm93ID0gYW5ub3RfY2x1c3RfcHNlc3ksDQogICAgICAgICBhbm5vdGF0aW9uX25hbWVzX3JvdyA9IEZBTFNFLA0KICAgICAgICAgY3V0cmVlX3Jvd3MgPSA2LA0KICAgICAgICAgY3V0cmVlX2NvbHMgPSAyLA0KICAgICAgICAgc2NhbGUgPSAicm93IiwNCiAgICAgICAgIHVzZV9yYXN0ZXIgPSBUUlVFLA0KICAgICAgICAgYW5nbGVfY29sID0gIjAiLA0KICAgICAgICAgbWFpbiA9ICJCaWNsdXN0ZXJpbmcgb2YgUC4gc3lyaW5nYWUgZ2VuZXMgKFBlYXJzb24gZGlzdGFuY2UpIA0KYW5kIHNhbXBsZXMgKFBlYXJzb24gZGlzdGFuY2UpIikNCmBgYA0KDQoNCg0KYGBge3IgaGVhdG1hcF9yaG9lbiwgZmlnLmhlaWdodD03LCBmaWcud2lkdGg9NywgaW5jbHVkZT1GQUxTRSwgZWNobz1GQUxTRSwgZXZhbD1GQUxTRX0NCiMjIyMgKlJob2RvY29jY3VzIGVuY2xlbnNpcyoNCg0KIyMjIyBIZWF0bWFwIHdpdGggYmljbHVzdGVyaW5nICMjIyMNCm1lc3NhZ2UoImhlYXRtYXAgd2l0aCBiaWNsdXN0ZXJpbmciKQ0KDQphbm5vdF9jbHVzdF9yaG9lbiA9IGRhdGEuZnJhbWUoYXMuZmFjdG9yKGdlbmVfc3RhdF9ub3JtX3Job2VuJGNsdXN0ZXIpKQ0KY29sbmFtZXMoYW5ub3RfY2x1c3RfcmhvZW4pIDwtICJjbHVzdGVyIg0Kcm93bmFtZXMoYW5ub3RfY2x1c3RfcmhvZW4pIDwtIHJvd25hbWVzKHJob2VuX2NscikNCg0KcGhlYXRtYXAocmhvZW5fY2xyLA0KICAgICAgICAgY2x1c3Rlcl9yb3dzID0gVFJVRSwNCiAgICAgICAgIGNsdXN0ZXJfY29scyA9IFRSVUUsDQogICAgICAgICBjbHVzdGVyaW5nX2Rpc3RhbmNlX3Jvd3MgPSBkaXN0X2dlbmVfcGVhcnNvbl9yaG9lbiwNCiAgICAgICAgIGNsdXN0ZXJpbmdfZGlzdGFuY2VfY29scyA9IGRpc3RfcGVhcnNvbl9yaG9lbiwNCiAgICAgICAgIGJvcmRlcl9jb2xvciA9IE5BLA0KICAgICAgICAgc2hvd19yb3duYW1lcyA9IEZBTFNFLA0KICAgICAgICAgYW5ub3RhdGlvbl9yb3cgPSBhbm5vdF9jbHVzdF9yaG9lbiwNCiAgICAgICAgIGFubm90YXRpb25fbmFtZXNfcm93ID0gRkFMU0UsDQogICAgICAgICBjdXRyZWVfcm93cyA9IDYsDQogICAgICAgICBjdXRyZWVfY29scyA9IDIsDQogICAgICAgICBzY2FsZSA9ICJyb3ciLA0KICAgICAgICAgdXNlX3Jhc3RlciA9IFRSVUUsDQogICAgICAgICBhbmdsZV9jb2wgPSAiMCIsDQogICAgICAgICBtYWluID0gIkJpY2x1c3RlcmluZyBvZiBSLiBlbmNsZW5zaXMgZ2VuZXMgKFBlYXJzb24gZGlzdGFuY2UpIA0KYW5kIHNhbXBsZXMgKFBlYXJzb24gZGlzdGFuY2UpIikNCmBgYA0KDQojIyBEaWZmZXJlbnRpYWwgYW5hbHlzZXMgd2l0aCBNVFhtb2RlbCAod2hvbGUgYXNzZW1ibGFnZSBtZXRhdHJhbnNjcmlwdG9taWNzIGRhdGEpDQoNCk1UWG1vZGVsIHdhcyB1c2VkIG9uIHRoZSBjb21wbGV0ZSBhc3NlbWJsYWdlIGF0IG9uY2UuIA0KQXMgaW5wdXQsIGZpbHRlcmVkIGRhdGEgKHdpdGhvdXQgdGhlIHVuZGV0ZWN0ZWQgZ2VuZXMpIGJ1dCB1bnRyYW5zZm9ybWVkIHdhcyBnaXZlbiB0byBNVFhtb2RlbCwgYXMgaXQgcGVyZm9ybXMgaXRzIG93biBDTFIgbm9ybWFsaXNhdGlvbi4gDQoNCmBhbmFseXNpc19tZXRob2QgPSAnTE0nYCANCg0KYGNvcnJlY3Rpb24gPSAnQkgnYCwgDQoNCg0KDQpgYGB7ciBmaXRfZGF0YV9yYXcsIGV2YWw9RkFMU0V9DQpmaXRfZGF0YSA8LSBNVFhtb2RlbCgNCiAgICBjb3VudHNfc3RhbmRhcmQsIG1ldGFkYXRhX3RhYmxlLCAnTVRYbW9kZWxfb3V0cHV0JywNCiAgICBjb3JlcyA9IDIsDQogICAgZml4ZWRfZWZmZWN0cyA9IGMoJ3RlbXBlcmF0dXJlJyksDQogICAgcmVmZXJlbmNlID0gYygidGVtcGVyYXR1cmUsNSIpLA0KICAgIG1pbl9hYnVuZGFuY2UgPSAwLA0KICAgIG1pbl9wcmV2YWxlbmNlID0gMCwNCiAgICBub3JtYWxpemF0aW9uID0gJ0NMUicsDQogICAgYW5hbHlzaXNfbWV0aG9kID0gJ0xNJywNCiAgICBjb3JyZWN0aW9uID0gJ0JIJywNCiAgICBzdGFuZGFyZGl6ZSA9IEZBTFNFLA0KICAgIHRyYW5zZm9ybSA9ICdOT05FJywNCiAgICBwbG90X3NjYXR0ZXIgPSBGQUxTRSwNCiAgICBwbG90X2hlYXRtYXAgPSBUUlVFKQ0KDQpgYGANCg0KDQoNCmBgYHtyIGZpbHRlcl9NVFhtb2RlbF9yZXN1bHRzfQ0KDQojIExvYWRpbmcgTVRYbW9kZWwgcmVzdWx0cw0KcmVzX210eCA8LSByZWFkLmNzdigiLi4vc2NyaXB0cy9NVFhtb2RlbF9vdXRwdXQvYWxsX3Jlc3VsdHMudHN2Iiwgc2VwPSJcdCIsIHJvdy5uYW1lcz0iZmVhdHVyZSIpDQoNCg0KdHJ5Q2F0Y2goew0KICBpbm5lcl9qb2luKHJvd25hbWVzX3RvX2NvbHVtbihyZXNfbXR4KSwgYW5ub3RhdGlvbl90YWJsZV9maWcsIGJ5PWMoInJvd25hbWUiID0gImdlbmUiKSkgLT4gcmVzX210eF9maWcNCiAgcmVzX210eF9maWcgJT4lIGZpbHRlcihhYnMocXZhbCkgPD0gMC4yKSAtPiByZXNfbXR4X2ZpZw0KICB9LA0KICBlcnJvcj1mdW5jdGlvbihlKXtzdHIoZSkNCiAgfQ0KKQ0KDQoNCiMjIERhdGEgZm9yIGZpbmFsIHRhYmxlDQphbm5vdGF0aW9uX3RhYmxlX2xvbmcgPC0gcmVhZC5jc3YoIi4uL2RhdGEvYW5ub3RhdGlvbnNfZmluYWxfY29tbXVuaXR5X2xvbmcyLnRzdiIsIHNlcD0iXHQiLCByb3cubmFtZXMgPSAiR2VuZWlkIikNCg0KdHJ5Q2F0Y2goew0KICBhbm5vdGF0aW9uX3RhYmxlX2xvbmckZ2VuZSA8LSByb3cubmFtZXMoYW5ub3RhdGlvbl90YWJsZV9sb25nKQ0KICBpbm5lcl9qb2luKHJvd25hbWVzX3RvX2NvbHVtbihyZXNfbXR4KSwgYW5ub3RhdGlvbl90YWJsZV9sb25nLCBieT1jKCJyb3duYW1lIiA9ICJnZW5lIikpIC0+IHJlc19tdHhfZmlsdA0KICByZXNfbXR4X2ZpbHQgJT4lIGZpbHRlcihhYnMocXZhbCkgPD0gMC4yKSAtPiByZXNfbXR4X2ZpbHQNCiAgfSwNCiAgZXJyb3I9ZnVuY3Rpb24oZSl7c3RyKGUpDQogIH0NCikNCg0KYGBgDQoNCg0KIyMgRGlmZmVyZW50aWFsIGFuYWx5c2VzIHdpdGggREVTZXEyIChtZXRhdHJhbnNjcmlwdG9taWNzIGRhdGEgc3BlY2llcyBieSBzcGVjaWVzKQ0KDQpBcyBhIGNvbXBsZW1lbnRhcnkgYXBwcm9hY2gsIHNlcGFyYXRlIGRpZmZlcmVudGlhbCBhbmFseXNlcyB3ZXJlIGNvbmR1Y3RlZCBmb3IgZWFjaCBzcGVjaWVzIHNlcGFyYXRlbHkgd2l0aCBERVNlcTIuDQoNCiMjIyBERVNlcTIgYW5hbHlzZXMgcHJlcGFyYXRpb24NCg0KYGBge3IgY29udmVydGluZ190b19mYWN0b3JzfQ0KbWV0YWRhdGFfdGFibGUkdGVtcGVyYXR1cmUgPC0gZmFjdG9yKG1ldGFkYXRhX3RhYmxlJHRlbXBlcmF0dXJlKQ0KDQpgYGANCg0KV2UgdXNlIGZpbHRlcmVkIGNvdW50cyBkYXRhIChvbmx5IGdlbmVzIGRldGVjdGVkIGluIGF0IGxlYXN0IDcwJSBvZiBiaW9sb2dpY2FsIHNhbXBsZXMpLiBaZXJvZXMgYXJlIGNvbnNlcnZlZCBhcyBpcy4gV2Ugcm91bmQgdGhlIGNvdW50cyBtYXRyaXggYmVmb3JlIHBlcmZvcm1pbmcgREVTZXEyIGFuYWx5c2lzLg0KDQpgYGB7ciBERVNlcTJfZGZ9DQpkaW9odV9kZiA8LSBjb3VudHNfZmlsdGVyZWRbZGlvaHVfZ2VuZWlkLF0NCnBzZWdyX2RmIDwtIGNvdW50c19maWx0ZXJlZFtwc2Vncl9nZW5laWQsXQ0KcHNlc3lfZGYgPC0gY291bnRzX2ZpbHRlcmVkW3BzZXN5X2dlbmVpZCxdDQpyaG9lbl9kZiA8LSBjb3VudHNfZmlsdGVyZWRbcmhvZW5fZ2VuZWlkLF0NCg0KYGBgDQoNCmBgYHtyIGluaXRpYWxpemVfcmVzX2dlbnVzX2RmfQ0KcmVzX2RmIDwtIGRhdGEuZnJhbWUoZ2VuZT1jaGFyYWN0ZXIoKSwgDQogICAgICAgICAgICAgICAgICAgICBiYXNlTWVhbj1udW1lcmljKCksDQogICAgICAgICAgICAgICAgICAgICBsb2cyRm9sZENoYW5nZT1udW1lcmljKCksIA0KICAgICAgICAgICAgICAgICAgICAgbGZjU0U9bnVtZXJpYygpLCANCiAgICAgICAgICAgICAgICAgICAgIHN0YXQ9bnVtZXJpYygpLCANCiAgICAgICAgICAgICAgICAgICAgIHB2YWx1ZT1udW1lcmljKCksIA0KICAgICAgICAgICAgICAgICAgICAgcGFkaj1udW1lcmljKCksIA0KICAgICAgICAgICAgICAgICAgICAgY29uZGl0aW9uPWZhY3RvcigpLA0KICAgICAgICAgICAgICAgICAgICAgU0FNUExFX0NPTVBBUklTT049ZmFjdG9yKCksDQogICAgICAgICAgICAgICAgICAgICBvcmdhbmlzbT1mYWN0b3IoKSkNCmBgYA0KDQojIyMgKkRpb3N6ZWdpYSBodW5nYXJpY2EqDQoNCmBgYHtyIERFU2VxMl9kaW9odV8yMDI0fQ0KY29tcHV0ZV9kZXNlcTJfYW5hbHlzaXMoZGlvaHVfZGYsIA0KICAgICAgICAgICAgICAgICAgICAgICAgbWV0YWRhdGFfdGFibGUsDQogICAgICAgICAgICAgICAgICAgICAgICAjc3Vic2V0X3ZhciA9ICJ0ZW1wZXJhdHVyZSIsIA0KICAgICAgICAgICAgICAgICAgICAgICAgI3NlbGVjdCA9ICIzLjUiLCANCiAgICAgICAgICAgICAgICAgICAgICAgIGNvbnRyYXN0X2NvbD0idGVtcGVyYXR1cmUiLCANCiAgICAgICAgICAgICAgICAgICAgICAgIHJlZj0iNSIsIA0KICAgICAgICAgICAgICAgICAgICAgICAgdGVzdGVkPSIxNyIpIC0+IHJlcw0KDQp0cnlDYXRjaCh7cmVzJG9yZ2FuaXNtIDwtICJELiBodW5nYXJpY2EifSwNCiAgZXJyb3I9ZnVuY3Rpb24oZSl7c3RyKGUpICMgcHJpbnRzIHN0cnVjdHVyZSBvZiBleGNlcHRpb24NCiAgfSkNCg0KcmVzX2RmIDwtIHJiaW5kKHJlc19kZiwgcmVzKSANCg0KYGBgDQoNCiMjIyAqUmhvZG9jb2NjdXMgZW5jbGVuc2lzKg0KDQpgYGB7ciBERVNlcTJfcmhvZW5fMjAyNH0NCg0KY29tcHV0ZV9kZXNlcTJfYW5hbHlzaXMocmhvZW5fZGYsIA0KICAgICAgICAgICAgICAgICAgICAgICAgbWV0YWRhdGFfdGFibGUsDQogICAgICAgICAgICAgICAgICAgICAgICAjc3Vic2V0X3ZhciA9ICJ0ZW1wZXJhdHVyZSIsIA0KICAgICAgICAgICAgICAgICAgICAgICAgI3NlbGVjdCA9ICIzLjUiLCANCiAgICAgICAgICAgICAgICAgICAgICAgIGNvbnRyYXN0X2NvbD0idGVtcGVyYXR1cmUiLCANCiAgICAgICAgICAgICAgICAgICAgICAgIHJlZj0iNSIsIA0KICAgICAgICAgICAgICAgICAgICAgICAgdGVzdGVkPSIxNyIpIC0+IHJlcw0KDQp0cnlDYXRjaCh7cmVzJG9yZ2FuaXNtIDwtICJSLiBlbmNsZW5zaXMifSwNCiAgZXJyb3I9ZnVuY3Rpb24oZSl7c3RyKGUpDQogIH0pDQoNCnJlc19kZiA8LSByYmluZChyZXNfZGYsIHJlcykgDQoNCmBgYA0KDQojIyMgKlBzZXVkb21vbmFzIHN5cmluZ2FlKg0KDQpgYGB7ciBERVNlcTJfcHNlc3lfMjAyNH0NCg0KY29tcHV0ZV9kZXNlcTJfYW5hbHlzaXMocHNlc3lfZGYsIA0KICAgICAgICAgICAgICAgICAgICAgICAgbWV0YWRhdGFfdGFibGUsDQogICAgICAgICAgICAgICAgICAgICAgICAjc3Vic2V0X3ZhciA9ICJ0ZW1wZXJhdHVyZSIsIA0KICAgICAgICAgICAgICAgICAgICAgICAgI3NlbGVjdCA9ICIzLjUiLCANCiAgICAgICAgICAgICAgICAgICAgICAgIGNvbnRyYXN0X2NvbD0idGVtcGVyYXR1cmUiLCANCiAgICAgICAgICAgICAgICAgICAgICAgIHJlZj0iNSIsIA0KICAgICAgICAgICAgICAgICAgICAgICAgdGVzdGVkPSIxNyIpIC0+IHJlcw0KDQp0cnlDYXRjaCh7cmVzJG9yZ2FuaXNtIDwtICJQLiBzeXJpbmdhZSJ9LA0KICBlcnJvcj1mdW5jdGlvbihlKXtzdHIoZSkNCiAgfSkNCg0KcmVzX2RmIDwtIHJiaW5kKHJlc19kZiwgcmVzKSANCg0KYGBgDQoNCiMjIyAqUHNldWRvbW9uYXMgZ3JhbWluaXMqDQoNCmBgYHtyIERFU2VxMl9wc2Vncl8yMDI0fQ0KDQpjb21wdXRlX2Rlc2VxMl9hbmFseXNpcyhwc2Vncl9kZiwgDQogICAgICAgICAgICAgICAgICAgICAgICBtZXRhZGF0YV90YWJsZSwNCiAgICAgICAgICAgICAgICAgICAgICAgICNzdWJzZXRfdmFyID0gInRlbXBlcmF0dXJlIiwgDQogICAgICAgICAgICAgICAgICAgICAgICAjc2VsZWN0ID0gIjMuNSIsIA0KICAgICAgICAgICAgICAgICAgICAgICAgY29udHJhc3RfY29sPSJ0ZW1wZXJhdHVyZSIsIA0KICAgICAgICAgICAgICAgICAgICAgICAgcmVmPSI1IiwgDQogICAgICAgICAgICAgICAgICAgICAgICB0ZXN0ZWQ9IjE3IikgLT4gcmVzDQoNCnRyeUNhdGNoKHtyZXMkb3JnYW5pc20gPC0gIlAuIGdyYW1pbmlzIn0sDQogIGVycm9yPWZ1bmN0aW9uKGUpe3N0cihlKQ0KICB9KQ0KDQpyZXNfZGYgPC0gcmJpbmQocmVzX2RmLCByZXMpIA0KDQpgYGANCg0KIyMjIEZpbHRlciBERVNlcTIgZGF0YQ0KDQpgYGB7ciBwcmVwX2RmX0RFU2VxMl9wbG90fQ0KDQoNCnRyeUNhdGNoKHsNCiAgcmVzX2RmICU+JSBpbm5lcl9qb2luKGFubm90YXRpb25fdGFibGVfZmlnLCBieT0iZ2VuZSIpIC0+IHRtcF9yZXNfZGYNCiAgdG1wX3Jlc19kZiAlPiUgZmlsdGVyKHBhZGogPD0gMC4yKSAtPiByZXNfZGVzZXFfZmlnIA0KICB9LA0KICBlcnJvcj1mdW5jdGlvbihlKXtzdHIoZSkNCiAgfQ0KKQ0KDQpybSh0bXBfcmVzX2RmKQ0KDQojIyBkYXRhIGZvciBmaW5hbCB0YWJsZQ0KYW5ub3RhdGlvbl90YWJsZV9sb25nIDwtIHJlYWQuY3N2KCIuLi9kYXRhL2Fubm90YXRpb25zX2ZpbmFsX2NvbW11bml0eV9sb25nMi50c3YiLCBzZXA9Ilx0Iiwgcm93Lm5hbWVzID0gIkdlbmVpZCIpDQoNCnRyeUNhdGNoKHsNCiAgYW5ub3RhdGlvbl90YWJsZV9sb25nJGdlbmUgPC0gcm93Lm5hbWVzKGFubm90YXRpb25fdGFibGVfbG9uZykNCiAgcmVzX2RmICU+JSBpbm5lcl9qb2luKGFubm90YXRpb25fdGFibGVfbG9uZywgYnk9ImdlbmUiKSAtPiByZXNfZGYNCiAgcmVzX2RmICU+JSBmaWx0ZXIocGFkaiA8PSAwLjIpIC0+IHJlc19kZXNlcV9maWx0ICMgJiBhYnMobG9nMkZvbGRDaGFuZ2UpID49IDENCiAgfSwNCiAgZXJyb3I9ZnVuY3Rpb24oZSl7c3RyKGUpDQogIH0NCikNCmBgYA0KDQojIyMgU2F2ZSBkaWZmZXJlbnRpYWxseSBleHByZXNzZWQgZ2VuZXMgKERFR3MpIGFsbCBtZXRob2RzDQoNCmBgYHtyIHNhdmVfREVHX2NvbW11X2FsbH0NCg0KdGVtcF9kZiA8LSByaWdodF9qb2luKHJvd25hbWVzX3RvX2NvbHVtbihyZXNfbXR4KSwgYW5ub3RhdGlvbl90YWJsZV9sb25nLCBqb2luX2J5KCJyb3duYW1lIj09ImdlbmUiKSkNCnJlbmFtZSh0ZW1wX2RmLCAiZ2VuZSIgPSAicm93bmFtZSIpIC0+IHRlbXBfZGYNCg0KDQoNCmFsbF9nZW5lcyA8LSBmdWxsX2pvaW4ocmVzX2RmLCB0ZW1wX2RmLCAgYnk9YygiZ2VuZSIsICJsb2N1c190YWciLCAidHJhbnNjcmlwdElkIiwgIk9yZ2FuaXNtIiwNCiAgICAgICAgICAgICAgICAgICAgICAgICAgICAgICAgICAgICAgICAgICAgICAiQ2hyIiwgIlN0YXJ0IiwgIkVuZCIsICJTdHJhbmQiLCAiTGVuZ3RoIiwNCiAgICAgICAgICAgICAgICAgICAgICAgICAgICAgICAgICAgICAgICAgICAgICAicHJvZHVjdCIsICJDT0dfcHJvY2VzcyIsICJDT0dfY2F0ZWdvcnkiLA0KICAgICAgICAgICAgICAgICAgICAgICAgICAgICAgICAgICAgICAgICAgICAgICJDT0dpZCIsICJHT3MiLCAiQ09HX2NhdCIsICJDT0dfY2F0ZWdvcnlfbG9uZyIsDQogICAgICAgICAgICAgICAgICAgICAgICAgICAgICAgICAgICAgICAgICAgICAgImVjTnVtIiApLA0KICAgICAgICAgICAgICAgICAgICAgICBzdWZmaXggPSBjKCIiLCAidyIpKQ0KDQpybSh0ZW1wX2RmKQ0KY29sdW1uc190b19yZW1vdmUgPC0gZ3JlcCgiLnciLCBuYW1lcyhhbGxfZ2VuZXMpKQ0KYWxsX2dlbmVzICU+JSBkcGx5cjo6c2VsZWN0KC1jb2x1bW5zX3RvX3JlbW92ZSkgJT4lIGZpbHRlcihxdmFsPD0wLjIgfCBwYWRqPD0wLjIgKSAtPiBhbGxfZ2VuZXNfZmlsdGVyZWQNCg0Kd3JpdGUudGFibGUoYWxsX2dlbmVzX2ZpbHRlcmVkLCAiLi4vcmVzdWx0cy9ERUdfYWxsX21ldGhvZHNfY29tbXVuaXR5LnRzdiIsIHNlcD0nXHQnLCByb3cubmFtZXMgPSBGQUxTRSkNCg0Kd3JpdGUudGFibGUocmVzX2RmLCAiLi4vcmVzdWx0cy9ERUdfREVTZXEyX2FsbF9jb21tdW5pdHkudHN2Iiwgc2VwPSdcdCcsIHJvdy5uYW1lcyA9IEZBTFNFKQ0Kc2lnbl9kZXNlcTJfZGYgPC0gcmVzX2RmDQpgYGANCg0KIyMgUGxvdCB0cmFuc2NyaXB0IGV4cHJlc3Npb24gY29lZmZpY2llbnQgKFNEIHZzIFdOKQ0KDQpgYGB7ciBwbG90X2FsbF9kZWdzX0ZpZ3VyZTRfcGFuZWxBfQ0KDQojIyMjIEJvdGggbWV0aG9kcyBERUdzDQoNCg0KDQpmdWxsX2pvaW4oYWxsX2dlbmVzX2ZpbHRlcmVkLCBhbm5vdGF0aW9uX3RhYmxlX2ZpZywgYnk9ImdlbmUiKSAtPiBkYXRhDQoNCg0KZGF0YSR0aXRsZSA8LSAiRGlmZmVyZW50aWFsbHkgZXhwcmVzc2VkIGdlbmVzIFNEIHZzIFdOIGJ5IHN0cmFpbiAoREVTZXEyICYgTVRYbW9kZWwpIg0KZGF0YSRPcmdhbmlzbSA8LSBmYWN0b3IoZGF0YSRvcmdhbmlzbSwgbGV2ZWxzPWMoIkQuIGh1bmdhcmljYSIsICJQLiBncmFtaW5pcyIsICJQLiBzeXJpbmdhZSIsICJSLiBlbmNsZW5zaXMiKSkNCg0KDQoNCg0KY3VzdG9tX3N0cmlwcyA8LSBzdHJpcF9uZXN0ZWQoYmFja2dyb3VuZF94ID0gZWxlbV9saXN0X3JlY3QoZmlsbCA9IGMoImxpZ2h0Z3JleSIsIA0KICAgICAgICAgICAgICAgICAgICAgICAgICAgICAgICAgICAgICAgICAgICAgICAgICAgICAgICAgICAgICAgICAgICAgc3BlY2llc19jb2xvdXJzW1siRC5odW5nYXJpY2EiXV0sIA0KICAgICAgICAgICAgICAgICAgICAgICAgICAgICAgICAgICAgICAgICAgICAgICAgICAgICAgICAgICAgICAgICAgICAgc3BlY2llc19jb2xvdXJzW1siUC5zeXJpbmdhZSJdXSwgDQogICAgICAgICAgICAgICAgICAgICAgICAgICAgICAgICAgICAgICAgICAgICAgICAgICAgICAgICAgICAgICAgICAgICBzcGVjaWVzX2NvbG91cnNbWyJSLmVuY2xlbnNpcyJdXSkpLA0KICAgICAgICAgICAgICAgICAgICAgICAgICAgICAgdGV4dF94ID0gbGlzdChlbGVtZW50X3RleHQoZmFjZSA9ICJwbGFpbiIsIGNvbG91ciA9ICJibGFjayIsIHNpemUgPSAxNyksDQogICAgICAgICAgICAgICAgICAgICAgICAgICAgICAgICAgICAgICAgICAgIGVsZW1lbnRfdGV4dChmYWNlID0gIml0YWxpYyIsIGNvbG91ciA9ICJ3aGl0ZSIsIHNpemUgPSAxNSksDQogICAgICAgICAgICAgICAgICAgICAgICAgICAgICAgICAgICAgICAgICAgIGVsZW1lbnRfdGV4dChmYWNlID0gIml0YWxpYyIsIGNvbG91ciA9ICJ3aGl0ZSIsIHNpemUgPSAxNSksIA0KICAgICAgICAgICAgICAgICAgICAgICAgICAgICAgICAgICAgICAgICAgICBlbGVtZW50X3RleHQoZmFjZSA9ICJpdGFsaWMiLCBjb2xvdXIgPSAid2hpdGUiLCBzaXplID0gMTUpKSwNCiAgICAgICAgICAgICAgICAgICAgICAgICAgICAgIGJ5X2xheWVyX3ggPSBGQUxTRSkNCg0KDQpnZ3Bsb3QoZGF0YSwgYWVzKHggPSBjb2VmLCB5ID0gQ09HX2NhdGVnb3J5LnksICBjb2xvciA9IGFmdGVyX3NjYWxlKGFscGhhKGZpbGwsIDAuMykpLCBmaWxsPUNPR19jYXRlZ29yeV9sb25nLnksIGFscGhhPTAuNywgbGFiZWw9bmFtZV9maWd1cmUpKSArIA0KICBnZW9tX3BvaW50KGFlcyhhbHBoYT0wLjcpLCBwb3NpdGlvbj0iZG9kZ2UiKSArDQogIGdlb21fdmlvbGluKGFlcyhhbHBoYT0wLjMpLCBzaG93LmxlZ2VuZCA9IFRSVUUpICsNCiAgYW5ub3RhdGUoInJlY3QiLCB4bWluPS1JbmYsIHhtYXg9MCwgeW1pbj0tSW5mLCB5bWF4PUluZiwgZmlsbD0iIzE4NGNhNSIsIGFscGhhPTAuMSkgKw0KICBhbm5vdGF0ZSgicmVjdCIsIHhtaW49MCwgeG1heD1JbmYsIHltaW49LUluZiwgeW1heD1JbmYsIGZpbGw9ImdvbGQiLCBhbHBoYT0wLjEpICsNCiAgZ2VvbV92bGluZSh4aW50ZXJjZXB0ID0gMCwgbGluZXR5cGU9ImRhc2hlZCIpICsNCiAgeWxhYigiQ09HIGNhdGVnb3J5IikgKw0KICB4bGFiKCJNVFhtb2RlbCBjb2VmZmljaWVudCIpICsNCiAgZ2dyZXBlbDo6Z2VvbV90ZXh0X3JlcGVsKG51ZGdlX3kgPSAwLjUsIHNlZ21lbnQuc2l6ZT0wLjEsIHNlZWQgPSA0MikgKw0KICBndWlkZXMoZmlsbD1ndWlkZV9sZWdlbmQobmNvbD00KSwgYWxwaGE9Im5vbmUiLCBjb2xvcj0ibm9uZSIpICsgI2NvbG9yPSJub25lIiwNCiAgc2NhbGVfeV9kaXNjcmV0ZShsaW1pdHM9cmV2KG5hbWVzKHZlY3RfQ09HX2NhdGVnb3J5X2xvbmcpKSkgKw0KICBzY2FsZV9jb2xvdXJfbWFudWFsKGxpbWl0cz12ZWN0X0NPR19jYXRlZ29yeV9sb25nLCB2YWx1ZXM9Q09HX2NvbG91cnMsIGRyb3A9RkFMU0UpICsgDQogIHNjYWxlX2ZpbGxfbWFudWFsKGxpbWl0cz12ZWN0X0NPR19jYXRlZ29yeV9sb25nLCB2YWx1ZXM9Q09HX2NvbG91cnMsIGRyb3A9RkFMU0UpICsNCiAgdGhlbWUoYXhpcy50aXRsZSA9IGVsZW1lbnRfdGV4dChzaXplPTEzLCAiRGlmZmVyZW50aWFsbHkgZXhwcmVzc2VkIGdlbmVzIFNEIHZzIFdOIiksDQogICAgICAgIGF4aXMudGV4dCA9IGVsZW1lbnRfdGV4dChzaXplPTEzKSwNCiAgICAgICAgc3RyaXAudGV4dC55ID0gZWxlbWVudF90ZXh0KHNpemUgPSAxNyksDQogICAgICAgICNzdHJpcC50ZXh0LnggPSBlbGVtZW50X3RleHQoc2l6ZSA9IDE3LCBmYWNlID0gIml0YWxpYyIpLA0KICAgICAgICBsZWdlbmQudGV4dCA9IGVsZW1lbnRfdGV4dChzaXplID0gMTIpLA0KICAgICAgICBsZWdlbmQudGl0bGUgPSBlbGVtZW50X2JsYW5rKCksDQogICAgICAgIGxlZ2VuZC5rZXkuc2l6ZSA9IHVuaXQoMC41LCAibGluZSIpLA0KICAgICAgICBsZWdlbmQucG9zaXRpb24gPSAiYm90dG9tIiwNCiAgICAgICAgcGFuZWwuYmFja2dyb3VuZCA9IGVsZW1lbnRfcmVjdChmaWxsID0gIiNmNGY0ZjQiKSkgKw0KICBmYWNldF9uZXN0ZWQofnRpdGxlICsgT3JnYW5pc20sIHN0cmlwID0gY3VzdG9tX3N0cmlwcywgZHJvcD1UUlVFKSAtPiBwbG90X2RlZ3MNCg0KIyBwcmludChwbG90X2RlZ3MpDQojIA0KIyBnZ3NhdmUocGxvdCA9IHBsb3RfZGVncywgZmlsZW5hbWUgPSAiLi4vZmlndXJlcy9ERUdfZGF5X3ZzX25pZ2h0X2NvbW11X2JvdGhfbWV0aG9kc192ZXJ0aWNhbC50aWZmIiwgZHBpID0gMzAwLCB3aWR0aCA9IDEzLCBoZWlnaHQgPSAxMywgYmc9IndoaXRlIikNCg0KYGBgDQoNCmBgYHtyIHBsb3RfYWxsX3RyYW5zY3JpcHRfRmlndXJlNF9wYW5lbEJ9DQoNCmNvbHVtbnNfdG9fcmVtb3ZlIDwtIGdyZXAoIi53IiwgbmFtZXMoYWxsX2dlbmVzKSkNCmFsbF9nZW5lcyAlPiUgZHBseXI6OnNlbGVjdCgtY29sdW1uc190b19yZW1vdmUpICU+JSBmaWx0ZXIoIWlzLm5hKGNvZWYpKSAtPiBhbGxfZ2VuZXNfdW5maWx0ZXJlZA0KDQpmdWxsX2pvaW4oYWxsX2dlbmVzX3VuZmlsdGVyZWQsIGFubm90YXRpb25fdGFibGVfbG9uZywgYnk9ImdlbmUiKSAlPiUgZmlsdGVyKCFpcy5uYShjb2VmKSkgLT4gZGF0YQ0KZGF0YVtkYXRhJENPR19jYXRlZ29yeV9sb25nLnkgPT0gIlM6IEZ1bmN0aW9uIFVua25vd24iLCAiQ09HX2NhdGVnb3J5X2xvbmcueSJdIDwtICJTOiBGdW5jdGlvbiB1bmtub3duIg0KZGF0YSR0aXRsZSA8LSAiQWxsIGRldGVjdGVkIGdlbmVzIg0KZGF0YSRzdWJ0aXRsZSA8LSAiQXNzZW1ibGFnZSINCmRhdGEkT3JnYW5pc20gPC0gZmFjdG9yKGRhdGEkb3JnYW5pc20sIGxldmVscz1jKCJELiBodW5nYXJpY2EiLCAiUC4gZ3JhbWluaXMiLCAiUC4gc3lyaW5nYWUiLCAiUi4gZW5jbGVuc2lzIikpDQpkYXRhJENPR19jYXRlZ29yeV9maWcgPC0gc3Vic3RyKGRhdGEkQ09HX2NhdGVnb3J5X2xvbmcueSwgMCwgMSkNCg0KDQpjdXN0b21fc3RyaXBzIDwtIHN0cmlwX25lc3RlZChiYWNrZ3JvdW5kX3ggPSBlbGVtX2xpc3RfcmVjdChmaWxsID0gYygibGlnaHRncmV5IiwgImJsYWNrIikpLA0KICAgICAgICAgICAgICAgICAgICAgICAgICAgICAgdGV4dF94ID0gbGlzdChlbGVtZW50X3RleHQoZmFjZSA9ICJwbGFpbiIsIGNvbG91ciA9ICJibGFjayIsIHNpemUgPSAxNyksDQogICAgICAgICAgICAgICAgICAgICAgICAgICAgICAgICAgICAgICAgICAgIGVsZW1lbnRfdGV4dChmYWNlID0gIml0YWxpYyIsIGNvbG91ciA9ICJ3aGl0ZSIsIHNpemUgPSAxNSkpLA0KICAgICAgICAgICAgICAgICAgICAgICAgICAgICAgYnlfbGF5ZXJfeCA9IEZBTFNFKQ0KDQpjdXN0b21fc3RyaXBzX3N0cmFpbnMgPC0gc3RyaXBfbmVzdGVkKGJhY2tncm91bmRfeCA9IGVsZW1fbGlzdF9yZWN0KGZpbGwgPSBjKCJsaWdodGdyZXkiLCANCiAgICAgICAgICAgICAgICAgICAgICAgICAgICAgICAgICAgICAgICAgICAgICAgICAgICAgICAgICAgICAgICAgICAgIHNwZWNpZXNfY29sb3Vyc1tbIkQuaHVuZ2FyaWNhIl1dLCANCiAgICAgICAgICAgICAgICAgICAgICAgICAgICAgICAgICAgICAgICAgICAgICAgICAgICAgICAgICAgICAgICAgICAgIHNwZWNpZXNfY29sb3Vyc1tbIlAuc3lyaW5nYWUiXV0sIA0KICAgICAgICAgICAgICAgICAgICAgICAgICAgICAgICAgICAgICAgICAgICAgICAgICAgICAgICAgICAgICAgICAgICAgc3BlY2llc19jb2xvdXJzW1siUi5lbmNsZW5zaXMiXV0pKSwNCiAgICAgICAgICAgICAgICAgICAgICAgICAgICAgIHRleHRfeCA9IGxpc3QoZWxlbWVudF90ZXh0KGZhY2UgPSAicGxhaW4iLCBjb2xvdXIgPSAiYmxhY2siLCBzaXplID0gMTcpLA0KICAgICAgICAgICAgICAgICAgICAgICAgICAgICAgICAgICAgICAgICAgICBlbGVtZW50X3RleHQoZmFjZSA9ICJpdGFsaWMiLCBjb2xvdXIgPSAid2hpdGUiLCBzaXplID0gMTUpLA0KICAgICAgICAgICAgICAgICAgICAgICAgICAgICAgICAgICAgICAgICAgICBlbGVtZW50X3RleHQoZmFjZSA9ICJpdGFsaWMiLCBjb2xvdXIgPSAid2hpdGUiLCBzaXplID0gMTUpLCANCiAgICAgICAgICAgICAgICAgICAgICAgICAgICAgICAgICAgICAgICAgICAgZWxlbWVudF90ZXh0KGZhY2UgPSAiaXRhbGljIiwgY29sb3VyID0gIndoaXRlIiwgc2l6ZSA9IDE1KSksDQogICAgICAgICAgICAgICAgICAgICAgICAgICAgICBieV9sYXllcl94ID0gRkFMU0UpDQoNCmdncGxvdChkYXRhLCBhZXMoeCA9IGNvZWYsIHkgPSBDT0dfY2F0ZWdvcnlfZmlnLGNvbG9yID0gYWZ0ZXJfc2NhbGUoYWxwaGEoZmlsbCwgMC4zKSksIGZpbGw9Q09HX2NhdGVnb3J5X2xvbmcueSwgYWxwaGE9MC45KSkgKyAjbGFiZWw9bmFtZV9maWd1cmUNCiAgIyBnZW9tX3BvaW50KGFlcyhhbHBoYT0wLjcpKSArICMsIHBvc2l0aW9uPSJkb2RnZSINCiAgZ2VvbV92aW9saW4oYWVzKGFscGhhPTAuMyksIHNob3cubGVnZW5kID0gVFJVRSkgKw0KICBnZW9tX2JveHBsb3Qob3V0bGllci5jb2xvdXIgPSAiYmxhY2siLCB3aWR0aD0wLjIsIGNvbG9yPSJ3aGl0ZSIsIG91dGxpZXIuYWxwaGEgPSAwLjQsIGFscGhhPTAuMiwgc2hvdy5sZWdlbmQgPSBGQUxTRSkgKw0KICBhbm5vdGF0ZSgicmVjdCIsIHhtaW49LUluZiwgeG1heD0wLCB5bWluPS1JbmYsIHltYXg9SW5mLCBmaWxsPSIjMTg0Y2E1IiwgYWxwaGE9MC4xKSArDQogIGFubm90YXRlKCJyZWN0IiwgeG1pbj0wLCB4bWF4PUluZiwgeW1pbj0tSW5mLCB5bWF4PUluZiwgZmlsbD0iZ29sZCIsIGFscGhhPTAuMSkgKw0KICBnZW9tX3ZsaW5lKHhpbnRlcmNlcHQgPSAwLCBsaW5ldHlwZT0iZGFzaGVkIikgKw0KICB5bGFiKCJDT0cgY2F0ZWdvcnkiKSArDQogIHhsYWIoIk1UWG1vZGVsIGNvZWZmaWNpZW50IikgKw0KICAjIGdncmVwZWw6Omdlb21fdGV4dF9yZXBlbChudWRnZV95ID0gMC41LCBzZWdtZW50LnNpemU9MC4xLCBzZWVkID0gNDIpICsNCiAgZ3VpZGVzKGZpbGw9Z3VpZGVfbGVnZW5kKG5jb2w9NCksIGFscGhhPSJub25lIiwgY29sb3I9Im5vbmUiKSArICNjb2xvcj0ibm9uZSIsDQogIHNjYWxlX3lfZGlzY3JldGUobGltaXRzPXJldihuYW1lcyh2ZWN0X0NPR19jYXRlZ29yeV9sb25nKSkpICsNCiAgc2NhbGVfY29sb3VyX21hbnVhbChsaW1pdHM9dmVjdF9DT0dfY2F0ZWdvcnlfbG9uZywgdmFsdWVzPUNPR19jb2xvdXJzLCBkcm9wPUZBTFNFKSArIA0KICBzY2FsZV9maWxsX21hbnVhbChsaW1pdHM9dmVjdF9DT0dfY2F0ZWdvcnlfbG9uZywgdmFsdWVzPUNPR19jb2xvdXJzLCBkcm9wPUZBTFNFKSArDQogIHRoZW1lKGF4aXMudGl0bGUgPSBlbGVtZW50X3RleHQoc2l6ZT0xMywgIkV4cHJlc3Npb24gY29lZmZpY2llbnQgKGluIFNEIHZzIFdOKSBvZiBhbGwgYXNzZW1ibGFnZSBkZXRlY3RlZCBnZW5lcyIpLA0KICAgICAgICBheGlzLnRleHQgPSBlbGVtZW50X3RleHQoc2l6ZT0xMyksDQogICAgICAgIHN0cmlwLnRleHQueSA9IGVsZW1lbnRfdGV4dChzaXplID0gMTcpLA0KICAgICAgICAjc3RyaXAudGV4dC54ID0gZWxlbWVudF90ZXh0KHNpemUgPSAxNywgZmFjZSA9ICJpdGFsaWMiKSwNCiAgICAgICAgbGVnZW5kLnRleHQgPSBlbGVtZW50X3RleHQoc2l6ZSA9IDEyKSwNCiAgICAgICAgbGVnZW5kLnRpdGxlID0gZWxlbWVudF9ibGFuaygpLA0KICAgICAgICBsZWdlbmQua2V5LnNpemUgPSB1bml0KDAuNSwgImxpbmUiKSwNCiAgICAgICAgbGVnZW5kLnBvc2l0aW9uID0gImJvdHRvbSIsDQogICAgICAgIHBhbmVsLmJhY2tncm91bmQgPSBlbGVtZW50X3JlY3QoZmlsbCA9ICIjZjRmNGY0IikpIC0+IHANCg0KICBwICsgZmFjZXRfbmVzdGVkKH50aXRsZSArIHN1YnRpdGxlLCBzdHJpcCA9IGN1c3RvbV9zdHJpcHMsIGRyb3A9VFJVRSkgLT4gcGxvdF9hc3NlbWJsYWdlDQogIHAgKyBmYWNldF9uZXN0ZWQofnRpdGxlICsgT3JnYW5pc20sIHN0cmlwID0gY3VzdG9tX3N0cmlwc19zdHJhaW5zLCBkcm9wPVRSVUUpICsgDQogICAgZ3VpZGVzKGZpbGw9Z3VpZGVfbGVnZW5kKG5jb2w9MyksIGFscGhhPSJub25lIiwgY29sb3I9Im5vbmUiKSArIA0KICAgIHRoZW1lKGxlZ2VuZC50ZXh0ID0gZWxlbWVudF90ZXh0KHNpemUgPSAxMCkpLT4gcGxvdF9zdHJhaW5zDQoNCiMgcHJpbnQocGxvdF9hc3NlbWJsYWdlKQ0KIyANCmdnc2F2ZShwbG90ID0gcGxvdF9hc3NlbWJsYWdlLCBmaWxlbmFtZSA9ICIuLi9maWd1cmVzL2V4cHJlc3NlZF9nZW5lc19NVFhfY29lZmZfYXNzZW1ibGFnZS50aWZmIiwgZHBpID0gMzAwLCB3aWR0aCA9IDEzLzMsIGhlaWdodCA9IDEzLCBiZz0id2hpdGUiKQ0KDQpnZ3NhdmUocGxvdCA9IHBsb3Rfc3RyYWlucywgZmlsZW5hbWUgPSAiLi4vZmlndXJlcy9leHByZXNzZWRfZ2VuZXNfTVRYX2NvZWZmX3N0cmFpbnMudGlmZiIsIGRwaSA9IDMwMCwgd2lkdGggPSAxMywgaGVpZ2h0ID0gMTMsIGJnPSJ3aGl0ZSIpDQoNCmBgYA0KDQpgYGB7ciBwbG90X0ZpZ3VyZTQsIGZpZy5oZWlnaHQ9MTIsIGZpZy5kcGk9MjAwLCBmaWcud2lkdGg9MTcuMzV9DQpwbG90X2FsbCA8LSBnZ2FycmFuZ2UocGxvdF9hc3NlbWJsYWdlLCBwbG90X2RlZ3MsIA0KICAgICAgICAgICAgICAgICAgICAgIGxhYmVscyA9IGMoIkEiLCAiQiIpLCANCiAgICAgICAgICAgICAgICAgICAgICBjb21tb24ubGVnZW5kPVRSVUUsDQogICAgICAgICAgICAgICAgICAgICAgbGVnZW5kPSJib3R0b20iLA0KICAgICAgICAgICAgICAgICAgICAgIG5yb3c9MSwNCiAgICAgICAgICAgICAgICAgICAgICB3aWR0aHMgPSBjKDEsIDMpKQ0KDQpwcmludChwbG90X2FsbCkNCmdnc2F2ZShwbG90ID0gcGxvdF9hbGwsIGZpbGVuYW1lID0gIi4uL2ZpZ3VyZXMvRmlndXJlXzQudGlmZiIsIGRwaSA9IDMwMCwgd2lkdGggPSAxMys0LjM1LCBoZWlnaHQgPSAxMywgYmc9IndoaXRlIikNCg0KcHJpbnQocGxvdF9zdHJhaW5zKQ0KYGBgDQoNCg0KDQojIyBWZW5uIGRpYWdyYW0gb2YgUk5BIGRpZmZlcmVudGlhbCBleHByZXNzaW9uIHJlc3VsdHMNCg0KYGBge3IgdmVubl9kaWFncmFtX01UWF9pbml0aWFsLCBlcnJvcj1GQUxTRSwgbWVzc2FnZT1GQUxTRSwgd2FybmluZz1GQUxTRSwgcmVzdWx0cz0naGlkZScsIGZpZy5oZWlnaHQ9OSwgZmlnLndpZHRoPTksIGZpZy5kcGk9MTAwfQ0KDQoNCmxpc3RfdmVubiA8LSBsaXN0KHJlc19kZXNlcV9maWx0WyhyZXNfZGVzZXFfZmlsdCRTQU1QTEVfQ09NUEFSSVNPTiA9PSAiMTdfVlNfNSIpLCAiZ2VuZSJdLA0KICAgICAgICAgICAgICAgICAgcmVzX210eF9maWx0WywgInJvd25hbWUiXSkNCg0KaW52aXNpYmxlKGdyaWQubmV3cGFnZSgpKSAgIA0KZHJhdy5wYWlyd2lzZS52ZW5uKGFyZWExID0gbGVuZ3RoKGxpc3RfdmVubltbMV1dKSwNCiAgICAgICAgICAgICAgICAgYXJlYTIgPSBsZW5ndGgobGlzdF92ZW5uW1syXV0pLA0KICAgICAgICAgICAgICAgICBjcm9zcy5hcmVhID0gbGVuZ3RoKGludGVyc2VjdC5WZWN0b3IobGlzdF92ZW5uW1sxXV0sIGxpc3RfdmVubltbMl1dKSksDQogICAgICAgICAgICAgICAgIGZpbGwgPSBjKCIjRDUzRjdGIiwgIiMwMzlFQkQiKSwNCiAgICAgICAgICAgICAgICAgbHR5ID0gImJsYW5rIiwNCiAgICAgICAgICAgICAgICAgZm9udGZhbWlseSA9ICJIZWx2ZXRpY2EiLA0KICAgICAgICAgICAgICAgICBjZXggPSByZXAoMiwgMyksDQogICAgICAgICAgICAgICAgIGNhdC5jZXggPSByZXAoMS41LCAyKSwNCiAgICAgICAgICAgICAgICAgY2F0LnBvcyA9IGMoLTUwLCA1MCksDQogICAgICAgICAgICAgICAgIGNhdC5kaXN0ID0gYygtMC4wNSwgLTAuMDUpLA0KICAgICAgICAgICAgICAgICBjYXQucHJvbXB0cyA9IFRSVUUsDQogICAgICAgICAgICAgICAgIGNhdC5jb2wgPSBjKCIjRDUzRjdGIiwgIiMwMzlFQkQiKSwNCiAgICAgICAgICAgICAgICAgY2F0LmZvbnRmYW1pbHkgPSAiSGVsdmV0aWNhIiwNCiAgICAgICAgICAgICAgICAgY2F0ZWdvcnkgPSBjKCJERVNlcSAgXG5hbG9uZSIsICJNVFhNb2RlbCAgXG5hc3NlbWJsYWdlIiksDQogICAgICAgICAgICAgICAgIHRpdGxlID0gIkRpZmZlcmVudGlhbGx5IGFidW5kYW50IGdlbmVzIGZvdW5kIGJ5IERFU2VxMiBhbmQgTVRYbW9kZWwiLA0KICAgICAgICAgICAgICAgICBtYXJnaW4gPSAwLjEpIC0+IHZlbm5fcGxvdA0KDQoNCiMgV3JpdGluZyB0byBmaWxlDQoNCmludmlzaWJsZShwbmcoZmlsZW5hbWUgPSAiLi4vZmlndXJlcy9WZW5uX2RpYWdyYW1fQUxERVhlMl9NVFhfREVTZXFfY29tbXVuaXR5LnBuZyIsIA0KICAgICB3aWR0aCA9IDEwMDAsIGhlaWdodCA9IDEwMDApKQ0KaW52aXNpYmxlKGdyaWQuZHJhdyh2ZW5uX3Bsb3QpKQ0KaW52aXNpYmxlKGRldi5vZmYoKSkNCmBgYA0KDQoNCmBgYHtyIG1lcmdlX2RpZmZlcmVudGlhbF9hbmFseXNlc19yZXN1bHRzfQ0KDQp2ZWN0X2RlZ3MgPC0gYWxsX2dlbmVzX2ZpbHRlcmVkJGdlbmUNCmNvdW50c19jbHJfZGVncyA8LSBhcy5tYXRyaXgoY291bnRzX2NscilbdmVjdF9kZWdzLF0NCmBgYA0KDQoNCiMjIERpZmZlcmVudGlhbCBNZXRhYm9saXRlcyBpbnRlbnNpdHkNCg0KDQpgYGB7ciBwcmVwYXJlX2ludGVuc2l0eV9tZXRhQl9kYXRhfQ0KDQppZGVudGlmaWVkX21ldGFib2xpdGVzIDwtIGxlZnRfam9pbihtZXRhYm9sb21pY3NfYW5ub3RhdGlvbnNbLGMoIm1ldGFib2xpdGUgaWRlbnRpZmljYXRpb24iLCAiSUQiKV0sDQogICAgICAgICAgICAgICAgICAgICAgICAgICAgICAgICAgICAgICAgcm93bmFtZXNfdG9fY29sdW1uKG1ldGFib2xvbWljc19hbGxfdGltZXNfZmlsdGVyZWQpLCBieSA9IGpvaW5fYnkoIklEIiA9PSAicm93bmFtZSIpKSAgDQoNCmlkZW50aWZpZWRfbWV0YWJvbGl0ZXMgPC0gaWRlbnRpZmllZF9tZXRhYm9saXRlc1tpZGVudGlmaWVkX21ldGFib2xpdGVzJGBtZXRhYm9saXRlIGlkZW50aWZpY2F0aW9uYCAhPSAidW5rbm93biIsXQ0KDQpyb3cubmFtZXMoaWRlbnRpZmllZF9tZXRhYm9saXRlcykgPC0gaWRlbnRpZmllZF9tZXRhYm9saXRlcyRgbWV0YWJvbGl0ZSBpZGVudGlmaWNhdGlvbmANCg0KaWRlbnRpZmllZF9tZXRhYm9saXRlcyA8LSBpZGVudGlmaWVkX21ldGFib2xpdGVzICU+JSB0KCkgJT4lIGRhdGEuZnJhbWUoKQ0KDQoNCmlkZW50aWZpZWRfbWV0YWJvbGl0ZXMgPC0gaWRlbnRpZmllZF9tZXRhYm9saXRlc1szOm5yb3coaWRlbnRpZmllZF9tZXRhYm9saXRlcyksXSANCmlkZW50aWZpZWRfbWV0YWJvbGl0ZXMkc2FtcGxlIDwtIHJvdy5uYW1lcyhpZGVudGlmaWVkX21ldGFib2xpdGVzKQ0KDQppZGVudGlmaWVkX21ldGFib2xpdGVzIDwtIGxlZnRfam9pbihpZGVudGlmaWVkX21ldGFib2xpdGVzLCByb3duYW1lc190b19jb2x1bW4obWV0YWRhdGFfdGFibGVfbWV0YUJfYWxsX3RpbWVzKSwgYnkgPSBqb2luX2J5KCJzYW1wbGUiID09ICJyb3duYW1lIikpDQoNCmlkZW50aWZpZWRfbWV0YWJvbGl0ZXMgJT4lIHJlbmFtZSgiMi1BbWlub2JlbnpvaWMgYWNpZCIgPSAiWDIuYW1pbm9iZW56b2ljLmFjaWQiLA0KICAgICAgICAgICAgICAgICAgICAgICAgICAgICAgICAgICAgICAiREwtTWV0aGlvbmluZSBzdWxmb3hpZGUiID0gIkRMLm1ldGhpb25pbmUuc3VsZm94aWRlIiwNCiAgICAgICAgICAgICAgICAgICAgICAgICAgICAgICAgICAgICAgIlB5cmlkb3hhbCIgPSAicHlyaWRveGFsIiwNCiAgICAgICAgICAgICAgICAgICAgICAgICAgICAgICAgICAgICAgIkQtUGFudG90aGVuaWMgYWNpZCIgPSAiRC5wYW50b3RoZW5pYy5hY2lkIiAsDQogICAgICAgICAgICAgICAgICAgICAgICAgICAgICAgICAgICAgICJONi1BY2V0eWwtTC1seXNpbmUiID0gIk42LmFjZXR5bC5MLmx5c2luZSIsDQogICAgICAgICAgICAgICAgICAgICAgICAgICAgICAgICAgICAgICJMLUdsdXRhbWljIGFjaWQiID0gIkwuZ2x1dGFtaWMuYWNpZCIsDQogICAgICAgICAgICAgICAgICAgICAgICAgICAgICAgICAgICAgICJMLUlzb2xldWNpbmUiID0gIkwuaXNvbGV1Y2luZSIsDQogICAgICAgICAgICAgICAgICAgICAgICAgICAgICAgICAgICAgICJCdXR5cnlsLUwtY2Fybml0aW5lIiA9ICJidXR5cnlsLkwuY2Fybml0aW5lIiwNCiAgICAgICAgICAgICAgICAgICAgICAgICAgICAgICAgICAgICAgIkFjZXR5bC1MLWNhcm5pdGluZSIgPSAiYWNldHlsLkwuY2Fybml0aW5lIiwNCiAgICAgICAgICAgICAgICAgICAgICAgICAgICAgICAgICAgICAgIklzb3ZhbGVyeWwtTC1jYXJuaXRpbmUiID0gImlzb3ZhbGVyeWwuTC5jYXJuaXRpbmUiKSAtPiBpZGVudGlmaWVkX21ldGFib2xpdGVzIA0KDQppZGVudGlmaWVkX21ldGFib2xpdGVzICU+JQ0KICBwaXZvdF9sb25nZXIoY29scyA9IGMoIjItQW1pbm9iZW56b2ljIGFjaWQiLCAiREwtTWV0aGlvbmluZSBzdWxmb3hpZGUiLCAiUHlyaWRveGFsIiwgDQogICAgICAgICAgICAgICAgICAgICAgICAiRC1QYW50b3RoZW5pYyBhY2lkIiwgIk42LUFjZXR5bC1MLWx5c2luZSIsICJMLUdsdXRhbWljIGFjaWQiLCANCiAgICAgICAgICAgICAgICAgICAgICAgICJMLUlzb2xldWNpbmUiLCAiQnV0eXJ5bC1MLWNhcm5pdGluZSIsICJBY2V0eWwtTC1jYXJuaXRpbmUiLCANCiAgICAgICAgICAgICAgICAgICAgICAgICJJc292YWxlcnlsLUwtY2Fybml0aW5lIiksIA0KICAgICAgICAgICAgICAgdmFsdWVzX3RvID0gImludGVuc2l0eSIpIC0+IGlkZW50aWZpZWRfbWV0YWJvbGl0ZXMNCiANCg0KICANCmlkZW50aWZpZWRfbWV0YWJvbGl0ZXNbaWRlbnRpZmllZF9tZXRhYm9saXRlcyR0ZW1wZXJhdHVyZSA9PSAxNyAmIGlkZW50aWZpZWRfbWV0YWJvbGl0ZXMkdGltZSA9PSAwLCAiY29uZGl0aW9uIl0gPC0gIlNEX1QwIg0KaWRlbnRpZmllZF9tZXRhYm9saXRlc1tpZGVudGlmaWVkX21ldGFib2xpdGVzJHRlbXBlcmF0dXJlID09IDUgJiBpZGVudGlmaWVkX21ldGFib2xpdGVzJHRpbWUgPT0gMCwgImNvbmRpdGlvbiJdIDwtICJXTl9UMCINCmlkZW50aWZpZWRfbWV0YWJvbGl0ZXNbaWRlbnRpZmllZF9tZXRhYm9saXRlcyR0ZW1wZXJhdHVyZSA9PSAxNyAmIGlkZW50aWZpZWRfbWV0YWJvbGl0ZXMkdGltZSA9PSAzLCAiY29uZGl0aW9uIl0gPC0gIlNEX1RGIg0KaWRlbnRpZmllZF9tZXRhYm9saXRlc1tpZGVudGlmaWVkX21ldGFib2xpdGVzJHRlbXBlcmF0dXJlID09IDUgJiBpZGVudGlmaWVkX21ldGFib2xpdGVzJHRpbWUgPT0gMywgImNvbmRpdGlvbiJdIDwtICJXTl9URiINCmlkZW50aWZpZWRfbWV0YWJvbGl0ZXNbaWRlbnRpZmllZF9tZXRhYm9saXRlcyR0ZW1wZXJhdHVyZSA9PSAxNywgImZpbGwiXSA8LSAiI0Y4QUQxOCINCmlkZW50aWZpZWRfbWV0YWJvbGl0ZXNbaWRlbnRpZmllZF9tZXRhYm9saXRlcyR0ZW1wZXJhdHVyZSA9PSA1LCAiZmlsbCJdIDwtICIjODlEREY4Ig0KaWRlbnRpZmllZF9tZXRhYm9saXRlc1tpZGVudGlmaWVkX21ldGFib2xpdGVzJHRlbXBlcmF0dXJlID09IDE3LCAiY29sb3VyIl0gPC0gIiNiMDc5MGIiDQppZGVudGlmaWVkX21ldGFib2xpdGVzW2lkZW50aWZpZWRfbWV0YWJvbGl0ZXMkdGVtcGVyYXR1cmUgPT0gNSwgImNvbG91ciJdIDwtICIjMWFhN2Q0Ig0KDQppZGVudGlmaWVkX21ldGFib2xpdGVzJGludGVuc2l0eSA8LSBhcy5udW1lcmljKGlkZW50aWZpZWRfbWV0YWJvbGl0ZXMkaW50ZW5zaXR5KQ0KDQoNCg0KYGBgDQoNCg0KIyMjIERpZmZlcmVudGlhbGx5IGFidW5kYW50IG1ldGFib2xpdGVzIGJldHdlZW4gU0QgYW5kIFdODQoNCmBgYHtyIHBsb3RfRmlndXJlMywgZmlnLmhlaWdodD03fQ0KICANCg0KaWRlbnRpZmllZF9tZXRhYm9saXRlcyAlPiUgDQogIGZpbHRlcihuYW1lICVpbiUgYygiMi1BbWlub2JlbnpvaWMgYWNpZCIsICJMLUdsdXRhbWljIGFjaWQiLA0KICAgICAgICAgICAgICAgICAgICAgIkRMLU1ldGhpb25pbmUgc3VsZm94aWRlIiwgIk42LUFjZXR5bC1MLWx5c2luZSIsDQogICAgICAgICAgICAgICAgICAgICAiRC1QYW50b3RoZW5pYyBhY2lkIiwgIlB5cmlkb3hhbCIgKSkgJT4lDQpmaWx0ZXIoY29uZGl0aW9uICVpbiUgYygiU0RfVEYiLCAiV05fVEYiKSkgJT4lDQogICMgZ2dwbG90KGFlcyh5PWludGVuc2l0eSwgeD1jb25kaXRpb24sIGdyb3VwPWNvbmRpdGlvbikpICsNCiAgZ2dwbG90KGFlcyh5PWludGVuc2l0eSwgeD1uYW1lLCBmaWxsPWNvbmRpdGlvbikpICsNCiAgZ2VvbV9ib3hwbG90KGFlcyhmaWxsPWZpbGwsIGNvbG91cj1jb2xvdXIpKSArDQogIHNjYWxlX2ZpbGxfaWRlbnRpdHkoKSArDQogIHNjYWxlX2NvbG9yX2lkZW50aXR5KCkgKw0KICB5bGFiKGV4cHJlc3Npb24oImludGVuc2l0eSAqIDEwIl4iIC0yIikpICsgDQogIHRoZW1lKHN0cmlwLnRleHQgPSBlbGVtZW50X3RleHQoZmFjZSA9ICJib2xkIiwgc2l6ZT0xMiksDQogICAgICAgIGF4aXMudGl0bGUueCA9IGVsZW1lbnRfYmxhbmsoKSwNCiAgICAgICAgYXhpcy50ZXh0LnggPSBlbGVtZW50X3RleHQoc2l6ZT0xMCkpICsNCiAgc2NhbGVfeF9kaXNjcmV0ZShsYWJlbHMgPSBmdW5jdGlvbih4KSBzdHJfd3JhcCh4LCB3aWR0aCA9IDE0LCB3aGl0ZXNwYWNlX29ubHkgPSBGQUxTRSkpICsNCiAgIyBmYWNldF93cmFwKH5uYW1lLCBzY2FsZT0iZnJlZSIsIG5jb2w9MikgKw0KICBzY2FsZV95X2NvbnRpbnVvdXMobGFiZWxzID0gZnVuY3Rpb24oeCkgZm9ybWF0KHggKiAxMDAsIHNjaWVudGlmaWMgPSBGQUxTRSksIA0KICAgICAgICAgICAgICAgICAgICAgbGltaXRzID0gYygwLDAuMDU1KSwgYnJlYWtzID0gZXh0ZW5kZWRfYnJlYWtzKG49OCkpIC0+IHBsb3Rfd24NCg0KDQppZGVudGlmaWVkX21ldGFib2xpdGVzICU+JSANCiAgZmlsdGVyKG5hbWUgJWluJSBjKCJMLUlzb2xldWNpbmUiLCAiQWNldHlsLUwtY2Fybml0aW5lIiwNCiAgICAgICAgICAgICAgICAgICAgICJCdXR5cnlsLUwtY2Fybml0aW5lIiwNCiAgICAgICAgICAgICAgICAgICAgICJJc292YWxlcnlsLUwtY2Fybml0aW5lIikpICU+JQ0KZmlsdGVyKGNvbmRpdGlvbiAlaW4lIGMoIlNEX1RGIiwgIldOX1RGIikpICU+JQ0KICAjIGdncGxvdChhZXMoeT1pbnRlbnNpdHksIHg9Y29uZGl0aW9uLCBncm91cD1jb25kaXRpb24pKSArDQogIGdncGxvdChhZXMoeT1pbnRlbnNpdHksIHg9bmFtZSwgZmlsbD1jb25kaXRpb24pKSArDQogIGdlb21fYm94cGxvdChhZXMoZmlsbD1maWxsLCBjb2xvdXI9Y29sb3VyKSkgKw0KICBzY2FsZV9maWxsX2lkZW50aXR5KCJDb25kaXRpb24iLCBsYWJlbHM9YygiV2ludGVyIE5pZ2h0IiwgIlN1bW1lciBEYXkiKSwgZ3VpZGUgPSAibGVnZW5kIikgKw0KICBzY2FsZV9jb2xvcl9pZGVudGl0eSgiQ29uZGl0aW9uIiwgbGFiZWxzPWMoIldpbnRlciBOaWdodCIsICJTdW1tZXIgRGF5IiksIGd1aWRlID0gImxlZ2VuZCIpICsNCiAgeWxhYihleHByZXNzaW9uKCJpbnRlbnNpdHkgKiAxMCJeIiAtMiIpKSArIA0KICB0aGVtZShzdHJpcC50ZXh0ID0gZWxlbWVudF90ZXh0KGZhY2UgPSAiYm9sZCIsIHNpemU9MTIpLA0KICAgICAgICBheGlzLnRpdGxlLnggPSBlbGVtZW50X2JsYW5rKCksDQogICAgICAgIGF4aXMudGV4dC54ID0gZWxlbWVudF90ZXh0KHNpemU9MTApKSArDQogIHNjYWxlX3hfZGlzY3JldGUobGFiZWxzID0gZnVuY3Rpb24oeCkgc3RyX3dyYXAoeCwgd2lkdGggPSAxNCwgd2hpdGVzcGFjZV9vbmx5ID0gRkFMU0UpKSArDQogICMgZmFjZXRfd3JhcCh+bmFtZSwgc2NhbGU9ImZyZWUiLCBuY29sPTIpICsNCiAgc2NhbGVfeV9jb250aW51b3VzKGxhYmVscyA9IGZ1bmN0aW9uKHgpIGZvcm1hdCh4ICogMTAwLCBzY2llbnRpZmljID0gRkFMU0UpLCANCiAgICAgICAgICAgICAgICAgICAgIGxpbWl0cyA9IGMoMCwwLjA1NSksIGJyZWFrcyA9IGV4dGVuZGVkX2JyZWFrcyhuPTgpKSAtPiBwbG90X3NkDQoNCnBsb3RfYWxsIDwtIGdnYXJyYW5nZShwbG90X3NkLCBwbG90X3duLCAgDQogICAgICAgICAgICAgICAgICAgICAgbGFiZWxzID0gYygiQSIsICJCIiksIA0KICAgICAgICAgICAgICAgICAgICAgIGNvbW1vbi5sZWdlbmQ9VFJVRSwNCiAgICAgICAgICAgICAgICAgICAgICBsZWdlbmQ9ImJvdHRvbSIsDQogICAgICAgICAgICAgICAgICAgICAgbnJvdz0xLA0KICAgICAgICAgICAgICAgICAgICAgIHdpZHRocyA9IGMoNCwgNikpDQoNCnByaW50KHBsb3RfYWxsKQ0KDQpnZ3NhdmUocGxvdCA9IHBsb3RfYWxsLCBmaWxlbmFtZSA9ICIuLi9maWd1cmVzL0ZpZ3VyZV8zLnRpZmYiLCBkcGkgPSAzMDAsIHdpZHRoID0gMTIsIGhlaWdodCA9IDYsIGJnPSJ3aGl0ZSIpDQpgYGANCg0KIyMjIFNlc3Npb24gaW5mb3JtYXRpb24NCg0KYGBge3IgU2Vzc2lvbl9pbmZvfQ0Kc2Vzc2lvbkluZm8oKQ0KYGBgDQo=
